# 37th International Symposium on Intensive Care and Emergency Medicine (part 2 of 3): Brussels, Belgium. 21-24 March 2017

**DOI:** 10.1186/s13054-017-1630-4

**Published:** 2017-03-21

**Authors:** D. Rob, R. Špunda, J. Lindner, J. Šmalcová, O. Šmíd, T. Kovárník, A. Linhart, J. Bìlohlávek, M. M. Marinoni, G. Cianchi, S. Trapani, M. L. Migliaccio, L. Gucci, M. Bonizzoli, A. Cramaro, M. Cozzolino, S. Valente, A. Peris, E. Grins, E. Kort, M. Weiland, N. Manandhar Shresta, P. Davidson, L. Algotsson, S. Fitch, G. Marco, J. Sturgill, S. Lee, M. Dickinson, T. Boeve, A. Khaghani, P. Wilton, S. Jovinge, A. N. Ahmad, R. Loveridge, S. Vlachos, S. Patel, E. Gelandt, L. Morgan, S. Butt, M. Whitehorne, V. Kakar, C. Park, M. Hayes, C. Willars, T. Hurst, T. Best, A. Vercueil, G. Auzinger, B. Adibelli, N. Akovali, A. Torgay, P. Zeyneloglu, A. Pirat, Z. Kayhan, S. S. Schmidbauer, J. Herlitz, T. Karlsson, H. Friberg, R. Knafelj, P. Radsel, F. Duprez, T. Bonus, G. Cuvelier, S. Mashayekhi, M. Maka, S. Ollieuz, G. Reychler, R. Mosaddegh, S. Abbasi, S. Talaee, V. Z. Zotzmann, D. S. Staudacher, T. W. Wengenmayer, D. D. Dürschmied, C. B. Bode, A. Nelskylä, J. Nurmi, M. Jousi, A. Schramko, E. Mervaala, G. Ristagno, M. Skrifvars, G. Ozsoy, T. Kendirli, E. Azapagasi, O. Perk, U. Gadirova, E. Ozcinar, M. Cakici, C. Baran, S. Durdu, A. Uysalel, M. Dogan, M. Ramoglu, T. Ucar, E. Tutar, S. Atalay, R. Akar, M. Kamps, G. Leeuwerink, J. Hofmeijer, O. Hoiting, J. Van der Hoeven, C. Hoedemaekers, A. Konkayev, V. Kuklin, T. Kondratyev, M. Konkayeva, N. Akhatov, M. Sovershaev, T. Tveita, V. Dahl, L. Wihersaari, M. B. Skrifvars, S. Bendel, K. M. Kaukonen, J. Vaahersalo, J. Romppanen, V. Pettilä, M. Reinikainen, A. Lybeck, T. Cronberg, N. Nielsen, H. Friberg, M. Rauber, K. Steblovnik, A. Jazbec, M. Noc, P. Kalasbail, F. Garrett, E. Kulstad, D. J. Bergström, H. R. Olsson, S. Schmidbauer, H. Friberg, I. Mandel, S. Mikheev, Y. Podoxenov, I. Suhodolo, A. Podoxenov, J. Svirko, A. Sementsov, L. Maslov, V. Shipulin, L. V. Vammen, S. R. Rahbek, N. S. Secher, J. P. Povlsen, N. J. Jessen, B. L. Løfgren, A. G. Granfeldt, A. Grossestreuer, S. Perman, P. Patel, S. Ganley, J. Portmann, M. Cocchi, M. Donnino, Y. Nassar, S. Fathy, A. Gaber, S. Mokhtar, Y. C. Chia, R. Lewis-Cuthbertson, K. Mustafa, A. Sabra, A. Evans, P. Bennett, W. Eertmans, C. Genbrugge, W. Boer, J. Dens, C. De Deyne, F. Jans, A. Skorko, M. Thomas, M. Casadio, A. Coppo, A. Vargiolu, J. Villa, M. Rota, L. Avalli, G. Citerio, J. B. Moon, J. H. Cho, C. W. Park, T. G. Ohk, M. C. Shin, M. H. Won, P. Papamichalis, V. Zisopoulou, E. Dardiotis, S. Karagiannis, D. Papadopoulos, T. Zafeiridis, D. Babalis, A. Skoura, I. Staikos, A. Komnos, S. Silva Passos, F. Maeda, L. Silva Souza, A. Amato Filho, T. Araújo Guerra Granjeia, M. Schweller, D. Franci, M. De Carvalho Filho, T. Martins Santos, P. De Azevedo, R. Wall, I. Welters, P. Tansuwannarat, P. Sanguanwit, T. Langer, M. Carbonara, A. Caccioppola, C. Ferraris Fusarini, E. Carlesso, E. Paradiso, M. Battistini, E. Cattaneo, F. Zadek, R. Maiavacca, N. Stocchetti, A. Pesenti, A. Ramos, F. Acharta, J. Toledo, M. Perezlindo, L. Lovesio, A. Dogliotti, C. Lovesio, N. Schroten, B. Van der Veen, M. C. De Vries, J. Veenstra, Y. B. Abulhasan, S. Rachel, M. Châtillon-Angle, N. Alabdulraheem, I. Schiller, N. Dendukuri, M. Angle, C. Frenette, S. Lahiri, K. Schlick, S. A. Mayer, P. Lyden, M. Akatsuka, J. Arakawa, M. Yamakage, J. Rubio, J. A. Rubio Mateo-Sidron, R. Sierra, M. Celaya, L. Benitez, S. Alvarez-Ossorio, J. Rubio, J. A. Rubio Mateo-Sidron, R. Sierra, A. Fernandez, O. Gonzalez, H. Engquist, E. Rostami, P. Enblad, J. Toledo, A. Ramos, F. Acharta, L. Canullo, J. Nallino, A. Dogliotti, C. Lovesio, M. Perreault, J. Talic, A. J. Frenette, L. Burry, F. Bernard, D. R. Williamson, D. Adukauskiene, J. Cyziute, A. Adukauskaite, L. Malciene, L. Luca, A. Rogobete, O. Bedreag, M. Papurica, M. Sarandan, C. Cradigati, S. Popovici, C. Vernic, D. Sandesc, V. Avakov, I. Shakhova, H. Trimmel, M. Majdan, G. H. Herzer, C. S. Sokoloff, M. Albert, D. Williamson, C. Odier, J. Giguère, E. Charbonney, F. Bernard, Z. Husti, T. Kaptás, Z. Fülep, Z. Gaál, M. Tusa, J. Donnelly, M. Aries, M. Czosnyka, C. Robba, M. Liu, A. Ercole, D. Menon, P. Hutchinson, P. Smielewski, R. López, J. Graf, J. M. Montes, M. Kenawi, A. Kandil, K. Husein, A. Samir, J. Heijneman, J. Huijben, F. Abid-Ali, M. Stolk, J. Van Bommel, H. Lingsma, M. Van der Jagt, R. C. Cihlar, G. Mancino, P. Bertini, F. Forfori, F. Guarracino, D. Pavelescu, I. Grintescu, L. Mirea, S. Alamri, M. Tharwat, N. Kono, H. Okamoto, H. Uchino, T. Ikegami, T. Fukuoka, M. Simoes, E. Trigo, P. Coutinho, J. Pimentel, A. Franci, D. Basagni, M. Boddi, M. Cozzolino, V. Anichini, A. Cecchi, A. Peris, D. Markopoulou, K. Venetsanou, I. Papanikolaou, T. Barkouri, D. Chroni, I. Alamanos, E. Cingolani, M. G. Bocci, L. Pisapia, A. Tersali, S. L. Cutuli, V. Fiore, A. Palma, G. Nardi, M. Antonelli, R. Coke, A. Kwong, D. J. Dwivedi, M. Xu, E. McDonald, J. C. Marshall, A. E. Fox-Robichaud, E. Charbonney, P. C. Liaw, I. Kuchynska, I. R. Malysh, L. V. Zgrzheblovska, L. Mestdagh, E. F. Verhoeven, I. Hubloue, J. Ruel-laliberte, R. Zarychanski, F. Lauzier, P. Lessard Bonaventure, R. Green, D. Griesdale, R. Fowler, A. Kramer, D. Zygun, T. Walsh, S. Stanworth, C. Léger, A. F. Turgeon, D. M. Baron, J. Baron-Stefaniak, G. C. Leitner, R. Ullrich, O. Tarabrin, A. Mazurenko, Y. Potapchuk, D. Sazhyn, P. Tarabrin, O. Tarabrin, A. Mazurenko, Y. Potapchuk, D. Sazhyn, P. Tarabrin, A. González Pérez, J. Silva, V. Artemenko, A. Bugaev, I. Tokar, S. Konashevskaya, I. M. Kolesnikova, E. V. Roitman, T. Rengeiné Kiss, Z. Máthé, L. Piros, E. Dinya, E. Tihanyi, A. Smudla, J. Fazakas, R. Ubbink, P. Boekhorst te, E. Mik, L. Caneva, G. Ticozzelli, S. Pirrelli, D. Passador, F. Riccardi, F. Ferrari, E. M. Roldi, M. Di Matteo, I. Bianchi, G. A. Iotti, G. Zurauskaite, A. Voegeli, M. Meier, D. Koch, S. Haubitz, A. Kutz, M. Bargetzi, B. Mueller, P. Schuetz, G. Von Meijenfeldt, M. Van der Laan, C. Zeebregts, K. B. Christopher, P. Vernikos, T. Melissopoulou, G. Kanellopoulou, M. Panoutsopoulou, D. Xanthis, K. Kolovou, T. Kypraiou, J. Floros, H. Broady, C. Pritchett, M. Marshman, N. Jannaway, C. Ralph, C. L. Lehane, C. K. Keyl, E. Z. Zimmer, D. T. Trenk, A. S. Ducloy-Bouthors, M. J. Jonard, F. Fourrier, F. Piza, T. Correa, A. Marra, J. Guerra, R. Rodrigues, A. Vilarinho, V. Aranda, S. Shiramizo, M. R. Lima, E. Kallas, A. B. Cavalcanti, M. Donoso, P. Vargas, J. Graf, J. McCartney, S. Ramsay, K. McDowall, I. Novitzky-Basso, C. Wright, M Grgic Medic, L Bielen, V Radonic, O Zlopasa, N Gubarev Vrdoljak, V Gasparovic, R Radonic, G. Narváez, D. Cabestrero, L. Rey, M. Aroca, S. Gallego, J. Higuera, R. De Pablo, L. Rey González, G. Narváez Chávez, J. Higuera Lucas, D. Cabestrero Alonso, M. Aroca Ruiz, L. Jaramillo Valarezo, R. De Pablo Sánchez, A. Quinza Real, T. W. Wigmore, I. Bendavid, J. Cohen, I. Avisar, I. Serov, I. Kagan, P. Singer, J Hanison, U Mirza, D Conway, A. Takasu, H. Tanaka, N. Otani, S. Ohde, S. Ishimatsu, F Coffey, P Dissmann, K Mirza, M Lomax, P. Dissmann, F. Coffey, K. Mirza, M. Lomax, JR Miner, R Leto, AM Markota, PG Gradišek, VA Aleksejev, AS Sinkovič, S. Romagnoli, C. Chelazzi, G. Zagli, F. Benvenuti, P. Mancinelli, P. Boninsegni, L. Paparella, A. T. Bos, O. Thomas, T. Goslar, R. Knafelj, M. Perreault, A. Martone, P. R. Sandu, V. A. Rosu, A. Capilnean, P. Murgoi, A. J. Frenette, A. Lecavalier, D. Jayaraman, P. Rico, P. Bellemare, C. Gelinas, D. Williamson, T. Nishida, T. Kinoshita, N. Iwata, K. Yamakawa, S. Fujimi, L. Maggi, F. Sposato, G. Citterio, C. Bonarrigo, M. Rocco, V. Zani, R. A. De Blasi, D Alcorn, L Barry, M. A. Riedijk, D. M. Milstein, J. Caldas, R. Panerai, L. Camara, G. Ferreira, E. Bor-Seng-Shu, M. Lima, F. Galas, N. Mian, R. Nogueira, G. Queiroz de Oliveira, J. Almeida, J. Jardim, T. G. Robinson, F. Gaioto, L. A. Hajjar, I. Zabolotskikh, T. Musaeva, W. Saasouh, J. Freeman, A. Turan, S. Saseedharan, E. Pathrose, S. Poojary, J. Messika, Y. Martin, N. Maquigneau, M. Henry-Lagarrigue, C. Puechberty, A. Stoclin, L. Martin-Lefevre, F. Blot, D. Dreyfuss, A. Dechanet, D. Hajage, J. Ricard, E. Almeida, J. Almeida, G. Landoni, F. Galas, J. Fukushima, E. Fominskiy, C. De Brito, L. Cavichio, L. Almeida, U. Ribeiro, E. Osawa, R. Boltes, L. Battistella, L. Hajjar, P. Fontela, T. Lisboa, L. Forgiarini Junior, G. F. Friedman, F. Abruzzi, J. Azevedo Peixoto Primo, P. Marques Filho, J. Stormorvski de Andrade, K. Matos Brenner, M. Scorsato boeira, C. Leães, C. Rodrigues, A. Vessozi, A. SantAnna Machado, M. Weiler, H. Bryce, A. Hudson, T. Law, R. Reece-Anthony, A. Molokhia, F. Abtahinezhadmoghaddam, E. Cumber, L. Channon, A. Wong, R. Groome, D. Gearon, J. Varley, A. Wilson, J. Reading, A. Wong, F. G. Zampieri, F. A. Bozza, M. Ferez, H. Fernandes, A. Japiassú, J. Verdeal, A. C. Carvalho, M. Knibel, J. I. Salluh, M. Soares, J. Gao, E. Ahmadnia, B. Patel, J. McCartney, A. MacKay, S. Binning, C. Wright, R. J. Pugh, C. Battle, C. Hancock, W. Harrison, T. Szakmany, F. Mulders, J. Vandenbrande, J. Dubois, B. Stessel, K. Siborgs, D. Ramaekers, M. Soares, U. V. Silva, W. S. Homena, G. C. Fernandes, A. P. Moraes, L. Brauer, M. F. Lima, F. De Marco, F. A. Bozza, J. I. Salluh, N. Maric, M. Mackovic, N. Udiljak, CE Bosso, RD Caetano, AP Cardoso, OA Souza, R Pena, MM Mescolotte, IA Souza, GM Mescolotte, H. Bangalore, E. Borrows, D. Barnes, V. Ferreira, L. Azevedo, G. Alencar, A. Andrade, A. Bierrenbach, L. Tadini Buoninsegni, M. Bonizzoli, L. Cecci, M. Cozzolino, A. Peris, J. Lindskog, K. Rowland, P. Sturgess, A. Ankuli, A. Molokhia, R Rosa, T Tonietto, A Ascoli, L Madeira, W Rutzen, M Falavigna, C Robinson, J Salluh, A Cavalcanti, L Azevedo, R Cremonese, D Da Silva, A Dornelles, Y Skrobik, J Teles, T Ribeiro, C Eugênio, C Teixeira, M. Zarei, H. Hashemizadeh, M. Eriksson, G. Strandberg, M. Lipcsey, A. Larsson, M. Lignos, E. Crissanthopoulou, K. Flevari, P. Dimopoulos, A. Armaganidis, JG Golub, AM Markota, AS Stožer, AS Sinkovič, H. Rüddel, C. Ehrlich, C. M. Burghold, C. Hohenstein, J. Winning, W. Sellami, Z. Hajjej, M. Bousselmi, H. Gharsallah, I. Labbene, M. Ferjani, J. Sattler, D. Steinbrunner, H. Poppert, G. Schneider, M. Blobner, K. G. Kanz, S. J. Schaller, K. Apap, G. Xuereb, G. Xuereb, K. Apap, L. Massa, G. Xuereb, K. Apap, L. Massa, N. Delvau, A Penaloza, G Liistro, F Thys, I. K. Delattre, P. Hantson, P. M. Roy, P. Gianello, L Hadîrcă, A Ghidirimschi, N Catanoi, N Scurtov, M Bagrinovschi, Y. S. Sohn, Y. C. Cho, B. Golovin, O. Creciun, A. Ghidirimschi, M. Bagrinovschi, R. Tabbara, J. Z. Whitgift, A. Ishimaru, A. Yaguchi, N. Akiduki, M. Namiki, M. Takeda, J. N. Tamminen, M. Reinikainen, A. Uusaro, C. G. Taylor, E. D. Mills, A. D. Mackay, C. Ponzoni, R. Rabello, A. Serpa, M. Assunção, A. Pardini, G. Shettino, T. Corrêa, P. V. Vidal-Cortés, L. Álvarez-Rocha, P. Fernández-Ugidos, A. Virgós-Pedreira, M. A. Pérez-Veloso, I. M. Suárez-Paul, L. Del Río-Carbajo, S. Pita Fernández, A. Castro-Iglesias, A. Butt, A. A. Alghabban, S. K. Khurshid, Z. A. Ali, I. N. Nizami, N. S. Salahuddin, M. Alshahrani, A. W. Alsubaie, A. S. Alshamsy, B. A. Alkhiliwi, H. K. Alshammari, M. B. Alshammari, N. K. Telmesani, R. B. Alshammari, L. P. Asonto, F. G. Zampieri, L. P. Damiani, F Bozza, J. I. Salluh, A. B. Cavalcanti, A. El Khattate, M. Bizrane, N. Madani, J. Belayachi, R. Abouqal, D. Ramnarain, B. Gouw-Donders, C. Benstoem, A. Moza, P. Meybohm, C. Stoppe, R. Autschbach, D. Devane, A. Goetzenich, L. U. Taniguchi, L. Araujo, G. Salgado, J. M. Vieira, J. Viana, N. Ziviani, I. Pessach, A. Lipsky, A. Nimrod, M. O´Connor, I. Matot, E. Segal, A. Kluzik, A. Gradys, P. Smuszkiewicz, I. Trojanowska, M. Cybulski, A. De Jong, M. Sebbane, G. Chanques, S. Jaber, R. Rosa, C. Robinson, M. Bessel, L. Cavalheiro, L. Madeira, W. Rutzen, R. Oliveira, J. Maccari, M. Falavigna, E. Sanchez, F. Dutra, C. Dietrich, P. Balzano, J. Rezende, C. Teixeira, S. Sinha, K. Majhi, J. G. Gorlicki, F. P. Pousset, J. Kelly, J. Aron, A. Crerar Gilbert, N. Prevec Urankar, R. Knafelj, M. Irazabal, M. Bosque, J. Manciño, A. Kotsopoulos, N. Jansen, W. Abdo, Ú. M. Casey, B. O’Brien, R. Plant, B. Doyle

**Affiliations:** 1 First Faculty of Medicine, Charles University and General University Hospital, Prague, Czech Republic; 2 University of Florence, Florence, Italy; 3 Careggi Teaching Hospital, Florence, Italy; 4 Scania Univ Hospital Lund, Lund, Sweden; 5 Spectrum Health Hospitals, Grand Rapids, MI USA; 6 Van Andel Institute, Grand Rapids, MI USA; 7 King´s College Hospital, London, UK; 8 Ankara Baskent Hospital, Ankara, Turkey; 9 Skåne University Hospital, Lund, Sweden; 10 PreHospen, University of Borås, The Pre-hospital Research Centre of Western Sweden, Borås, Sweden; 11 Health Metrics, Institute of Medicine, Sahlgrenska Academy, University of Gothenburg, Gothenburg, Sweden; 12 Rihard Knafelj, Ljubljana, Slovenia; 13 Epicura, Hornu, Belgium; 14 Condorcet, Tournai, Belgium; 15 UCL, Bruxelles, Belgium; 16 Iran University of Medical Sciences, Tehran, Iran; 17 Heart Center, Freiburg, Germany; 18 Helsinki University Hospital, Helsinki, Finland; 19 Helsinki University Hospital and University of Helsinki, Helsinki, Finland; 20 Istituto di Ricerche Farmacologiche “Mario Negri”, Milan, Italy; 21 Ankara Univercity, Ankara, Turkey; 22 Radboudumc, Nijmegen, Netherlands; 23 Rijnstate, Arnhem, Netherlands; 24 Canisius Wilhelmina Ziekenhuis, Nijmegen, Netherlands; 25 Astana Medical University, Astana, Kazakhstan; 26 Akershus University Hospital, Oslo, Norway; 27 The Arctic University of Norway, Tromsø, Norway; 28 University Hospital of Northern Norway, Tromsø, Norway; 29 North Karelia Central Hospital, Joensuu, Finland; 30 Australian and New Zealand Intensive Care Research Centre, School of Public Health and Preventive Medicine, Monash University, Melbourne, Australia; 31 Kuopio University Hospital, Kuopio, Finland; 32 Helsinki University and Helsinki University Hospital, Helsinki, Finland; 33 Eastern Finland Laboratory Centre, Kuopio, Finland; 34 Skane University Hospital, Lund University, Lund, Sweden; 35 Helsingborg Hospital, Lund University, Helsingborg, Sweden; 36 University Medical Centre Ljubljana, Ljubljana, Slovenia; 37 Cleveland Clinic, Cleveland, OH USA; 38 Garrett Technologies, Northbrook, IL USA; 39 Advocate Christ Medical Center, Oak Lawn, IL USA; 40 Skåne University Hospital, Lund, Sweden; 41 City Clinical Hospital No 83 of FMBA of Russia, Moscow, Russia; 42 Cardiology Research Institute, Tomsk, Russia; 43 Siberian State Medical University, Tomsk, Russia; 44 Aarhus University Hospital, Aarhus C, Denmark; 45 Regional Hospital of Randers, Randers, Denmark; 46 Regional Hospital of Horsens, Horsens, Denmark; 47 Aarhus University, Aarhus, Denmark; 48 Beth Israel Deaconess Medical Center, Boston, MA USA; 49 University of Colorado, Denver, CO USA; 50 Cairo University, Giza, Egypt; 51 Tan Tock Seng Hospital, Singapore, Singapore; 52 Swansea University, Swansea, UK; 53 Abertawe Bro Morgannwg Health Board, Swansea, UK; 54 Ziekenhuis Oost-Limburg, Genk, Belgium; 55 Bristol Royal Infirmary, Bristol, UK; 56 University of Milano-Bicocca, Monza, Italy; 57 San Gerardo Hospital, Monza, Italy; 58 Kangwon National University, Chuncheonsi, South Korea; 59 General Hospital of Larissa, Larissa, Greece; 60 University Hospital of Larissa, Larissa, Greece; 61 University of Campinas, Campinas, Brazil; 62 Royal Liverpool University Hospital, Liverpool, UK; 63 Ramathibodi hospital, Bangkok, Thailand; 64 University of Milan, Milan, Italy; 65 Fondazione IRCCS Ca’ Granda, Ospedale Maggiore Policlinico, Milan, Italy; 66 Sanatorio Parque, Rosario, Argentina; 67 Grupo Oroño, Rosario, Argentina; 68 VUMC, Amsterdam, Netherlands; 69 OLVG, Amsterdam, Netherlands; 70 Montreal Neurological Institute and Hospital, Montreal, Canada; 71 McGill University, Montreal, Canada; 72 Cedars-Sinai Medical Center, Los Angeles, CA USA; 73 Mount Sinai Medical Center, New York, USA; 74 Japanese Red Cross Kitami Hospital, Kitami, Japan; 75 Sapporo Medical University School of Medicine, Sapporo, Japan; 76 Hospital Universitario Puerta del Mar, Cadiz, Spain; 77 Hospital Xanit, Benalmadena, Malaga, Spain; 78 Hospital Universitario Puerta del Mar, Cadiz, Spain; 79 Hospital Xanit, Benalmadena, Malaga, Spain; 80 Department of Neuroscience/Neurosurgery, Uppsala University, Uppsala, Sweden; 81 Sanatorio Parque, Rosario, Argentina; 82 Grupo Oroño, Rosario, Argentina; 83 The Montreal General Hospital, Montreal, Canada; 84 University of Geneva, Geneva, Switzerland; 85 Hôpital Sacré-Coeur de Montreal, Montreal, Canada; 86 Mount Sinai Hospital, Toronto, Canada; 87 Hospital of Lithuanian University of Health Sciences, Kaunas, Lithuania; 88 Lithuanian University of Health Sciences, Kaunas, Lithuania; 89 Innsbruck Medical University Hospital, Innsbruck, Austria; 90 Klaipeda University Hospital, Klaipeda, Lithuania; 91 University of Medicine and Pharmacy ”Victor Babes” Timisoara, Timisoara, Romania; 92 Emergency County Hospital ”Pius Brinzeu”, Clinic of Anesthesia and Intensive Care ”Casa Austria”, Timisoara, Romania; 93 Tashkent Medical Academy, Tashkent, Uzbekistan; 94 Université de Strasbourg, Strasbourg, France; 95 Landesklinikum Wr. Neustadt, Wiener Neustadt, Austria; 96 Department of Public Health, 91701 Trnava, Slovakia; 97 Hôpital du Sacré-Coeur de Montréal, Montréal, Canada; 98 Centre Hospitalier Universitaire de Montréal, Montreal, Canada; 99 Bács- Kiskun County Hospital, Kecskemét, Hungary; 100 Brain Physics Laboratory, Division of Neurosurgery, Department of Clinical Neurosciences, Cambridge Biomedical Campus, University of Cambridge, Cambridge, UK; 101 MUMC, Maastricht, Netherlands; 102 Division of Anaesthesia, Department of Medicine, Addenbrooke’s Hospital, University of Cambridge, Cambridge, UK; 103 Division of Neurosurgery, Department of Clinical Neurosciences, Addenbrooke’s Hospital, University of Cambridge, Cambridge, UK; 104 Clínica Alemana de Santiago, Santiago, Chile; 105 Cairo University Hospital, Cairo, Egypt; 106 Helal Hospital, Cairo, Egypt; 107 Erasmus Medical Center, Rotterdam, Netherlands; 108 Nemocnice Ceske Budejovice, C eske Budejovice, Czech Republic; 109 Anaesthesia And Intensive Care Department, Azienda Ospedaliero Universitaria Pisana, Pisa, Italy; 110 Emergency Hospital Floreasca, Bucharest, Romania; 111 Riyadh National Hospital, Riyadh, Saudi Arabia; 112 Kurashiki Central Hospital, Kurashiki City, Japan; 113 Centro Hospitalar e Universitario de Coimbra, Coimbra, Portugal; 114 Careggi Teaching Hospital, Florence, Italy; 115 Kat Hospital Athens, Kifisia, Greece; 116 Azienda Ospedaliera San Camillo Forlanini, Roma, Italy; 117 Fondazione A. Gemelli, Rome, Italy; 118 Infermi, Rimini, Italy; 119 McMaster University, Hamilton, Canada; 120 TaARI, DBRI, Hamilton, Canada; 121 St. Michael´s Hospital, Toronto, Canada; 122 Hamilton Health Sciences, Hamilton, Canada; 123 Hopital du Sacre-Coeur, Montreal, Canada; 124 Shupyk National Medical Academy, Kiev, Ukraine; 125 Uzbrussel, Brussel, Belgium; 126 Evert Verhoeven, Vorselaar, Belgium; 127 Université Laval, Québec, Canada; 128 University of Manitoba, Manitoba, Canada; 129 Dalhousie University, Halifax, Canada; 130 University of British Columbia, Vancouver, Canada; 131 University of Toronto, Toronto, Canada; 132 University of Calgary, Calgary, Canada; 133 University of Alberta, Edmonton, Canada; 134 University of Edinburgh, Edinburgh, UK; 135 University of Oxford, Oxford, UK; 136 Medical University of Vienna, Vienna, Austria; 137 Odessa National Medical University, Odessa, Ukraine; 138 Odessa National Medical University, Odessa, Ukraine; 139 Hospital Universitario Central de Asturias, Oviedo, Spain; 140 MC INTO-SANA, Odessa, Ukraine; 141 Pirogov Russian National Research Medical University, Moscow, Russia; 142 Semmelweis University, Budapest, Hungary; 143 Erasmus Medical Center Rotterdam, Rotterdam, Netherlands; 144 IRCCS Policlinico S. Matteo, SC Anestesia e Rianimazione 2, Pavia, Italy; 145 IRCCS Policlinico S.Matteo US Patologia Aorta Toracica-UOC Chirurgia Vascolare, Pavia, Italy; 146 Università degli Studi di Pavia, Pavia, Italy; 147 Kantonsspital Aarau, Aarau, Switzerland; 148 University of Groningen, Groningen, Netherlands; 149 Brigham and Women’s Hospital, Boston, MA USA; 150 Laiko General Hospital, Athens, Greece; 151 Royal Cornwall Hospital, Truro, UK; 152 Herzzentrum Freiburg Bad krozingen, Bad Krozingen, Germany; 153 CHRU, Lille, France; 154 Centre Hospitalier, Lens, France; 155 Hospital Israelita Albert Einstein, Sao Paulo, Brazil; 156 Universidade de Sao Paulo, Sao Paulo, Brazil; 157 Hospital do Coração, Sao Paulo, Brazil; 158 Clinica Alemana, Santiago, Chile; 159 Queen Elizabeth University Hospital, Glasgow, UK; 160 Beatson West of Scotland Cancer Centre, Glasgow, UK; 161 Clinical Hospital Zagreb, Zagreb, Croatia; 162 Emergency Medicine Center Sisak, Sisak, Croatia; 163 Hospital Universitario Ramón y Cajla, Madrid, Spain; 164 Hospital Ramón y Cajal, Madrid, Spain; 165 Hospital La Fe, Valencia, Spain; 166 Royal Marsden Hospital, London, UK; 167 Rabin Medical Center, Petah Tikva, Israel; 168 Manchester Royal Infirmary, Manchester, UK; 169 University of Manchester, Manchester, UK; 170 St.Luke´s International Hospital, Tokyo, Japan; 171 St. Luke´s International University Center for Clinical Epidemiology, Tokyo, Japan; 172 Nottingham University Hospitals NHS Trust, Nottingham, UK; 173 James Cook University Hospital, Middlesbrough, UK; 174 Colchester Hospital University Foundation NHS Trust, Colchester, UK; 175 Mundipharma Research Limited, Cambridge, UK; 176 Emergency Department, James Cook University Hospital, Middlesbrough, UK; 177 Emergency Department, Nottingham University Hospitals NHS Trust, Nottingham, UK; 178 Accident and Emergency Department, Colchester Hospital University Foundation NHS Trust, Colchester, UK; 179 Mundipharma Research Limited, Cambridge, UK; 180 Hennepin County Medical Center, Minneapolis, MN USA; 181 Derriford hospital PHNT, Plymouth, UK; 182 University Medical Centre Maribor, Maribor, Slovenia; 183 University Medical Centre Ljubljana, Ljubljana, Slovenia; 184 Osp. Azienda Ospedaliero Univ Careggi, Florence, Italy; 185 VieCuri MC Venlo, Venlo, Netherlands; 186 Rihard Knafelj, Ljubljana, Slovenia; 187 The Montreal General Hospital, Montreal, Canada; 188 Hôpital de Verdun, Verdun, Canada; 189 Hopital du Sacre-Coeur, Montreal, Canada; 190 Jewish General Hospital, Montreal, Canada; 191 Osaka General Medical Center, Osaka, Japan; 192 Campus Biomedico, Rome, Italy; 193 Azienda Ospedaliera Sant´Andrea, Università La Sapienza, Rome, Italy; 194 Royal Alexandra Hospital, Paisley, UK; 195 Academic Medical Center, Amsterdam, Netherlands; 196 Academic Medical Center, Oral & Maxillofacial Surgery, Amsterdam, Netherlands; 197 University of Sao Paulo, Sao Paulo, Brazil; 198 University of Leicester, Leicester, UK; 199 Kuban State Medical University, Krasnodar, Russia; 200 Cleveland Clinic Foundation, Cleveland, OH USA; 201 Respiratory Motion, Inc, Waltham, MA USA; 202 S L Raheja Hospital, Mumbai, India; 203 Hôpital Louis Mourier, Colombes, France; 204 CHD de Vendée, La Roche-sur-Yon, France; 205 Gustave Roussy, Villejuif, France; 206 Instituto do Cancer, Sao Paulo, Brazil; 207 IRCCS San Raffaele Scientific Institute, Milan, Italy; 208 Academician EN Meshalkin Novosibirsk State Budget Research Institute of Circulation Pathology, Novosibirsk, Russia; 209 UFRGS, Porto Alegre, Brazil; 210 Centro Universitário Metodista - IPA, Porto Alegre, Brazil; 211 Hospital Ernesto Dornelles, Porto Alegre, Brazil; 212 Hospital Mae de Deus, Porto Alegre, Brazil; 213 University Hospital Lewisham, London, UK; 214 Shiraz University of Medical Sciences (SUMS), Shiraz, Iran; 215 John Radcliffe Hospital, Oxford, UK; 216 Addenbrooke’s Hospital, Cambridge University Hospitals NHS Foundation Trust, Cambridge, UK; 217 John Radcliffe Hospital, Oxford, UK; 218 HCor-Hospital of the Heart, São Paulo, Brazil; 219 D’Or Institute for Research and Education, Rio de Janeiro, Brazil; 220 Hospital São Francisco, Riberão Preto, Brazil; 221 Hospital São Luiz Brasil, São Paulo, Brazil; 222 Rede Amil, Rio de Janeiro, Brazil; 223 Hospital Barra D’Or, Rio de Janeiro, Brazil; 224 UDI Hospital, São Luís, Brazil; 225 Hospital São Lucas Copacabana, Rio de Janeiro, Brazil; 226 Royal Brompton Hospital, London, UK; 227 Queen Elizabeth University Hospital, Glasgow, UK; 228 Glan Clwyd Hospital, Rhyl, UK; 229 Morriston Hospital, Swansea, UK; 230 Public Health Wales, Cardiff, UK; 231 Cardiff University, Cardiff, UK; 232 Jessa Hospitals, Hasselt, Belgium; 233 DOr Institute for Research and Education - IDOR, Rio De Janeiro, Brazil; 234 Hospital de Câncer de Barretos, Barretos, Brazil; 235 Hospital Barra DOr, Rio de Janeiro, Brazil; 236 Santa Casa de Misericórdia de Juiz de Fora, Juiz de Fora, Brazil; 237 Hospital de Câncer do Maranhão Dr Tarquinio Lopes Filho, Sao Luis, Brazil; 238 Hospital São Luiz Itaim, Sao Paulo, Brazil; 239 Hospital Esperança Recife, Recife, Brazil; 240 Hospital viValle, Sao Jose dos Campos, Brazil; 241 Clinical Hospital Sveti Duh, Zagreb, Croatia; 242 Instituto do Coração de Presidente Prudente, Presidente Prudente, Brazil; 243 Universidade do Oeste Paulista, Presidente Prudente, Brazil; 244 Great Ormond Street Hospital & St Andrews Burns Centre, Chelmsford, UK; 245 St Andrew´s Burns Centre, Chelmsford, UK; 246 Research and Education Institute, Hospital Sirio-Libanes, São Paulo, Brazil; 247 University of Sao Paulo, Sao Paulo, Brazil; 248 Federal University of Goias, Goiania, Brazil; 249 Careggi Teaching Hospital, Florence, Italy; 250 Lewisham & Greenwich NHS Trust, London, UK; 251 Hospital Moinhos de Vento, Porto Alegre, Brazil; 252 Instituto D´Or de Pesquisa e Ensino, Rio de Janeiro, Brazil; 253 Hospital do Coração, São Paulo, Brazil; 254 Universidade de São Paulo, São Paulo, Brazil; 255 McGill University, Montreal, Canada; 256 Hospital Universitário de Goiânia, Goiânia, Brazil; 257 North Khorasan University of Medical Sciences, Bojnurd, Iran; 258 Quchan Branch, Islamic Azad University, Quchan, Iran; 259 Surgical Sciences, Uppsala, Sweden; 260 Medical Sciences, Uppsala, Sweden; 261 Attiko University Hospital, Haidari, Greece; 262 Beng MSc in Bioengineering, Athens, Greece; 263 University Medical Centre Maribor, Maribor, Slovenia; 264 University of Maribor, Maribor, Slovenia; 265 Jena University Hospital, Jena, Germany; 266 Military Hospital of Tunis, Tunis, Tunisia; 267 Klinikum Rechts der Isar der TUM, Munich, Germany; 268 Branddirektion München, Munich, Germany; 269 Mater Dei Hospital, Msida, Malta; 270 Mater Dei Hospital, L-Imsida, Malta; 271 Mater Dei Hospital, L-Imsida, Malta; 272 Cliniques Universitaires Saint-Luc, Brussels, Belgium; 273 GHdC, Charleroi, Belgium; 274 UCL, LTAP, Brussels, Belgium; 275 CHU, Angers, France; 276 UCL, CHEX, Brussels, Belgium; 277 National Centre of Prehospital Emergency Medicine, Chisinau, Moldova; 278 State Medical and Pharmaceutical University “Nicolae Testemitanu”, Chisinau, Moldova; 279 Soon Chun Hyang University Hospital Seoul, Seoul, South Korea; 280 National Centre of Prehospital Emergency Medicine, Chisinau, Moldova; 281 Ministry of Healthcare, Chisinau, Moldova; 282 Queen Elizabeth Kings Lynn Hospital, Norfolk, UK; 283 Tokyo Women´s Medical University, Tokyo, Japan; 284 Kuopio University Hospital, Kuopio, Finland; 285 North Karelia Central Hospital, Joensuu, Finland; 286 NHS Greater Glasgow & Clyde, Glasgow, UK; 287 Hospital Israelita Albert Einstein, São Paulo, Brazil; 288 CHU Ourense, Ourense, Spain; 289 CHU A Coruña, A Coruña, Spain; 290 King Faisal Specialist Hospital & Research Center, Riyadh, Saudi Arabia; 291 Dammam University, Khobar, Saudi Arabia; 292 King Fahad Hospital of the University, Khobar, Saudi Arabia; 293 HCor-Hospital of the Heart, São Paulo, Brazil; 294 D’Or Institute for Research and Education, Rio de Janeiro, Brazil; 295 Ibn Sina University Hospital, Rabat, Morocco; 296 Elisabeth Tweesteden Hospital Tilburg, Tilburg, Netherlands; 297 University Hospital RWTH Aachen, Aachen, Germany; 298 University Hospital Frankfurt, Frankfurt, Germany; 299 National University of Ireland Galway, Galway, Ireland; 300 Research and Education Institute, Sao Paulo, Brazil; 301 Kunumi, Belo Horizonte, Brazil; 302 Sheba Medical Center, Tel-Hashomer, Israel; 303 Intensix, Predictive Critical Care LTD, Netanya, Israel; 304 Tel Aviv Medical Center, Tel-Aviv, Israel; 305 University of Chicago Medicine, Chicago, IL USA; 306 Assuta Medical Centers, Tel-Aviv, Israel; 307 Heliodor Swiecicki Clinical Hospital at the Karol Marcinkowski Medical University in Poznan, Poznan, Poland; 308 University of Medical Science, Poznan, Poland; 309 Montpellier University Hospital, Montpellier, France; 310 Hospital Moinhos de Vento, Porto Alegre, Brazil; 311 Apollo hospitals, Bhubaneswar, India; 312 Hôpital Lariboisière, Paris, France; 313 Hôpital Pitié-Salpêtrière, Paris, France; 314 St George’s Hospital, London, UK; 315 Rihard Knafelj, Ljubljana, Slovenia; 316 Hospital General de Catalunya, Barcelona, Spain; 317 Hospital Germans Trias i Pujol, Barcelona, Spain; 318 Elisabeth Tweesteden Hospital, Tilburg, Netherlands; 319 Dutch Transplant Foundation, Leiden, Netherlands; 320 Radboud UMC, Nijmegen, Netherlands; 321 Cork University Hospital, Cork, Ireland

## P171 The use of extracorporeal membrane oxygenation for ventricular septal rupture complicated by refractory cardiogenic shock

### D Rob, R Špunda, J Lindner, J Šmalcová, O Šmíd, T Kovárník, A Linhart, J Bìlohlávek

#### First Faculty of Medicine, Charles University and General University Hospital, Prague, Czech Republic, Prague, Czech Republic

**Introduction:** Ventricular septal rupture (VSR) is an unusual mechanical complication of myocardial infarction (MI) in the era of reperfusion therapy, but the mortality rate of patients who present with cardiogenic shock (CS) remains extremely high. Whereas current American and European guidelines recommend urgent surgical repair regardless of hemodynamic status, promising outcomes have been repeatedly reported with the use of circulatory support, enabling hemodynamic stabilization and delaying repair after consolidation of the infarct scar. Therefore, we analyzed our experience with the use of Veno-Arterial Extracorporeal Membrane Oxygenation (V-A ECMO) in post-infarction VSR.

**Methods:** We conducted a retrospective search of institutional database of all patients presenting with post-infarction VSR from January 2007 to June 2016. Data of 33 consecutive patients were retrospectively reviewed and analyzed.

**Results:** In our center, 7 out of 33 patients with post-MI VSR and refractory CS (despite vasopressor and intraaortic balloon pump therapy) received V-A ECMO support. V-A ECMO improved end-organ perfusion with lower lactate levels 24 hours after implantation (7.514 vs. 1.514, p < 0.005), normalized arterial pH (7.25 vs. 7.40, p < 0.036), improved mean arterial pressure (64 mm/Hg vs. 83 mm/Hg, p < 0.001) and lowered heart rate (115/min vs. 68/min, p < 0.001) in all patients. Mean duration of ECMO support was 12 days, 5 out of 7 patients underwent surgical repair, 4 were successfully weaned from ECMO, 3 survived 30 days and 2 survived more than 1 year. The most frequent complication (5 patients) as well as the cause of death (3 patients) was bleeding.

**Conclusions:** Our experience suggest that V-A ECMO support in patients with VSR and refractory CS improves end-organ perfusion, provides hemodynamic stabilization and increases time for cardiovascular team decision. Bleeding complications are an important limitation of this method.

## P172 Feasibility of cerebral circulatory arrest diagnosis by TCD in VA ECMO patients

### MM Marinoni^1^, G Cianchi^2^, S Trapani^2^, ML Migliaccio^2^, L Gucci^2^, M Bonizzoli^2^, A Cramaro^2^, M Cozzolino^2^, S Valente^2^, A Peris^2^

#### ^1^University of Florence, Florence, Italy; ^2^Careggi Teaching Hospital, Florence, Italy

**Introduction:** The aim of our study is to investigate the feasibility of Transcranial Doppler (TCD) in Veno-arterial (VA) ECMO patients for confirmation of Cerebral Circulatory Arrest (CCA) in Brain Death (BD) diagnosis.

BD can occur in VA ECMO patients [1] and TCD is an accepted technique for BD confirmation in many countries and also in Italy [2]. In these patients, presence of Intra-aortic Balloon Pump (IABP) and residual cardiac contractility can influence TCD patterns.[3,4]

**Methods:** In this monocentric retrospective study TCD was performed in 5 patients evolved to BD. Left Ventricular Ejection Fraction (LVEF) values and the presence or absence of IABP were taken into account.

**Results:** Haemodynamic conditions of the sample study are summarized in Table 1. TCD diagnostic patterns of CCA were found in all patients in all cerebral arteries. In 2 patients TCD evaluation was available before and in CCA.

**Conclusions:** In the case of BD, TCD seems to be a reliable instrumental test for CCA diagnosis in patients on VA ECMO treatment with a pulsatile flow (native or IABP support).

**References**

1. Pokersnik JA et al. J Card Surgery 27: 246–252, 2012

2. National Transplantation Council Guidelines, 2009

3. Yang F et al. J Transl Med 12:106, 2014

4. Kavi T et al. Journal of Stroke and Cerebrovascular Diseases, 2016

**Table 1 (abstract P172).** Haemodynamic and TCD findings of patients evolved to BD

Patient (Pt)	ECMO flow rate (L/min)	IABP (ratio)	LVEF (%)	TCD before CCA
Pt 1	3.0	NO	40	NO
Pt 2	2.97	NO	45	YES
Pt 3	3.1	NO	25	NO
Pt 4	3.9	YES (1:1)	45	YES
Pt 5	3.5	YES (1:1)	<20	NO

## P173 A dysbalance in t-cell response predicts in-house mortality in VA-ECMO patients

### E Grins^1^, E Kort^2^, M Weiland^3^, N Manandhar Shresta^2^, P Davidson^3^, L Algotsson^1^, S Fitch^2^, G Marco^2^, J Sturgill^2^, S Lee^2^, M Dickinson^2^, T Boeve^2^, A Khaghani^2^, P Wilton^2^, S Jovinge^2^

#### ^1^Scania Univ Hospital Lund, Lund, Sweden; ^2^Spectrum Health Hospitals, Grand Rapids, MI, United States; ^3^Van Andel Institute, Grand Rapids, MI, United States

**Introduction:** ECMO treatment has continuously been associated with high mortality. CD4/CD8 T-cell ratio has been used to monitor loss in immune function HIV patients and high ratio has been reported in allograft connected to worse outcome.

**Methods:** Patients (n = 51) eligible for VA ECMO treatment at Meijer Heart Centre in Grand Rapids Michigan were consented through by themselves or their Legal Representatives. Blood was drawn before the patient was cannulated.

**Results:** Non-survivors had higher CD4/CD8 p < 0.0266 than non-survivers. IN ROC regression CD4/CD8 ratio performed second best AUC 0.72

**Conclusions:** A more aggressive T-cell activation as reflected by CD4/CD8 ratio is related to mortality.

**Fig. 1 (abstract P173).**
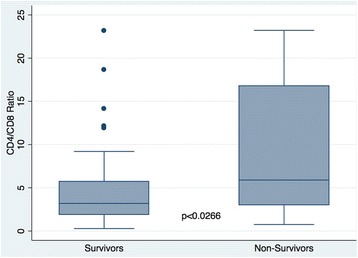
CD4/CD8 ratio Survivors vs Non-Survivors

**Fig. 2 (abstract P173).**
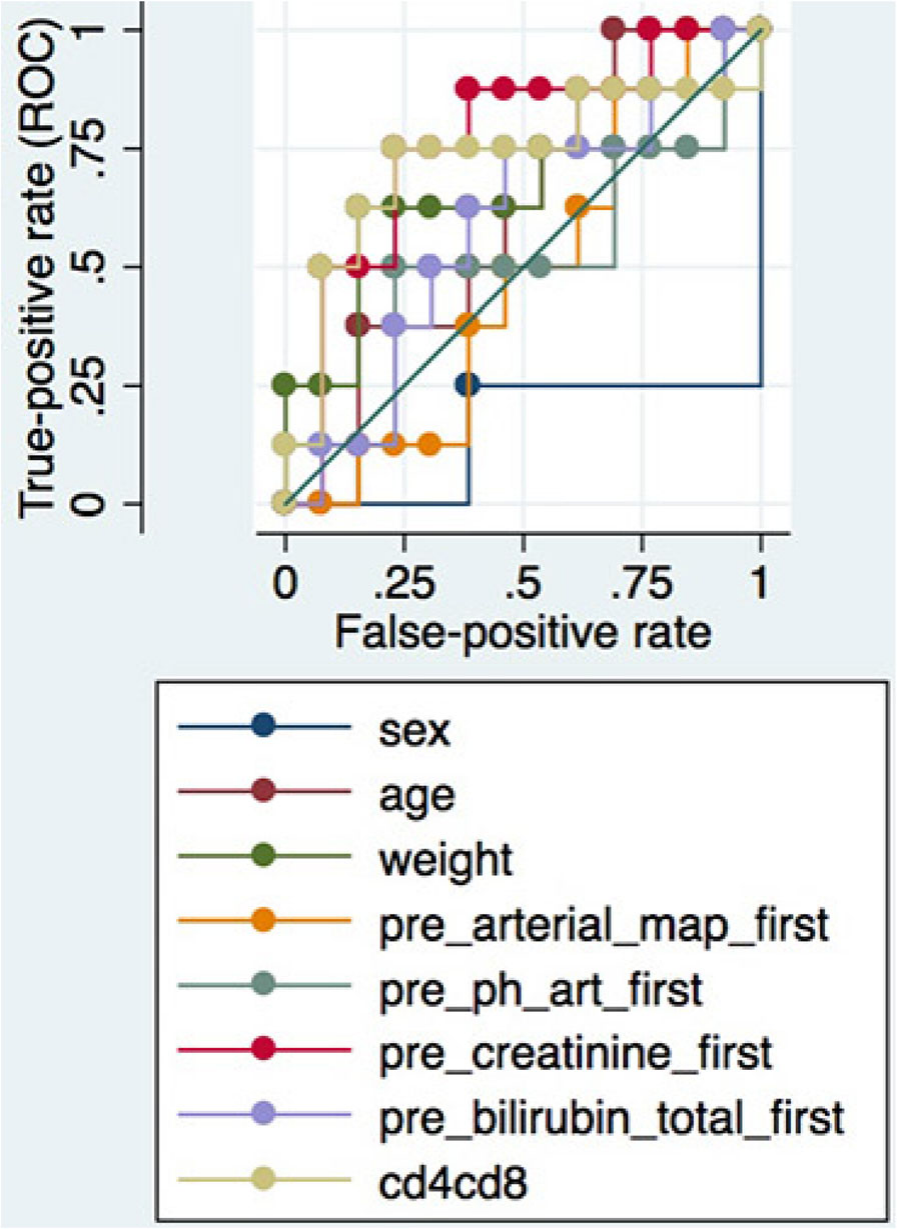
ROC Regression plot predictors of ECMO Mortality

## P174 ENCOURAGE…ing results for veno-arterial ECMO in myocardial infarction

### AN Ahmad, R Loveridge, S Vlachos, S Patel, E Gelandt, L Morgan, S Butt, M Whitehorne, V Kakar, C Park, M Hayes, C Willars, T Hurst, T Best, A Vercueil, G Auzinger

#### King´s College Hospital, London, United Kingdom

**Introduction:** The ENCOURAGE score is a mortality risk score for acute myocardial infarction (AMI) patients treated with veno-arterial extracorporeal membrane oxygenation (VA-ECMO) using pre-ECMO parameters. [1]

Only 41% of patients in the ENCOURAGE dataset survived six months with 20% of them requiring VAD or heart transplant.

This study aims to evaluate our 6-month outcomes against those predicted by the score.

**Methods:** All consecutive patients receiving VA-ECMO post AMI between 2012 and 2016 had data prospectively collected for analysis and an ENCOURAGE score calculated, using the seven parameters that comprise the score: age, sex, BMI, GCS, creatinine, lactate and prothrombin activity.

Patient outcomes using six-month follow-up data were compared and a standardized mortality ratio (SMR) calculated.

**Results:** 12 patients were included. Median ENCOURAGE score was 27 (IQR 6) with 83% in the worst risk classes. Predicted survival of our cohort according to the score was 18%.

42% were initiated as eCPR, 92% were after cardiac arrest (45% OHCA, 55% IHCA), mean lactate was 11.9 (SD + - 6.7), median SOFA 16 (IQR 4).

75% were ECMO survivors and 50% were alive at six months without VAD or transplantation, all with good Cerebral Performance Category scores.

The ENCOURAGE SMR is 0.611 (95% CI 0.25-1.27).

**Conclusions:** Risk prediction models that use pre-ECMO criteria can be used to assess the performance of ECMO centres [2,3] and this technique can be extended to sub-group analysis.

Previous studies have suggested poorer outcomes (36% [4], 21% [5]), in line with the ENCOURAGE dataset but the service exceeds predicted outcomes, and we recommend against restrictive criteria for the use of VA-ECMO.

The UK also needs to consider destination therapy for those surviving ECMO but not ICU.

**References**

[1] Muller G et al. Intensive care medicine. 2016;42(3):370–8.

[2] Chaddock et al. EuroELSO 2016

[3] Loveridge et al. Crit Care 20:94. 2016

[4] Boqambar et al. EuroELSO 2016

[5] Francis et al. EuroELSO 2016

## P175 Veno-arterial extracorporeal membrane oxygenation for cardiac support: a single center experience

### B Adibelli, N Akovali, A Torgay, P Zeyneloglu, A Pirat, Z Kayhan

#### Ankara Baskent Hospital, Ankara, Turkey

**Introduction:** Veno-arterial extracorporeal membrane oxygenation (VA ECMO) ensures end-organ perfusion while fully replacing heart and lung function to allow time for possible heart recovery or may bridge patients to heart transplantation or ventricular assist device (VAD) implantation. We review our 4-year experience regarding VA-ECMO use for cardiac support including early, midterm outcomes and survival.

**Methods:** This is a retrospective analysis of patients undergoing VA-ECMO for cardiac support from January 2012 to December 2015. ECMO was performed through the femoral vessels percutaneously with ultrasound guidance in 39 of them and through the right atrium to ascending aorta in 7 of them during cardiac surgery. VA-ECMO was applied during or after cardiac surgery in 30 (65%), not related to surgery in 16 (35%) out of 46 patients. Extracorporeal CPR was done in 6 patients.

**Results:** A total of 46 patients were supported with VA-ECMO. Mean age of patients was 47.7 ± 20.9 years with 67.7% males. The leading diagnoses were dilated cardiomyopathy, heart failure and coronary artery disease. Mean duration of VA-ECMO support was 226.9 ± 283.4 hours. Overall, 14 (30.4%) patients were successfully weaned off ECMO and survived, 32 (69.6%) patients died due to multifactorial complications. Overall, 12 patients were bridged to heart transplantation and 8 patients to left VAD. The 30-day and 6-month survival rates were 41% and 20%, respectively. Requirement for mechanical ventilation before and after VA-ECMO implantation was significantly less in patients who survived (p < 0.05).

**Conclusions:** In cases of cardiomyopathy refractory to medical treatment, failure to wean off cardiopulmonary bypass and refractory shock post cardiac arrest, circulation can be supported with VA-ECMO. Our experience among 46 patients implanted with VA-ECMO for cardiac support, survival to discharge was 30%. Survival was significantly better in those patients who did not require mechanical ventilation before and after VA-ECMO implantation.

## P176 Use of automated chest compression devices after out-of-hospital cardiac arrest in Sweden

### SS Schmidbauer^1^, J Herlitz^2^, T Karlsson^3^, H Friberg^1^

#### ^1^Skåne University Hospital, Lund, Lund, Sweden; ^2^PreHospen, University of Borås, The Pre-hospital Research Centre of Western Sweden, Borås, Sweden; ^3^Health Metrics, Institute of Medicine, Sahlgrenska Academy, University of Gothenburg, Gothenburg, Sweden

**Introduction:** Although shown not to increase survival rates, automated chest compression (ACC) devices are frequently utilised after out-of-hospital cardiac arrest (OHCA) in Sweden. With no implementation guidelines available, it is not known how these devices are put to use. In this retrospective observational study, we evaluated the utilisation of ACC devices in Sweden between the years 2011-2015. The association between ACC-CPR and 30-day survival was also assessed.

**Methods:** The Swedish Cardiopulmonary Resuscitation Registry is a prospectively recorded nationwide registry of modified Utstein-style parameters with a coverage of close to 100% of OHCA cases where resuscitation was attempted. Propensity score matching (PSM) and logistic regression with multiple imputation (MI) were used to study the association between ACC-CPR and survival.

**Results:** During the study period, 25898 patients were identified in the registry. After exclusions, 24316 were included in the study population. Of these, 32.4% received ACC-CPR. Overall, unadjusted 30-day-survival was 6.3% in the ACC-CPR group, 12.8% in the manual CPR group and 10.7% for the entire study population. Male gender and an initial shockable rhythm were factors associated with ACC device use, whereas crew witnessed status was associated with manual CPR. Administration of adrenaline and antiarrhythmics was also more prevalent in the ACC-CPR group, and so was intubation (Table 2).

The odds ratio for 30-day survival regarding ACC device utilisation was 0.72 (95% CI 0.62-0.84), p < .001 by means of PSM (n = 13922). Similar results were seen using stratification on the PS (n = 20633) as well as logistic regression with MI (data not shown).

**Conclusions:** Automated chest compression devices are frequently used after OHCA and predominantly so for patients with a more refractory condition. Their use might be associated with lower survival rates.

**Table 2 (abstract P176).** baseline characteristics and treatment data

	All patients (n = 24316)	ACC-CPR (n = 7877)	Manual CPR (n = 16439)	*p*
Age (years, 10th-90th decentile)	71 (48-87)	71 (48-87)	71 (48-88)	.10
Female sex (%)	33.6	31.4	34.6	<.001
Shockable rhythm (%)	22.3	24.3	21.3	<.001
Crew witnessed event (%)	14.6	10.9	16.4	<.001
Bystander witnessed event (%)	51.1	55.3	49.1	<.001
Adrenaline (%)	79.8	92.3	73.7	<.001
Antiarrhythmics (%)	11.5	15.1	9.7	<.001
Intubation (%)	33.9	38.9	31.5	<.001

## P177 Mechanical ventilation during CPR

### R Knafelj, P Radsel

#### Rihard Knafelj, Ljubljana, Slovenia

**Introduction:** Guidelines for mechanical ventilation (MV) during cardiopulmonary resuscitation suggest low frequency (f) with normal tidal volume (Vt) avoiding hyperventilation and hyperinflation. During inhospital cardiac arrest in patients that are already mechanically ventilated, optimal ventilatory strategy is not known. We hypothesized that using CPR ventilation mode results in better Vt and (f) compared to bag ventilation, volume (V-AC) or pressure (P-AC) ventilation.CPR mode has pre-configured settings (FiO2 1.0,PEEP 5/20 cmH2O, f 12/min), alarms are deactivated, ventilation is synchronized with chest compressions

**Methods:** 40 tests were performed. Ventilation with V-AC (450 mL, PEEP 5 cmH2O, max pressure limit 30 cmH2O) was set (Elisa 800,HL,Germany). After chest compressions started group 1remained in V-AC, group 2 was switched to bag ventilation, group 3 to BIPAP, group 4 to CPR mode. During chest compressions changes in settings but not in mode were allowed. Vt, f of ventilation and chest compressions rate were measured in all groups

**Results:** During CPRf of chests compression did not differ across groups (118 ± 4, 110 ± 12, 113 ± 10, 112 ± 8 group 1, 2, 3, 4 respectively). Vt were significantly lower in groups 1, 3 and 4 (87 ± 47, 48 ± 71, 268 ± 46 mL respectively) compared to group 2 (1139 ± 133, p < 0.005) due to reached high pressure limit (group 1, 3, 4) or excessive bagging (group 2). Group 4 received higher Vt compared to group 2 and 3 (p < 0.005). Ventilation f was higher in groups 1, 2 and 3 (18 ± 3, 21 ± 4, 20 ± 3 respectively) compared to group 4 (12 ± 0p, <0.005)

**Conclusions:** CPR ventilation mode prevents hypo/hyperventilation and hyperinflation compared to other modes. Better guidelines compliance was demonstrated for chest compression rate. Clinical impact of newly implemented CPR ventilation mode warrants further studies.

**Reference**

Soar J et al. ALCS. Resuscitation.2015.95:100–47

**Fig. 3 (abstract P177).**
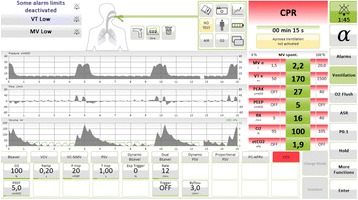
CPR mode

**Fig. 4 (abstract P177).**
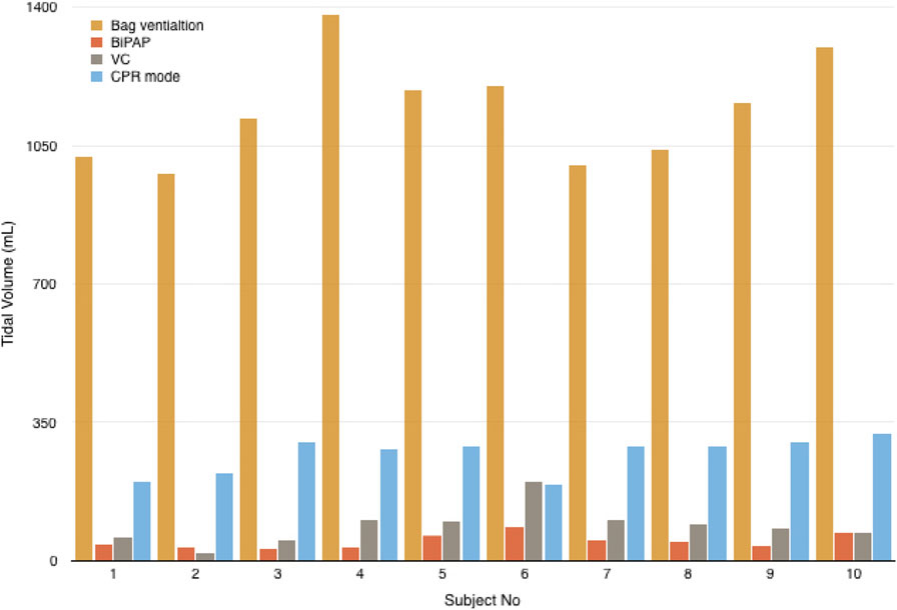
Vt during CPR

## P178 New system to control FDO2 with bag valve mask for premature infants

### F Duprez^1^, T Bonus^1^, G Cuvelier^2^, S Mashayekhi^1^, M Maka^1^, S Ollieuz^1^, G Reychler^3^

#### ^1^Epicura, Hornu, Belgium; ^2^Condorcet, Tournai, Belgium; ^3^UCL, Bruxelles, Belgium

**Introduction:** According to the recommendations of the European Resuscitation Council (ERC), Cardio Pulmonary Resuscitation (CPR) in premature infants must be made with a fraction of delivered oxygen (FDO[sub]2[/sub]) not exceeding 30%. Bag valve masks for premature infant (BVMp) can be used for ventilation and oxygenation during CPR. In such a case, even with a low oxygen flow rate (OFR), a BVMp delivers higher FDO[sub]2[/sub] than recommended. Indeed, with a BVMp, FDO[sub]2[/sub] rises proportionally to OFR but decreases inversely proportionally to minute ventilation (MV). Therefore, in neonatology resuscitation, controlling and maintaining the FDO[sub]2[/sub] below 30% is very difficult, even with a low OFR. To meet the ERC recommendations, we developed a new system aimed at delivering adequate FDO[sub]2[/sub] with a BVMp: the DupRey system. This system uses the Venturi effect to provides a stable air-oxygen mixture to a BVMp. The present study was aimed at evaluating the actual O[sub]2[/sub] fraction delivered between bag valve masks for premature infant used conventionally or used with the DupRey system.

**Methods:** On a bench study, a BVMp (Laerdal™ for premature infant type 850150) was connected to a test lung (Maquet™ VA800 - compliance 0,02 L/cm H[sub]2[/sub]O - resistance 20 cm H[sub]2[/sub]O/L/sec. With the BVMp, two MV (0.7 L/min and 1 L/min) were generated. A metronome gave the frequency of insufflations. The BVMp was tested both with and without oxygen reservoir (OR) and the pop off valve was closed. Two OFR: 0.6 and 1 L/min were analyzed and compared to the DupRey with Venturi 24% and 28% (OFR: 5 L/min). OFR were analyzed by a thermal mass flow meter Vogtlyn™ Red Y. The FDO[sub]2[/sub] and MV measurements were made using an analog iWorx® acquisition system (GA207 gas analyzer associated with digital IWx 214) and LabScribe II ® software.

Statistical: One Way Repeated Measures Analysis of Variance followed by Holm Sidak method.

**Results:** Statistical differences (p < .001) were found between A5-A6 / B5-B6 and all other columns. However, no statistical differences were found between: B2 and A2, A6 and B6, B5 and A5.

**Conclusions:** For an oxygen flow ranging from 0.6 to 1 L/min and two MV analyzed, a Bag Valve Masks for premature infant with an OR delivers very high FDO[sub]2[/sub] (>86%). Without OR, at same OFRs, FDO[sub]2[/sub] decreases but they maintain high values (>44%). The DupRey delivers FDO[sub]2[/sub] < to 30% regardless MV. The DupRey system is easily accessible for medical teams who do not have access to modern technology.

**References**

WyllieJ. ERC Guidelines for Resuscitation 2015: Resuscitation. 2015 Oct. 95: 249–63.

**Fig. 5 (abstract P178).**
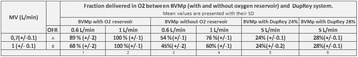
Fraction delivered in O2 between BVMp (with and without oxygen reservoir) and DupRey system

## P179 Could echocardiography have prognostic value in patients after successful resuscitation?

### R Mosaddegh, S Abbasi, S Talaee

#### Iran University of Medical Sciences, Tehran, Iran

**Introduction:** After successful cardio pulmonary resuscitation, many patients have poor outcome, because of current illness that causes cardiopulmonary arrest, or complications of resuscitation or any other problem. Researchers want to know if echocardiography have prognostic value in these patients.

**Methods:** In this case series the researchers enroll 50 patients with successful resuscitation in three general hospitals. Echocardiography was done for all patients without considering the duration of resuscitation in 24 hours after ROSC and ventricular and septal wall motion was observed for hypokinesia, akinesia or dyskinesia. Ejection Fraction (EF), E-Point Septal Separation (EPSS), Inferior Vena Cava (IVC) diameter and presence of Hepatic Portal Vein Gas (HPVG) and 24 hour's survival was measured. The relation between outcome and these echocardiographic findings were observed then.

**Results:** Twenty eight participants were survived and 22 were died. The median age of participants was 55.52 (SD: ±23.57) years. The mean EF for all participants was 26.74 ± 18.26 percent. The mean EF was 27.82 (SD ± 15.79) in survivors and 25.89 (SD ± 20.23) without statistically significant difference (P < 0.05). Ventricular wall motion, HPVG presence and IVC diameter has no statistically significant difference in both survivors and non survivors (P < 0.05).

**Conclusions:** Based on the results of this study it seems that echocardiographic findings in the first 24 hours of post resuscitation period could not help to predict the prognosis of survivors of cardio-pulmonary arrest, and it's not reasonable to send the post resuscitative patients to the other wards to do echocardiography in the first 24 hours after resuscitation.

## P180 Diagnostic and therapeutic value of coronary angiography and ct-scan after extracorporeal cardiopulmonary resuscitation (eCPR) – a single center registry study

### VZ Zotzmann, DS Staudacher, TW Wengenmayer, DD Dürschmied, CB Bode

#### Heart Center, Freiburg, Germany

**Introduction:** Implantation of a venoarterial extracorporeal membrane oxygenation (ECMO) in patients with ongoing cardiopulmonary resuscitation without return of spontaneous circulation (eCPR) can stabilize hemodynamics. Further diagnostic work up is needed in order to diagnose and treat the cause of the collapse. Patients after eCPR compromise a heterogeneous population with more severe underlying pathologies when compared to patients with return of spontaneous circulation. Aim of this study is the evaluation of the diagnostic value of coronary angiography and CT-scan after eCPR.

**Methods:** All patients after eCPR treated at a single tertiary referral hospital between December 2010 and November 2015 were included in a retrospective registry study.

**Results:** A total of 123 patients were considered (age 59.5 ± 15.3 years, low-flow time 59.0 ± 28.2 min, survival 11.4%). 52 patients presented with non-shockable rhythm (age 63.8 ± 16.1 years, low-flow duration 51.0 ± 23.1 min, survival 15.4%) while 71 patients presented with either a shockable rhythm or ST-elevation (age 56.3 ± 14.0 years, low-flow duration 64.8 ± 30.2 min, survival 8.5% p < .01, <.01 and .03, respectively).

**Fig. 6 (abstract P180).**
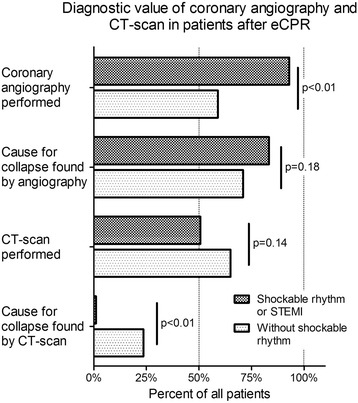
Diagnostic Of coronary angiography and CT-scan in patients after eCPR

Coronary angiography was performed significantly less frequent in patients with non-shockable rhythm (59.6% vs. 93.0% p < .01), see Fig. 6. A lesion deemed responsible for collapse however was found at similar rates in both groups (71.0% with non-shockable rhythm vs. 83.3% with ST-elevation or shockable rhythm, p = .18).

CT-scan was performed at similar rates in both groups (65.4% vs 50.7%, p = .14). Pathologies deemed responsible for collapse however were found more often in patients with non-shockable rhythm (23.5% vs. 0%, p < .01). CT-Scan yielded findings relevant to the further treatment frequently in both groups (92.2% vs 91.6%, p = 1). Cause of collapse could be detected by CT-scan at significantly lower rates when compared to coronary angiography in both groups (23.5% vs.71.0% and 0% vs. 83.3%, both p < .01).

**Conclusions:** Coronary angiography yielded a significantly better diagnostic value than CT-scan after eCPR disregarding initial rhythm or presence of ST-elevation. Considering the potential therapeutic option, a coronary angiography first approach might be preferable. A routine CT-scan however might be reasonable in all patients since findings relevant to the further treatment are frequent.

## P181 Comparison of FiO2 50% or 100% on brain oxygenation and cardiac mitochondrial function in experimental cardiac arrest

### A Nelskylä^1^, J Nurmi^2^, M Jousi^2^, A Schramko^1^, E Mervaala^2^, G Ristagno^3^, M Skrifvars^1^

#### ^1^Helsinki University Hospital, Helsinki, Finland; ^2^Helsinki University Hospital and University of Helsinki, Helsinki, Finland; ^3^Istituto di Ricerche Farmacologiche “Mario Negri”, Milan, Italy

**Introduction:** Guidelines advocate 100% oxygen during CPR. We hypothesized that 50% oxygen during CPR maintains cerebral oxygenation and compared to 100%, alleviates cardiac mitochondrial injury.

**Methods:** With Finnish National Animal Experiment Board (ESAVI/1077/04.10.07/2016) approval, ventricular fibrillation (VF) was induced electrically in anaesthetized adult pigs and left untreated for 7 minutes, followed by randomization to mechanical CPR (LUCAS) with 50% or 100% oxygen. Defibrillation was performed at 13 minutes and if unsuccessful, CPR continued for 20 minutes with defibrillation and 1 mg adrenaline every 2 minutes. Cerebral oxygenation was measured with near-infrared spectroscopy (rSO2, INVOS™ 5100C Cerebral Oximeter) and invasive brain tissue oxygen (pbO2) with an intraparenchymal probe (NEUROVENT-PTO, RAUMEDIC) in the frontal cortex. A heart biopsy was obtained 20 minutes after ROSC with analysis of mitochondrial respiration (OROBOROS Instruments Corp., Innsbruck, Austria) and compared to 4 control animals. Data are shown as mean with standard deviation (SD). Brain rSO2 and pbO2 were compared between groups over time with mixed linear model with mean arterial blood pressure (MAP) as covariate. Mitochondrial respiration was compared with analysis of variance.

**Results:** Of 20 pigs, one had a breach of protocol and were excluded leaving 9 in the 50% group and 10 in the 100% group. Groups were similar regarding rSO2 and pbO2 before CPR. With a median time of 15 minutes, 6 pigs achieved ROSC in the 50% group and 8 pigs in the 100% group. During resuscitation time was significantly associated with rSO2 (p < 0.001) and pbO2 (p < 0.001). The rSO2 (0.012) was lower with FiO2 50%, but pbO2 was not (p = 0.43). Mean arterial pressure was associated with pbO2 (p = 0.045). After ROSC the rSO2 (p < 0.001) and pbO2 (p < 0.001) increased significantly over time without difference between oxygen groups (p = 0.37, p = 0.18). Compared to controls mitochondrial respiration was decreased with adenosine diphosphate (ADP) levels of 57 (17) Pmol/sec/mg compared to 92 (23) Pmol/sec/mg (p = 0.008), no difference was seen between oxygen groups (p = 0.79).

**Conclusions:** Use of 50% oxygen during CPR results in lower oxygen content in blood, but brain tissue oxygenation can be maintained with efficient CPR. After ROSC brain oxygen increases rapidly. Cardiac arrest results in disturbed cardiac mitochondrial respiration but this is not alleviated with the use of 50% oxygen.

## P182 Pediatric extracorporeal cardiopulmonary resuscitation experiences of a pediatric intensive care unit

### G Ozsoy, T Kendirli, E Azapagasi, O Perk, U Gadirova, E Ozcinar, M Cakici, C Baran, S Durdu, A Uysalel, M Dogan, M Ramoglu, T Ucar, E Tutar, S Atalay, R Akar

#### Ankara Univercity, Ankara, Turkey

**Introduction:** Extracorporeal membrane oxygenation (ECMO) has been used during cardiopulmonary resuscitation (CPR) to improve the outcomes in selected patients. According to the international registry of the Extracorporeal Life Support Organization (ELSO), more than 1232 (41%) children have benefited from ECMO-assisted CPR (ECPR) since its introduction. We want to summarize our ECPR experiences in our unit.

**Methods:** This is a retrospective study performed in the 15-bed tertiary Pediatric Intensive Care Unit (PICU) at Ankara University Hospital. The children who underwent ECPR from September 2014 to August 2016 were assessed.

**Results:** Eight children underwent ECPR in our unit. Their median age and weight were 80.5 months and 35 kilograms respectively. They were all hospital-arrest. Their primary diseases were 62.5% cardiac and 37.5% non-cardiac. The causes of cardiac arrest were dysrhythmia (37.5%), heart failure (25%), sepsis (12.5%), bleeding (12.5%), low cardiac output syndrome (12.5%) and airway disease (12.5%). ECPR was performed in PICU (6) and in operation room (2). ECMO was started in the 78 minutes (median) of CPR. The time of ECPR was 85 minute (median). ECMO cannulas were placed in femoral vein-femoral artery (5), central (2), and internal jugular vein-carotid artery (1). The cannulas were not able to place in two patients. ECMO did not work in two patients which cannulated central and femoral vein-femoral artery. ECMO succeeded in 4 (50%) patients. The range of the ECMO time after ECPR was 12 hours and 9 days.

**Conclusions:** ECPR improves survival after cardiac arrest, especially in the patients who might benefit from this treatment.

## P183 Midazolam is an independent risk factor for prolonged awakening after cardiac arrest

### M Kamps^1^, G Leeuwerink^2^, J Hofmeijer^2^, O Hoiting^3^, J Van der Hoeven^1^, C Hoedemaekers^1^

#### ^1^Radboudumc, Nijmegen, Netherlands; ^2^Rijnstate, Arnhem, Netherlands; ^3^Canisius Wilhelmina Ziekenhuis, Nijmegen, Netherlands

**Introduction:** Neurologic prognostication after cardiac arrest is a delicate process with lots of confounders that influence the outcome of these patients. Sedation is known as one of the major confounders. We assessed the hypothesis that prolonged awakening after cardiac arrest is mainly caused by the use of long-acting sedation.

**Methods:** This is a retrospective, multicentre, cohort study. We studied patients with ROSC after cardiac arrest in three different hospitals. We studied variables such as the cumulative dosage of sedatives, time of awakening after cessation of sedation, renal function, targeted temperature and GOS score after six months. Renal function was defined as the RIFLE score at admission. Early awakening was defined as awakening within 48 hours after cessation of sedation. The Glasgow coma scale was used to score awakening, with a motor score of 6 with an eye score of 3 or 4 defined as awakening. The patients with good neurologic outcome were divided in an early and late awakening group. Good neurologic outcome was defined as a GOS score of 4 or 5 after six months.

**Results:** We studied 122 patients with a good neurologic outcome after six months. Demographic variables such as age, weight, ROSC and targeted temperature were similar in the early and late awakening group. In the late awakening group 92% of the patients were treated with midazolam at day 0 compared to 57% in the early awakening group (p = 0.021). At day 1 83% of the patients with late awakening were treated with midazolam vs 56% in the early awakening group (p = 0.063). The cumulative dosage of midazolam did not differ between both groups (169 mg/24 hr in the early awakening group and 188 mg/24 hr in the late awakening group P = 0.613). Renal function was similar in both groups, RIFLE 0 in 81.8% in the early awakening group vs 67.7% in the late awakening group (P = 0.269) The patients in the early awakening group were significantly more often treated with propofol 74.5% vs 41.7% the late awakening group (P = 0.017) The dosage was significantly higher in the early awakening group. (2466 mg/24 hr in the early awakening group vs 643 mg/24 hr late awakening group p = 0.05)

**Conclusions:** In conclusion the use of midazolam, independent of the dosage or renal function, is a risk factor for prolonged awakening after cardiac arrest. Thereby midazolam could be a major confounder in the prognostication of neurologic outcome. The current protocol advises the use of opioids and hypnotics. We suggest that, if possible, patients after cardiac arrest are treated with short-acting sedation only to prevent inaccurate neurologic prognostication due to sedation effects.

## P184 The influences of ketamine or morphine on hemodynamics, acid-base status and early survival in rats after asphyxia cardiac arrest: a pilot study

### A Konkayev^1^, V Kuklin^2^, T Kondratyev^3^, M Konkayeva^1^, N Akhatov^1^, M Sovershaev^4^, T Tveita^3^, V Dahl^2^

#### ^1^Astana Medical University, Astana, Kazakhstan; ^2^Akershus University Hospital, Oslo, Norway; ^3^The Arctic University of Norway, Tromsø, Norway; ^4^University Hospital of Northern Norway, Tromsø, Norway

**Introduction:** Acute hypoxia results in uncontrolled release of glutamate and the consequent stimulation of NMDA receptors, which affects the whole ionic homeostasis and finally activates apoptosis of neurons [1]. A potential therapeutic approach to prevent this sequence of events is a blockade of NMDA receptors. Meanwhile, in different models of acute hypoxia, activation of delta-opioid receptors by morphine demonstrates cardioprotective effect with a consequent increase in animal survival [2]. Thus, we aimed to test the effects of morphine or ketamine on hemodynamics, acid-base status and early survival in rats after asphyxia cardiac arrest (ACA)

**Methods:** After instrumentation under anaesthesia with Thiopental sodium (60 mg/kg, i.p.), Wistar rats (n = 21) weighing between 350–400 g were randomly assigned to three groups where: 1. Morhpine 5 mg/kg iv (n = 7) was given 10 min before ACA; 2. Ketamine 40 mg/kg iv (n = 7) was given 10 min before ACA; 3. Control (n = 7), the same amount of NaCl 0,9% iv was given 10 min before ACA. The rats were asphyxiated by clamping the tracheostomia tube at the end of expiration for 5 min. Resuscitation included epinephrine (0.02 mg/kg, iv), manual thoracic compressions (180 per min) and mechanical ventilation (21% O2, 80 breaths/min). Invasive MAP was recorded at the baseline (BL), every 1 min during ACA and every 5 min in post-resuscitation (PR) period. Blood gas samples were taken at the BL and 10 min at the PR period. Early survival was determined at the 20 min after ACA.

**Results:** No differences in MAP between the rats was found at the BL period. The rats pre-treated by ketamine got significantly higher MAP during PR period (133.9 ± 30.4 vs 52.7 ± 37.3 and 60.0 ± 26.2 mm Hg, respectively, p < 0.002) and had significantly lower production of lactate (11.8 ± 2.2 vs 13.5 ± 1.5 and 15.6 ± 1.2 mmol/l, respectively, p < 0.002) when compared to the rats treated by morhpine and only saline. Six of the seven rats survived at the 20 min after ACA in the ketamine group while four of the seven and two of the seven rats survived in the morhpine and Control groups respectively (P = 0.122).

**Conclusions:** Pre-treatment with ketamine attenuated significantly disturbances in hemodynamics and lactate after ACA, but it did not improve early survival when compared to the rats pre-treated by morphine or saline.

**References**

1. Choi DW. Neuron 1988;1:623–34

2. Endoh H, et al. Crit Care Med 2001;29:623–7

## P185

### Withdrawn

## P186 Ubiquitin c-terminal hydrolase l1 as a predictor of neurological outcome after cardiac arrest and resuscitation

### L Wihersaari^1^, MB Skrifvars^2^, S Bendel^3^, KM Kaukonen^4^, J Vaahersalo^4^, J Romppanen^5^, V Pettilä^4^, M Reinikainen^1^

#### ^1^North Karelia Central Hospital, Joensuu, Finland; ^2^Australian and New Zealand Intensive Care Research Centre, School of Public Health and Preventive Medicine, Monash University, Melbourne, Australia; ^3^Kuopio University Hospital, Kuopio, Finland; ^4^Helsinki University and Helsinki University Hospital, Helsinki, Finland; ^5^Eastern Finland Laboratory Centre, Kuopio, Finland

**Introduction:** Ubiquitin C-terminal hydrolase L1 (UCHL1) is an enzyme present in central nervous system neurons. We aimed to assess UCHL1 as a predictor of neurological outcome after cardiac arrest in comparison with neuron-specific enolase (NSE) in this FINNRESUSCI substudy [1].

**Methods:** We prospectively collected data on 249 patients who were admitted to 21 intensive care units after out-of-hospital cardiac arrest (OHCA) between March 1, 2010 and February 28, 2011. Of these patients, 177 (71%) had a shockable initial rhythm. We measured serum concentrations of UCHL1 and NSE at 24 h and 48 h after cardiac arrest. UCHL1 concentrations were analysed by a commercial ELISA kit and NSE concentrations were measured by electrochemiluminescence immunoassay. We evaluated the ability of these biomarkers to predict poor outcome (defined as Cerebral Performance Category 3-5, indicating death or severe neurologic deficits) at 12 months after cardiac arrest using the area under the receiver operating characteristic curve (AUROC).

**Results:** Overall, 121 patients (49%) had a poor outcome at 12 months. For both UCHL1 and NSE, the concentrations were higher for patients with poor outcome than for those with good outcome (Table 3). The NSE concentration at 48 h was the best predictor of poor outcome (AUROC 0.72).

The median time from cardiac arrest to return of spontaneous circulation (ROSC) was 20 min. The prognostic performance of NSE at 48 h was particularly good for patients with the time to ROSC longer than 20 min, with AUROC 0.80 (95% CI, 0.71-0.89). For patients with ROSC under 20 min, the AUROC for NSE at 48 h was only 0.53 (0.41-0.66), but the AUROC for UCHL1 at 24 h was 0.70 (0.59-0.81).

**Conclusions:** Post-cardiac arrest UCHL1 concentrations are higher in patients with poor outcome than in those with good outcome, but the ability of UCHL1 to predict long-term outcome is weaker than that of NSE in the overall population of ICU-treated OHCA patients. However, for patients with ROSC under 20 min, the prognostic performance of UCHL1 at 24 h was satisfactory.

**Reference**

**1.** Vaahersalo J et al. Therapeutic hypothermia after out-of-hospital cardiac arrest in Finnish intensive care units: the FINNRESUSCI study. Intensive Care Med 39: 826–37, 2013.

**Table 3 (abtsract P186).** Concentrations (ng/ml) presented as means ± standard deviations.

	Poor outcome	Good outcome	p	AUROC (95% CI)
UCHL1 at 24 h	19.8 ± 27.3	10.8 ± 9.5	0.001	0.66 (0.60-0.73
UCHL1 at 48 h	22.6 ± 20.7	15.6 ± 15.9	0.006	0.66 (0.59-0.74)
NSE at 24 h	31.1 ± 65.2	11.0 ± 10.6	0.001	0.65 (0.58-0.72)
NSE at 48 h	42.8 ± 58.8	10.5 ± 8.3	<0.001	0.72 (0.65-0.80)

## P187 Time to awakening after cardiac arrest and target temperature management

### A Lybeck^1^, T Cronberg^1^, N Nielsen^2^, H Friberg^1^

#### ^1^Skane University Hospital, Lund University, Lund, Sweden; ^2^Helsingborg Hospital, Lund University, Helsingborg, Sweden

**Introduction:** In this post hoc analysis of the target Target Temperature Management-trail (TTM-trail) [1], we investigate the time until awakening and its relationship to target temperature and neurological outcome. Sedation is also compared.

**Methods:** The TTM-trial randomized 950 patients to a target temperature of 33 °C (TTM33) or 36 °C (TTM36) in 36 hospitals in 12 countries, with no difference in survival or neurological outcome between groups. Awakening was defined as Glasgow Coma Scale motor score (GCS-M) 6 in the ICU. Neurological outcome was assessed using the cerebral performance category scale (CPC) at 180 days. Cumulative doses of sedative drugs (propofol, midazolam, fentanyl, morphine, remifentanil) were retrospectively collected at 12, 24 and 48 hours. There was a strict protocol for prognostication and withdrawal of care.

**Results:** 496 patients had registered awakening in the ICU. Day of awakening occurred later in TTM33 (median 4, IQR 3-6) vs TTM36 (median 4, IQR 3-5), p < 0.0021 (Mann-Whitney-U). The latest recorded awakening was at day 22. We found a correlation between day of awakening and neurological outcome (Spearmans correlation coefficient 0.20, p < 0.0001), but there was no difference in neurological outcome between treatment groups (p = 0.21, Chi-squared). Doses of sedative drugs were available for 352 patients from 20 trial sites. We found no difference in doses of sedative drugs at 12, 24 or 48 hours between TTM33 and TTM36.

**Conclusions:** Time to awakening was longer in TTM33 than in TTM36. Day of awakening correlated with neurological outcome. In patients who awoke, there was no difference in neurological outcome between treatment groups.

**Reference**

1. Nielsen, N et al. N Engl J Med 369(23): 2197–206, 2013.

## P188 Target temperature management in comatose survivors of cardiac arrest - comparison of endovascular, esophageal and surface cooling

### M Rauber, K Steblovnik, A Jazbec, M Noc

#### University Medical Centre Ljubljana, Ljubljana, Slovenia

**Introduction:** Target temperature management represents important part of post-resuscitation care in comatose survivors of out of hospital cardiac arrest (OHCA). Early induction of hypothermia, tight maintenance and prevention of hyperthermia during rewarming appear to be essential to maximize neuroprotection. The aim of our study was to compare endovascular, esophageal and surface cooling.

**Methods:** Endovascular cooling (ENC) using InnerCool Accutrol Catheter (Philips Healthcare, San Diego, CA, USA), esophageal cooling (ESC) using dedicated device (Advanced Cooling Therapy, Chicago, IL, USA) with concomitant 0.9% saline (0-4 °C, 30 ml/kg in 30 min) and surface cooling (SFC) with ice packs and concomitant cold saline were compared. Target temperature was 32-34 °C. After 24 hours of maintenance, gradual rewarming targeted at 0.1-0.2 °C/h was performed. Core body temperature was measured continuously by thermistor placed intravesically.

**Fig. 7 (abstract P188).**
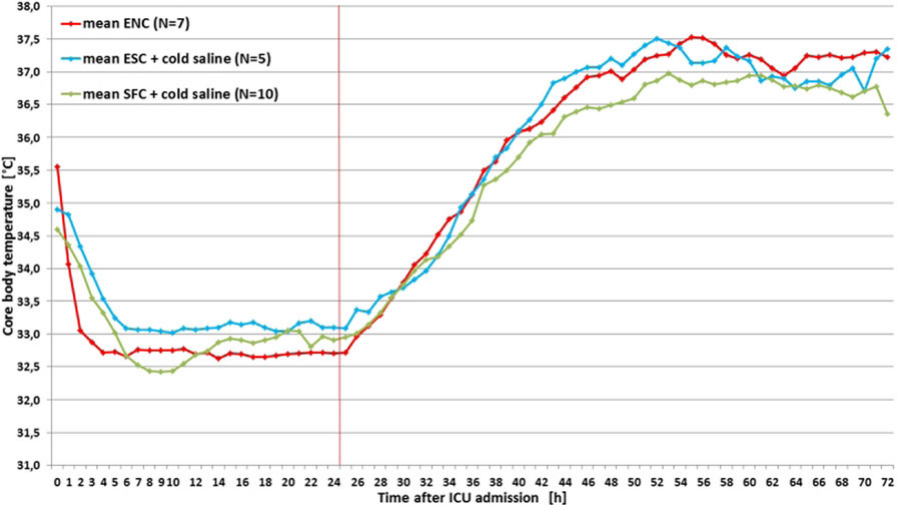
Mean patients’ temperatures after ICU admission for each cooling method.

**Results:** A total of 22 OHCA patients were included. ENC was used in 7 patients, ESC in 5 patients and SFC in 10 patients (Fig. 7). By extrapolating temperature curves, mean time from initiation of cooling to 34 °C was 1.1 h in ENC group, 2.8 h in ESC group and 2.1 h in SC group (p = 0.08). Variation of temperature during the maintenance phase expressed as mean hourly standard deviation was 0.26 °C in ENC, 0.42 °C in ESC and 0.76 °C in SFC groups, respectively (p < 0.001). The percentage of patients with post-rewarming hyperthermia (>38 °C) at any measurement was 43% in ENC group, 20% in ESC group and 50% in SFC group (p = 0.3). The post-rewarming hyperthermia in ENC and ESC groups was linked with device removal prior to 72 hours post OHCA.

**Conclusions:** Our non-randomized comparison indicates that ENC provides the fastest induction of hypothermia and best temperature maintenance. Significant proportion of patients still experience temporary hyperthermia during rewarming regardless of the cooling method.

## P189 Influence of an esophageal cooling and warming device on patient temperature in the operating room

### P Kalasbail^1^, F Garrett^2^, E Kulstad^3^

#### ^1^Cleveland Clinic, Cleveland, OH, United States; ^2^Garrett Technologies, Northbrook, IL, United States; ^3^Advocate Christ Medical Center, Oak Lawn, IL, United States

**Introduction:** Temperature management is important for a number of clinical conditions, and a variety of methods exist to effect changes in body temperature. In general, these methods are divided into external (surface) or internal/core (intravascular) approaches. A new device (the ECD, or Esophageal Cooling/Warming Device) utilizes a closed circulation of cooled or warmed water via the esophageal route to achieve patient heat transfer, offering core temperature control without the need to access the intravascular space. We sought to measure the influence of this device on the temperature of patients undergoing surgical procedures in the operating room.

**Methods:** This was a secondary analysis of data from a prospective interventional study of patients undergoing elective non-cardiac surgery at the Cleveland Clinic, USA. The primary outcome was quantification of heat transferred to or from patients. Under this IRB approved study, after written informed consent, patients were subjected to two distinct 30-minute periods of either warming or cooling (with the order randomized), during which inlet temperature, outlet temperature, and flow rate through the ECD was measured, providing the data necessary for heat balance calculations. In this secondary analysis we examined patient temperatures recorded during the study, and determined mean temperature change over each 30 minute interval. Temperatures were recorded using zero-flux cutaneous thermometry (3 M SpotOn).

**Results:** Nineteen patients were enrolled in this study from April to November, 2016. During the warming cycle, mean patient starting temperature was 35.5 C +/- 0.52 C, increasing to 35.8 +/- 0.62 C over 30 minutes. The mean warming rate was 0.56 +/- 0.64 C/hr. During the cooling cycle, mean patient starting temperature was 35.7 C +/- 0.61 C, decreasing to 35.3 +/- 0.61 C over 30 minutes. The mean cooling rate was 0.88 +/- 0.87 C/hr. The finding that cooling was faster than warming may be attributed to the greater difference in core-to-perfusion temperature (water temperature of 7 C cooling, 42 C warming); however, extraneous factors such as patient exposure, body habitus, external warming, and room temperature, may also have influenced performance.

**Conclusions:** Although patients differed slightly in their rates of temperature change, these data suggest that patient temperature modulation utilizing the esophageal route is effective, and that clinically meaningful core patient temperature change can be attained in both cooling and warming, without the need to access the intravascular space.

**Reference**

Markota A, Fluher J, Balazic P, Kit B, Sinkovic A: Therapeutic hypothermia with esophageal heat transfer device. Resuscitation 2015, 96:138.

## P190 Outcomes and interventions among out-of-hospital cardiac arrest patients transported to hospital with ongoing cardiopulmonary resuscitation

### DJ Bergström, HR Olsson, S Schmidbauer, H Friberg

#### Skåne University Hospital, Lund, Lund, Sweden

**Introduction:** The introduction of automatic chest compression (ACC) devices has made it possible to transport patients with ongoing cardiopulmonary resuscitation (CPR) following out-of-hospital cardiac arrest (OHCA). In the region of Skåne in Sweden, all ambulances are equipped with an ACC device and local guidelines encourage prompt transportation of a majority of patients to hospital, regardless of whether field return of spontaneous circulation (ROSC) is achieved. In this retrospective registry study, we investigated the outcomes for patients transported with ongoing CPR between 2010-2015. We also studied the frequency of hospital-bound interventions against suspected reversible causes of cardiac arrest.

**Methods:** A local register containing Utstein-style data from all OHCA patients transported to SUS Lund was used and additional data were retrieved from medical records. If and where patients achieved ROSC was recorded. Interventions against suspected reversible causes of cardiac arrest were also noted and categorised per the “4Hs & 4Ts”, outlined in resuscitation guidelines. Patients with in-ambulance arrest were excluded.

**Results:** During the study period, 639 patients were transported to hospital following OHCA. A total of 160 patients achieved sustained ROSC before admission to hospital, 72 of whom (45%) survived to hospital discharge. Seventy-eight patients were excluded due to in-ambulance arrest (n = 49), arrest at hospital grounds (n = 11) and missing data (n = 18). The remaining 401 patients were transported with ongoing CPR of whom 52 (13%) eventually had sustained ROSC and admitted to the ICU. Eight patients (2%) survived to hospital discharge, 4 did not receive any further in-hospital intervention while 4 received an intervention that could not have been performed in the prehospital setting. These interventions were: angiography with epinephrine injected in the aortic root (n = 1), percutaneous coronary intervention (n = 1), insertion of a trans-venous pacemaker (n = 1), intubation and suction of aspirated fluids (n = 1). Three of these four survivors had intermittent ROSC in the field. The initial rhythm was shockable in 5 of 8 survivors and 3 had pulseless electrical activity.

**Conclusions:** The prognosis for patients transported to hospital with ongoing CPR is poor and the extra treatment potential in-hospital are rarely utilised. No patient with asystole without ROSC in the field survived.

## P191 Hypoxic and hyperoxic preconditioning in organ protection against ischemia-reperfusion injury: the experimental study

### I Mandel^1^, S Mikheev^2^, Y Podoxenov^2^, I Suhodolo^3^, A Podoxenov^2^, J Svirko^2^, A Sementsov^2^, L Maslov^2^, V Shipulin^2^

#### ^1^City Clinical Hospital No 83 of FMBA of Russia, Moscow, Russia; ^2^Cardiology Research Institute, Tomsk, Russia; ^3^Siberian State Medical University, Tomsk, Russia

**Introduction:** Preconditioning with a moderate hypoxia and hyperoxia is an effective drug-free way to mitigate organ dysfunction to ischemia-reperfusion injury [1, 2].

**Methods:** The prospective study included 20 rabbits divided into four groups: hypoxic preconditioning (HypP), n = 5; hyperoxic preconditioning (HyperP), n = 5; hypoxic-hyperoxic preconditioning (HHP), n = 5; and control group, n = 5. Study was approved by local ethics committee. All animals were anesthetized by sevoflurane and mechanically ventilated via nasotracheal tube. In HypP group we exposed rabbits to two series of 10% oxygen for 10 min with 5 min reoxygenation. In HyperP group rabbits were exposed to 80% oxygen for 30 min. In HHP group rabbits were exposed to two series of 10% oxygen for 10 min with 5 min reoxygenation followed by 80% oxygen for 30 min. Then we started CPB and induced acute myocardial infarction by ligation of left coronary artery. After 45 min of ischemia reperfusion was performed for 60 min. We investigated myocardial slices and measured ischemic area (IA) and risk area (RA) [3] and calculated IA/RA ratio; also we conducted light microscopy of gut mucosa, kidneys, liver, spleen and lungs.

**Results:** IA/RA decreased in HypP group by 23%, in HyperP group 26%, in HHP group by 32% in comparison with control group (p = 0.009, Kruskal-Wallis test) [3]. Acid-based status, blood lactate and glucose levels were stable during all types of preconditioning. Single ventricular arrhythmia was observed more often than multiple ventricular arrhythmia in preconditioning animals and the opposite in control group. Incidence of ventricular fibrillation were lower in HHP group (differences did not reach statistical significance, X2 test). Light microscopy of myocardium revealed less damage in HHP group as compared to other groups. Light microscopy of kidneys revealed marked edema of cortical and medullar substances in the control group. Gut mucosa and liver parenchyma had enlarged capillaries, sometimes filled with erythrocytes. Microscopic structure of kidneys, small intestine and liver was less affected in HHP group.

**Conclusions:** Hypoxic-hyperoxic preconditioning provided the highest tolerance of the myocardium and splanchnic organs to the effects of ischemia-reperfusion injury.

**References**

**1.** Petrosillo G. et al. Free Radic Biol Med. - 2011. - Vol 50(3). – p.. 477–483.

2. Xu K. et al. Adv Exp Med Biol. - 2014. - Vol. 812. – p. 309–315.

3. Mandel I. et al. Journal of Cardiothoracic and Vascular Anesthesia. - 2016. - Vol. 30 (Supplement 1), p. S6–S7.

## P192 Diabetes in an animal model worsens neurological outcome following cardiac arrest

### LV Vammen^1^, SR Rahbek^2^, NS Secher^1^, JP Povlsen^3^, NJ Jessen^4^, BL Løfgren^1^, AG Granfeldt^1^

#### ^1^Aarhus University Hospital, Aarhus C, Denmark; ^2^Regional Hospital of Randers, Randers, Denmark; ^3^Regional Hospital of Horsens, Horsens, Denmark; ^4^Aarhus University, Aarhus, Denmark

**Introduction:** Cardiac arrest carries a poor prognosis. The average cardiac arrest patient is comorbid and retrospective studies suggest that diabetes mellitus is an independent risk factor for increased mortality after cardiac arrest. Despite this, cardiac arrest animal studies are conducted on healthy young animals, limiting our knowledge regarding the post-cardiac arrest organ dysfunction and the impact of type 2 diabetes mellitus (T2DM).

We hypothesize that T2DM, in a rat model of cardiac arrest, is associated with increased brain injury and reduced left ventricular function following resuscitation.

**Methods:** We used the Zucker Diabetic Fatty (ZDF) rat as an animal model of T2DM. The ZDF rats (n = 13), non-diabetic Zucker Lean Control (ZLC) rats (n = 15), and healthy Sprague Dawley (SprD) rats (n = 8) underwent asphyxia-induced cardiac arrest and were resuscitated and monitored for 180 min. after return of spontaneous circulation (ROSC). Brain injury was evaluated by neuron specific enolase (NSE) and left ventricular function was measured as fractional shortening (FS) by echocardiography both measured at baseline and 180 min. after ROSC.

**Results:** Total asphyxia time was 639 s (SD 24) in the ZDF group which was significantly longer than 592 s (SD 18, p < 0.0001) in the ZLC group, but no different from the SprD group at 634 s (SD 20, p = 0.6). There were no differences among groups in baseline NSE or FS. 180 min. after ROSC median levels of NSE were significantly increased in the ZDF group, 10.8 ng/mL [25%Q;75%Q: 7.6;11.3], compared with the two control groups: ZLC group 2.0 ng/mL [25%Q;75%Q 1.7;2.3, p = 0.0004] and SprD group 2.8 ng/mL [25%Q;75%Q 2.3;3.4, p = 0.0004]. At the end of experiment mean FS was significantly higher in the ZDF group at 36% (SD 6), compared with the ZLC group, 22% (SD 3, p = 0.0031), and the SprD group, 24% (SD 6, p < 0.0001). At baseline lactate was 1.9 (SD 0.9) in the ZDF group, which was comparable with 1.4 (SD 0.4, p = 0.512) in the ZLC group and 1.2 (SD 0.5, p = 0.052) in the SprD group. At the end of experiment lactate concentration in the ZDF group was at 8.2 mmol/L (SD 3.1) significantly higher than the control groups: ZLC group 2.6 mmol/L (SD 1.7, p = 0.001) SprD group 1.8 mmol/L (SD 0.9, p < 0.0001).

**Conclusions:** Cardiac arrest in an animal model of T2DM results in increased brain injury, while in contrast left ventricular function was increased when compared to non-diabetic animals.

## P193 Increased energy required to reach target temperature in post-cardiac arrest patients is associated with better outcomes

### A Grossestreuer^1^, S Perman^2^, P Patel^1^, S Ganley^1^, J Portmann^1^, M Cocchi^1^, M Donnino^1^

#### ^1^Beth Israel Deaconess Medical Center, Boston, MA, United States; ^2^University of Colorado, Denver, CO, United States

**Introduction:** We hypothesized the amount of energy required by the surface device to reach target temperature (Ttarget) in post-cardiac arrest patients treated with therapeutic hypothermia (TH) may be associated with outcomes by serving as a proxy for patient thermoregulatory ability and may modify the relationship between the time to Ttarget and outcomes. Some studies have shown that TH-treated post-arrest patients who reach Ttarget quickly have worse outcomes than those who cool more slowly. However, the ischemia-reperfusion insult of cardiac arrest may cause temperature derangements that affect the time trajectory of TH independent of external cooling factors.

**Methods:** Adult patients with sustained return of spontaneous circulation treated with TH between 2008-2015 with serial temperature data were included. Time to Ttarget was defined as time from TH initiation to the first time the patient temperature was < =34C. Patients with Ttarget >34C were excluded. The energy required to bring a patient to Ttarget (“energy units”) was calculated as average inverse water temperature x 100 x hours between initiation and Ttarget. Primary outcome was neurologic status (measured by Cerebral Performance Category [CPC] score); secondary outcome was survival, both at hospital discharge. Univariate analyses were performed using Wilcoxon rank-sum tests; multivariate analyses used logistic regression. P < 0.05 was considered statistically significant.

**Results:** Of 205 patients included, those with CPC 3-5 required less energy to reach Ttarget (median 8.1 (IQR: 3.6-21.6) vs median 20.0 (IQR: 9.0, 33.5) energy units, p = 0.001) and reached Ttarget quicker (median 2.3 (IQR: 1.5, 4.0) vs median 3.6 (IQR: 2.0, 5.0) hours, p = 0.01) than patients with CPC 1-2. Patients who did not survive required less energy than survivors (median 8.1 (IQR: 3.6-20.8) vs median 19.0 (IQR: 6.5, 33.5) energy units, p = 0.001) and reached Ttarget quicker (median 2.2 (IQR: 1.5, 3.8) vs median 3.6 (IQR: 2.0, 5.0) hours; p = 0.01). Controlling for average water temperature between initiation and Ttarget, the relationship between outcomes and time to Ttarget was no longer significant. Controlling for location, witnessed arrest, age, initial rhythm, and neuromuscular blockade use, increased energy was associated with better neurologic (aOR: 1.01 (95%CI 1.00-1.03), p = 0.039) and survival (aOR: 1.01 (95%CI 1.00-1.03), p = 0.045) outcomes.

**Conclusions:** Increased energy requirement during TH initiation is associated with better outcomes at hospital discharge and may affect the relationship between time to Ttarget and outcomes.

## P194

### Withdrawn

## P195 Clinical outcomes of patients with different co-morbideties witnessed in cardiac arrests inside the intensive care unit

### Y Nassar, S Fathy, A Gaber, S Mokhtar

#### Cairo University, Giza, Egypt

**Introduction:** We aimed for clinical assessment of cardiopulmonary resuscitation (CPR) procedures of witnessed cardiac arrests inside the intensive care units (ICU) and follow up of patients surviving to discharge.

**Methods:** Data were collected prospectively from patients who were witnessed in cardiac arrest and underwent CPR inside an adult medical ICU of Cairo University in the period from Jan. 2013 to Feb 2015. Resuscitation protocol was done according to the latest recommendation of the European society of cardiology. Clinical data were recorded and surviving patients were clinically followed daily until hospital discharge.

**Results:** The study included 110 patients: 41 females (37%) and 69 males (63%). There were 26(24%) patients under 50 years and 84(76%) patients above 50 years. ROSC occurred in 60(55%) and 22(20%) survived to discharge.

According to underlying illness:

- ROSC increased with CNS comorbidities (p0.05), Shock (p0.008), Low MPM0-III (p 0.015) While ROSC decreased with and Respiratory failure (p 0.01)
- Long term survival increased with Low MPM0-III score (p0.018), Low Sofa score (p 0.01), and Rapidly correctable causes (Hypoxia, Hypovolemia, Hydrogen ion acidosis, Hypokalemia, Hyperkalemia, Hypoglycemia, Hypothermia, Toxins, Cardiac Temponade, Tension pneumothorax, Thrombosis and Trauma) (p0.004), while Long term survival decreased with CNS comorbidities (p0.02), Shock (p,0.01), Respiratory Failure post-arrest (p0.02) and Mechanical ventilation post-arrest (p < 0.001)

According to CPR procedures:

- ROSC increased with AF Rhythm (p0.03),less duration of CPR (p0.03), number of cycles of CPR < 2 (p < 0.001), number of DC shocks <2 (p0.02), EF >50% (p0.01), Low HCO3 pre-arrest (p0.009), low HCO3 during arrest (p0.03) and Noradrenaline post-arrest (p0.003).ROSC decreased with high PaCO2 pre-arrest (p 0.002) and high PaCO2 during arrest (p 0.001).
- Long term survival increased with RBBB (p < 0.001) and frequent PVCs (p < 0.001). Long term survival decreased with Asystole (p 0.01).

**Conclusions:** CPR in the ICU may achieve variable rates of short and long term survival depending on the associated comorbidities

## P196 Outcomes of non-traumatic out-of-hospital cardiac arrests witnessed by layperson

### YC Chia

#### Tan Tock Seng Hospital, Singapore, Singapore

**Introduction:** To look at the outcomes of patients who suffered a non-traumatic OHCA, witnessed by layperson.

**Methods:** This is a retrospective case record review. Inclusion criteria included all patients who suffered a non-traumatic OHCA conveyed by emergency medical services (EMS) to our Emergency Department (ED) from 1st Aug 2012 to 31st Aug 2014. Exclusion criteria included traumatic OHCA and all patients declared dead at scene. EMS data were extracted from National Cardiac Arrest registry. Data of patients admitted were extracted from inpatient electronic case records. Patient discharged from hospital were followed up for 30 days.

**Results:** There were 888 OHCA. 529 were witnessed. Among witnessed, 279 by family, 134 by layperson, 76 by EMS, and 40 by healthcare providers.

134 OHCA witnessed by layperson, 47 occurred in night-time between 2000-0800 hrs. 87 occurred in daytime between 0800-2000 hrs. 4 happened in healthcare facility, 40 in a residential area, 8 in industrial area, 13 in places of recreation, 43 in commercial / public area, 20 in the streets or highway and 6 in brothels.

57 received bystander CPR while 77 did not. Among 57 who received bystander CPR, 9 also had community AED applied. The rhythms on the AED were 4 unknown, 4 asystole and 1 PEA. None was shockable. The initial rhythms by EMS on those 57 patients who received bystander CPR were 24 asystole, 8 PEA, 10 unknown and 15 VF. 3 of the patients with VF were subsequently discharged from hospital alive with CPC 1.

None of the 77 patients who had a witnessed OHCA by a layperson and did not received bystander CPR survived.

134 OHCA witnessed by layperson, 21 were admitted to hospital. 4 were discharged alive with cerebral performance category (CPC) 1. Among these 4 patients with good outcome, 3 had VF as initial presenting rhythm, while 1 had PEA. All happened in public area in daytime, all had bystander CPR, all did not have community AED applied but all had field ROSC

**Conclusions:** Our results showed patients with OHCA witnessed by layperson tend to occur in public areas during daytime and most did not received bystander CPR or application of community AED. Some of these arrests were witnessed by laypersons, however, none survived when CPR was not started. This warrants further study to find out the reasons for not initiating CPR. There should be more efforts directed at community CPR programs. Technology can help to direct rescuers to OHCA victim so that early CPR can be initiated.

## P197 Reducing the risk of a poor outcome: considering the ‘other’ victim of out-of-hospital cardiac arrest (OOHCA) following unsuccessful resuscitation

### R Lewis-Cuthbertson^1^, K Mustafa^2^, A Sabra^2^, A Evans^2^, P Bennett^1^

#### ^1^Swansea University, Swansea, United Kingdom; ^2^Abertawe Bro Morgannwg Health Board, Swansea, United Kingdom

**Introduction:** Early intervention and prevention of psychological disorders could help to significantly reduce costs to the NHS. Relatives who experience the sudden and unexpected death of a relative following OOHCA may be at high risk of developing both psychological and physical health problems. As many of these deaths occur within the Emergency Department(ED), it is important to understand the support needs of relatives to help minimise risk of a poor outcome.

**Methods:** Next of kin of deceased non-traumatic, non-paediatric OOHCA patients were invited to take part in face-to-face interviews 3 months’ after death. Twelve male and female participants who experienced the death of a relative following an OOHCA and who had either i)witnessed the event and provided CPR, ii)witnessed the event and did not provide CPR, and iii)those who did not witness the event completed audio-recorded interviews lasting up to 90 minutes. Audio-recordings were transcribed verbatim and subjected to inductive thematic analysis.

**Results:** Three major themes were identified. 1)Negative psychological and physical health outcomes: Post-traumatic stress symptoms were reported including vivid and intrusive re-experiencing of the event. Flashbacks, recurring traumatic images, and experiencing physical symptoms associated with the heart were common. Intimations of mortality were associated with hyperarousal symptoms and health anxieties. Coping techniques included avoidance behaviours and emotional numbing, often masking their distress and support needs. 2)Am I to Blame: Self-critical thinking regarding one’s own actions in relation to this event were evident. Relatives blamed themselves for not noticing sooner that something was wrong, particularly when an underlying heart condition was identified as cause of death. Many were pre-occupied with the thought: ‘Could I have done more?’. 3)Information and Support Needs: Many felt uncertain and uninformed about what was happening creating feelings of anger, frustration and confusion. Seeking information was important for relatives to help both try to make sense of what happened and exonerate feelings of guilt and self-blame.

**Conclusions:** Findings suggest that the psychological impact of experiencing the sudden death of a relative following an OOHCA may be profound. Information provision is crucial to help relatives make sense of their experience and exonerate feelings of guilt and self-blame. Support of relatives needs to be a more serious consideration to help minimise risk of poor psychological outcome and reduce the health economic burden this may pose.

## P198

### Withdrawn

## P199 he use of the bispectral index and suppression ratio to predict poor neurological outcome in post-cardiac arrest patients treated with targeted temperature management at 33°c

### W Eertmans, C Genbrugge, W Boer, J Dens, C De Deyne, F Jans

#### Ziekenhuis Oost-Limburg, Genk, Belgium

**Introduction:** Bispectral index (BIS) monitoring has been considered as a promising electrophysiological tool for early prognostication after out-of-hospital cardiac arrest (OHCA). In recent years, a broad range of BIS thresholds has been put forward at diverse time points to predict neurological outcome in OHCA patients. This study aimed to reach consensus about the optimal time point and threshold for predicting poor neurological outcome after OHCA using the BIS monitor.

**Methods:** A prospective, observational study was performed during TTM at 33 °C in 77 successfully resuscitated OHCA patients. After admission to the ICU, BIS and Suppression Ratio (SR) monitoring was started using the BIS VISTA™ (Aspect Medical Systems, Inc. Norwood, USA). BIS and SR values were continuously recorded during the hypothermic and rewarming phase. During this time period, mean BIS and SR values per hour were calculated and used for analysis. The Cerebral Performance Category (CPC) scale was used to define patient’s outcome at 180 days after OHCA (CPC 1-2: good - CPC 3-5: poor neurologic outcome). Receiver operator characteristics curves were constructed to determine the best cut-off value and time point to predict poor neurological outcome.

**Results:** At 180 days post-cardiac arrest, 38 patients (49%) had a good neurological outcome (CPC 1-2), while 39 patients (51%) had a poor outcome (CPC 5). There were no patients with a CPC 3 or 4. Patients with a good neurological outcome had higher BIS and lower SR values than non-survivors. Using a mean BIS value below 25.5 at hour 12 as threshold criteria, poor neurological outcome was predicted with a sensitivity of 49% (95% CI 30-65%) and specificity of 97% (95% CI 85-100%) (AUC: 0.722 (0.570-0.875); p = 0.006). With a cut-off value of SR above 2.5, the optimal sensitivity (74%, 95% CI 56-87%) and specificity (92%, 95% CI 78-98%) for poor neurological outcome was obtained at hour 23 (AUC: 0.836 (0.717-0.955); p < 0.001).

**Conclusions:** This prospective, observational study confirmed that mean BIS values at hour 12 can be used to predict poor neurological outcome. In addition, we showed that the predictive ability of the SR might be even higher as compared to the one of BIS. Overall, our results suggest that BIS and SR monitoring can be used to assist with early neuroprognostication after OHCA.

## P200 Retrospective analysis of trends in outcome following Out of Hospital Cardiac Arrest from a UK regional cardiac arrest centre, with a focus on haematological parameters

### A Skorko, M Thomas

#### Bristol Royal Infirmary, Bristol, United Kingdom

**Introduction:** The Bristol Royal Infirmary is a tertiary out of hospital cardiac arrest (OHCA) centre, serving a population of 1,000,000 in the South West of England.

Debate is ongoing regarding the optimal antiplatelet strategy for survivors of OHCA, given the concerns of both bleeding and clotting complications in this population. Studies vary in the rates of bleeding seen in this population, from 0 to 56% [1].

To understand why OHCA survivors are at risk of bleeding and whether this impacts survival to hospital discharge, we retrospectively analysed laboratory parameters of coagulation(PT and APTT), platelet count, and haemoglobin then stratified these by survival to hospital discharge, over the period January 2009 to August 2016.

**Methods:** Routinely collected electronic data was used to identify patients for this study. Data was extracted for those coded as ‘Anoxic or ischaemic coma or encephalopathy’ with ‘Acute myocardial infarction’ or ‘Ventricular tachycardia or fibrillation´.

Parameters of; status at hospital discharge, platelet count, APTT and haemoglobin within 24 hours of admission were extracted from the database.

**Results:** Comparing the relative risks; An INR of >2 gave a risk ratio of death of 1.29 (95% CI 0.828- 2.01, p =0.257). A platelet count of <50 gave a risk ratio of death of 1.30 (95% CI 0.866- 1.97, p =0.301).

**Conclusions:** Our data demonstrates that platelet count, hemoglobin, PT and APTT do not differ between survivors and non-survivors of OHCA.

However, absolute values do not give an indication as to the function of platelets or of the coagulation cascade. An analysis of platelet function and the coagulation cascade as a whole may provide better insights into the risks of bleeding in this population. We therefore propose to carry out a prospective analysis of thromoelastometric coagulation assessment and platelet inhibition, and correlate this with bleeding events and administration of antiplatelet therapy

**References**

1. Nolan JP et al. Increasing survival after admission to UK critical care units following cardiopulmonary resuscitation. Critical Care. 2016;20.

**Table 4 (abstract P200).** median and interquartile ranges (IQR) for haematological parameters

	Survivors	non survivors	Mann-Whitney u test
Median Haemoglobin (IQR)	12.90 (11.8 – 14.1)	12.90 (11.8 – 14.1) 12.60 (10.9-13.9)	P = 0.03
Median platelets (IQR)	187.00 (142.75 – 226)	181.00 (137 – 234)	P = 0.861
Median APTT (IQR)	35.40 (26.52 - 80.6)	32.70 (27.75-68.1)	P = 0.689
Median PT (IQR)	11.50 (10.8 -13.5)	12.10 (11-16.9)	P = 0.01

**Fig. 8 (abstract P200).**
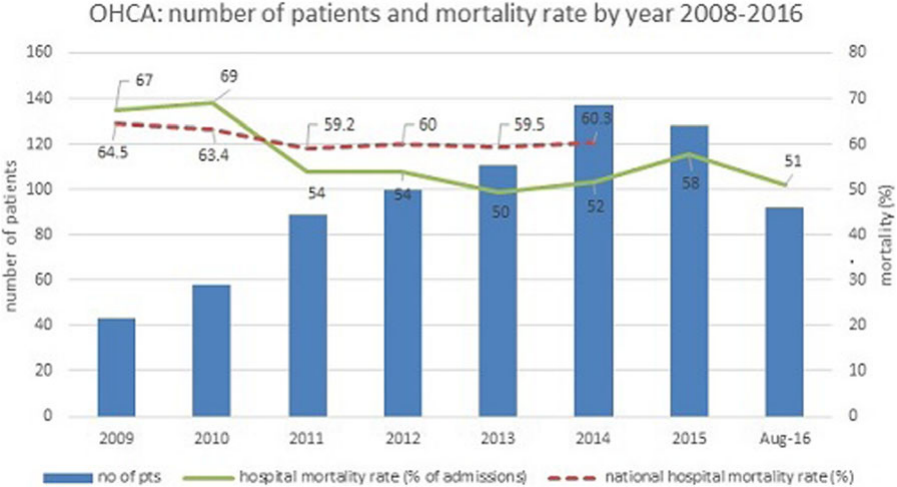
Number of patients treated by year and % survival to hospital discharge

## P201 Organ donation after brain death in refractory cardiac arrest treated with extracorporeal CPR

### M Casadio^1^, A Coppo^2^, A Vargiolu^2^, J Villa^1^, M Rota^1^, L Avalli^2^, G Citerio^1^

#### ^1^University of Milano-Bicocca, Monza, Italy; ^2^San Gerardo Hospital, Monza, Italy

**Introduction:** Cardiac arrest (CA) is a catastrophic event with a high rate of mortality, often resulting in devastating brain injury that might evolve to brain death (BD)[1]. Organ donation from BD after CA patients with ECMO support is still a poorly explored field[2].

**Methods:** We retrospectively enrolled all patients admitted to our hospital between January 2011 and September 2016 after refractory CA treated with eCPR.

**Fig. 9 (abstract P201).**
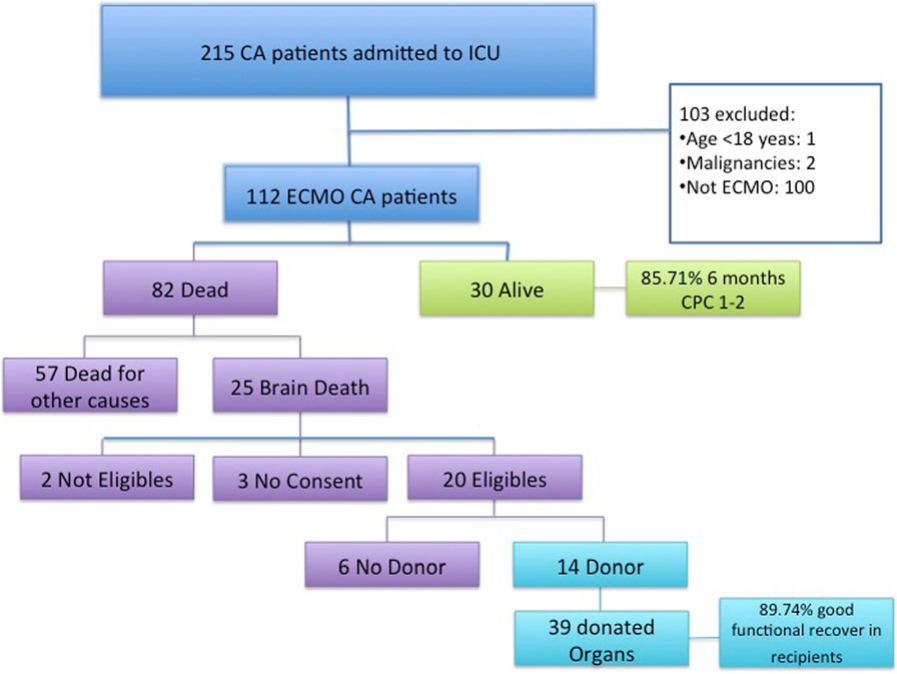
See text for description

**Results:** In the study period 112/215 CA patients received eCPR (52.09%). 30 eCPR-subjects (26.78%) survived at 6 months (85.71% with good cerebral performance, CPC 1-2). 82 died in ICU (25 BD, 22.32% and 57 for other causes, 50.89%) [Fig. 9]. Deads vs. alives differed in age (p = 0.02), comorbidities (p = 0.001), CA (intra or extra-hospital p = 0.03), low flow time (p < 0.0001), mean arterial pressure (p = 0.004), glycemia (p = 0.04) anemia (p = 0.02), renal function (creatinine p < 0.0001, urea p = 0.0005), and early neurological evaluation (CT scan p < 0.001, EEG recording p < 0.001, brainstem reflexes p < 0.001, presence of somatosensory potentials p = 0.03 and GCS p < 0.0001). BD and dead from other causes patients differed in early neurological evaluation (CT scan p < 0.0001, EEG p = 0.004, brainstem reflexes p = 0.02), thrombocytopenia (p = 0.008), coagulation derangement (p = 0.01), inotropic support (p = 0.03). Tab1 shows characteristics of eligible patients at the time of donation. Rate of donation in BD patients was 56% (refusal based on organ biopsy or evaluation in the operation room) with 39 donated organs (23 kidneys, 12 livers, 4 lungs, 89.74% with good functional recovery).

**Conclusions:** eCPR patients might become BD and be considered potential resource for organ donation with a similar success rate as organs retrieved from patients deceased from other causes.

**References**

**1.** Sandroni C et al Int Care Med 42(11):1661–1671,2016

2. Citerio G et al Int Care Med 42(3):305–15,2016

**Table 5 (abtsract P201).** See text for description

		HAEMODYNAMICS AND BIOCHEMICAL DATA		CBC AND COAGULATION	
Day of BD diagnosis	4.68 ± 3.5	MAP (mmHg)	60.7 ± 13.8	WBC (x109/L)	11.7 ± 6.9
ARTERIAL BLOOD GASES		NE max dose (mcg/kg/min)	0.2 ± 0.2	Hb(g/dl)	10.9 ± 1.9
pH	7.35 ± 0.1	DBT max dose (mcg/kg/min)	5.3 ± 3.09	Hct	26.43 ± 4.06
P/F	173.8 ± 225.9	Creatinine (mg/dL)	1.8 ± 0.7	PTLs (x103/L)	91.5 ± 70.6
pCO2(mmHg)	47.3 ± 10.7	Urea (mg/dl)	65.9 ± 34.2	INR	1.3 ± 0.4
Lactate(mmol/L)	3.5 ± 4.5	Bilirubin(mg/d)	0.8 ± 0.8	aPTT ratio	1.4 ± 0.2

## P202

### Withdrawn

## P203

### Withdrawn

## P204 Effects of long-term post-ischemic treadmill exercise on gliosis in the aged gerbil hippocampus induced by transient cerebral ischemia

### JB Moon, JH Cho, CW Park, TG Ohk, MC Shin, MH Won

#### Kangwon National University, Chuncheonsi, South Korea

**Introduction:** Therapeutic exercise is an integral component of the rehabilitation of patients with stroke. The objective of the present study was to investigate effects of post-ischemic exercise on neuronal damage or death and gliosis in the aged gerbil hippocampus after transient cerebral ischemia using immunohistochemistry.

**Methods:** Aged gerbils (male, 22 to 24 months) induced by ischemia were subjected to treadmill exercise for 1 or 4 weeks. Neuronal death was apparently found in the stratum pyramidale of the hippocampal CA1 region and in the polymorphic layer (PoL) of the dentate gyrus (DG) using cresyl violet and Fluoro-Jade B histofluorescence staining.

**Results:** In addition, no significant difference in neuronal death was found after 1 or 4 weeks of post-ischemic treadmill exercise. However, post-ischemic treadmill exercise apparently affected gliosis (activation of astrocytes and microglia). GFAP immunoreactive astrocyte and Iba-1 immunoreactive microglia were activated in the CA1 and PoL of the DG of the group without treadmill exercise. However, 4 weeks after treadmill exercise significantly alleviated ischemia-induced astrocyte and microglia activation, although the gliosis was not alleviated in the animals with 1-week exercise.

**Conclusions:** These findings suggest that long-term post-ischemic treadmill exercise after transient cerebral ischemia could not influence neuronal protection, however, it could effectively alleviate astrocyte and microglial activation in the aged hippocampus induced by 5 min of transient cerebral ischemia.

## P205 Predictors of need for critical care support after stroke thrombolysis in an intensive care unit

### P Papamichalis^1^, V Zisopoulou^1^, E Dardiotis^2^, S Karagiannis^1^, D Papadopoulos^1^, T Zafeiridis^1^, D Babalis^1^, A Skoura^1^, I Staikos^1^, A Komnos^1^

#### ^1^General Hospital of Larissa, Larissa, Greece; ^2^University Hospital of Larissa, Larissa, Greece

**Introduction:** One of the most promising interventions for acute ischemic strokes is intravenous thrombolysis (IVT). In our hospital it is performed in the Intensive Care Unit (ICU), with a 10 year experience for the intervention and participation at international studies [Safe Implementation of Thrombolysis in Stroke – MOnitoring STudy (SITS-MOST)] [1] and registries [Safe Implementation of Treatments in Stroke - International Stroke Thrombolysis Register (SITS-ISTR)]. The aim of the study was to evaluate which factors can predict the need for critical care support after thrombolysis.

**Methods:** Retrospective study including 124 patients with acute ischemic stroke, with mean age 65 years and National Institutes of Health Stroke Scale (NIHSS) at admission 11/range 2 – 28. They all fulfilled the international inclusion criteria [2] and received IVT with alteplase. Demographic data and severity scores [Simplified Acute Physiology Score (SAPS) II and NIHSS] were recorded. Patients were divided to those who demanded advanced life support and neurocritical care interventions after IVT (n = 14) and those who did not (n = 110). Comparison amongst the two groups was performed with application of proper statistical tests.

**Results:** The need for critical care support was significantly greater for patients with higher SAPSII, higher NIHSS after 2 hours, at 24 hours and at 7 days (Mann-Whitney U test for the above mentioned comparisons) and patients with history of vascular disease (Fisher’s exact test) (p < 0.05 for all comparisons). Off-Label Thrombolysis, NIHSS <5, age >80 years, sex, age, NIHSS at admission, aggregate thrombolysis time, history of: diabetes mellitus, arrhythmia, hypertension, smoking, hyperlipidemia, former ischemic stroke did not significantly correlate with critical care need.

**Conclusions:** In accordance with previous studies [3,4] higher severity scores (SAPSII, NIHSS) and presence of vascular disease can serve as markers for prediction of need for critical care support after IVT. Our report is the first one in the international literature of SAPSII correlating with thrombolysis results.

**References**

1) Wahlgren N et al, Lancet 369:275–282, 2007

2) The European Stroke Organisation (ESO) Executive Committee and the ESO Writing Committee, Cerebrovasc Dis 25:457–507, 2008

3) Faigle R et al, PLoS One 9:e88652, 2014

4) Mazya M et al, Stroke 43:1524–1531, 2012

## P206 A life threatening emergency: PRES - cases series and literature review

### S Silva Passos, F Maeda, L Silva Souza, A Amato Filho, T Araújo Guerra Granjeia, M Schweller, D Franci, M De Carvalho Filho, T Martins Santos, P De Azevedo

#### University of Campinas, Campinas, Brazil

**Introduction:** To describe comorbidities, clinical presentation, diagnostic method, treatment and outcome of 6 patients with Posterior Reversible Encephalopathy Syndrome (PRES). PRES is consequence of a reversible subcortical brain edema in patients with acute neurological symptoms, such as headache, impaired sensorium, visual abnormalities, nausea/vomiting, cerebellar syndrome, focal neurological deficits and seizures. Causes include hypertension, eclampsia/pre-eclampsia, sepsis, autoimmune disease, immunosuppressive agents, chemotherapy and renal failure. Radiological findings on computed tomography (CT) and magnetic resonance imaging (MRI) include abnormalities of white and grey matter, predominantly affecting parietal and occipital lobes. The treatment is based on the removal of the underlying cause.

**Methods:** Retrospective and descriptive study of 6 medical records of patients hospitalized with diagnosis of PRES at the tertiary hospital of the University of Campinas, São Paulo, between 2015 and 2016.

**Fig. 10 (abstract P206).**
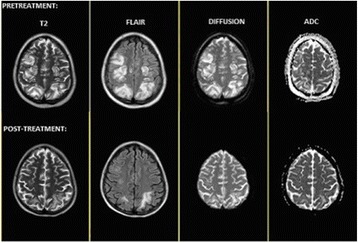
Multiple hyperintense areas with mass effect in the high convexity

**Results:** Four patients had lupus, 2 were solid-organ transplant patients (1 kidney and 1 liver). Immunosuppression and headache were found in all patients, 5 presented hypertensive emergencies, 4 had seizures, 3 showed decreased level of consciousness and nausea and vomiting, 1 had status epilepticus. The diagnosis was made clinically and with CT in 4 cases and MRI in 2 cases (Fig. 10). Treatment was performed with intravenous blood pressure lowering agents and antiepileptic drugs. The length of stay ranged from 22 days to 66 days. Five patients showed full recovery, and 1 died of intracranial hemorrhage.

**Conclusions:** Autoimmune disease, use of immunosuppressant and hypertension are important risk factors for PRES. Patients usually have a good recovery after prolonged stay in hospital, but death and neurological disability may occur. Therefore, early recognition and appropriate treatment may change patient´s outcome.

## P207 A case report: chronic lymphocytic inflammation with pontine perivascular enhancement responsive to steroids (CLIPPERS)

### R Wall, I Welters

#### Royal Liverpool University Hospital, Liverpool, United Kingdom

**Introduction:** Chronic lymphocytic inflammation with pontine perivascular enhancement responsive to steroids (CLIPPERS) was first described in 2010 [1]. Symptoms include diplopia and gait ataxia. Pontine lesions are a commonly seen on magnetic resonance imaging. Patients display a favourable response to glucocorticosteroid therapy.

**Methods:** A 54 year-old man with no significant past medical history presented to the emergency department with a 10-day history of vomiting and feeling generally unwell. He suffered from dizziness, ataxia, diplopia, bilateral nystagmus, taste and hearing disturbances. Within 24 hours of admission he developed right sided weakness. GCS dropped to 8 and he required intubation. He had downgoing plantar reflexes, myoclonic jerks, tonic-clonic movements of lower limbs and spasticity of both upper and lower limbs. Seizures occurred daily and were terminated with lorazepam. Two weeks after admission, the patient developed pyrexia of 42^0^ C and required cooling for 7 days.

**Results:** The patient was treated for infective encephalitis with amoxicillin, ceftriaxone and acyclovir. Cerebrospinal fluid showed a white cell count of 96 cells/mcl (40% polymorphs and 60% lymphocytes). A second lumbar puncture a week later had a white cell count of 72 (5% polymorphs and 95% lymphocytes). All cultures, viral PCR and cryptococcal antigens were negative. The first MR head showed high T2 signal and swelling in the pons, which extended into the cerebral peduncle on the left. This area and the middle cerebral peduncles had high signal on the FLAIR sequence. There was restricted diffusion within the pons and middle cerebellar peduncle. A second MR scan identified several irregular regions of enhancement within the midbrain and pons, leading to a diagnosis of CLIPPERS. High-dose methylprednisolone was commenced intravenously. The patient’s clinical condition improved rapidly. He was alert and followed commands within days. Nystagmus persisted, but he had no further spasticity. A third MR after five days of steroid treatment described significant improvement in the pontine lesions, but a small focus of high signal remained. Midbrain oedema present on previous scans had resolved.

**Conclusions:** CLIPPERS is a diagnosis of exclusion, with patients often initially treated for stroke or encephalitis. Pyrexia has not been reported in cases of CLIPPERS. Central pyrexia is common in neurological conditions. An infective cause was not identified, however, with no response to antimicrobials in the absence of positive cultures, central pyrexia is likely. The rapid response to steroids supports a diagnosis of CLIPPERS.

**Reference**

1. Pittock S et al. Brain 2010;13:2626

## P208 Comparison of successful rate between ultrasound guided lumbar puncture and surface landmark method for difficult LP patients in ED; a randomized controlled trial

### P Tansuwannarat, P Sanguanwit

#### Ramathibodi hospital, Bangkok, Thailand

**Introduction:** Lumbar puncture (LP) is the main procedure to obtain the diagnosis of meningitis and subarachnoid hemorrhage. However the success rate could be compromised in difficult LP patients such as patients with obesity or scoliosis. Ultrasound-guided LP (UGLP) was proposed as a potential method to improve the success rate of this procedure.We aimed to investigate the success rate of UGLP compared to surface-landmarked LP (SMLP) in patients who visited an Emergency Department at Ramathibodi Hospital, Bangkok, Thailand.

**Methods:** This is a prospective randomized controlled trail from August 2015 to July 2016. All adult (>18 years old) patients with a diagnosis of suspected meningitis were included. Difficult LP patient was defined as patient with bone mineral density of 25 kg/m2 or more and scoliosis. Primary outcome was the success rate of first LP attempt. Secondary outcome included number of attempts, time to complete the procedure, and post-procedure complication.

**Results:** There were 40 patients included with a mean age of 60 + 19.34 years and 53% were male. The majority of patients were suspected for meningitis (82%).There were 20 patients in each group. Success rate at the first LP attempt was greater in UGLP group compared to SMLP group [16(80%) vs 7(35%), p 0.009]. Median time (minutes) to complete the procedure was shorter in UGLP compared to SMLP group [(5 vs. 13.5), p 0.002]. Post-procedure complication (blood-contaminated CSF) occurred less in UGLP compared to SMLP group [(2(10%) vs. 6(30%), p 0.235

**Conclusions:** UGLP significantly improve the success rate of first LP attempt and decrease time to complete the procedure in difficult LP patients.

**Reference**

UGLP significantly improve the success rate of first LP attempt and decrease time to complete the procedure in difficult LP patients.

**Table 6 (abtsract P208).** Generalized Characteristics From All Enrolled Patients

Characteristics	US Guided LP(20)	Surface landmark LP(20)	P value
Sex(Male)	9(45%)	12(60%)	0.342
Age > 50	17(85%)	13(65%)	0.144
BMI	27.14	27.48	0.497
Underlying disease(Yes)	14(70%)	17(85%)	0.451

**Table 7 (abtsract P208).** Compare sucess rate in 1st attempt and complication between USLP VS SLLP

Parameters	US Guided LP(20)	Surface landmark LP(20)	P value
Success rate in 1st attempt	16(80%)	7(35%)	0.009
Time to success median(range)	5 (3-18)	13.5(5-30)	0.002
Complication(Yes)	2(10%)	6(30%)	0.235

## P209 Acid-base characteristics of the cerebrospinal fluid of patients with subarachnoid hemorrhage and in control subjects according to Stewart’s approach

### T Langer ^1^, M Carbonara^2^, A Caccioppola^1^, C Ferraris Fusarini^2^, E Carlesso^1^, E Paradiso^1^, M Battistini^1^, E Cattaneo^1^, F Zadek^1^, R Maiavacca^2^, N Stocchetti^1^, A Pesenti^1^

#### ^1^University of Milan, Milan, Italy; ^2^Fondazione IRCCS Ca’ Granda, Ospedale Maggiore Policlinico, Milan, Italy

**Introduction:** The pathophysiology of the acid-base equilibrium of the cerebrospinal fluid (CSF) is important, as it influences respiration [1]. Aim of the present study was to describe CSF acid-base of patients with subarachnoid hemorrhage (SAH) and compare them with control subjects.

**Methods:** In patients with SAH, a CSF sample was taken from the external ventricular drain simultaneously with an arterial blood sample to measure electrolytes, albumin, phosphates, PCO[sub]2[/sub] and pH. A similar procedure was performed in patients without significant comorbidities undergoing spinal anesthesia for elective surgery. For each sample the Strong Ion Difference (SID[sub]CSF[/sub]) and the total concentration of weak, non-volatile acids (A[sub]TOT[/sub]) were calculated using standard formulae. Furthermore plasma SID and its difference with SID[sub]CSF[/sub] was computed (Δ SID). Comparison between groups was performed via t-test or Rank Sum Test, as appropriate.

**Results:** Ten patients with SAH (55 ± 16 years) and 7 controls (56 ± 11 years) were enrolled. Acid-base results are summarized in Table. In SAH patients, SID[sub]CSF[/sub] was lower, mainly due to a higher lactate (3.2 ± 1.5 vs. 1.5 ± 0.3 mEq/L, p < 0.001) and chloride concentration (125 ± 2 vs. 120 ± 2 mEq/L, p < 0.001). Despite the acidifying effect of lower SID[sub]CSF[/sub], a lower CSF PCO[sub]2[/sub], with unchanged CSF A[sub]TOT[/sub] led to CSF pH values similar to controls. Finally, despite a lower plasma SID in the SAH group, Δ SID was significantly higher in these patients.

**Conclusions:** The CSF of patients with SAH, as compared to control subjects, has lower SID and PCO[sub]2[/sub], but similar pH. In these patients, the reduction in SID[sub]CSF[/sub] is not coupled with a similar reduction in plasma SID, leading to a higher Δ SID, i.e. a more pronounced acidification of CSF as compared to plasma.

**Reference**

1. Langer T et al. Intensive Care Med 42(3):436–9, 2016

**Table 8 (abstract P209).** SAH = subarachnoid hemorrhage; Data presented as mean ± standard deviation.

Variables	SAH patients	Control subjects	p-value
CSF PCO_2_ [mmHg]	37 ± 6	47 ± 3	<0.001
CSF SID [mEq/L]	22.0 ± 1.7	27.0 ± 1.4	<0.001
CSF A_TOT_ [mmol/L]	1.3 ± 0.9	1.2 ± 0.2	0.13
CSF pH	7.38 ± 0.08	7.35 ± 0.03	0.53
Plasma SID [mEq/L]	34.4 ± 1.8	36.5 ± 1.6	0.02
Δ SID [mEq/L]	12.3 ± 2.1	9.5 ± 2.2	0.02

## P210 Polyuria and natriuresis after aneurysmal subarachnoid haemorrhage

### A Ramos^1^, F Acharta^1^, J Toledo^1^, M Perezlindo^1^, L Lovesio^1^, A Dogliotti^2^, C Lovesio^1^

#### ^1^Sanatorio Parque, Rosario, Argentina; ^2^Grupo Oroño, Rosario, Argentina

**Introduction:** Natriuresis and polyuria are common events after aneurysmal subarachnoid haemorrhage (aSAH). A relationship has been found between polyuria, cerebral salt wasting syndrome (CSWS) and vasospasm [1]. The aim of this study is to determine the relationship between creatinine clearance and natriuresis, and to identify variables related to natriuresis.

**Methods:** During 2 years (2014-2016) 29 patients with aSAH and polyuria were identified. The tomographic characteristics and neurological clinical scores were considered. 24-hour urine was obtained in individuals with polyuria (>3 liters / day). CSWS was defined as the 75th percentile (>923 mEq / l urinary sodium). Symptomatic vasospasm was defined as clinical deterioration confirmed with cerebral angiography.

**Fig. 11 (abstract P210).**
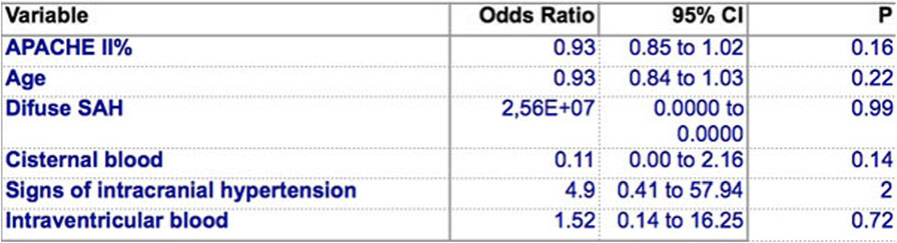
Multivariate analysis

**Fig. 12 (abstract P210).**
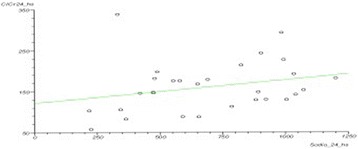
Correlation between creatinine clearance and natriuresis

**Results:** 29 patients were included, eight of them (27.6%) developed CSWS. No patient presented hyponatremia. No predictors of CSWS were found (Fig. 11). Neither sodium in 24 hours (AUC: 0.57, 95% CI: 0.37-0.75 p = 0.5), nor the volume of diuresis predicted symptomatic vasospasm (AUC: 0.57, 95% CI: 0.37-0.75 p = 0.5). There was no correlation between creatinine clearance and natriuresis (p = 0.17) (Fig. 12).

**Conclusions:** We found no relationship between polyuria, natriuresis and symptomatic vasospasm.

**Reference**

1. Brown RJ et al. Polyuria and cerebral vasospasm after aneurysmal subarachnoid hemorrhage. BMC Neurology. 15:1–7, 2015.

## P211 Coma in late night amsterdam; do not forget the travel history

### N Schroten^1^, B Van der Veen^2^, MC De Vries^2^, J Veenstra^2^

#### ^1^VUMC, Amsterdam, Netherlands; ^2^OLVG, Amsterdam, Netherlands

**Introduction:** Unconscious young patients are admitted to hospitals in Amsterdam nearly on a daily basis, mostly due to intoxications. However, the following case underscores that routine laboratory and imaging investigations do not replace a detailed interrogation.

**Methods:** A 17-year old woman was found unconscious in the early morning. Her family mentions she had complained about headaches, but no other complaints or fever. At the emergency department her temperature was 37 °C, respiratory frequency 31/min and pulse 120/min. She was unresponsive and had uncontrolled jerking movements, without signs of lateralisation and with normal stem reflexes. Laboratory analyses showed C-reactive protein 36 mg/l; Leukocytes 6,4x10^9/l; Haemoglobin 5,4 mmol/l; Mean corpuscular volume 86 fl; Thrombocytes 156x10^9/l; LD 318 U/l, bilirubin 28 μ mol/l. Cerebral computer tomography and analysis of cerebrospinal fluid were unremarkable. Empiric therapy with broad-spectrum antibiotics, acyclovir and dexamethasone were started.

**Results:** At the intensive care unit she developed a fever up to 40 °C and haemoglobin levels decreased to 3,8 mmol/l. Further interrogation of the parents reported a visit to Ghana for 2 weeks 3 months before. She had taken mefloquine prophylactically. A thick smear was positive: plasmodium falciparum with a parasitaemia of 0,3%. After artesunate 2,4 mg/kg twice daily intravenously the patient recovered rapidly. On follow up she had minor concentration problems.

**Conclusions:** Cerebral malaria is a diffuse symmetric encephalopathy. Children are at a higher risk than adults. Focal signs are unusual. CT scans and cerebrospinal fluid analysis are usually unremarkable. It is important to note that cerebral malaria may have a prolonged incubation time, especially in patients using malaria prophylaxis, like in the current case, and may be lethal even at a low parasitemia. Early treatment is vital. The authors confirm they have received informed consent to publish from the patient.

**References**

**1.** Jakka SR, Veena S, Atmakuri RM, Eisenhut M. Characteristic abnormalities in cerebrospinal fluid biochemistry in children with cerebral malaria compared to viral encephalitis. Cerebrospinal Fluid Res. 2006;3:8.

2. Giha HA1, A-Elbasit IE, A-Elgadir TM, Adam I, Berzins K, Elghazali G, Elbashir MI. Cerebral malaria is frequently associated with latent parasitemia among the semi-immune population of eastern Sudan. Microbes Infect. 2005 Aug-Sep;7(11-12):1196–203.

## P212 Healthcare-associated infections in the neurological intensive care unit: 6-year surveillance study at a major tertiary care center

### YB Abulhasan^1^, S Rachel^1^, M Châtillon-Angle^1^, N Alabdulraheem^1^, I Schiller^2^, N Dendukuri^2^, M Angle^1^, C Frenette^1^

#### ^1^Montreal Neurological Institute and Hospital, Montreal, Canada; ^2^McGill University, Montreal, Canada

**Introduction:** To report incidence rates, pathogens distribution, and patient related outcomes of healthcare-associated infections (HAIs) in a neurological intensive care unit (Neuro-ICU) patient population over a 6-year period.

**Methods:** We are presenting a prospective cohort study of all patients admitted in a 14 bed Neuro-ICU part of a highly-specialized referral center from April 1, 2010 to March 31, 2016. Surveillance for HAIs was carried by infection control professionals who reviewed laboratory results and targeted specific clinical indicators to match National Healthcare Safety Network infection criteria. Rates were calculated per 1,000 patient days and per 1,000 device days. Differences in infection rates were analyzed by emergency neurocritical care diagnostic categories. We studied the association between: i) primary diagnosis and infection using Cox proportional hazards model, ii) infection and length of stay using linear regression, and iii) infection and mortality using Cox proportional hazards model. Yearly objectives were set to reduce HAIs with implementation of targeted infection control measures.

**Results:** There were 6,034 Neuro-ICU admissions resulting in 20,845 Neuro-ICU days. A total of 228 HAIs were identified. Pooled mean HAI incidence rates were pneumonia 17.1, UTI 3.7, ventriculostomy-associated infection (VAI) 0.8, central line-associated blood stream infection (CLABSI) and primary bacteremia 0.2, Clostridium difficile-associated diarrhea (CDAD) 0.6, and other HAIs 0.7 per 1,000 Neuro-ICU days. For device-associated infections, which accounted for 80.7% of HAIs, pooled mean rates were 18.5 ventilator-associated pneumonia (VAP), 5.0 catheter-associated urinary tract infection (CAUTI), 4.0 VAI, and 0.6 CLABSI episodes per 1,000 device days. Among the various diagnostic categories, intracerebral/intraventricular hemorrhage (ICH/IVH), subdural hematoma, seizure/status epilepticus, and subarachnoid hemorrhage were associated with the highest pooled HAIs with incidence rates of 22.2, 21.3, 16.5, and 15.2 per 1,000 Neuro-ICU days respectively. Pathogen frequencies were S. aureus (27%) and Klebsiella species (12.2%) for pneumonias, E. coli (44.8%) for UTIs, S. epidermidis (57.9%) for VAIs. Among patients with HAIs, all-cause 30-day case mortality proportion was 9.7% and occurred a median of 14 days after the HAI. Prolonged Neuro-ICU length of stay was strongly associated with all HAIs (P < 0.05).

**Conclusions:** Pneumonia, UTI and VAI are the commonest HAIs among our cohort of neurocritical care patients with pooled rates of HAIs most pronounced among ICH/IVH patients.

## P213 Paradoxical cerebrovascular hemodynamic changes with nicardipine

### S Lahiri^1^, K Schlick^1^, SA Mayer^2^, P Lyden^1^

#### ^1^Cedars-Sinai Medical Center, Los Angeles, CA, United States; ^2^Mount Sinai Medical Center, New York, United States

**Introduction:** IV nicardipine is commonly used for blood pressure reduction. Few studies have described its effects on cerebrovascular hemodynamics as measured by transcranial Doppler (TCD) waveform analysis and pulsatility index (PI).

**Methods:** The data presented are from patients who underwent TCD monitoring before, after, or during nicardipine administration.

**Results:** TCD waveforms during nicardipine infusion are characterized by a prominent systolic peak and dicrotic notch. Systolic deceleration is more pronounced and PIs are significantly elevated in patients on nicardipine (p < 0.05).

**Conclusions:** This study provides first evidence of paradoxical intracranial vasoconstriction associated with nicardipine. This is a consistent finding in patients treated with IV nicardipine and is contradictory to what is expected from a vasodilator and anti-hypertensive.

**Fig. 13 (abstract P213).**
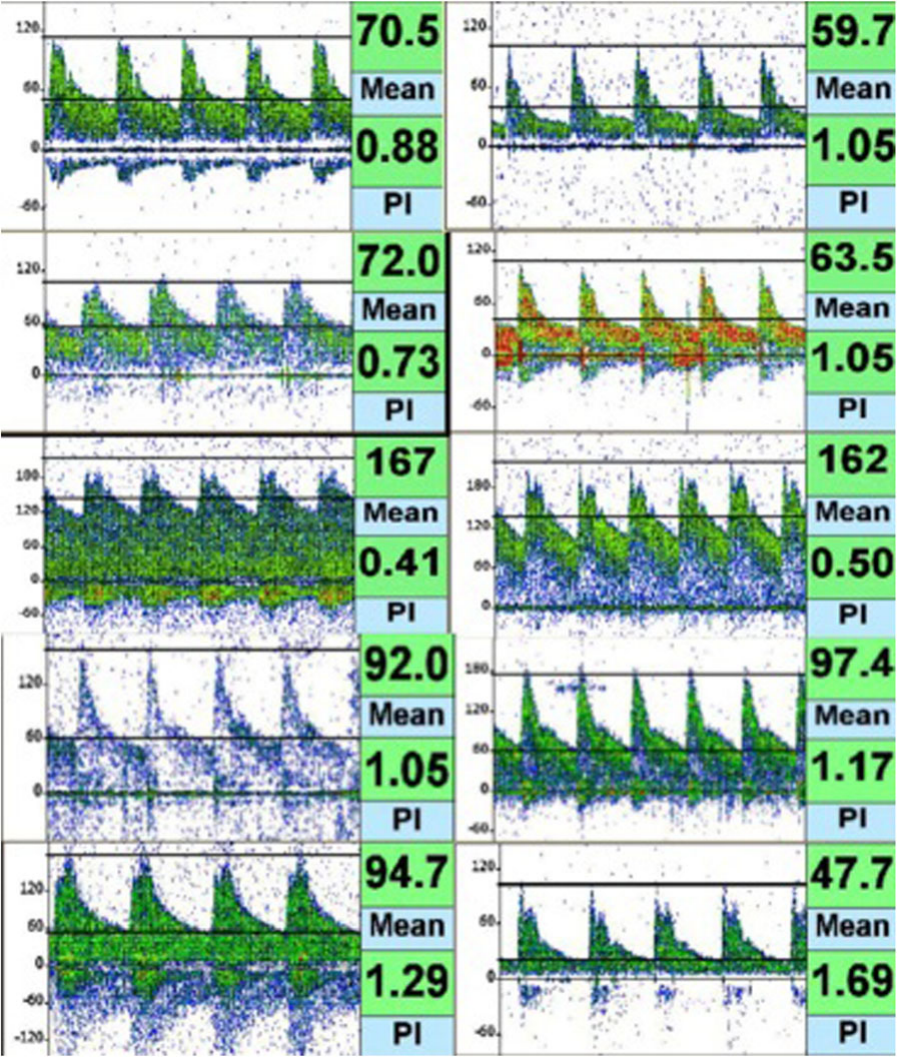
Left panel without nicardipine, right panel with nicardipine

## P214 Clinical manifestations and diagnosis of patients with cerebral venous thrombosis: retrospective study and literature review

### M Akatsuka^1^, J Arakawa^1^, M Yamakage^2^

#### ^1^Japanese Red Cross Kitami Hospital, Kitami, Japan; ^2^Sapporo Medical University School of Medicine, Sapporo, Japan

**Introduction:** Cerebral venous thrombosis (CVT) is a rare neurovascular disorder with a highly variable presentation that accounts for only 0.5% of all cases of stroke [1]. It can lead to neurologic impairment. There have been some studies and case reports about CVT; however, there is a lack of information on the incidence and clinical features of CVT due to its rarity. We, therefore, conducted this study to clarify important aspects of the epidemiology, diagnosis, and prognosis of CVT.

**Methods:** We carried out a retrospective observational study of all patients with a diagnosis of CVT in our hospital between January 2003 and October 2016. From the electronic patient data management system, we obtained information on the patient• fs age, gender, symptoms, risk factors, location of thrombosis, and outcome as well as images. We also performed a systemic review of the literature for CVT using a multiple web research platform (PubMed) from 2000 to 2016.

**Results:** Four patients were diagnosed with CVT in our hospital during the study period. A total of 17 articles were found, from which 1220 cases were determined eligible for review and the author• fs cases were added. The mean age of the patients was 39.3 years (standard deviation [SD]: 17.4 years), and 70.8% of the patients were female. The most frequent symptom was headache (884 cases, 72.2%). The use of an oral contraceptive was one of the most frequent predisposing risk factors (290 patients, 33.5%). For diagnosis of CVT, the majority of patients underwent computed tomography (CT) (80.5%), magnetic resonance imaging (MRI) (83%), and magnetic resonance venography (MRV) (66.8%). The most frequent site of involvement was the superior sagittal sinus (543 cases, 44.4%). The hospital mortality rate was 5.4%. There was no association between hospital mortality and location of thrombosis. The results of CT or MRI were normal in some cases, but the results of MRV were abnormal in all cases and lead to the diagnosis of CVT.

**Conclusions:** Patients presenting with headache, especially patients taking an oral contraceptive, should be examined carefully. Not only CT but also MRI in combination with MRV has high sensitivity and specificity for establishing a diagnosis of CVT. Although there is no typical red flag symptomatology, recognition of CVT is important.

**Reference**

[1] Bousser MG et al.: Lancet Neurol 6: 162–70, 2007

## P215 Diaphragmatic electric activity during apnea testing for brain death determination

### J Rubio^1^, JA Rubio Mateo-Sidron^2^, R Sierra^1^, M Celaya^1^, L Benitez^1^, S Alvarez-Ossorio^1^

#### ^1^Hospital Universitario Puerta del Mar, Cadiz, Spain; ^2^Hospital Xanit, Benalmadena, Malaga, Spain

**Introduction:** Absence of respiratory control reflexes in the brainstem in response to hypercapnic stimulation through the observation of the thoracic and abdominal movements, positive apnea testing (AT), is a key component in the clinical assessment of brain death (BD). False negative results of AT may occur due to ventilatory auto-triggering which could hamper and delay BD determination. Electrical activity of the diaphragm (EAdi) reflects the neural respiratory drive. We hypothesized that EAdi monitoring would add accuracy and safety to AT procedure.

**Methods:** We performed a single centre prospective observational study of adult patients admitted to the ICU with devastating acute brain injury and clinical examination consistent with BD. All patients were mechanically ventilated using the Servo-i® and the conventional nasogastric tube was replaced with an EADi catheter (16Fr/125 cm; Maquet Critical Care, Solna, Sweden) designed to be used with neurally adjusted ventilation. Stable patients on ventilator control mode were switched to Pressure Support during the period of apnea. Respiratory movements, arterial pressure, hearth rhythm and SpO2 were continuously monitored and airway pressure, airflow and EADi were also saved using a Ventilation Record Card for later analysis. Study variables were recorded at 3 time points: basal (T1), start of AT (T2) end of AT (T3). AT duration and complications during the procedure were recorded.

**Results:** We included 8 patients in the study. EADi signals recorded during AT were tonic and with very low voltage. EADi amplitude values at T2 and T3 ranged between 0,2 ± 0,15 μV to 0,5 ± 0,52 μV and 0,2 ± 0,07 μV to 0,55 ± 0,52 μV respectively. The median procedure duration was 12 ± 2,5 min. Sensitive auto-triggering was observed in a case. The AT was positive and completed in all patients and no severe complications were noted.

**Conclusions:** We found that EADi monitoring and analysis is a safe and effective tool for diagnosing apnea during BD confirmation, avoiding false negative diagnosis based on direct observation. This strategy of rapid implementation, operator-independent, minimally invasive and low cost, may be introduced in the AT protocols.

## P216 Usefulness of a method for doing apnea testing during brain death determination

### J Rubio^1^, JA Rubio Mateo-Sidron^2^, R Sierra^1^, A Fernandez^1^, O Gonzalez^1^

#### ^1^Hospital Universitario Puerta del Mar, Cadiz, Spain; ^2^Hospital Xanit, Benalmadena, Malaga, Spain

**Introduction:** Apnea Testing (AT) is a key element for Brian Death (BD) determination but accepted guidelines including the procedure itself do not exist. All current methods of AT have as main purpose to elevate CO2 and observe the patient for any spontaneous effort by close observation of respiratory movements that may sometimes be subtle and doubtful. Moreover, commonly AT practice involve disconnection of ventilator circuit, adding an adjustable CPAP valve to the distal extremity of the T-piece extension in order to try to preserve oxygenation. This study was aimed to evaluate an alternative AT method that allows the patient to stay connected to the mechanical ventilator circuit during AT procedure and avoid the disadvantages of the current methods.

**Methods:** We performed a single centre prospective observational study of adult patients admitted to the ICU with devastating acute brain injury and clinical examination consistent with BD. All patients were mechanically ventilated using the Servo-i® ventilator (Maquet Critical Care, Solna, Sweden). After 10 minutes of preoxygenation, controlled mode ventilation was switched to Pressure Support (PS) during the whole test, setting level of PS to 8 cmH2O above PEEP 5 cmH2O, Trigger Pressure -2 cmH2O and FiO2 100% with backup ventilation off. Patients were closely monitored throughout the procedure by clinical observation. Airway pressure, airflow and volume waveforms were displayed on the ventilator screen. Patient and screen data were saved to a Ventilation Record Card for later analysis. Duration of AT, invasive arterial pressure, heart rate and SpO2 were recorded. Physiologic, ventilatory mode and settings, respiratory mechanics and arterial blood gases were collected at 3 time points: basal (T1), start of AT (T2), end of AT (T3). After AT ventilator was switched to prior settings.

**Results:** Nine patients were studied. Main data collected are showed in the table. Analysis of pressure and flow waveform tracings showed absence of airflow and maintenance of PEEP at stable levels. A case of sensitive auto-triggering was clearly observed due to the selected PS level but the procedure did not have to be aborted. There were no complications or discontinued procedures.

**Conclusions:** This method appears to be safe and effective for AT during BD assessment. We speculate that these findings could have a significant impact if the strategy used really contributes to increase numbers of organ procurements and under the best possible conditions.

**Table 9 (abstract P216).** Results

(mean ± SD)	T1	T2	T3
pH	7,43 ± 0,06	7,39 ± 0,06	7,2 ± 0,08
PaO2 (mmHg)	114 ± 45,9	298 ± 90,9	149 ± 129,2
PaCO2 (mmHg)	37 ± 5,6	39,7 ± 7,6	67 ± 12,2
SpO2 (%)	99 ± 0,8	99,9 ± 0,5	98,7 ± 4,8

## P217 Effect of HHH therapy on CBF after severe subarachnoid hemorrhage: regional cerebral blood flow studied by bedside Xenon-enhanced CT

### H Engquist, E Rostami, P Enblad

#### Uppsala University, Dpt of Neuroscience/Neurosurgery, Uppsala, Sweden

**Introduction:** Detection and management of delayed cerebral ischemia (DCI) after severe subarachnoid hemorrhage (SAH) is difficult, and tools are lacking to guide the therapy. In the present study, bedside Xenon-enhanced computerized tomography (XeCT) was used to assess regional cerebral blood flow (rCBF) prior to clinical suspicion of DCI and during treatment to resolve DCI.

**Methods:** Patients diagnosed with SAH and requiring mechanical ventilation, were prospectively enrolled in the study. XeCT, using inhaled stable Xenon as an inert contrast agent, was scheduled at day 0-3, 4-7 and 8-12. Patients clinically diagnosed with DCI received standard five-day treatment to augment CBF by hypervolemia, hemodilution and hypertension (HHH-therapy). XeCT at 0-2 days before start of HHH was considered as baseline, and next XeCT was performed during the therapy. Corresponding data were collected for non-DCI patients with XeCT measurements in matching time-windows (day 2-4 and 5-8 respectively).

**Results:** Nineteen patients who later developed DCI were included, and twenty-six patients without DCI were identified as a comparison group. There were no significant differences in the systemic hemodynamic parameters or pCO[sub]2[/sub] before vs during HHH, although a slightly elevated systolic blood pressure (SBP) was noted. Median global cortical CBF for the DCI group increased from 28.0 (IQR 24.6-34.5) to 37.8 (IQR 26.9-41.7) ml/100 g/min, P = 0.008. Median rCBF of the worst vascular territory increased from 19.8 (IQR 14.8-26.5) to 27.5 (IQR 17.4-34.5) ml/100 g/min, P = 0.033. For the group with no DCI, global CBF at baseline was higher and there was no significant change at the XeCT in the second time-window.

**Conclusions:** The initial low global CBF found in patients diagnosed with DCI, increased significantly during HHH-therapy despite modest changes of SBP. A concomitant increase in rCBF was also found in the vascular territories with worst rCBF. The increase in CBF may be related to the HHH-therapy, but a time-dependent natural recovery of CBF cannot be ruled out. XeCT may be helpful in managing poor grade SAH patients.

**Fig. 14 (abstract P217).**
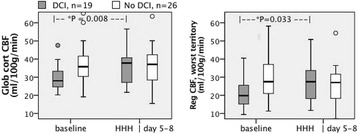
CBF at baseline and during HHH-therapy in DCI

## P218 Spontaneous primary intracerebral hemorrhage: factors influencing poor outcome. A two-centers series

### J Toledo^1^, A Ramos^1^, F Acharta^1^, L Canullo^1^, J Nallino^1^, A Dogliotti^2^, C Lovesio^1^

#### ^1^Sanatorio Parque, Rosario, Argentina; ^2^Grupo Oroño, Rosario, Argentina

**Introduction:** Patients presenting intracerebral hemorrhage (ICH) have commonly been reported to have a poor prognosis. We prospectively analyzed data from 98 patients with spontaneous primary ICH to evaluate possible predictors of poor outcome defined as a Glasgow Outcome Scale (GOS) < =3 at hospital discharge after ICH.

**Methods:** Between june 1, 2013 and september 30, 2016; 98 patients with ICH were treated. On admission data including patient characteristics, clinical findings, radiologic features, and functional neurologic outcome were assessed and further analyzed.

A multivariate analysis was performed to identify predictors of poor outcome.

**Results:** 98 patients were included in the study and 46% of them had an unfavorable evolution. In multivariate analysis, patients taking antiplatelet therapy at hospital admission (OR: 15.7, 95% CI: 1.03-241; p = 0.047). and elevated ICH score had worse outcome. For every point of ICH score, the probability of poor outcome increases in 43% (p < 0.001).

**Conclusions:** We have found that the ICH score and receiving antiplatelet agents are associated with poor prognosis in patients with spontaneous ICH.

## P219 Agitation after mild to moderate traumatic brain injury in the intensive care unit

### M Perreault ^1^, J Talic^2^, AJ Frenette^3^, L Burry^4^, F Bernard^3^, DR Williamson^3^

#### ^1^The Montreal General Hospital, Montreal, Canada; ^2^University of Geneva, Geneva, Switzerland; ^3^Hôpital Sacré-Coeur de Montreal, Montreal, Canada; ^4^Mount Sinai Hospital, Toronto, Canada

**Introduction:** Traumatic brain injury (TBI) is a leading cause of mortality and disability worldwide. Among TBI complications, agitation is a frequent behavioural problem. Agitation leads to harm and treatment interference, unnecessary chemical and physical restraints, increased hospital length of stay, delayed rehabilitation and impedes functional independence. Agitation is reported in 70% of TBI patients in rehabilitation units but not well described during ICU. The objective of this study was to describe the frequency, timing and clinical impact of agitation in mild (GCS 13-15) to moderate (GCS 9-12) TBI in the intensive care unit (ICU).

**Methods:** This was a prospective observational study of mild to moderate TBI adult patients in two critical care trauma centers. Patients aged > = 18 years admitted for more than 48 hours were screened. We excluded patients with severe TBI (GCS 3-8), RASS -4 or -5 during ICU stay, pre-existing cognitive deficiency, inability to communicate in French or English and deafness or blindness were excluded. Agitation was defined as a RASS score of +2 or +3 and assessed during three time periods (00:00 to 07:59, 08:00 to 15:59 and 16:00 to 23:59). Agitated and non-agitated patients were compared using a T-test or Mann Whitney as appropriate for continuous variables and Chi-square test for dichotomous variables.

**Results:** During an 8-month period, 226 patients were assessed for eligibility. In total, 62 patients (74.2% men) with a median age of 58 years (IQR 33), a mean APACHE II score 11.3 ± 6.5 and mean ISS of 23.3 ± 9.3 were enrolled. At least one episode of agitation was reported in 72.6% of patients. Persistent agitation defined as at least 2 episodes on separate days was reported 54.8% of patients. Persistently agitated patients were more often mechanically ventilated (79.4% vs 46.4%; p = 0.01), had more moderate TBIs (47.1% vs 10.7%; p = 0.02) and had higher APACHE II scores (12.6 +/- 6.1 vs 9.8+/- 6.6; p = 0.09). Episodes of agitation presented at night-time, daytime and evening in 20.6%, 22.8% and 22.9%, respectively. Persistent agitation was associated with a longer length of stay (9.1 vs 4,7 days; p = 0.001), increased restraint use (82.4% vs 28.6%;p < 0.001), increased antipsychotic use (55.9% vs 7.1%; p < 0.001) and increased self-removal of arterial and central venous catheters (29.4% vs 3.6%;p = 0.009) and nasogastric tubes (20.6% vs 3.6%;p = 0.06).

**Conclusions:** Agitation is common in mild to moderate TBI ICU patients, occurs at all times of the day and is associated with increased use of restraints, antipsychotics and potential self-harm.

## P220 Acute subdural hematoma after isolated traumatic brain injury: associated factors and prediction of lethal outcome

### D Adukauskiene^1^, J Cyziute^2^, A Adukauskaite^3^, L Malciene^4^

#### ^1^Hospital of Lithuanian University of Health Sciences, Kaunas, Lithuania; ^2^Lithuanian University of Health Sciences, Kaunas, Lithuania; ^3^Innsbruck Medical University Hospital, Innsbruck, Austria; ^4^Klaipeda University Hospital, Klaipeda, Lithuania

**Introduction:** The aim of this study was to determine associated factors with lethal outcome also prediction of it in case of acute subdural hematoma (ASH) after isolated traumatic brain injury (ITBI).

**Methods:** Retrospective analysis of 162 patients with ASH after ITBI treated in Neurosurgical Intensive Care Unit in Hospital Kaunas Clinics of Lithuanian University of Health Sciences during two years was carried out.

**Results:** Sixty–seven patients (41%) of 162 have died with ASH after ITBI. Twelve patients (31%) of 39 have died in age group of < = 44 years, 16 patients (37%) of 43 in group of 45 – 54 years, 14 patients (36%) of 39 in group of 55 – 64 years, but 25 patients (61%) of 41 in group of > = 65 years, p < 0.003. Twenty–four patients (26%) of 93 have died with pupillary light reflex and 43 patients (62%) of 69 without of it, p < 0.001. Five patients (25%) of 20 have died in group of Glasgow Coma Scale (GCS) score 12 – 15, 5 patients (20%) of 25 in group of 9 – 11, but 57 patients (49%) of 117 in group of 3 – 8, p < 0.002. Twenty–one patient (28%) of 75 has died with white blood cell count <10.1 x 10^9/l, but 46 patients (53%) of 87 with > = 10.1 x 10^9/l, p < 0.001. Five patients (17%) of 30 have died with glycemia 3.3 – 5.5 mmol/l and 62 patients (47%) of 132 with glycemia > = 5.6 mmol/l, p < 0.001. One patient (5%) of 22 has died in group of APACHE II score < = 10 points, 12 patients (24%) of 50 in group of score 11 – 15, but 54 patients (60%) of 90 in group of score > = 16, p < 0.001. Fifty–two patients (63%) of 82 have died in the group of estimated lethal outcome risk >25%, p < 0.001 (0.95CI: 0.53 – 0.74) and prognostic test sensitivity was found to be 78%, specificity 76%.

**Conclusions:** The mortality rate of acute subdural hematoma after isolated traumatic brain injury was 41%. Factors associated with lethal outcome were estimated to be age > = 65 years, absence of pupillary light reflex, Glasgow coma scale score 3 – 8, white blood cell count > = 10.1 x 10^9/l, glycemia > = 5.6 mmol/l and APACHE II score > = 16 points on the first day after trauma. Predicted lethal outcome has coincided with real mortality when the risk of lethal outcome was higher than 25%.

## P221 Multimodal monitoring in critically ill polytrauma patient with traumatic brain injury (TBI)

### L Luca^1^, A Rogobete^1^, O Bedreag^1^, M Papurica^1^, M Sarandan^2^, C Cradigati^2^, S Popovici^1^, C Vernic^1^, D Sandesc^1^

#### ^1^University of Medicine and Pharmacy ”Victor Babes” Timisoara, Timisoara, Romania; ^2^Emergency County Hospital ”Pius Brinzeu”, Clinic of Anesthesia and Intensive Care ”Casa Austria”, Timisoara, Romania

**Introduction:** The most significant injury found in trauma patients is represented by traumatic brain injury which has the greatest impact on mortality. Intracranial pressure (ICP) monitoring is required in severe traumatic head injury because it optimizes treatment based on ICP values and cerebral perfusion pressure, respectively (CPP). The objective of the study was to identify the incidence of systemic complications of acute TBI and establishing a plan for diagnostic and therapeutic approach.

**Methods:** From a total of 64 patients admitted in the Intensive Care Unit "Casa Austria", Emergency County Hospital “Pius Brinzeu” Timisoara, Romania, between January 2016 and August 2016, only patients who received ICP monitoring (n = 10) were analysed. In the control group were analysed 13 patients. Depending on the time passed since trauma and to the time of ICP monitoring, the patients were divided into 3 categories (<18 hours, 19-24 hours, > 24 hours). There were also compared a series of clinical outcomes between the groups.

**Results:** Monitoring of ICP initiated after 36 hours of trauma reveals significantly higher values compared to monitoring in 24 hours ICP on demonstrating effective monitoring therapeutic management of critical patient. In monitoring patients who ICP was installed in the first 18 hours after trauma the length of stay in ICU was 12.5 ± 5 days compared to those from which the installation was done after 36 hours of trauma where the length of stay in ICU was 21.75 ± 9.64 days. After 120 hours of aggressive monitoring and therapy in accordance with the parameters analyzed, there is a decrease in the ICP and a normalization of values MAP or PPC. For patients who install monitoring was conducted in the first 18 hours after trauma, the mean of ICP was 22.99 ± 12.47 for those from which the installation was done within 19 to 24 hours after trauma the average value for ICP was 11.02 ± 2.09, and for patients whose installation was carried out after 36 hours of trauma, the mean of ICP was 29.86 ± 18.82 . It reported a relationship statistically significant between ICP and MAP values (p = 0.0008), respectively ICP and PPC (p = 0.0284), confirming that under impaired autoregulation of cerebral growth PIC TAM induce growth without significantly improving cerebral perfusion.

**Conclusions:** The multimodal monitoring in the management of the trauma patient a significant influence on the rate of survival.

## P222 Hypothermia in management of traumatic brain injury

### V Avakov^1^, I Shakhova^2^

#### ^1^Tashkent Medical Academy, Tashkent, Uzbekistan; ^2^Université de Strasbourg, Strasbourg, France

**Introduction:** It is found that high body temperature in patients with high intracranial pressure is burdening association [1] due neuroexcitotoxicity [2], which further damage the nerve cells and stimulate the autoimmune processes [3]. Mechanisms of injury, exacerbated by hyperthermia, can be mitigated by mild hypothermia.

**Methods:** We studied 47 patients (18-72 years) with traumatic brain injury. Level of consciousness of patients at admission to hospital was 3-8 points of GCS. According to the basic conditions of management and treatment, the patients were identicals.

**Fig. 15 (abstract P222).**
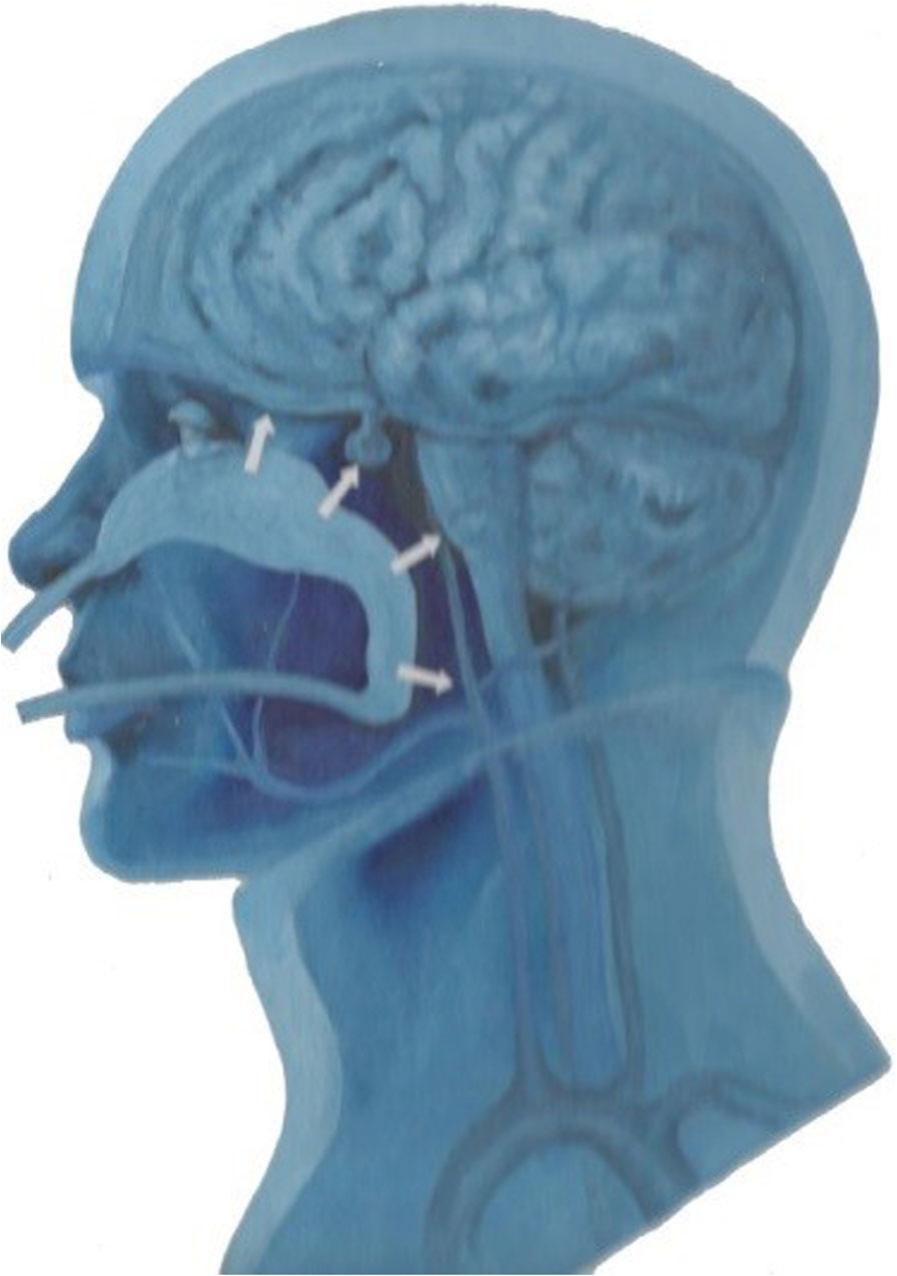
See text for description

However, first group of patients (n = 27) during initial 6 hours has been assigned to brain hypothermia by combination of nasopharyngeal cooling with cooling of cranial vault and carotid bifurcation projection (Fig. 15).

Duration of cooling was 33,5 ± 4,2 h; brain target temperature (in the ear canal)–33-34 °C, body (axillary)–35,5-36,5 °C.

**Results:** Malignant hyperthermia central genesis in patients of first group was not observed, while in the second group, its frequency increased from 30 to 55%.

Duration of patient’s stay in ICU and hospital was shorter by 1.4 times in first group; GOS was the most favorable in first group-4.2 points compared with 3.7 points in second group. Mortality rate was 42% versus 65%, respectively.

**Conclusions:** Nasopharyngeal cooling, added to the conventional had cooling for suppress the severity of ischemic processes in brainstem and the activity of thermoregulation center, promoted neuroprotection, improved neurological outcomes, survival, and reduced duration and cost of treatment.

**References**

1. Zdravev P., 1951

2. Irazuzta J.E. et al., 1999

3. Prandini M.N. et al., 2005

## P223 Citicoline in severe brain trauma: matched pair analysis suggests improved outcome

### H Trimmel ^1^, M Majdan^2^, GH Herzer^1^

#### ^1^Landesklinikum Wr. Neustadt, Wiener Neustadt, Austria; ^2^Department of Public Health, 91701 Trnava, Slovakia

**Introduction:** Goal-oriented management of severe traumatic brain injury (sTBI) from emergency site to intensive care unit can save the lives of millions of affected patients worldwide and/or improve their long-term outcome thus enhancing quality of life and saving enormous socio-economic costs. However, promising sTBI treatment strategies with neuroprotective agents, such as citicoline (CDP-choline), lacked evidence or produced contradictory results in clinical trials, some of them maybe due to inappropriate study design or insensitive methodology. As preceding evaluations provided evidence of beneficial outcome in citicoline treated sTBI patients at Wiener Neustadt Hospital (WNH), we aimed to investigate the potential role of citicoline administration in those patients.

**Methods:** In the course of a prehospital TBI project to optimize early TBI care within 14 Austrian Level I trauma centers, data on 778 TBI patients were prospectively collected. Unexpected superior outcome of WNH patients gave the impetus to compare patients from WNH with citicoline administration and matched patients from the other Austrian centers without citicoline use in a retrospective subgroup analysis. Patients with Glasgow Coma Scale score < 13 on site and/or Abbreviated Injury Scale of the region “head” > 2 were included.

**Results:** Our analysis revealed significantly reduced rates of ICU mortality (5% vs. 24%, p < 0.01), hospital mortality (9% vs. 24%, p = 0.035) and six months mortality (13% vs. 28%, p = 0.031), as well as of unfavorable outcome (34% vs. 57%, p = 0.015) and observed vs. expected ratio for mortality (0.42 vs. 0.84) in the citicoline group. Adjusted OR revealed significantly better odds for ICU survival (OR = 6.7, p = 0.014) and six months favorable outcome (OR = 2.6, p = 0.022) in citicoline treated patients.

**Conclusions:** Despite the limitations of a retrospective subgroup analysis the findings suggest a correlation between early and consequent citicoline administration and beneficial outcomes. Therefore, we aim to set up an initiative for a prospective randomized controlled trial with citicoline in sTBI patients.

**References**

1. Secades JJ. Citicoline: pharmacological and clinical review, 2010 update. Revista de neurologia. 2011;52 Suppl 2:S1–s62.

2. Brazinova A, Majdan M, Leitgeb J, Trimmel H, Mauritz W. Factors that may improve outcomes of early traumatic brain injury care: prospective multicenter study in Austria. Scandinavian journal of trauma, resuscitation and emergency medicine. 2015;23:53.

**Table 10 (abstract P223).** Demographic and baseline characteristics of the treatment groups

Variable	Citicoline WNH (N = 67)	Control Austrian centers (N = 67)	p
Sex, N (% male)	52 (78%)	50 (75%)	0.839
ISS, mean (SD)	27.4 (12.4)	31.7 (17.3)	0,1
Total GCS assessed at admission, mean (SD)	6.4 (4.7)	6.0 (4.2)	0.662
Pupillary reactivity in field, N (%)			0.702
Both reactive	41 (61%)	36 (54%)	
One reactive	2 (3%)	2 (3%)	
None reactive	3 (5%)	6 (9%)	
Unknown	21 (31%)	23 (34%)	
Pupillary reactivity at admission, N (%)			0.416
Both reactive	28 (42%)	29 (43%)	
One reactive	2 (3%)	5 (8%)	
None reactive	1 (2%)	3 (5%)	
Unknown	36 (54%)	30 (45%)	
Rotterdam CT score, N (%)			0.052
1	2 (3%)	2 (3%)	
2	22 (34%)	19 (31%)	
3	31 (48%)	17 (28%)	
4	7 (11%)	19 (31%)	
5	2 (3%)	3 (5%)	
6	0	1 (2%)	
Subarachnoid hemorrhage, N (% Yes)	39 (58%)	41 (61%)	0.861
Epidural hematoma, N (% Yes)	8 (12%)	13 (19%)	0,342
Subdural hematoma, N (% Yes)	37 (55%)	43 (64%)	0.379
Prehospital hypotension, N (% Yes)	1 (2%)	4 (6%)	0.328
Prehospital hypoxia, N (% Yes)	5 (8%)	12 (18%)	0.19
Prehospital intubation, N (% Yes)	33 (49%)	40 (60%)	0.057
Predicted six months mortality, mean % (SD)	30.7% (18.6)	33.4% (21.7)	0.682
Predicted six months unfav. outcome, mean % (SD)	51.8% (21.6)	53.9% (23.7)	0.626

**Table 11 (abstract P223).** Outcomes at hospital discharge and six months post trauma

Variable	Citicoline (N = 67)	Control (N = 67)	p
ICU mortality, % (N)	5% (3)	24% (16)	<0.01
Hospital mortality, % (N)	9% (6)	24% (16)	0.035
Six months mortality, % (N)	13% (9)	28% (19)	0.031
Predicted six months mortality, mean % (SD)	30.7% (18.6)	33.4% (21.7)	0.682
Observed vs. expected ratio for mortality	0.42	0.84	
Six months unfavorable outcome, % (N)	34% (23)	57% (38)	0.015
Predicted six months unfav. outcome, mean % (SD)	51.8% (21.6)	53.9% (23.7)	0.626
Observed vs. expected ratio for unfav. outcome	0.66	1.06	

## P224 Early measurement of low cerebral blood flow is associated with brain hypoxemia after traumatic brain injury

### CS Sokoloff^1^, M Albert^1^, D Williamson^1^, C Odier^2^, J Giguère^1^, E Charbonney^1^, F Bernard^1^

#### ^1^Hôpital du Sacré-Coeur de Montréal, Montréal, Canada; ^2^Centre Hospitalier Universitaire de Montréal, Montreal, Canada

**Introduction:** Management of traumatic brain injury (TBI) can include monitoring of brain tissue oxygenation (PbtO2) to prevent secondary brain injury. A few studies suggest measuring blood flow velocity could predict cerebral hypoxia [1]. Cerebral oxygenation measured by PbtO2 depends on blood O2 content, cerebral blood flow (CBF) and diffusion. We aimed to study the relationship between flow velocity using trans-cranial Doppler (TCD) in patients with moderate to severe TBI and PbtO2 during cerebral hypoxic episodes.

**Methods.** Serial TCD studies were done to assess CBF velocity of the middle cerebral artery (MCA). Measurements were done bilaterally after the insertion of PbtO2 monitoring, on a daily basis for 5 days, and during auto-regulation, vaso-reactivity and hyperoxic challenge tests when feasible. Various parameters were collected simultaneously: PbtO2, PaO2, PaCO2, Hb level, ICP, CPP and cardiac index.

**Results.** A total of 85 TCD studies in 17 consecutive patients were obtained. We observed 29 episodes of cerebral hypoxia (PbtO2 ≤ 20 mmHg) distributed as follow: 10 episodes of PbtO2 ≤ 10 mmHg, 5 episodes of 10-15 mmHg, and 14 episodes of 16-20 mmHg. Overall, no correlation between PbtO2 and MCA’s mean blood flow velocity (Vmean) was found. For TCD studies obtained within 24 h from trauma (N = 14), there was a weak correlation between Vmean and PbtO2 (r2 = 0.41; p = 0.035) with median values of Vmean of 42.9 ± 38.4 cm/s and of PbtO2 of 14.95 ± 8.74 mmHg. Among these, all Vmean < 40 m/s (n = 6) were associated with a PbtO2 ≤ 20 mmHg. We did not find any association with other factors.

**Conclusions.** Low CBF velocity (<40 m/s) measured with TCD within the first 24 h of TBI is associated with brain tissue hypoxia measured with PbtO2. Interventions to optimize CBF and O2 content immediately after TBI could help minimize early secondary injuries.

**Reference**

[1] van Santbrink H. et al. Serial Transcranial Doppler Measurements in Traumatic Brain Injury with Special Focus on the Early Posttraumatic Period. Acta Neurochir 2002: 144: 1141–1149

## P225 The prognostic role of tractography in the management of traumatic brain injury

### Z Husti, T Kaptás, Z Fülep, Z Gaál, M Tusa

#### Bács- Kiskun County Hospital, Kecskemét, Hungary

**Introduction:** The management of patients with traumatic brain injury (TBI) is a great challenge for intensive care units. In the case of TBI patients without obvious space occupying lesion and with stable cardiorespiratory condition, available conventional imaging techniques (CT, MRI) may be insufficiently sensitive to predict long- term neurological outcome. Tractography (TG) is a special technique of MRI using diffusion weighted images (DWI) which enable to visualize neuronal networks formed by connections among cortical and subcortical regions. This method be able to early detect diffuse axonal lesion that may draw attention to poor outcome unlike conventional CT or MRI. The aim of our study was to determine the role of TG in the diagnosis of TBI without elevated ICP regarding to the prognosis.

**Methods:** Diffusion tensor imaging (DTI) TG was carried out by applying Philips Achieva 1,5 T, software 5.2.0. Tractographic images were constructed by Medlnria 2.2.3 and DSI studio 2.2016.11.02 software. TG was performed on four selected TBI patients characterized by GCS 3 with stable cardiorespiratory condition and sufficient oxygenation at praehospital period. Neither of them showed increased ICP confirmed by imaging techniques and intracranial pressure monitoring. Patients were followed- up regarding to the neurological outcome. The relationships between the severity of TG images and prognosis were investigated.

**Results:** DTI TG obtained from all examined patients showed radical destruction of the tracts in the white matter as seen on demonstrated pictures comparing with an anisotropic map from a healthy individual. Neurological results of patients showed as the following: The first patient remained unconsciousness in a stable vegetative condition. The second patient follows simple instructions, however, permanently aphasic. The third patient developed neurological improvement after 3 weeks from the injury but died during rehabilitation. In the fourth case where TG showed the most serious damage comparing with control recording, neurological improvement was not observed and the patient died after 10 days from the trauma. These results show that TG appropriately predicted a poor neurological outcome in patients with TBI despite obvious space occupying lesion.

**Conclusions:** TG may be a suitable imaging technique for predicting prognosis and can be a significant part of the decision making process during TBI management. However, further large case number of studies are needed to define the prognostic value of this method in the course of TBI management.

## P226 Optimal cerebral perfusion pressure; bedside application after severe traumatic brain injury

### J Donnelly^1^, M Aries^2^, M Czosnyka^1^, C Robba^3^, M Liu^1^, A Ercole^3^, D Menon^3^, P Hutchinson^4^, P Smielewski^1^

#### ^1^Brain Physics Laboratory, Division of Neurosurgery, Department of Clinical Neurosciences, Cambridge Biomedical Campus, University of Cambridge, Cambridge, United Kingdom; ^2^MUMC, Maastricht, Netherlands; ^3^Division of Anaesthesia, Department of Medicine, Addenbrooke’s Hospital, University of Cambridge, Cambridge, United Kingdom; ^4^Division of Neurosurgery, Department of Clinical Neurosciences, Addenbrooke’s Hospital, University of Cambridge, Cambridge, United Kingdom

**Introduction:** Previously, a cerebrovascular reactivity-based method that yields continuous estimates of optimal cerebral perfusion pressure (CPPopt) has been developed using a TBI cohort. Using recent CPPopt data from patients after severe TBI, we aimed to assess the real-time clinical value of CPPopt by testing the hypothesis that bedside CPPopt data without artifact removal remains an independent determinant of patient outcome.

**Fig. 16. (abstract P226).**
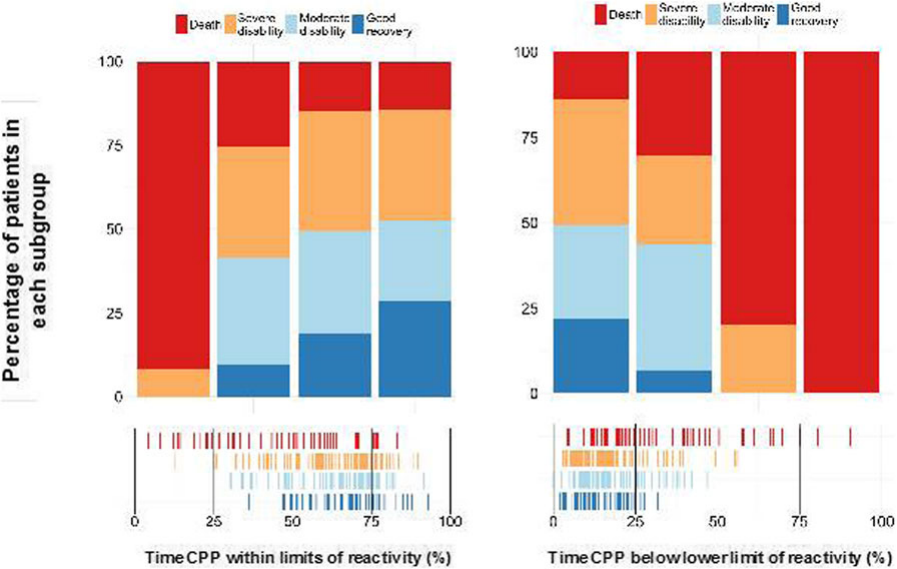
Time spent within (**a**) or below (**b**) CPP pressure reactivity limits

**Methods:** Single center cerebral monitoring data from severe TBI patients admitted between 2010 and 2015 were used. Treatment was guided by controlling absolute values of ICP and CPP. CPPopt was determined using a previously published curve-fitting protocol. No manual cleaning of signal artifacts was performed. The difference between current CPP and CPPopt, coupled with the current PRx value (with a threshold for impaired autoregulation (PRx) of +0.15), was used to determine whether each CPP was below the lower limit of reactivity (LLR), above the upper limit of reactivity (ULR) and within the reactivity limits. The time each patient spent below, above or within these reactivity limits was compared across GOS groups (Fig. 16).

**Results:** ICP monitoring data from 231 patients were available. Time spent with CPP within reactivity limits or the time spent with CPP below the LLR were both associated with GOS, independent of age, GCS motor score, pupillary reactivity and mean ICP (OR for %time CPP below LLR = 0.96 (0.94-0.98), OR for %time CPP within reactivity limits = 1.03 (1.01-1.05).

**Conclusions:** This observational report demonstrates that recent prospective bedside monitoring of CPPopt after severe TBI is both practical and retains independent prognostic significance.

## P227 Regional brain tissue oxygen tension normalized to arterial oxygen tension predicts outcome in traumatic brain injury

### R López, J Graf, JM Montes

#### Clínica Alemana de Santiago, Santiago, Chile

**Introduction:** Regional brain tissue oxygen tension (PbtO2) depends on regional perfusion, regional oxygen extraction and arterial oxygen tension (PaO2). If PbtO2 is to be used as a brain tissue oxygen extraction variable it should be normalized for PaO2. We explored the outcome prediction potential of normalized PbtO2 [PbtO2N = (PaO2-PbtO2)/PaO2] as compared to PbtO2, intracranial pressure (ICP) and cerebral perfusion pressure (CPP) in a cohort of traumatic brain injury (TBI) patients.

**Methods:** Retrospective analysis of 14 patients with severe TBI and a PbtO2 probe inserted on proximity at brain parenchymal lesion. Functional outcome was classified using the Glasgow Outcome Scale-Extended (GOSE). We considered > =5 points as favorable outcome (FO). Areas under the receiver operating characteristic curves (AUC) for outcome prediction were determined for the fraction the initial 24 h (%t) with high ICP, %t with CPP <60 mmHg, %t with PbtO2 < 15 mmHg and PbtO2N at 24 h.

**Fig. 17 (abstract P227).**
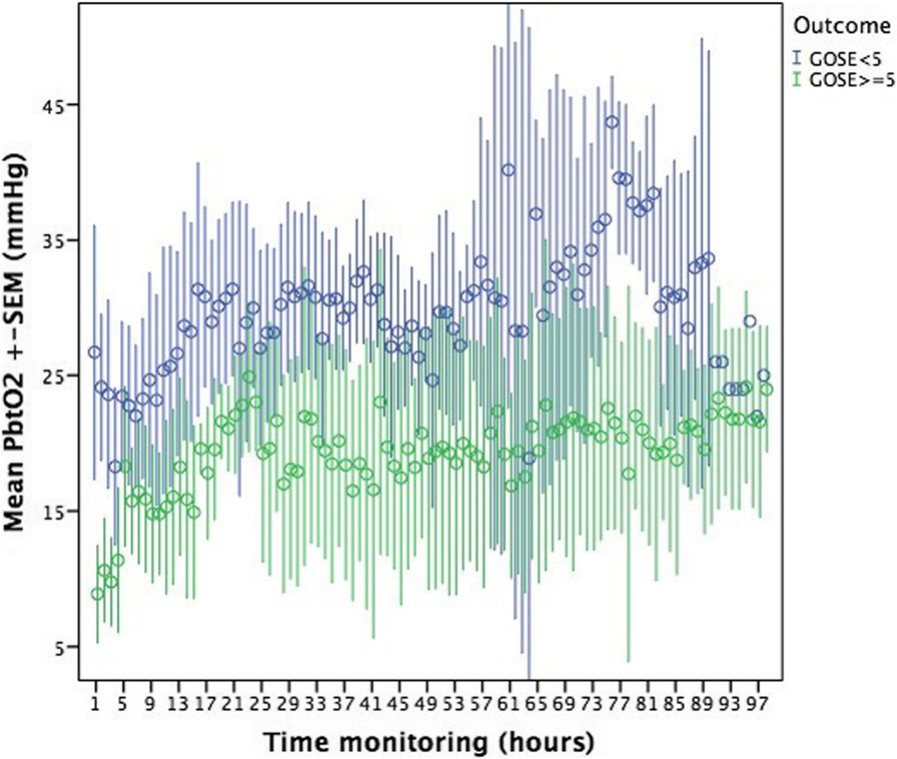
Time course of PbtO2 according to functional outcome

**Results:** Seven patients had FO. Table 12 shows demographic and physiological data and Fig. 17 shows time course of PbtO2 according to outcome. Patients with FO exhibited lower initial and subsequent rise in PbtO2 values. PbtO2N displays the best AUC (0.939, CI = 0.818-1, p = 0.006) among the variables tested for outcome prediction with values >0.80 associated to FO.

**Conclusions:** In agreement with previous reports [1], low initial PbtO2 value with gradual recovery is associated with FO. PbtO2 normalized for PaO2 at 24 h seems better than PbtO2, ICP or CPP for functional outcome prediction.

**Reference**

[1] Ponce LL, et al.: Neurosurg 2012; 70:1492–1503

**Table 12 (abstract P227).** Demographic and physiologic data according to functional outcome

	GOSE > =5	GOSE < 5	p value
Patients, N	7	7	
Male, N	4	4	n/s
Age, mean (SD)	35 (20)	59(21)	n/s
Decompressive Craniotomy, N	2	3	n/s
%t High ICP (SD)	10(20)	1(2)	n/s
%t CPP < 60 mmHg (SD)	5(7)	4(6)	n/s
%t PbtO2 < 15 mmHg (SD)	37(31)	6(14)	0.03
PbtO2N at 24 h (SD)	0.85 (0.07)	0.72(0.09)	0.01

**Fig. 18 (abstract P227).**
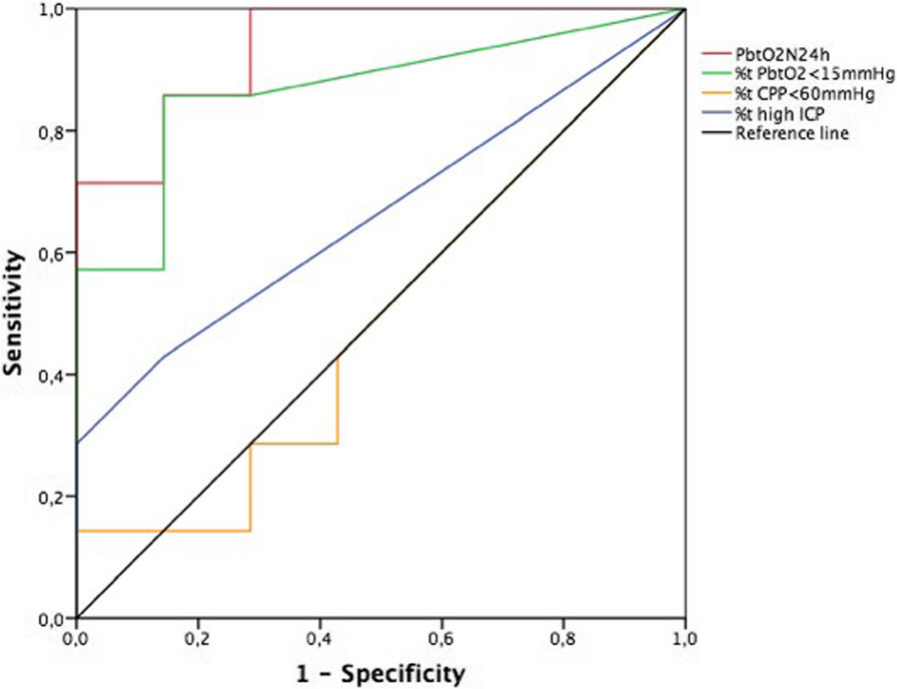
ROC curves for outcome prediction

## P228 Predictive value of common intensive care severity scores in traumatic brain injury

### M Kenawi^1^, A Kandil^2^, K Husein^1^, A Samir^1^

#### ^1^Cairo university hospital, Cairo, Egypt; ^2^Helal hospital, Cairo, Egypt

**Introduction:** Traumatic Brain Injury (TBI) causes a severe toll on society as a leading cause of mortality worldwide and the major cause of disability among young adults. The prognosis after TBI had been particularly challenging to predict, with limited availability of robust prognostic models. We aimed to evaluate the usefulness of the APACHE II (Acute Physiology and Chronic Health Evaluation II), SAPS II (Simplified Acute Physiology Score II) and SOFA (Sequential Organ Failure Assessment) scores compared to simpler models based on age and Glasgow Coma Scale (GCS) in predicting a six-month mortality of patients with moderate to severe traumatic brain injury (TBI) in the intensive care unit (ICU).

**Methods:** A Prospective cohort study conducted on acute TBI patients admitted to I.C.U at EL-HELAL trauma Centre and KASR AL AINI university hospital, Egypt during the period from August 2014 to April 2015. All patients were followed-up for 6 months from the day of admission. Our patients were divided into two groups (survivors and non-survivors).

**Results:** A total of 104 patients were enrolled. Mean age was 37 ± 17.16 years, the overall six-month mortality was 25 patients (24.4%). The univariate analysis showed that APACHE II, SAPS II, SOFA, GCS, and age had a significant statistical difference regarding mortality between both groups (P-value < 0.05) and the optimal cut-off point as mortality indicator was 14, 26, 4, 9 and 49 respectively with area under the curve (AUC) 0.88, 0.87, 0.83, 0.80 and 0.79 respectively. By Multivariate analysis using logistic regression, we found only Age and GCS had a statistically significant impact on outcome (P-value; 0.001, 0.022).

**Conclusions:** A simple prognostic model based only on GCS and Age displayed good predictor for six-month mortality of ICU treated patients with TBI. The use of the more complex scoring systems (APACHE II, SAPS II and SOFA) added little to the prognostic performance.

## P229 Development of a process indicator-based plan-do-act-check cycle to improve quality of care in severe traumatic brain injury patients

### J Heijneman, J Huijben, F Abid-Ali, M Stolk, J Van Bommel, H Lingsma, M Van der Jagt

#### Erasmus Medical Center, Rotterdam, Netherlands

**Introduction:** Treatment of traumatic brain injury (TBI) patients aims at secondary brain injury prevention. Intracranial pressure (ICP) is a crucial parameter. Process indicators, which refer to appropriateness of delivered care, can be used to monitor protocol adherence. However, use of quality indicators for TBI and ICP has been scarce. We developed and applied an ICP process indicator and additionally constructed a Plan-Do-Check-Act (PDCA) cycle describing key learning points to improve quality of care.

**Fig. 19 (abstract P229).**
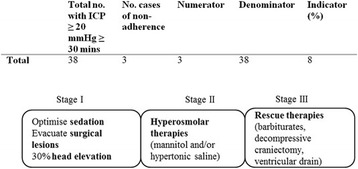
ICP Indicator metrics and ICP management escalation stages I-III

**Methods:** The study took place at an academic neurotrauma center ICU. We used focus group interviews to reach consensus on a practical ICP indicator. The indicator was applied to adult TBI patients receiving ICP monitoring (Apr.-Sept. 2016). Patients with infaust prognosis were excluded. Data on ICP levels and applied treatments were collected. Protocol non-adherence (ICP > 20 mmHg, > = 30 minutes without appropriate escalation of treatment according to protocol (Fig. 19) was first assessed by a research nurse and subsequently interpreted by an ICU-fellow and neurointensivist. Details on non-adherence were assessed and incorporated in a PDCA-cycle construct.

**Fig. 20 (abstract P229).**
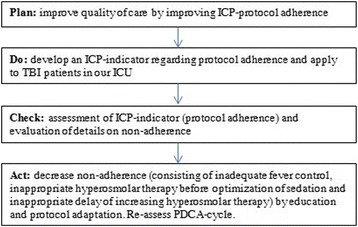
PDCA-cycle

**Results:** We analysed 43 patients of whom 5 had an infaust prognosis, resulting in 38 included patients. Protocol adherence was inadequate in 3 cases (8%) (Fig. 19). In 1 patient there was an adequate intervention but not within the set time, while in 2 patients therapy was inadequate (failure to control fever, inadequate osmotic therapy). With these data we constructed a PDCA-cycle (Fig. 20).

**Conclusions:** We showed feasibility of an ICP process indicator for protocol adherence to construct a simple PDCA-cycle. Apart from repeating the cycle, striving for 100% adherence, future steps may include protocol adaptation, education and assessment of a possible association between ICP-indicator metrics and patient outcomes.

## P230 Skin cover plasty after decompression craniectomy, as a method of solving intracranial hypertension – case history

### RC Cihlar

#### Nemocnice Ceske Budejovice, C eske Budejovice, Czech Republic

**Introduction:** A possibility to solve intracranial hypertension by means of a skin cover plasty in patient after decompression craniectomy, which is followed by a further increase in ICP.

**Methods:** The case history of a man, aged 29, suffering from a severe TBI. ICP was monitored by the parenchymatous sensor inserted into the left frontal lobe in the usual manner. After conservative procedures of intracranial hypertension treatment had been exhausted, a broad decompression craniectomy was conducted on the right, which led to a decrease in ICP. During 48 hours, a repeated increase in ICP occurred in the patient, with signs of intracranial hypertension in a CT examination. After considering all possibilities, the decompression craniectomy was further enlarged by dissolving the skin suture and conducting a plastic surgery of the damage caused, by means of the combined COM 30 bandaging fabric. This is a temporary fabric cover that substitutes the skin and is used especially in the treatment of burns, as a cover of skin transplants.

**Results:** This procedure led to a decrease in ICP and the disappearance of CT signs of intracranial hypertension in the patient. No infectious, bleeding or necrotic complications were observed. After the remission of the cerebral oedema, COM 30 was removed after 14 days, and a suture of the skin cover was conducted above the decompression craniectomy. The patient left hospital with a satisfactory neurological finding, with GOS 5.

**Conclusions:** The use of the artificial skin cover in the patient with decompression craniectomy resulted in a distinct, lasting decrease in intracranial hypertension below the value of 20 mmHg. This method may be recommended as an extreme procedure to solve intracranial hypertension in patients with decompression craniectomy and a continuing increase in ICP. Informed consent to published has been obtained from the patient.

**Fig. 21 (abstract P230).**
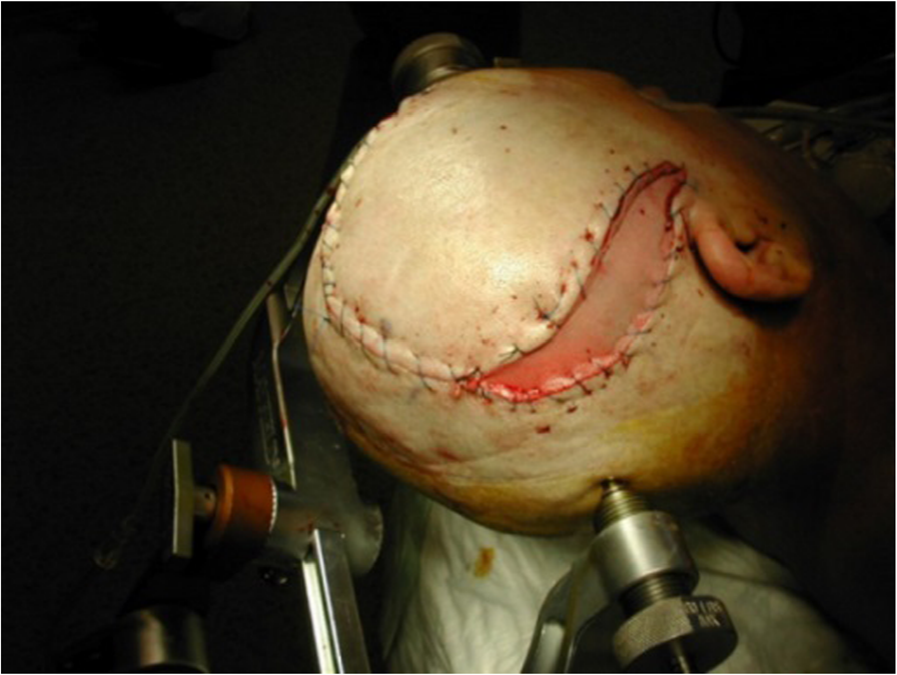
Photographic documentation from the operating theatre

## P231

### Withdrawn

## P232 Haemorrhagic shock in war wounded: mortality at a surgical center for war victims in Afghanistan

### G Mancino, P Bertini, F Forfori, F Guarracino

#### Azienda Ospedaliero Universitaria Pisana, Anaesthesia And Intensive Care Department, Pisa, Italy

**Introduction:** A small percentage of war wounded patients is hypotensive on arrival, requiring aggressive fluid resuscitation before surgery. This population has been described, in order to identify factors related with morbidity and mortality.

**Methods:** Patients who presented to the ER with hemorrhagic shock and a SBP lower than 90 mmHg were enrolled. Data were collected with regards to timing and mechanism of trauma, number of organs involved, the type and amount of fluid used for the resuscitation, the number of units of whole blood transfused, the surgery performed, the body temperature and blood gas data in the immediate postoperative time. The development of complications, the duration of mechanical ventilation, the use of α dose dopamine, the mean length of stay in ICU and the in-hospital mortality were collected as well. Injury severity score (ISS) and new injury severity score (NISS) were calculated retrospectively.

**Results:** 60 patients with a mean ISS of 17.88 ± 5.76 were enrolled. 38.3% of the patients developed complications during the stay, and the overall in hospital mortality was 26.6% (n = 16). Patients who died were older than survivors, had higher ISS and NISS (respectively 23.50 ± 1.19 and 26.38 ± 9.65) and had received a preoperative infusion of colloids. Mortality rate was higher for those who underwent damage control surgery and who received a greater amount of ringer lactate for resuscitation (2.85 ± 0.45 liters vs 1.98 ± 0.16 liters, p = 0.0241). In the postoperative period all the variables taken into consideration showed a statistically significant correlation with mortality: survivors had higher values of body temperature, pH and hemoglobin and lower levels of lactates and base deficit. The transfusion of a higher number of units of whole blood, the infusion of dopamine and the development of complications had a strong correlation with mortality.

**Conclusions:** Given a relatively low mortality rate, patients with a worse outcome were those who suffered from hypothermia, acidosis and coagulopathy. The study showed that a higher amount of fluids and of units of blood transfused was associated with a higher mortality rate, maybe through its hemodiluition effect and the consequent development of coagulopathy. The data collected confirmed the association between mortality and ISS, NISS, amount of cristalloids and of blood units used for resuscitation. These results encourage the use of damage control resuscitation in selected trauma patients, in order to promptly control the hemorrhage and reduce the amount of fluid administered to patients suffering from hemorrhagic shock.

## P233 Late versus early surgical fixation of femoral bone fracture could increase the neurocognitive dysfunction and postoperative morbidity of the eldery patients

### D Pavelescu, I Grintescu, L Mirea

#### Emergency Hospital Floreasca, Bucharest, Romania

**Introduction:** Neurocognitive dysfunction is a particular problem in elders with 30-80% becoming delirious after major surgery and 30-40% developing early cognitive dysfuntion (POCD).

The cognitive morbidity is important, delirium and POCD were associated with longer hospital stay and costs, with higher morbidity and mortality.

**Methods:** After written informed consent and approval by institutional review board, 112 patients, 65-101 years old admitted in Emergency Hospital with traumatic femoral bone fracture, were enrolled in an prospective, observational study. Surgical fixation was made early in 68 patients (allocated in group A) and was delayed for at least 48 hours in the other 52 patients (allocated in group B), for different reasons (chronic anticoagulation, comorbidities). Surgical procedure for all patients was done under spinal anesthesia. All patients were evaluated at admission and on day 7 from admission. We asssess the incidence of pre and postoperative neurocognitive dysfunction using Informant Questionnaire on Cognitive Decline in the elderly, which range from 1 to 5, supplemented with MMSE. A score of more or equql of 3,38 indicates neurocognitive dysfunction. We also evaluate the presence of aquired neuromuscular weakness on day &, using Medical Research Council Score (<48), the incidence of postoperative complications (pulmonary Thromboembolism, pneumonia) and mortality. Statistical analysis was made using SPSS tools, unpaired t-test and Mann-Whytney U test, a p-value < 0,005 was considered statistically significant.

**Results:** No significant difference concerning neurocognitice dysfunction prior surgery between the two groups (10,8% vs 9, 2%). here is a significant higher incidence in postoperative neurocognitive dysfunction in group B (14,7% vs 66,6%), also a higher incidence of thromboembolism and pneumonia in group B. Aquired neuro-muscular weaness appear early in the first week and there is a strong correlation with late surgical intervention and delayed mobilisation. The mortality is higher in group B (5,55% vs 1,49%, p-value < 0,005).

**Conclusions:** Neurocognitive dysfunction is an important complication that could induce the development of aquired neuro-muscular weakness by late mobilisation, could impede the recovery and dramatically increase the postoperative morbidity and mortality.

Early surgical fixation of femoral bone fracture could improve the clinical and neurological status of the elderly patients and increase the quality of life.

## P234 The effictiveness of prophylactic inferior vena cava filters insertion in trauma patients

### S Alamri^1^, M Tharwat^1^

#### ^1^Riyadh National Hospital, Riyadh, Saudi Arabia

**Introduction:** Trauma patients are at high risk of developing venous thromboembolism (VTE) including deep venous thrombosis and pulmonary embolism (PE). The epidemiology of VTE in trauma patients showed that PE is the third major cause of death after trauma in patients who survive longer than 24 hours after onset of injury [1]. Besides, patients recovering from trauma have the highest rate of VTE among all subgroups of hospitalized patients. PE following development of DVT is one of the most preventable causes of death in hospitalized patients [2].

IVC filters have been suggested in some studies to decrease the risk of PE in various patient populations including the critically ill and trauma patients [2].

**Methods:** 32 trauma patients were admitted to ICU at National Care hospital in Riyadh,KSA with pelvic or femur fractures in the period from 4/2015 to 10/2016, all candidate patients were started on prophylactic anticoagulation with enoxaparin, were followed during their ICU stay and up to the hospital discharge, for development of pulmonary embolism and to compare those who had IVC filter inserted and those who didn’t have IVC filter, in relation to the development of pulmonary embolism events

**Results:** 32 adult trauma patients were enrolled in the study. 5 patients had no IVC filter inserted(15.6%). 2 patients of the no IVC filter group had PE (40%) (p = 0.000689). 27 patients had IVC filter(84.4%), none of them had positive PE. 2 patients died in the study but only one died of major PE and this patient had anticoagulation started but with no IVC filter inserted.

**Conclusions:** Trauma patients with lower limb long bone or pelvic fractures are at high risk of thromboembolic events including major PEs. Early insertion of IVC filter along with prophylactic anticoagulation prevented the development of PE. These results need to be confirmed in large scale randomized trial.

**References**

1. Sachdeva A, Dalton M, Amaragiri SV, Lees T. Elastic compression stockings for prevention of deep vein thrombosis. Cochrane Database Syst Rev. 2010 Jul 7;(7)

2. Torbicki A, Perrier A, Konstantinides S, Agnelli G, Galiè N, Pruszczyk P, et al. Guidelines on the diagnosis and management of acute pulmonary embolism: The Task Force for the Diagnosis and Management of Acute Pulmonary Embolism of the European Society of Cardiology (ESC) Eur Heart J. 2008;29:2276–315.

**Fig. 22 (abstract P234).**
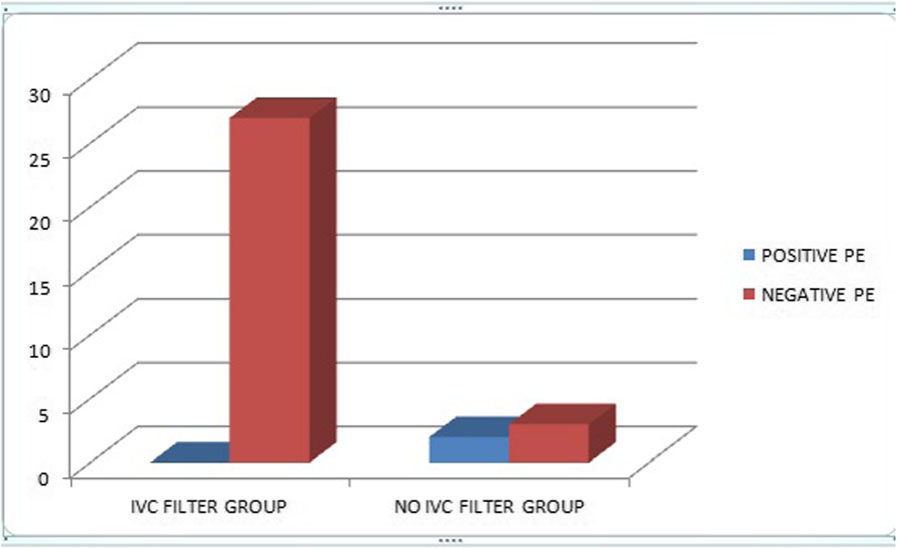
See text for description

## P235 Complications following resuscitative endovascular balloon occlusion of the aorta in patients with polytrauma

### N Kono, H Okamoto, H Uchino, T Ikegami, T Fukuoka

#### Kurashiki Central Hospital, Kurashiki City, Japan

**Introduction:** Resuscitative endovascular balloon occlusion of the aorta (REBOA) is a temporary bleeding control method for patients who experience hemorrhagic shock. Since it is less invasive than resuscitative thoracotomy, REBOA is being increasingly used for polytrauma patients. However, despite the increased use of this procedure, its safety is yet to be thoroughly evaluated. The aim of our study is to review the complications we experienced related to REBOA in our emergency department.

**Methods:** This study is a retrospective cohort study conducted at a tertiary referral hospital in Japan from October 2014 to September 2016. All trauma patients who required REBOA for the control of bleeding were included. All procedures were performed by an emergency physician and any other combined treatments were managed at the discretion of the attending trauma surgeon. The primary outcome was complications related to REBOA. We collected data from our electronic medical records, including age, sex, site of injury, mechanism of injury, injury severity score (ISS), complications, and outcomes. We described these data as number (%) and median (interquartile range).

**Results:** Ten patients were included. The median age was 45 (24-83), and 6 patients (60%) were male. The mechanisms of injury were motor vehicle accident 8 (80%), fall 1 (10%), and entrapment by heavy machinery 1 (10%). The sites of injury were pelvic fractures 9 (90%), lower extremity fractures 7 (70%), and traumatic brain injuries 6 (60%). The median ISS was 46 (41-59), and 9 patients (90%) achieved systolic blood pressure > 90 mmHg after REBOA. Complications related to REBOA were observed in 3 patients (30%). Of these, 2 patients experienced a deterioration of traumatic intracranial hemorrhage, and the remaining patient experienced an intraoperative device problem (ruptured balloon during the procedure).

**Conclusions:** Our study revealed 3 REBOA related complications. Although REBOA may be less invasive, is potentially beneficial, and now it is widely indicated to control hemorrhage, our results suggest that the physician must pay more attention when undertaking REBOA due to its potential complications and safety issues.

## P236 Evaluation of trauma patients admitted to ICU with trauma and injury severity score (TRISS)

### M Simoes, E Trigo, P Coutinho, J Pimentel

#### Centro Hospitalar e Universitario de Coimbra, Coimbra, Portugal

**Introduction:** Trauma is one of the leading causes of mortality and morbidity in ICU. Injury severity and prognosis assessing is a complex process. The Trauma and Injury Severity Score (TRISS), introduced in 1981, was developed combining patient age with an anatomical score, the Injury Severity Score (ISS), and a physiological score, the Revised Trauma Score (RTS). Despite some limitations, it still remains the most used scoring system in trauma patients.

**Methods:** Retrospective analysis of clinical records of all adult severe trauma patients admitted to a trauma center in a university hospital’s ICU in one year (2015). Gender, age, mechanism of injury, type of injury, length of ICU and hospital stay and mortality were studied. RTS, ISS and TRISS scores (using 1995 and 2009 revisions) were determined for every patient based on clinical records. Statistical analysis of TRISS’s performance as predictor for survival.

**Results:** In 2015 were admitted to our ICU 124 adult trauma patients, the majority were male (83.1%). Age ranged from 18-91 years, with mean of 52.4 years. Most patients were admitted directly from Emergency Room (82.3%), 40.3% of all the patients came from other hospitals. Regarding mechanism of injury, road traffic collisions were responsible for 46.0% of cases, followed by falls (39.5%). Road traffic collision patients were younger than those with injuries caused by falls (45.72 years and 62,14 years, respectively). Trauma brain injury was the most frequent type of lesion admitted to ICU (67.7%), followed by thoracic injury (45.2%). The average length of ICU stay was 13.8 days. The average hospital length of stay was 32.6 days, although this value was probably higher, as 56 patients were transferred back to smaller hospitals. The hospital mortality rate was 26.6% (25 patients died at the ICU and 8 died after ICU discharge), but no information regarding the outcome of transferred patients is known to the authors, therefore the mortality ratio might have been higher. Mortality ratio was higher in older patients (47.2% in patients aged 65 and over and 18,2% in patients under 65 years). Excluding those transferred patients, TRISS showed a statistical difference between the survivors and non survivors (87.7% vs 78.0%, p = 0.0249).

**Conclusions:** TRISS was a valuable tool for assessing the clinical outcome, as significant difference was found between survivors and deceased patients. Injury evaluation and outcome prediction is relevant issue in critical care trauma patients.

## P237 Management of major bleeding trauma in ICU: preliminary data on the use of thromboelastometry in a tertiary care hospital

### A Franci, D Basagni, M Boddi, M Cozzolino, V Anichini, A Cecchi, A Peris

#### Careggi Teaching Hospital, Florence, Italy

**Introduction:** Diffusion of viscoelastic methods, combined with the availability of coagulation factors’ concentrates, allowed the introduction of goal-directed treatment’s protocols (GDT), aimed at the correction of specific haemostatic alterations.

The aim of the study was to evaluate if thromboelastometry could determine a better outcome and a reduction in blood products’ consumption and in treatment’s costs in bleeding trauma patients.

**Methods:** We conducted an observational retrospective study, including patients with an Injury Severity Score (ISS) > =15 and transfused with at least 3 units of Red Blood Cells within the first 24 H. The patients have been divided into 2 groups, according to the hemostatic treatment received: Group A (GDT) and Group B (conventional therapy).

**Results:** The 2 groups didn’t shown differences in outcome, though the use of blood products and coagulation factors’ concentrates was significantly greater in Group A. This might depend on the trend of using thromboelastometry in the most critical bleedings: Group A had on average a greater ISS and a lower mean arterial pressure and received a more aggressive fluid therapy in prehospital setting. We can also hypothesize a tendency to adopt a traditional approach for the treatment of these patients (high consumption of plasma and low of concentrates). However, it may partially depend on the earlier detection of the coagulopathy by the thromboelastometry.

**Conclusions:** Introduction of thromboelastometry wasn’t associated with an improvement in outcome and a reduction in blood products’ consumption and costs. Therefore, we decided to broadcast staff training programs and to implement goal-directed treatment’s protocols for bleeding trauma.

**Table 13 (abstract P237).** See text for description

Mean ± SD	Group A	Group B	p
RBC total (U)	12.79 ± 7.77	6.58 ± 2.67	p = .0028
FFP total (U)	10.36 ± 6.38	5.00 ± 4.27	p = .0069
PLT total (U)	7.50 ± 7.55	2.95 ± 3.36	p = .0258
Fibrinogen total (g)	4.29 ± 3.02	1.63 ± 1.46	p = .0022
PCC total (ml)	2000.00 ± 1951.33	500.00 ± 816.50	p = .0050

## P238 How inflammatory markers are correlated with the onset of fever in ICU trauma patients; preliminary results

### D Markopoulou, K Venetsanou, I Papanikolaou, T Barkouri, D Chroni, I Alamanos

#### Kat Hospital Athens, Kifisia, Greece

**Introduction:** The acute multiple trauma state is often followed fever. It’s known that traumatic injuries stimulate an inflammatory reaction and cytokines cascade. The aim of this study is to investigate the correlation of fever with inflammatory markers and circulating endotoxin, in patients admitted in ICU.

**Methods:** Eighteen trauma patients were admitted in ICU and 18 healthy volunteers enrolled in this study as control group; 20 ml of peripheral blood was sampled from the trauma patients (10 ml during admission and 10 ml during the onset of fever and 10 ml from the control group. Clinical and demographic data were recorded on admission. Serum/plasma samples were isolated with centrifugation and stored at -70 ° C Interleukin-6 (IL-6),Lipopolysacharide binding protein (LBP), Procalcitonin (PCT), C-reactive protein (CRP) measured with ELISA and endotoxin with chromatometric assay.

**Results:** On admission, IL-6 (P < 0.001), CRP (P < 0.001) and PCT (P < 0.01) release, were significantly higher compared to control group, while LBP and endotoxin had no significant difference. The onset of fever was accompanied by abundant LBP release (P < 0.001), parallel reduction of circulating endotoxin (P < 0.01) and further significant increase of PCT and CRP (P < 0.05), compared to admission values. No significant difference found for IL-6 (P > 0.05).

The onset of fever was positively correlated with LBP (P < 0.001, Spearman coefficient (Spc) 0,866) and PCT (P < 0.05, Spc 0.281) and negatively with LAL (P < 0.01, Spc -0.436), but no significant results were found for IL-6.

**Conclusions:** LBP, the specific protein binding for circulating endotoxin, is more reliable and patent marker than PCT, CRP and IL-6, as far as fever concerned trauma patients.

**References**

1. Saxena, Manoj, et al. " Intensive care medicine 41.5 (2015): 823–832.

2. Markopoulou D et al. Int Care Med Exp 2016; 4 SUPPL: A306.

## P239 The ECS algorithm application in trauma patients: a pre-post analysis

### E Cingolani^1^, MG Bocci^2^, L Pisapia^2^, A Tersali^2^, SL Cutuli^2^, V Fiore^3^, A Palma^1^, G Nardi^3^, M Antonelli^2^

#### ^1^Azienda Ospedaliera San Camillo Forlanini, Roma, Italy; ^2^Fondazione A. Gemelli, Rome, Italy,^3^Infermi, Rimini, Italy

**Fig. 23 (abstract P239).**
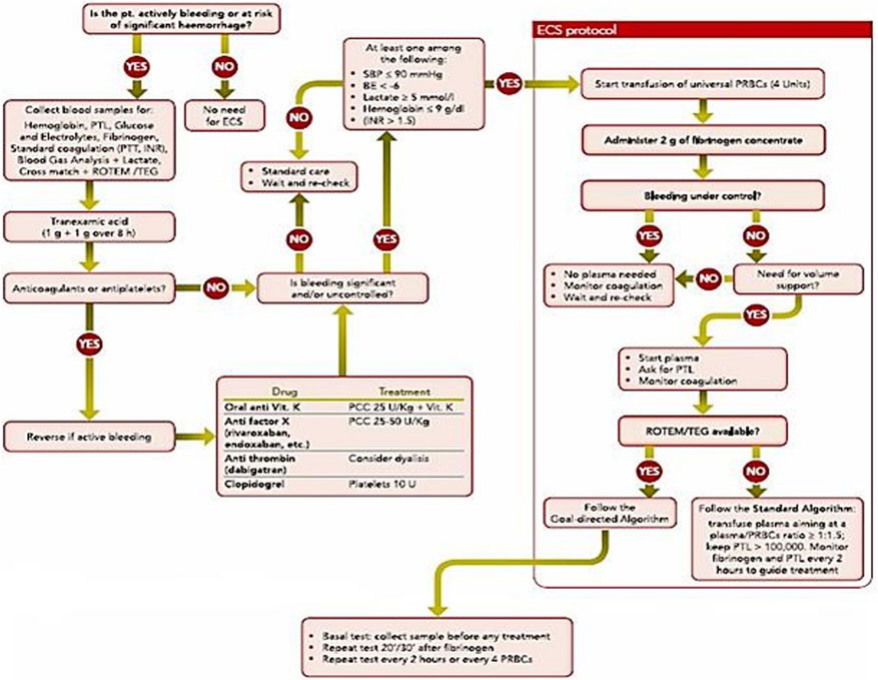
See text for description

**Introduction:** This study, performed by Policlinico Gemelli, aims to evaluate blood components consumption, mortality and morbidity associated with trauma, hemorrhage and blood transfusion pre and post the introduction of the Early Coagulation Support (ECS)[1]. The ECS is an algorithm developed by the Trauma Centers Network (TUN) (Fig. 23), aiming to improve and homogenize the treatment of trauma patients with significant bleeding and at high risk of massive transfusion.

**Methods:** ECS algorithm was applied to all severely injured patients with a ISS > 15, admitted to the hospital for trauma occurred within six hours, excluding patients who suffered of cardiac arrest prior to admission. A Propensity Score Analysis (PSA) was performed. A prospective Study Period (SP) started from 01 Jan. 2014 to 31 Dec. 2014 and a retrospective Control Period (CP) lasted from 01 Jan 2012 to 31 Dec. 2012.

**Fig. 24 (abstract P239).**
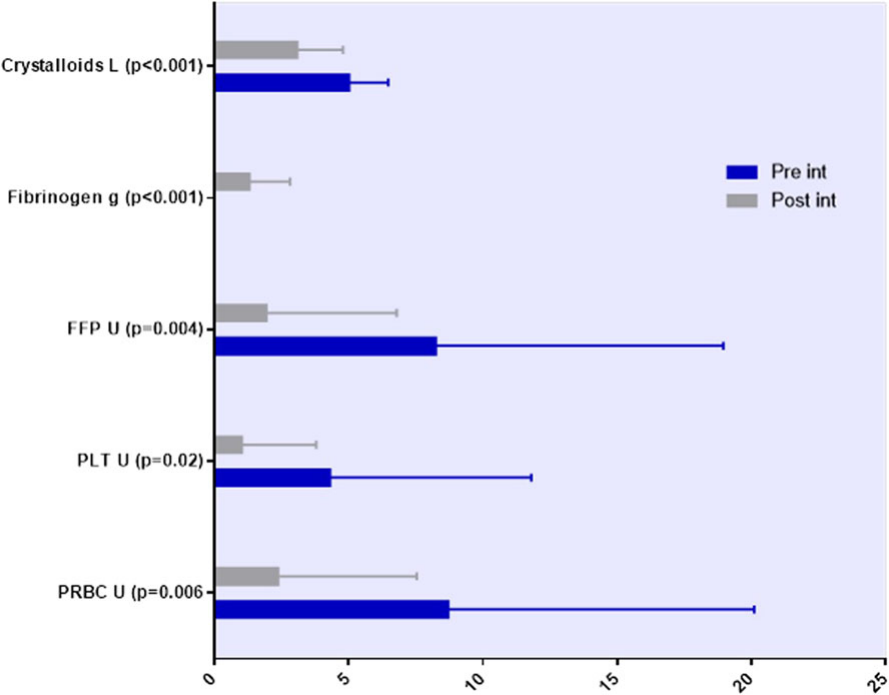
See text for description

**Results:** The PSA was performed on 64 patients, 32 in each group (CP and SP), matched on the estimated probability to receive a massive transfusion. Variables considered for PSA were: age (47 ± 20 vs 51 ± 19), SBP (91 mmHg in both), ISS (41[IQR 29-66] vs 41[IQR 27-57]), pH (7.28 ± 0.1 vs 7.25 ± 0.2), lactates (4.6 ± 2.9 vs 5 ± 2.9 mmol/l), platelets (196 ± 101 vs 200 ± 94 109/l), hemoglobin (10.7 ± 2.5 vs 11.4 ± 2.4 g/dl), fibrinogen (169 ± 78 vs 196 ± 59 mg/dl). Statistically significant reductions were observed in the SP group for blood components consumption, volume of infused crystalloids (Fig. 24). Furthermore, there was a significant reduction of surgical procedures performed in the first 24 hours of hospitalization in the SP but no relevant differences were observed neither for damage control surgery nor for each single type of trauma surgery. The 30-days survival analysis showed no difference between the two groups. It was observed a decrease of morbidity in SP group, although it was not statistically significant.

**Conclusions:** The ECS must be considered as a part of a comprehensive Damage Control Resuscitation. This algorithm allows to avoid plasma use in patients who need PRBCs massive transfusions, reducing related complications. Furthermore, the early restoration of fibrinogen blood concentration improves coagulation support. This study shows the saving in numbers of blood products and the carrying out of pertinent therapeutic strategies without an increasing of morbidity and mortality.

**Reference**

1. Nardi G, Agostini V, Rondinelli BM, et al. Prevention and treatment of trauma induced coagulopathy (TIC). An intended protocol from the Italian trauma update research group. J Anesthesiol Clin Sci. 2013;2(1):22.

## P240 Cell free DNA and protein C in trauma patients: an observational study

### R Coke^1^, A Kwong^1^, DJ Dwivedi^2^, M Xu^1^, E McDonald^1^, JC Marshall^3^, AE Fox-Robichaud^4^, E Charbonney^5^, PC Liaw^2^

#### ^1^McMaster University, Hamilton, Canada; ^2^TaARI, DBRI, Hamilton, Canada; ^3^St. Michael´s Hospital, Toronto, Canada; ^4^Hamilton Health Sciences, Hamilton, Canada,^5^Hopital du Sacre-Coeur, Montreal, Canada

**Introduction:** In trauma patients, tissue necrosis and cell death can result from direct injury, and/or inflammatory reactions. Cell free DNA (cfDNA), a potent procoagulant mediator, is released by activated neutrophils and other cells in response to injury or infection. Previous studies have shown that cfDNA levels are elevated in trauma patients, and correlate with injury severity, organ dysfunction, and survival. In addition, plasma levels of protein C (PC), an anticoagulant that inhibits clotting in the microcirculation, is decreased in trauma patients. The objective of this study was to describe a) the time dependent changes in cfDNA and PC in a cohort of trauma patients and, b) determine the correlation of these markers with organ dysfunction and 28 day mortality.

**Methods:** Plasma samples were obtained from 2 separate cohorts: a cohort of trauma patients with an episode of shock admitted to ICU as part of the DYNAMICS study [NCT0135504], and a single centre observational study (ENPOLY) of trauma patients admitted to ICU. Serial blood samples and clinical data were collected. In addition to baseline demographics, the variables analyzed included MODS components, lactate, as well as plasma levels of cfDNA (ug/ml) and PC (% of normal). This study was approved by the respective Research Ethics Boards of all participating centres. Data is reported as median and 25th and 75th IQR due to skewing with a Kruskal-Wallis test to assess for significance.

**Results:** 88 patients were included (49 DYNAMICS, 39 ENPOLY). Median age was 48.8y, 12.5% were female and 57% sustained head injury. Relative to healthy controls, baseline levels of cfDNA were higher, and PC levels lower. In non-survivors (n = 16) PC levels were significantly lower than survivors through the first week of ICU stay with the lowest levels seen at day 2 [(57% (43, 84) compared to 84% (66,103) p = 0.026]. In addition, platelet counts were significantly lower in non-survivors at ICU admission, as well as at and beyond day 4. Lactate levels were also significantly elevated during the first ICU week in non-survivors. There was correlation between daily organ dysfunction score (MODS) and PC. cfDNA levels did not discriminate between survivors and non-survivors in this study (p = 0.90).

**Conclusions:** Our findings suggest that coagulopathy (as reflected by decreased PC and platelet levels) and elevations in lactate are predictors of organ dysfunction and poor outcome in trauma patients. Unlike our previous findings in septic patients, this study suggests that cfDNA, while elevated in trauma, does not contribute significantly to the procoagulant pathophysiology.

## P241 The impact of APRV/BIPAP on the outcome of polytrauma patients with multiply organ failure syndrome

### I Kuchynska, IR Malysh, LV Zgrzheblovska

#### Shupyk National Medical Academy, Kiev, Ukraine

**Introduction:** Mortality after polytrauma in Ukraine varies very widely: from 10% to 60%, which is 7-10 times higher than in developed countries (I Shlapak, 2008). Polytrauma outcomes depend upon the development of multiply organ failure syndrome (MOF) in post injury period.

**Methods:** The 106 patients: 85 (80,1%) – male, and 21 (19.8%) - female. 100% patients had acute respiratory failure (ALI) caused by multiple rib fractures, lung contusion, aspiration of gastric contents and blood. Their age ranged from 18 to 60 with the mean of 32 years. Inclusion criteria: MV (>72 hours), ISS - 25-35 points; GCS >5 points on admission. Exclusion criteria: irreversible traumatic shock with CPR in the prehospital phase, comorbidities (COPD, etc).

Patients were divided in 2 groups. Start mode of MV in both groups - ventilation controlled by pressure (PCV). In control group (n = 51) respiratory support continued with pressure synchronized intermittent mandatory ventilation (PSIMV). In main group (n = 55) - with biphasic modes (BIPAP/APRV). Transition to spontaneous breathing was conducted by CPAP. In both groups, we used the same sedation protocols.

**Fig. 25 (abstract P241).**
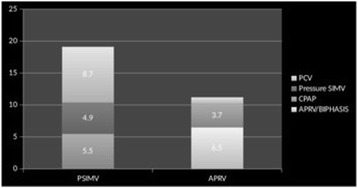
Comparison of the duration of MV

**Results:** The APRV group had: less duration of sedation - 6.2 ± 2.7 days vs PCV-PSIMV 10.7 ± 4.1 d (<0,05), shorter ventilations days-11,2 ± 3,1 vs 18.1 ± 2.3; (<0,05) (Fig. 25), reduced the ICU time (17 vs 24.6). (Table 14).

**Conclusions:** In Ukraine, the majority of patients with polytrauma has MOF and requires prolonged stay in the ICU. Biphasic modes allowed us to shorten the duration of MV and stay in the ICU.

**Table 14 (abstract P241).** Length of stay in the ICU, days

	APRV X (min-max)	PCV- PSIMV X (min-max)	P (APRV- PCV- PSIMV)
In ICU	17.0 (4-32)	24.6 (10-86)	0.002

## P242 A descriptive analysis of a national survey about the use of prophylac-tic low molecular weight heparin in the ED

### L Mestdagh^1^, EF Verhoeven^2^, I Hubloue^1^

#### ^1^Uzbrussel, Brussel, Belgium; ^2^Evert Verhoeven, Vorselaar, Belgium

**Introduction:** Low molecular weight heparins (LMWH) are commonly prescribed in the emergency department (ED). Not prescribing LMWH for patients with lower limb immobilization (LLI) and at risk for venous thromboembolic events (VTE) can be life-threatening. The actual incidence of VTE in patients with LLI is estimated between 5 - 39%. In an exten-sive literature search we were not able to find ED-specific (inter)national guidelines regarding this topic. This prompted us to conduct a multi-center survey in Belgium regarding the use of LMWH in patients with LLI.

**Methods:** A questionnaire was developed and, after approval of the Ethical Committee, made available online. Participants were asked about the existence of formal guidelines within their ED, their prescribing behavior and the level of evidence for their behavior.

**Results:** 100 questionnaires were filled out. Information of 46 hospitals was collected.

When asked about guidelines 40% of the respondents confirmed having guidelines within their ED, evenly distributed for university and non-university hospitals. The prescribing behavior was based on experience (73%), literature (77%), local guidelines (46%) and eminence (68%).

The responding physicians had different back-grounds. Of the respondents 41% would always prescribe LMWH in patients with LLI. This would vary among specialties: 68.2% of the surgeons, 24.6% of the emergency physicians, 16.7% of the internal medicine physicians and 77.8% of the anesthesiologists.

**Conclusions:** We conclude that a lot of Belgian respondents aren’t familiar with the use of prophylactic LMWH in the ED.

The lack of clear guidelines might contribute to patients not getting the correct VTE prophylaxis. Also, the prescribing behavior is mostly based on personal experience and case reports instead of international guidelines based research. The difference between different specialties was expected, but the low prescribing rate for the emergency physicians identifies a clear point of focus for education and training and the need for specialty based guidelines.

## P243 Effect of age of transfused red blood cells on neurological outcome in critically ill patients with traumatic brain injury (able-TBI study)

### J Ruel-laliberte^1^, R Zarychanski^2^, F Lauzier^1^, P Lessard Bonaventure^1^, R Green^3^, D Griesdale^4^, R Fowler^5^, A Kramer^6^, D Zygun^7^, T Walsh^8^, S Stanworth^9^, C Léger^1^, A F. Turgeon^1^

#### ^1^Université Laval, Québec, Canada; ^2^University of Manitoba, Manitoba, Canada; ^3^Dalhousie University, Halifax, Canada; ^4^University of British Columbia, Vancouver, Canada; ^5^University of Toronto, Toronto, Canada; ^6^University of Calgary, Calgary, Canada; ^7^University of Alberta, Edmonton, Canada; ^8^University of Edinburgh, Edinburgh, United Kingdom; ^9^University of Oxford, Oxford, United Kingdom

**Introduction:** Anemia is frequent in critically ill patients with traumatic brain (TBI), often leading to red blood cells (RBC) transfusions. RBC can be stored up to 42 days, but prolonged storage may cause a decreased ability to carry oxygen. Considering the susceptibility of the brain to hypoxemia, the age of RBC transfused to TBI patients may have a potential impact on outcomes.

**Methods:** We conducted an a priori planned analysis of the TBI patients enrolled (n = 217) in the ABLE study, a large multicenter RCT comparing the use of fresh blood (<8 days) to the use of standard issued blood in critically ill patients on mechanical ventilation (ISRCTN44878718). Our primary outcome measure was the Glasgow Outcome Scale extended (GOSe); secondary outcomes were ICU, hospital and 6-month mortality.

**Fig. 26 (abstract P243).**
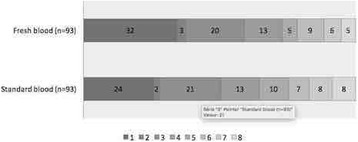
Number of patients for each score of the GOSe

**Results:** Age, Glasgow Coma Scale and main patient characteristics were comparable between groups (fresh group, n = 110; group, n = 107). RBC were stored for 5 ± 3 days in the fresh group and 18 ± 6 days in the standard group (p < 0.0001). The GOSe was available in 93 patients in each group. 26.9% of the patients in the fresh group had a favorable outcome at 6 months (GOSe 5 to 8) as compared to 35.5% in the standard group (p = 0.21). Sliding dichotomy analysis with the GOSe showed no significant difference in outcome for the overall GOSe (Fig. 26). No effect on ICU, in-hospital or 6 months mortality was observed.

**Conclusions:** Our results suggest that transfusing fresh RBC is not associated with improved 6-months neurological outcome in critically ill patients with TBI.

## P244 Transfusion of stored blood induces pulmonary vasoconstriction in critically ill patients after cardiac surgery: a double-blind, randomized clinical trial

### DM Baron, J Baron-Stefaniak, GC Leitner, R Ullrich

#### Medical University of Vienna, Vienna, Austria

**Introduction:** Transfusion of packed red blood cells (PRBCs) stored for 40 days increased pulmonary arterial pressure (PAP) and pulmonary vascular resistance (PVR) in lambs [1]. These vasoconstrictor effects were augmented by endothelial dysfunction. Furthermore, transfusion of PRBCs stored for 40 days increased PAP in obese adults [2]. We hypothesized that transfusion of PRBCs stored for prolonged periods would induce pulmonary vasoconstriction in critically ill patients after cardiac surgery.

**Methods:** This study was performed as a double-blind, parallel-group, randomized clinical trial at the Medical University of Vienna after local ethics committee approval and registration at clinicaltrials.org (NCT02050230). Written informed consent was obtained before enrollment. Critically ill patients requiring one unit of PRBCs as standard care were randomized to receive PRBCs stored for < =14 days (fresh PRBCs; fPRBCs) or standard-issue PRBCs (siPRBCs; the oldest compatible unit available in the blood bank) over 15 min. The increase of PAP during transfusion (Δ PAP) was defined as primary outcome parameter. PAP, mean arterial pressure (MAP), and cardiac output (CO) were measured at baseline and after transfusion. PVR and systemic vascular resistance (SVR) were calculated. Concentrations of macrophage migration inhibitory factor (MIF) and syndecan-1 (SDC1) in serum and in supernatant of PRBCs were measured with ELISA. Statistical analysis was performed with Welch’s test.

**Results:** Six patients received fPRBCs (storage duration 10 ± 3 days) and five patients received siPRBCs (storage duration 33 ± 4 days). Demographic patient data did not differ among groups. Δ PAP was greater after transfusion of siPRBCs than fPRBCs (7 ± 3 vs. 2 ± 2 mmHg, P = 0.01). Similarly, PVR (81 ± 50 vs. -1 ± 37 dyn · s · cm-5, P = 0.01) and SVR (166 ± 61 vs. 9 ± 72 dyn · s · cm-5, P = 0.004) increased to a greater extent after transfusion of siPRBCs than fPRBCs. Changes in MAP (P = 0.12) and CO (P = 0.21) did not differ among groups. siPRBCs increased systemic MIF concentrations by 56 ± 70% (P = 0.02), while fPRBCs did not (P = 0.54). Concentrations of MIF were greater in supernatants of siPRBC units (521 ± 436 ng/ml) than in those of fPRBC units (158 ± 115 ng/ml, P = 0.002). Systemic SDC1 concentrations increased after transfusion of fPRBCs and siPRBCs (P < 0.05), but did not differ among groups (P = 0.99).

**Conclusions:** Transfusion of standard-issue PRBCs induces pulmonary vasoconstriction in critically ill patients after cardiac surgery.

**References**

1. Baron DM et al. Anesthesiology 116:637–47, 2012

2. Berra L et al. AJRCCM 190:800–7, 2014

## P245 Reducing the level of blood loss in patients with obstetric massive bleeding

### O Tarabrin, A Mazurenko, Y Potapchuk, D Sazhyn, P Tarabrin

#### Odessa National Medical University, Odessa, Ukraine

**Introduction:** Using of fresh frozen plasma (FFP) in massive bleeding therapy is associated with the risk of transfusion-related acute lung injury (TRALI), one of the main causes of death after transfusion. Use of prothrombin complex concentrate (PCC) may reduce this risk..

**Methods:** Research involved 69 patients with massive bleeding (MB) after cesarean section. Patients were divided into 2 groups. Both groups received packed red blood cells. In first group (n = 18) therapy of obstetric bleeding included PCC in a dose of 1 ml/kg (25 IU/kg). Patients of 2nd group (n = 54) received FFP in a dose of 20 ml/ kg. Method of low-frequency piezoelectric thromboelastography (LPTEG) was used to study the functional state of the hemostasis system.

**Results:** We checked in patients with MB next constants -Intensity of contact coagulation (ICC), Intensity of coagulation drive (ICD), clot maximum density (MA) and fibrinolytic activity - Index of retraction and clot lysis (IRCL). ICC in patients with MB was reduced by 46.72%, ICD was less than normal at 57.62%, MA was reduced by 85.77%, IRCL was 87,63% above the norm. 1st group which received infusion of PCC - ICC reduced by 15.46%; ICD was less than normal at 9.34%, MA was reduced by 17.58%, IRCL was 14,32% above the norm 2 hours after, and became normal 4 hours after infusion of PCC. Patients of 2nd group received FFP have ICC reduced by 24.83%, ICD reduced by 22.64%, MA was reduced by 33.12%, IRCL was above the norm to 24.38% 4 hours after, and became to the normal 6 hours after infusion of FFP. In the 2nd group the study showed the two cases of transfusion related acute lung injury..

**Conclusions:** Using of PCC can reduce the use of blood transfusions in the intensive care unit. Therefore it can prevent development of TRALI syndrome. The prothrombin complex concentrate reduce the level of blood loss, decrease volume of transfusion packed red blood cells and infusion therapy.

## P246 Methods of correction of coagulopathy in patients with polytrauma

### O Tarabrin, A Mazurenko, Y Potapchuk, D Sazhyn, P Tarabrin

#### Odessa National Medical University, Odessa, Ukraine

**Introduction:** According to the World Health Organization, approximately 3.5 million people die each year in as a result of injuries of various kinds [1]. In developed countries, injuries occupy the third place in the list of causes of mortality. Trauma-induced coagulopathy (TIC) is one of the main reason of lethal outcome for patients with polytrauma.

**Methods:** This study involved 105 patients with traumatic injuries: concomitant skeletal trauma, fractures of pelvis, femur, humerus. All patients were divided into 2 groups. Both groups received TXA 15 mg/kg during 10 minutes followed by an infusion 1 g during 8 hours. 1st group (n = 53) received additional prothrombin complex concentrate (PCC) 1 ml/kg (25 IU/kg), 2nd group (n = 52) received FFP in a dose 15 ml/kg. Method of low-frequency piezoelectric thromboelastography (LPTEG) was used to study the functional state of the hemostasis system.

**Results:** Next constants of blood coagulation have been checked -Intensity of contact coagulation (ICC), Intensity of coagulation drive (ICD), clot maximum density (MA) and fibrinolytic activity - Index of retraction and clot lysis (IRCL). In both groups before therapy ICC was reduced by 28.77%, ICD- less than normal at 38.61%, MA was reduced by 73.52%, IRCL was 91,18% above the norm. Patients of 1st group according to LPTEG had ICC reduced by 15.22%, compared to the norm; parameters of coagulation and fibrinolysis have reliable trend toward normal and decreasing the activity of fibrinolysis index reaches normal reference values. Patients of 2nd group have ICC reduced by 25.62%, ICD reduced by 19.97%, MA was reduced by 24.22%, compared to the norm and IRCL was in the normal range. As the result, patients of the 1st group needed less volume of blood transfusions as patients of the 2nd group.

**Conclusions:** The use of prothrombin complex concentrate in combination with tranexamic acid can reduce the severity of disorders of blood coagulation in patients with traumatic injuries, reduce mortality and reduce the volume of blood transfusions.

[1]- Guidelines for essential trauma care, WHO, 2014

## P247 Fresh frozen plasma transfusions impact pneumonia rates after cardiac surgery

### A González Pérez, J Silva

#### Hospital Universitario Central de Asturias, Oviedo, Spain

**Introduction:** Pneumonia is a known complication that may arise after cardiac surgery and increases the morbidity and mortality of these patients.[1] On the other hand, it is known that blood compounds transfusion increases risk infections of postoperative cardiac surgery patients.As described [2], the transfusion of FFP presents a greater risk of developing acute lung injury than other blood components. The aim of this study is to analyze the relationship between intraoperative cardiac surgery FFP transfusions and the development of pneumonia during ICU stay.

**Methods:** Retrospective observational study of a cohort of patients that underwent to cardiac surgery at our institution between 2006 and 2014. Demographic and clinical variables were collected from de Hospital Database. FFP units transfused during intraoperation procedure and the develop of nosocomial pneumonia were analysed to determinate their relationship. A multivariate logistic regression model was used to estimate the Odds Ratio of pneumonia associated with the FFP transfusion with confidence interval of 95%, p < 0.05. Values expressed as mean +/- SD or %.

**Results:** Cohort of 3563 patients ; 64.8 males; 67.78+/- 11.13 years old; BMI 28.36 +/- 6.81 ; Logistic EuroScore 6.36 +/- 4.85; ICU stay 6.58 +/- 15.41 days ; postoperative bleeding at first 24 hours 560.74 +/- 447.95 cc; preoperative haemoglobin 13.58 +/- 5.72 gr/ dL. By pass time 104.31 +/- 231.75; cross clamp time 71.76 +/- 32.74 minutes. 265 patients were diagnostic of pneumonia during the ICU stay. These patients were most frequently males, older, less BMI, higher EuroScore and lower hematocrit and hemoglobin but no significant differences were found in these variables. Pneumonia group patients were transfused more FFP (1.31 +/- 1.85 vs 2.27 +/- 1.94 units p < 0.001) and presented a longer duration of CPB and clamp times (97.79 +/- 45.8 vs 126.19 +/- 3.52; p <0.001 and 70.50 +/- 33.44 vs 98.88 +/- 24.09; p < 0.001 respectively. The relative risk of developing pneumonia was 1.50 for the group that received transfusion of blood components. In the multivariate analysis patients exposed to FFP transfusions had a significant OR 1.149 of pneumonia 95% CI (1.046 to 1.261) p < 0.001.

**Conclusions:** Known deleterious effects that can cause transfusions of blood components, clinical teams should explore strategies to prevent unnecessary transfusions. The transfusion of FFP demonstrated in this study that it could increase the morbidity after cardiac surgery. Further studies are needed to corroborate these results.

**References**

(1) Likosky DS et al. Red Blood Cell Transfusions Impact Pneumonia Rates After Coronary Artery Bypass Grafting. Ann Thorac Surg. 2015 Sep;100(3):794–800;

(2) Khan H. et al. Fresh-frozen plasma and platelet transfusions are associated with development of acute lung injury in critically ill medical patients.Chest. 2007 May;131:1308–14.

## P248 Effect of perioperative use of tranexamic acid on the blood loss in cemented total hip replacement

### V Artemenko, A Bugaev, I Tokar, S Konashevskaya

#### MC INTO-SANA, Odessa, Ukraine

**Introduction:** Amount of cemented total hip replacement (CTHR) has grown due to the aging of patients, new mode treatments of degenerative hip disease [1]. After CTHR can be many complications: DVT, APE, and coagulopathy with blood loss[2,3]. That patients need either RBC and FFP transfusions in the early postoperative period or new blood safe methods. Among other agents, tranexamic acid (TXA) affects on blood loss[2]. We analyzed the effect of perioperative use of TXA on the blood loss in patients with CTHR and even made changes in our perioperative guidelines.

**Methods:** The case cohort study involved 18 pts. under CTHR (2014-2016). Base group (n-9, age 73,8 ± 11,3 yrs). Control group retrospective analysis of medical records(n-9, age 78,2 ± 8,9 yrs). In base group TXA was injected at initial dose 15 mg/kg in 30 min prior to surgery, and following infusion 2.5 mg/kg/hr in 12 hr. In the control group there was not an infusion of TXA. Anesthesia in both groups was similar: the operation performed under conduction anesthesia- lumbar plexus block with Ropivacaine0.5%-30 mL, sedation by dexmedetomidine 1.5-2 mg/kg/hr. Length of surgery 240 ± 45 min. At first postop. day patients were in ICU. If hemoglobin level was less 70 g/L RBC transfusion was performed at 10 ml/kg and FFP 15 ml/kg – target level 100 g/l hb. The blood loss evaluated by gravimetric meth.

**Results:** The results showed that both groups were fully compatible by sex, age, type of surgery and anaesthesia. In base group 2pts. (22.2%)required blood transfusion, mean blood loss(ml) was 243.75 ± 75 vs. the control group where 5 pts. (55.5%) required blood transfusion due to blood loss 416 ± 210. this is obviously different but not statistically significant in a small group (p^>^0.05). The amount of RBC in base group was significally lower 116.25 ± 8,1vs.491.5 ± 560 in control group (p^<^0.0006). Pearson's coefficent 0.323 moderate. Based on results we applied an TXA in our local perioperative guideline.

**Conclusions:** Perioperative use of tranexamic acid:1.Decreased blood loss during CTHR.

2. Reduced the amount of RBC from 416 ± 210 ml till 116.25 ± 8,1 ml.

3. Additional study with bigger group is required to give more statistically significant results.

**References**

1. Lemaire R. Strategies for blood management in orthopaedic surgery J.Bone Joint Surg. Br.2008;90-B(9):1128–1136.

2. Ellis M.H., at.all.: The effect of tourniquet application and tranexamic acid on the procoagulant systems J.Clin.Anesth. 2001; 13:509–513.

3. Mahdy M., Webster N.R. Perioperative systemic haemostatic agents. B.J.A 2004;93(6):842–858.

## P249 The effect of volunteer’s gender on the reference range for INNOVANCE® PFA-200

### IM Kolesnikova, EV Roitman

#### Pirogov Russian National Research Medical University, Moscow, Russia

**Introduction:** Platelet Functional Analyzer (PFA) defines platelet aggregation in conditions closest to the natural. Inside PFA-cartridge the citrated whole venous blood moves through a inner capillary towards an aperture with membrane coated some platelets inductors. The time needed for occluding the aperture by plug formation is called closure time (CT). PFA User Manual indicates the reference ranges of some CT defined from European population but does not account for gender factor. However the aim was to establish PFA reference intervals for adult population living in Moscow depending on the volunteer’s gender.

**Methods:** Study’s population consisted of 90 healthy volunteers (males – 66, females – 24). We used S-Monovette PFA (Sarstedt, Germany) blood collection system (CN 3,8%; pH 5,5). INNOVANCE® PFA-200 (Simens, Germany) cartridges with collagen and adenosine diphosphate (COL/ADP); with collagen and epinephrine (COL/EPI) and cartridge P2Y were applied. Reference intervals were presented as 5th-95th percentile. Obtained reference ranges were compared to European population reference intervals. Besides reference range gender differences were evaluated. Differences were detected using Mann — Whitney test.

**Results:** Obtained COL/ADP reference interval reached CT 96-115 sec that was more narrow than for European population (CT 68-121 sec). No significant differences related to gender-based volunteers, were found (p > 0.05). For COL/EPI test the general reference range indicated CT between 158-189 sec that was longer than the same for European population (CT 84-160 sec). Besides females had significantly short CT for COL/EPI than males (males 161-200 sec, females 131-193 sec; p < 0.05). According to PFA User Manual the normal values of CT for P2Y test should be fewer than 106 sec. We obtained reference range for P2Y test within 73-88 sec regardless of volunteer's gender.

**Conclusions:** The obtained data showed average ranges for urban population of Central Russia. It seems that normal PFA aggregation might have different CT ranges for men and for women. Aggregation response to epinephrine is different depending on gender factor. Shorter CT in a whole and wider range of reference values of CT was defined for women. These differences may be associated with a positive effect on intracellular free calcium of the progesterone metabolites. Platelet stimulation of ADP leads on the one hand their activation and on the other hand an increase in intracellular calcium concentration. It explains the absence of differences in the reference ranges for the cartridges COL/ADP and P2Y.

## P250 The hemostasis changes in bloodless liver transplantation

### T Rengeiné Kiss, Z Máthé, L Piros, E Dinya, E Tihanyi, A Smudla, J Fazakas

#### Semmelweis University, Budapest, Hungary

**Introduction:** On international scale Massicotte had published 600 bloodless liver transplants (OLTx) based on acute normovolemic hemodilution and “cell saver” technique, but the special characteristics of hemostatic changes in these patients were not reported [1]. The aim of the study was to evaluate the hemostatic changes defined as hemostasis reserve capacity (HRC) in the first perioperative 48 h of bloodless OLTx.

**Methods:** The HRC in bloodless OLTx pts (n = 26) was designed by the implementation of the “Görlinger pyramid methodology” based on lower reserves of RBCs, factor levels and thrombocytes (FI: 1 g/l, FII-V-VII-X: 30%, FXIII: 60%, Platelets: 30.000 109/l, AT III: 40%, Hematocrit: 27%). Laboratory measurements followed by the counting of HRC were done before OLTX (T1), at arrival on ICU (T2) and 12-24-48 h after OLTX (T3-5). Demographic data of the patients, the hemodynamic parameters, the vasopressor requirement, the ventilation support, the MELD, DRI, APACHE II, SOFA scores and LOS were also recorded. The data are given mean ± SD and were analyzed with rAnova, Fisher exact by SPSS 20.0.

**Results:** The cohort MELD, DRI and APACHE score were: 11 ± 0.68, 1.56 ± 0.06 and 12.8 ± 2.6. None of the pts needed RBC, FFP or platelets replacement at all, but 7 pts of 26 required FI (2 g) or PCC (1500 IU) substitution in absence of coagulopathy bleeding during surgery. The intraoperative weakening and postoperative improvement of HRC was noticed (T1 = T5). A significant decrease of factors were observed due to the intraoperative vascular bleeding, factor consumption and dilution (T1-T2): FI 0.9 ± 0.5 g/l; FII 28 ± 11%, FV 44 ± 23%; FVII 32 ± 18%; FX 47 ± 23%; FXIII 29 ± 23%; ATIII 36 ± 17%; p < 0.001. Platelet count were found modified by 0.2 ± 50% of the preoperative value. Only 7 pts of 26 required FI (2 g), FXIII (1250 IU) substitution in lack of bleeding 48 h after surgery. As an indicator of the early graft synthesis the factor levels significantly increase in the first 24 h (T2-T4) FI with 0.89 ± 0.65 g/l, and next 24 h (T3-T5) FII-V-VII with 22 ± 14%, 48 ± 29%, 31 ± 28% p < 0.001, except FXIII which remain decreased by 10 ± 19% of the preoperative value. No correlation between DRI and HRC improvement was found.

**Conclusions:** The individualized, multimodal approach of hemostasis based on repeated evaluation of HRC focuses on the weakest link within the system. In the absence of bleeding some patients needed only factor replacement, so besides bloodless OLTx, the algorithm could be useful in Jehovah witness patient’s hemostasis management as well.

**Reference**

1. Massicotte et al.: Transplantation. 2014; 98(2): e13–5.

## P251 Novel technique to monitor effect of transfusion on mitochondrial oxygenation

### R Ubbink, P Boekhorst te, E Mik

#### Erasmus Medical Center Rotterdam, Rotterdam, Netherlands

**Introduction:** Red blood cell transfusion (RBCT) is not without risks and unnecessary transfusions should be avoided [1]. No objective methods are available to determine an individual’s need for RBCT [2, 3]. Since ultimately the mitochondria are the target for oxygen delivery, it seems reasonable to use mitochondrial oxygen tension (mitoPO2) as a measure. In animal experiments a correlation between cutaneous mitoPO2 and critical hematocrit has been demonstrated [4]. The recent development of the COMET measuring system allows cutaneous mitoPO2 measurements in humans.

**Methods:** The COMET (Photonics Healthcare BV, Utrecht, The Netherlands) uses the protoporphyrin IX-Triplet State Lifetime Technique (PpIX-TSLT) to measure the mitoPO2 [5]. It is being evaluated in an ongoing IRB-approved study in chronic anemia patients. Following informed consent a 5-minolevulinic acid (ALA) patch was applied the evening before RBC transfusion to induce mitochondrial PpIX in the skin.

**Fig. 27 (abstract P251).**
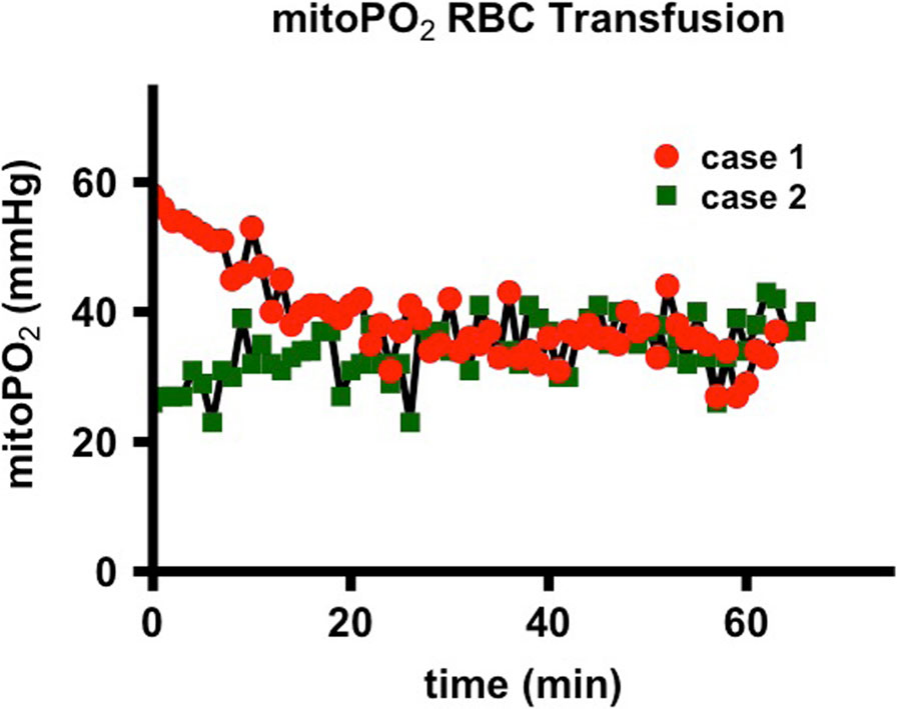
MitoPO[sub]2[/sub] during blood transfusion

**Results:** Two RBCT cases are presented. First case is a man with myelodysplastic syndrome Hb 4.8 mmol/L, mitoPO2 decreased during RBCT. Second case is a man with X-linked sideroblastic anemia Hb 3.8 mmol/L, mitoPO2 increased during transfusion, seen in Fig. 27.

**Conclusions:** Our preliminary data show that COMET is able to detect opposing responses of mitoPO2 during RBCT. Providing a novel clinical tool for research in transfusion medicine.

**References**

1. Spahn DR et al.: Transfus Med Hemotherapy 2015; 42:110–114

2. Stowell CP.: Transfusion 2009; 49:620–621

3. Shander A et al.: Br J Anaesth 2012; 109:55–68.

4. Römers LHL et al.: Anesthesiology 2016; 125:124–32

5. Mik EG et al.: Nat Methods 2006; 3:939–45

## P252 Thromboelastography goal-directed haemostatic therapy (TEG-GDHT) during open thoracoabdominal aortic aneurysm (TAAA) repair

### L Caneva^1^, G Ticozzelli^1^, S Pirrelli^2^, D Passador^1^, F Riccardi^1^, F Ferrari^1^, EM Roldi^3^, M Di Matteo^3^, I Bianchi^3^, GA Iotti^1,3^

#### ^1^IRCCS Policlinico S. Matteo, SC Anestesia e Rianimazione 2, Pavia, Italy; ^2^IRCCS Policlinico S.Matteo US Patologia Aorta Toracica-UOC Chirurgia Vascolare, Pavia, Italy; ^3^Università degli Studi di Pavia, Pavia, Italy,

**Introduction:** TAAA operations are associated with extensive blood loss and often require high-dose allogeneic blood transfusion, mostly because of impaired coagulation[1]. Assessment of clot formation and strength measured by viscoelastic methods (thromboelastography-TEG), is useful for the diagnosis of intraoperatively acquired coagulation disorder. Rapid measurement of functional fibrinogen(FLEV) using TEG is a potentially important new tool, although results obtained by FLEV are different from traditional fibrinogen plasma concentration determined by Clauss method (FibC)[2]. We retrospectively compared a TEG-guided GDHT vs standard care haemostatic therapy for hemocomponents transfusion during TAAA surgery and the concordance on fibrinogen concentration measured by FLEV vs FibC.

**Methods:** We analyzed 16 patients(pts) who underwent TAAA repair from January 2013 to May 2016 in our centre. We divided patients in 2 groups based on transfusion strategy: the TEG-GDHT group(Gteg) and the standard care group(Gsc), defined by the transfusion strategy decided by the anesthesiologist. We collected data on population characteristics, TEG parameters, FibC, hemocomponents transfusion. We analysed FibC by using MultifibrenRU-Siemens-Heathcare as reagent and TEG-5000. We used Mann-Whitney and Bland-Altman tests for statistical analysis. We expressed data as mean ± SD

**Results:** We analyzed 16 patients, comparing Gsc(11pts) vs Gteg(5pts). Compared to Gsc, we found a significantly lower plasma transfusion in Gteg(10.9 ± 5.9 vs 2.8 ± 3.7 p = 0.01); we also noted a tendency to reduction for platelets(7.6 ± 6 vs 3.2 ± 3.4 p = 0.14) and red blood cells transfusion(10.9 ± 8.3 vs 3,6 ± 2.7 p = 0.06), while fibrinogen administration was not different(1.4 ± 1.6 vs 2 ± 1.4 g p = 0.49). When we restricted our analysis to elective surgery, results were similar. Baseline MA was 75,9 ± 3,6 mm, MA-FF 42.7 ± 10.2. The correlation between FLEV and FibC values at baseline was far from strong(778 ± 186 vs 378 ± 100 mg/dL, r2 = 0.3).

**Conclusions:** Use of a TEG-guided GDHT dramatically reduced plasma transfusion in patients undergoing TAAA surgery. In our population with baseline fibrinogen higher than normal, we found a poor correlation between functional fibrinogen assessed by TEG and plasma fibrinogen measured by the Claus method.

**References**

1] N.Rahe-Meyer et al, J Thorac-Cardiovasc-Surg 2009;138:694–702

[2] A.Ågren et al, International-Anesthesia-Research-Society 2014;DOI:10.1213

## P253 Association of red cell distribution width and poor outcome is mainly explained by inflammation: results of a prospective study

### G Zurauskaite, A Voegeli, M Meier, D Koch, S Haubitz, A Kutz, M Bargetzi, B Mueller, P Schuetz

#### Kantonsspital Aarau, Aarau, Switzerland

**Introduction:** There is growing interest on red blood cell distribution width (RDW) as a predictor for disease outcome, but underlying pathophysiological mechanisms are incompletely understood. Our aim was to investigate the prognostic value of RDW in unselected patients presenting to the emergency department (ED) and to study pathophysiological pathways explaining our findings.

**Methods:** Consecutive adult, medical patients seeking ED care were included into this observational, cohort study. We prospectively followed outcome of these inpatients for 30 days. Multivariate regression analysis was used to study association of admission RDW and all-cause 30 days mortality, and as secondary outcomes ICU admission and hospital readmission.

**Results:** The 30-day mortality of the 4273 included patients was 5.6% and increased from 1.4% to 14.3% from the lowest to the highest RDW quartile. RDW was strongly correlated with different pathways particularly inflammation (R^2^ = 0.30; p < 0.001), nutrition (R^2^ = 0.20; p < 0.001) and blood diseases (R^2^ = 0.30; p < 0.001).Overall, there was a strong association of RDW and mortality in unadjusted analysis (OR 1.32; 95% CI 1.27 - 1.39, p < 0.001). However, most of this association was explained by above mentioned pathophysiological pathways with associations of RDW and outcome losing significance when adding these factors into the regression model. In the fully adjusted model, the OR of RDW was 1.02; 95% CI 0.93 – 1.12; p = 0.664. Results were similar for secondary outcomes.

**Conclusions:** Our findings indicate that RDW is a surrogate for different pathophysiological states mirroring chronic inflammation, malnutrition and malignant disease, which explains the strong association of RDW and mortality in unselected medical ED patients.

**Fig. 28 (abstract P253).**
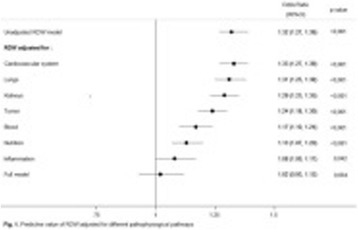
See text for description

## P254 Red cell distribution width at hospital discharge and out-of hospital outcomes in critically ill non-cardiac vascular surgery patients

### G Von Meijenfeldt^1^, M Van der Laan^1^, C Zeebregts^1^, KB Christopher^2^

#### ^1^University of Groningen, Groningen, Netherlands; ^2^Brigham and Women's Hospital, Boston, MA, United States

**Introduction:** Red cell distribution width (RDW) is associated with mortality and bloodstream infection risk in the critically ill. In vascular surgery patients surviving critical care it is not known if RDW can predict subsequent risk of all-cause mortality following hospital discharge. We hypothesized that an increase in RDW at hospital discharge in vascular surgery patients who received critical care would be associated with increased mortality following hospital discharge.

**Methods:** We performed a two-center observational cohort study of adult critically ill non-cardiac vascular surgery patients surviving admission treated between 1997 and 2012 in Boston. Our exposure was RDW measured within 24 hours of hospital discharge and categorized as < =13.3%, 13.3-14.0%, 14.0-14.7%, 14.7-15.8%, >15.8%. The primary outcome was all cause mortality in the 90 days following hospital discharge. Logistic regression was used to determine the RDW-mortality association.

**Results:** The cohort included 4,715 patients (male 58%; white 83%; mean age 62.9 years). 90 and 365-day post discharge mortality was 7.5% and 14.4% respectively. In the cohort, 47.3% were discharged to a care facility and 14.8% of patients were readmitted within 30 days. After adjustment for age, gender, race, Deyo-Charlson Index, acute organ failures, prior vascular surgery and vascular surgery category, patients with a discharge RDW 14.7-15.8% or >15.8% have an adjusted OR of 90-day post discharge mortality of 2.52 (95%CI, 1.29-4.90; P = 0.007) or 5.13 (95%CI, 2.70-9.75; P <0.001) relative to patients with a discharge RDW < =13.3%. The adjusted model has good discrimination for 90-day post discharge mortality (AUC = 0.80; 95%CI 0.78-0.83). Analyzing the adjusted model with and without the discharge RDW term shows significantly improved discrimination for 90-day post-discharge mortality with discharge RDW included (χ 13.89, P < 0.001). The adjusted odds of 30-day readmission in the RDW >15.8% group was 1.52 (95%CI, 1.12-2.07; P =0.007) relative to patients with a discharge RDW < =13.3%. Similar robust associations are present regarding discharge RDW and discharge to a care facility.

**Conclusions:** In critically ill vascular surgery patients who survive hospitalization, an elevated RDW at hospital discharge is a robust predictor of subsequent mortality, hospital readmission and placement in a care facility.

## P255 Thrombotic thrombocytopenic purpura (TTP) in ICU

### P Vernikos, T Melissopoulou, G Kanellopoulou, M Panoutsopoulou, D Xanthis, K Kolovou, T Kypraiou, J Floros

#### Laiko General Hospital, Athens, Greece

**Introduction:** TTP is a rare disorder characterized by thrombus formation in small blood vessels. Only a minority of patients present with the pentad of thrombocytopenia, MAHA (microangiopathic haemolytic anaemia - schistocytes, elevated LDH level, and indirect hyperbilirubinemia), neurological signs (confusion, paresis, aphasia, visual problems, encephalopathy, coma), renal impairment and fever. Deterioration of neurological signs with low Glasgow Coma Score is the main cause of ICU admission.Prompt recognition of TTP and treatment with plasma exchange (PE) reduce mortality which without treatment remains high (90%).

**Methods:** During the last decade, eleven patients (7 males / 4 females) with a mean age of 40, 81 (27 – 76) years old were admitted in ICU because of TTP. Eight of them were intubated while the other 3 weren’t but had low GCS (<10). At the admission in ICU, laboratory tests were: PLT : 7000/mm3 - 20000/mm3 (average 12545), Hct : 16.5% - 24.4%, LDH : 592 – 5445 (average 2254), Bil : 1.46 mg/dl – 8.35 mg/dl (average 3.85), Cr : 0.9 mg/dl – 3.3 mg/dl (average 1.72), InR, aPTT and fibrinogen had normal values, while ADAMTS13 activity ranged from 2 to 18 (average 6.81). Thrombocytopenia and MAHA with normal coagulation tests, neurological symptoms and ADAMTS13 activity < 10% set the diagnosis of TTP. PE with fresh frozen plasma was initiated to all patients within the first 8-12 hours of admission. PE was performed every day for the first 5- 7 days (1 – 1.5 x plasma volume), and then every second day until the resolution of neurological symptoms and/or laboratory tests improvement. Usually, the majority of responses were seen within the first 10 PE.

**Results:** PLT values were improved in 8/11 patients, bilirubin in almost all of them, LDH average value was improved by 52.30%. On ICU discharge, HCT ranged from 15.0 to 33.8%, PLT from 14,000 to 361,000/mm3, creatinine from 0.7 to 4.8 mg/dl, bilirubin from 0.71 to 9.6 mg/dl and LDH from 221 to 3696. All but one patient were discharged from ICU in good neurological status, after 5 - 31 days (average 13.45 d) of ICU treatment.

**Conclusions:** Thrombocytopenia and MAHA with neurological signs and ADAMTS13 activity < 10% is justification to initiate plasma exchange therapy, even if the patient is intubated in ICU. Thrombocytopenia and neurological symptoms improvement are the main indications for patient weaning from mechanical ventilation and discharge from ICU.

## P256 Acute haemorrhage in a large district general hospital – who bleeds and what blood products do they get? do we use those products wisely?

### H Broady, C Pritchett, M Marshman, N Jannaway, C Ralph

#### Royal Cornwall Hospital, Truro, United Kingdom

**Introduction:** In this study we investigated our baseline number of acute haemorrhages and their treatment, to establish feasibility of acquiring viscoelastometry to guide blood product usage. Unnecessary use of blood products in critically ill patients can be harmful[1]. There is evidence that using both thromboelastometry (TEM™) and thromboelastography (TEG™) can reduce blood product transfusion using algorithms and protocols[2].

**Methods:** This retrospective audit included acute bleeds with a request for >4 units of any type of blood product, over a one year period (2015/16). Patients were identified from the blood bank electronic data base and details of bleed type, blood products administered (red blood cells [RBC], fresh frozen plasma [FFP], platelets and cryoprecipitate), were recorded. Average numbers of each product used were calculated for each bleed.

**Results:** 72 patients were included. The three most common causes for bleeding were upper GI (32%), ruptured aortic aneurysm (21%) and trauma (13%). 83% of the patients actually received an acute transfusion of at least one blood product. Major obstetric haemorrhage (MOH) was infrequent (7% of bleeds) but used the most blood products (9.8 units average; 5.2 RBC, 3.4 FFP, 0.6 platelets, 0.6 cryoprecipitate). UGI bleeds were frequent and used large amount of blood products (9.5 units average, 6 RBC, 3.1 FFP, 0.2 platelets, 0.1 cryoprecipitate).

**Conclusions:** Despite the relatively large number of ruptured AAA patients we see annually, they were less likely than other causes to receive large volumes of blood products. This may be due to effective use of intra operative cell salvage. The few women who had a MOH went on to need more blood products than quoted in a recent study using ROTEM guided transfusion (9.8 vs 3.0 units)^2^. Given our results, recently updated AAGBI transfusion committee guidelines^3^ and the evidence that TEG / TEM can reduce acutely transfused blood product usage, we will now pilot a device in our hospital to help guide transfusions during major acute bleeds, and use blood more wisely.

**References**

1. Görlinger K and Saner FH. Prophylactic plasma and platelet transfusion in the critically Ill patient: just useless and expensive or even harmful? BMC Anesthesiology (2015) 15:86.

2. Mallaiah S et al. Introduction of an algorithm for ROTEM-guided fibrinogen concentrate administration in major obstetric haemorrhage. Anaesthesia 2015, 70, 166–175.

3. Klein AA, Arnold P, Bingham RM, Brohi K, Clark R, Collis R et al. AAGBI guidelines: the use of blood components and their alternatives 2016 Anaesthesia 2016; 71:829–842.

## P257 Activated partial thromboplastin time versus diluted thrombin time in argatroban monitoring

### CL Lehane, CK Keyl, EZ Zimmer, DT Trenk

#### Herzzentrum Freiburg Bad krozingen, Bad Krozingen, Germany

**Introduction:** The direct thrombin inhibitor argatroban is recommended for the treatment of patients with confirmed or suspected heparin-induced thrombocytopenia.Monitoring of the anticoagulant effect of argatroban is mandatory to gain information on under- or overdosing, respectively. Underdosing of direct and indirect inhibitors is connected with an increased risk of thromboembolism or recurrent thromboembolism and overdosing is associated with an enhanced risk of major haemorrhage. Because activated partial thromboplastin time (aPTT) correlates poorly with argatroban levels and can be influenced by multiple factors in the perioperative setting, (such as deficiencies in coagulation factors), we hypothesized that the diluted thrombin time (dTT) may have advantages in comparison to the aPTT in argatroban monitoring.

**Methods:** Using plasma specimens from 25 critically ill adult cardiac surgery patients receiving treatment with argatroban. Included were patients with renal failure, and those at at high hemorrhagic and thromboembolic risk. Plasma specimens were used to compare the aPTT (using the BCS-XP analyser Siemens Healthcare Diagnostics GmbH, Marburg, Germany) and the dilute thrombin time (dTT) (using hemoclot thrombin inhibitors, Hyphen BioMed, Neuville-sur-Oise, France). The relationship between argatroban plasma levels and aPTT and DTT, repectively, was analysed using a linear regression model (Sigma Plot 12, Systat Software, San Jose, CA, USA).

**Results:** The dTT correlated better than the aPTT to argatroban plasma concentration. Compared to aPTT, dTT showed a linear relationship to argatroban plasma levels. Thereby, dTT values demonstrating a satisfactory coefficient of determination (R) (R = 0.84 for dTT and argatroban plasma levels versus R = 0.28 for aPTT and argatroban plasma levels; p < 0.001).

**Conclusions:** The current study demonstrates for the first time the relationship between argatroban plasma concentration, aPTT and dTT, in critically ill patients. Our results show that the dTT is superior to the aPTT for monitoring parental argatroban administration. Further studies are needed to evaluate dTT in argatroban monitoring and its correlation with clinical outcomes as well as defining argatroban therapeutic concentration ranges.

## P258 Comparison of two diagnostic scores of disseminated intravascular coagulation in pregnant women admitted to the ICU

### AS Ducloy-Bouthors^1^, MJ Jonard^2^, F Fourrier^1^

#### ^1^CHRU, Lille, France; ^2^Centre Hospitalier, Lens, France

**Introduction:** Objective: To compare the validity of two previously published diagnostic scores of disseminated intravascular coagulation (DIC) in pregnant women admitted to ICU for an acute thrombotic or hemorrhagic complication of delivery and postpartum.

**Methods:** This was a population based retrospective study of 154 patients admitted to ICU for severe delivery and postpartum complications in a University Hospital. A recently published score (adapted to physiological changes of pregnancy and based on three components: platelet count, prothrombin time difference and fibrinogen) was compared to the International Society for Thrombosis and Hemostasis (ISTH) score (based on four components: platelet count, fibrinogen, prothrombin time, and fibrin related marker). Both scores were calculated at delivery, ICU admission (day 0), day 1 and day 2 during the postpartum ICU stay. The validity of both scores was assessed by comparison with the consensual and blinded analysis of two experts. The sensitivity, specificity, and area under the curve (AUC) of each score were calculated at each time and overall by generalized linear mixed model. The agreement between the two scores was evaluated by the Kappa coefficient.

**Results:** The new score had a sensitivity of 0.78, a specificity of 0.97 (p <0.01) and a global AUC of 96% while the ISTH score had a sensitivity of 0.31, a specificity of 0.99 and an AUC of 94% (p <0.01). The Kappa coefficient of correlation between both scores was 0.35. The lower sensitivity of the ISTH score was mainly explained by the lack of fibrinogen and fibrin-related peptides thresholds adapted to the physiological changes of coagulation induced by pregnancy.

**Conclusions:** The new DIC score seems highly discriminant in the subset of patients admitted to the ICU after delivery for an acute specific complication. The ISTH score is not recommended in pregnant women because of its poor sensitivity.

**References**

1. Toh CH, Hoots W. SSC on Disseminated Intravascular Coagulation of the ISTH. The scoring system of the Scientific and Standardisation Committee on Disseminated Intravascular Coagulation of the International Society on Thrombosis and Haemostasis: a 5-year overview. J Thromb Haemost. 2007 5:604–6.

2. Erez O, Novack L, Beer-Weisel R, Dukler D, Press F, Zlotnik A, et al. DIC Score in Pregnant Women – A Population Based Modification of the International Society on Thrombosis and Hemostasis Score. PLoS ONE. 2014. 9: e93240.

## P259 Thromboelastometry analysis of thrombocytopenic dengue patients: a cross-sectional study

### F Piza^1^, T Correa^1^, A Marra^1^, J Guerra^1^, R Rodrigues^1^, A Vilarinho^1^, V Aranda^1^, S Shiramizo^1^, MR Lima^1^, E Kallas^2^, AB Cavalcanti^3^

#### ^1^Hospital Israelita Albert Einstein, Sao Paulo, Brazil; ^2^Universidade de Sao Paulo, Sao Paulo, Brazil; ^3^Hospital do Coração, Sao Paulo, Brazil

**Introduction:** Dengue virus infection (DVI) is a prevalent and potentially fatal viral disease associated with coagulopathy. So far, the coagulation profile of DVI patients with thrombocytopenia has not been assessed through a viscoelastic test such as rotational thromboelastometry. We aimed to describe the prevalence and characteristics of coagulation abnormalities in dengue fever outpatients with thrombocytopenia, addressed by both rotational thromboelastometry and conventional coagulation tests.

**Methods:** This was a cross-sectional study conducted between April 6th and May 5th 2015 in São Paulo, Brazil during a dengue outbreak. Thromboelastometry (ROTEM®) and the conventional coagulation tests prothrombin time (PT), international normalized ratio (INR), activated partial thromboplastin time (aPTT), thrombin time (TT), platelet count and fibrinogen levels were performed in 53 patients with DVI and thrombocytopenia.

**Results:** Despite a median interquartile range (IQR) platelet count of 77 (63-88) x 10 9/L in DVI patients, conventional coagulation tests and plasma fibrinogen levels were within the normal range. Subjects demonstrated hypocoagulability in 71.7% (38/53) in INTEM and 54.7% (29/53) in EXTEM DVI patients. FIBTEM analyses detected only 5.7% (3/53) with hypocoagulability among this population. The median (IQR) clotting time (CT), clot formation time (CFT) and maximum clot firmness (MCF) on INTEM were, respectively, 177 (160-207) sec, 144 (108-178) sec and 48 (42-52) mm. On EXTEM, median (IQR) CT, CFT and MCF were, respectively, 69 (65-78) sec, 148 (126-198) sec and 49 (44-55) mm. Median (IQR) MCF on FIBTEM was 15 (13-18) mm.

**Conclusions:** Thromboelastometry impairment is highly prevalent in DVI patients with thrombocytopenia, particularly in INTEM and EXTEM analyses, while standard coagulation tests are normal in this setting. Clinical implications remain to be established.

**References**

Chen R, Vasilakis N. Dengue--quo tu et quo vadis? Viruses. 2011;3(9):1562–608.

2. Guzmán MG, Kouri G. Dengue: an update. Lancet Infect Dis. 2002;2(1):33–42.

3. Kurane I. Dengue hemorrhagic fever with special emphasis on immunopathogenesis. Comp Immunol Microb. 2007;30(5-6):329–40.

4. Favaloro EJ, Lippi G. Coagulation update: What's new in hemostasis testing? Thromb Res. 2011;127:S13–S16.

## P260

### Withdrawn

## P261 Coagulopathy during ECLS is equally corrected by circuit removal or exchange

### M Donoso, P Vargas, J Graf

#### Clinica Alemana, Santiago, Chile

**Introduction:** Coagulation is a major concern during ECLS, and disseminated intravascular coagulation has been described as a complication. We have observed that thrombopenia and hypofibrogenemia are usual manifestations of ECLS-related coagulopathy (ECLS-RC). We describe a case series of ECLS-RC that was managed either by circuit removal or exchange.

**Methods:** Retrospective analysis of patients from our ECLS cohort between July 2014 and November 2016 who developed ECLS-RC (defined as thormbopenia and/or hypofibrogenemia) and required either circuit removal or exchange. We excluded circuit exchange or removal for other reasons. The decision of circuit removal or exchange was made on clinical grounds.

**Results:** From a total of 23 ECLS runs we found 4 cases of circuit removal and 4 cases of circuit exchange due to ECLS-RC in 7 patients. Epidemiological data are shown in Table 15.

**Fig. 29 (abstract P261).**
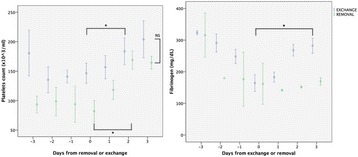
Time course of platelet/fibrinogen levels referred to circuit removal/exchange

We found a similar trend of ECLS-RC progression and resolution between circuit exchange and removal (Fig. 29). We found no evidence of gross hemolysis, hypoprothrombinemia or circuit malfunction in these cases. We did not screen D-dimer and fibrin degradation products routinely. There were no bleeding complications related to this coagulopathy.

**Conclusions:** These data suggest that in ECLS-RC the circuit behaves like a diseased organ that self-perpetuates coagulopathy; if unable to wean ECLS, circuit exchange may therefore be a valid approach in this scenario. Given the retrospective nature of the study and the small number of cases, prospective studies are warranted to confirm this hypothesis.

**Table 15 (abstract P261).** Patients characteristics

Median (range)	Exchange (n = 4)	Removal (n = 4)
Age (years)	64 (36-74)	42(36-68)
APACHE II	32 (31-37)	34 (24-37)
Circuit run (d)	11.5 (9-15)	8.5 (5-13)
Influenza A (n)	3	3

## P262 Outcomes and factors influencing outcome in patients admitted to the intensive care unit (ICU) following allogeneic stem cell transplant

### J McCartney^1^, S Ramsay^1^, K McDowall^1^, I Novitzky-Basso^2^, C Wright^1^

#### ^1^Queen Elizabeth University Hospital, Glasgow, United Kingdom,^2^Beatson West of Scotland Cancer Centre, Glasgow, United Kingdom

**Introduction:** ICU admission post allogeneic stem cell transplant (SCT) is known to be associated with a high mortality rate. We reviewed the requirement for ICU post SCT in our population and identified factors associated with adverse outcomes.

**Methods:** Retrospective observational study for purpose of service review. Patients with history of allogeneic SCT for haematological conditions admitted to ICUs serving the Scottish Blood and Marrow Transplant service in Glasgow, Scotland, between 1st of January 2010 and 18th of May 2016 were included. Local ICU patient database was interrogated. Data on baseline demographics, APACHE II score, critical care interventions, and presence of graft versus host disease (GVHD) was collected – these criteria were selected based on previous studies [1]. Univariate analysis was performed using MedCalc to identify factors associated with primary outcome measure of mortality at 6 months.

**Results:** A total of 20 patients were admitted to ICU following allogeneic SCT during this time period. 65% (n = 13) of patients were male with a mean age of 34 for the total cohort. Overall 6 month mortality rate was 65% (n = 13). 9 patients died prior to ICU discharge with a further 4 patients prior to final discharge from hospital. All patients who survived to hospital discharge were alive at 6 months post ICU admission. The following factors were associated with death at 6 months on univariate analysis: Increasing age (p = 0.03), increasing APACHE II score (p = 0.03), requirement for invasive ventilation (p = <0.01), and requirement for renal replacement therapy (RRT) (p = 0.02), indeed no patients who required invasive ventilation or RRT survived to 6 months. Of note presence of suspected GVHD was not associated with adverse outcome (p = 0.46).

**Conclusions:** In our cohort patients who survived to hospital discharge did well and were still alive at 6 months, that said it is a small cohort and the mortality rate is significant. Factors associated with death at 6 months on univariate analysis were increasing age, increasing APACHE II score, requirement for ventilation or renal replacement therapy – this is comparable with the current literature [1]. We advocate a trial of intensive care in critically ill patients following allogeneic SCT, however patients and their relatives should be aware at the outset that this carries a high risk of death.

**Reference**

1. Orvain C et al: Blood, 124:2465, 2014

## P263 Outcome of mechanically ventilated patients with hematologic malignancies and acute respiratory failure treated with different ventilation strategies

### M Grgic Medic^1^, L Bielen^1^, V Radonic^2^, O Zlopasa^1^, N Gubarev Vrdoljak^1^, V Gasparovic^1^, R Radonic^1^

#### ^1^Clinical Hospital Zagreb, Zagreb, Croatia; ^2^Emergency Medicine Center Sisak, Sisak, Croatia

**Introduction**

Acute respiratory failure (ARF) is the most frequent reason for admission of patients with hematologic malignancies (HM) to intensive care unit ICU. The survival rate of patients with HM requiring mechanical ventilation (MV) in the ICU has improved over the last few decades, but the need for MV tends to aggravate their prognosis. Non-invasive ventilation (NIV) was associated with a decreased mortality compared to invasive mechanical ventilation (IMV), possibly by avoiding complications related to orotracheal intubation. The objective of this study was to identify factors affecting the in-hospital mortality of mechanically ventilated patients, and to assess the role of NIV.

**Methods**

We prospectively analyzed patients with HM requiring MV for ARF in a 4 year period. We identified factors within 24 h of ICU admission associated with ICU mortality in univariate analyses, and compared patients treated with IMV or NIV as initial strategy. The effect of NIV on mortality was assessed using a pair-wise matched exposed-unexposed analysis.

**Results**

A total of 146 hematological patients (median age 50+/-15 years, 66% women) required MV. Overall mortality rate was 76.7%. At admission, 104 (71,2%) patients required IMV, and their mortality rate was 82,7%. NIV was initially started in 42 (28,8%) patients and was successful in 19/42 (45,2%), i.e. their respiratory status was stabilized with NIV and there was no need for IMV. Mortality rate of patients who failed NIV was 91,3% (21/23 patients), and was 26.3% in group treated only by NIV. Patients treated with IMV had higher APACHE II score (p < 0.01) and required vasoactive therapy more often (p < 0.0001). In the univariate analysis, ICU mortality was significantly associated with the need for vasopressors on the day of admission to the ICU (p < 0.0001), IMV as initial strategy (p < 0.0001), APACHE II and SOFA at admission (P < 0.001, and p < 0.01, respectively). Forty two patients who received NIV were matched for APACHE II with 84 patients who required immediate intubation on a 1:2 basis. The ICU mortality rate was 65.4% in NIV group and 85% in IMV group (p 0.076).

**Conclusions**

The ICU mortality rate of patients treated with MV remains high. Small proportion of patients is suitable for NIV. The IMV was associated with higher morality, but we could not clearly demonstrate the protective role of NIV. After mathing patients for APACHE II score, a lower mortality trend in NIV group was observed. In selected patients with HM and ARF, NIV trial could be a good therapeutic strategy after careful clinical assessment.

## P264 Outcomes in hematological patients in critical care ward

### G Narváez, D Cabestrero, L Rey, M Aroca, S Gallego, J Higuera, R De Pablo

#### Hospital Universitario Ramón y Cajla, Madrid, Spain

**Introduction**

The aim of this study is to identify factors that increase mortality in patients with hematological conditions who require admission to intensive care unit.

**Methods**

An observational and retrospective study was conducted, in which we reviewed all patients that were admitted into the Intensive Care Unit (ICU) of a University Hospital, with hematological diseases between May 2013 to February 2016. The analyzed variables were age, SOFA, APACHE II; SAPS; hematological disease, failure of organ and therapy used.

**Results**

A total of 140 patients (p) met inclusion criteria, with an average age of 55.11 ± 14.9 (12-82). Of those, 58% were male and 42% were female. The mean SOFA severity score of 9.3 ± 4.3 (2-20), APACHE II 22.64 ± 13.15 (3-65) and SAPS 2: 50.27 ± 20.82 (2-101). The underlying hematological condition was leukemia (53%), lymphoma (24%), multiple myeloma (15%) and a solid tumor (8%). Mortality rate was 38.6%.

Following organ failures were found in this group of patients: hemodynamic failure in 77.1%, respiratory failure in 62.9%, hepatic failure in 20.7% and metabolic failure in 7.1% (Table 16). Each patient had a mean of 2.32 ± 1.66 (0-6) number of organ failures, with 72,1% of them requiring vasopressors, 53.6% mechanical ventilation and 26.4% renal replacement therapy (RRT) (Table 17).

Applying non parametrical tests, severity scores were related with mortality: SOFA (p < 0.0001), APACHE II: (p < 0. 0001) and SAPS 2: (p < 0.0001). Using the Chi square test we found that the hemodynamic failure is related with mortality (p < 0.0001) equal as respiratory failure p < 0.0001, renal failure p < 0.0001 and hepatic failure p < 0.05. Applying same test, used therapies as vasoactive drugs (p < 0.0001), mechanical ventilation (p < 0.0001), RRT (p < 0.003) and increased number of failure (p < 0.0001) were associated with mortality.

**Conclusions**

Severity scores SOFA, APACHE II and SAPS 2 were found to be useful outcome predictors of mortality in hematological patients requiring Critical Care setting. Hemodynamic and respiratory failure were associated with a higher mortality rate, as well as a higher number of organ failures.

Main therapies associated with a poor outcome were mechanical ventilation, followed by hemodynamic support and renal replacement therapy. As a highlight, we found the use of vasopressors to be associated with an increased in ICU mortality, regardless of the used dose.

**Table 16 (abstract P264).** See text for description

Organ Failure	Incidence	Mortality NO	Mortality YES	p
Hemodynamic	77.1% (108p)	40.7%	51%	<0.001
Respiratory	71.4% (100p)	36.4%	35%	<0.001
Renal	62.9% (88p)	31.4%	31.4%	<0.003
Hepatic	20.7% (29p)	7.9%	12.9%	<0.05
Metabolic	7.1% (10p)	2.1%	5%	0.45

Legend: 6 cm

**Table 17 (abstract P264).** See text for description

Therapies	Incidence	Mortaliity NO	Mortality YES	p
Vassoactive drugs	72.1% (101p)	37.1%	35%	<0.001
Mechanical Ventilation	53.6% (75p)	19.3%	34.3%	<0.001
RRT	26.4% (37p)	10.7%	15%	<0.001

Legend: 6 cm

## P265 Characteristics and outcome of haematopoietic stem cell transplant recipients admitted to the intensive care unit

### L Rey González, G Narváez Chávez, J Higuera Lucas, D Cabestrero Alonso, M Aroca Ruiz, L Jaramillo Valarezo, R De Pablo Sánchez

#### Hospital Ramón y Cajal, Madrid, Spain

**Introduction**

Haematopoietic stem cell transplantation (HSCT) is a standard procedure for patients with haematological malignancies. Advances in the treatments have contributed to incrementing the number of patients admitted to Intensive Care Units (ICU). The aim of this study is to describe the characteristics and prognosis of these patients.

**Methods**

Retrospective analysis of HSCT recipients admitted to the ICU of a tertiary hospital between May 2013 and February 2016. The analysed variables were: age, sex, type/stage of haematological disease, type/time of HSCT: autologous (Au) /allogenic (Al), SOFA, APACHE II, SAPS II, graft-versus-host disease (GVHD), reason for admission, organ failures, organ supports, presence of infection, mortality (ICU and at 30 days).

**Results**

236 patients were transplanted in study period (128 Au, 108 Al): 17.79% were admitted to ICU (57.1% male), mean age of 50.55 ± 11.81. Previous diagnoses were: leukaemia 64.3%, multiple myeloma 21.4%, lymphoma 14.3%; 61.9% had a complete response at the moment of their admission. 81% had received an Al transplant. 26% was admitted to ICU before the 30th day after HSCT, and 50% before the 100th day. 59.9% were diagnosed with GVHD (intestinal 42.9%, cutaneous 35.7%, hepatic 35.7%, pulmonary 7.1%).

The main reason for admission was respiratory failure (45.2%), sepsis (33.3%) and haemodynamic instability (16.7%). The severity scores were: SOFA 10.71 ± 4.50, APACHE II 23.31 ± 10.53, SAPS II 49-45 ± 22.98. Observed organ failures: respiratory 88.1%, hemodynamic 85.7%, renal dysfunction 73.8%, liver failure 26.2% and metabolic disorders 4.8% (mean number of failures 2.88 ± 1 .38). 83.3% needed vasopressors, invasive mechanical ventilation (IMV) was provided in 57,1% and 28,6% required renal replacement therapy (RRT).

The presence of infection was documented in 83.3% of the sample: bacterial (50%), viral (23.8%), fungical (21.4%). The most frequent focus of infection was the respiratory system (50%).

ICU mortality was 50% and mortality within 30 days was 66,6%.

Appling non parametrical test, high APACHE II and SAPS II scores have relation with mortality (p = 0.03 and p = 0.05).

Appling Chi square test, only vasopressors and IMV were significant risk factors for ICU and 30-days mortality (p = 0.04, OR 8 and p < 0.0001, OR 15, respectively).

**Conclusions**

HSCT recipients have poor prognosis in ICU.

Both, mechanical ventilation and vasopressors were risk factors for ICU mortality and mortality within the first 30 days after ICU admission.

APACHE II and SAPS II scores may be useful in severity evaluation of HSCT recipients admitted to the ICU.

## P266 Outcome of hemato-oncology patients transferred to the critical care unit in the last five years at Royal Marsden Hospital

### A Quinza Real^1^, TW Wigmore^2^

#### ^1^Hospital La Fe, Valencia, Spain; ^2^Royal Marsden Hospital, London, United Kingdom

**Introduction**

The Royal Marsden Hospital (RMH) is a tertiary referral cancer center and is a 2 site hospital, with the CCU geographically remote from the center caring for hemato-oncology (HO) patients. In 2013, the RMH changed the care model for critically ill HO patients, with the pre-existing high dependency unit (HDU) at the remote site becoming a “treat and transfer” center, with the aim of stabilising patients prior to transfer. There was a simultaneous introduction of a 24 hr outreach service (upscaling from the previous 12 hr/day service).

**Methods**

We conducted a retrospective study of all HO patients transferred to critical care from the 1st of January 2012 to the 30th of June 2016 to review the evolution of survival, critical care unit (CCU) length of stay (LOS) and pre-CCU admission characteristics as the time spent from referral to outreach on the ward and in the HDU/”treat and transfer” facility and severity of illness (as assessed by APACHE II).

**Results**

We found that over the period studied, the NEWS scores of patients referred to Critical Care Outreach did not change. However, patients spent more time on the ward before admission (from 6.1 hours in 2012 to 12.4 in 2016). Patients spent significantly less time in the Treat and transfer area than they had in the previous HDU, and on transfer to critical care, patients were less ill, with the APACHE II score decreasing from 21.1 in 2012 to 19.7 in 2016 (Table 18). There was a concomitant reduction in CCU LOS and an increase in CCU, hospital and 6 month survival (Table 19).

**Conclusions**

Introduction of a 24 hr outreach and “Treat and transfer” model increased support on the ward and allowed patients that were suitable for ward care to remain. However, it also enabled more rapid identification of patients that required critical care, resulting in earlier transfer and lower mortality.

**Table 18 (abstract P266).** See text for description

Year	Number	Referral NEWS (average)	Time on ward (hr)	Time on HDU/TnT (hr)	APACHE II	CCU LOS (days)
2012	31	4	6.1	29.8	21.2	12.4
2013	37	5	8.9	25.5	21.8	12.2
2014	26	5	9.4	13.1	20.9	11.0
2015	41	6	11.5	10.4	22.2	8.9
2016 (Jan to June)	20	5	12.4	8.1	19.7	8.0

Legend: Number of patients, CCU length of stay and pre-CCU admission characteristics

**Table 19 (abstract P266).** See text for description

Year	Number	CCU survival n(%)	Hospital survival n(%)	6 months survival n(%)
2012	31	16 (52)	15 (48)	10 (32)
2013	37	21 (57)	14 (38)	11 (30)
2014	26	18 (69)	13 (50)	13 (50)
2015	41	25 (61)	23 (56)	16 (39)
2016 (Jan to June)	20	15 (75)	11 (55)	10 (50)

Legend: CCU, hospital and 6 months survival.

## P267 Prevention of exposure keratopathy in critically ill patients: a randomized, prospective pilot study comparing ocular lubrication with bandage contact lenses

### I Bendavid, J Cohen, I Avisar, I Serov, I Kagan, P Singer

#### Rabin Medical Center, Petah Tikva, Israel

**Introduction**

Exposure keratopathy is a common disorder among critically ill patients (up to 60%) and may lead to bacterial keratitis and vision loss. We compared the effectiveness of bandage contact lenses compared to standard care with ocular lubricants in preventing exposure keratopathy.

**Methods**

A prospective randomized study was performed in a single mixed medical-surgical ICU on patients requiring mechanical ventilation and continuous sedation (as measured by the RASS score, -2 or lower to be included) for > 24 hours. Patients were randomized to eye care with either bandage contact lenses (n = 33) or ocular lubricants (n = 38). An ophthalmologist blinded to the assigned group assessed the degree of keratopathy, presence of chemosis and lagophthalmos every 4 days and at the time of withdrawal from the study in each eye. Patients were withdrawn from the study if they returned to consciousness, were discharged from the ICU, were weaned from mechanical ventilation or died. Lenses were changed every 4 days.

**Results**

The grade of keratopathy in the ocular lubricant group increased significantly in both eyes over the study period (p = 0.01 for both eyes) while no worsening was seen in the lens group. A subgroup analysis demonstrated significant improvement in patients randomized to the lens group with evidence of keratopathy at the initial examination (p = 0.02 and 0.018 in left and right eyes, resp); this effect was not evident in the ocular lubrication group. There were no significant between group differences in the degree of chemosis or lagophthalmos. No infections or other complications were noted in the lens group.

**Conclusions**

In this unique study we have shown that eye care with bandage contact lenses was more effective at preventing worsening of exposure keratopathy than ocular lubricants and was effective in healing eyes with initial corneal abrasions. No evidence of infection was noted. The lenses are easy to insert, provide continuous protection, may safely be left in situ for at least 4 days and may require less intensive surveillance than other measures.

## P268 Sleep helps healing: a quality improvement project

### J Hanison^1^, U Mirza^2^, D Conway^1^

#### ^1^Manchester Royal Infirmary, Manchester, United Kingdom; ^2^University of Manchester, Manchester, United Kingdom

**Introduction**

Delirium is a common occurrence in critical care units. It is estimated that approximately 45% of non-elective patients in UK critical care units experience delirium.[1] It is well known that there are a number of modifiable factors that disrupt patients’ sleep patterns when they are critically ill such as noise, light and patient care episodes. It has been demonstrated in US critical care units that the introduction of a multifaceted sleep promoting programme reduces rates on delirium.[2] This project attempted to introduce a multifaceted sleep promoting programme in a large UK critical care unit and assess whether rates of delirium could be reduced.

**Methods**

The project consisted of 2 time points of patient data collection with the quality improvement intervention in between these. Data was collected from 19 patients in the critical care unit prior to the intervention. Delirium was assessed using the CAM ICU score;[3] a positive score at any point during the study was recorded. The intervention consisted of local educational sessions, posters and reminders sent via email communication. After 6 weeks; data was collected from a further 47 patients.

**Results**

Delirium was present in 11/19 patients (58%) of patients prior to the intervention. Following the introduction of the sleep promoting quality improvement programme; the repeat audit found 11/47 (23%) of patients had delirium (chi squared P = 0.006).

**Conclusions**

This study has demonstrated that a relative reduction of 40% cases of delirium can be achieved with the introduction of a multifaceted sleep promoting programme in a UK critical care unit. This study demonstrates that such programmes are feasible and useful in the setting of a UK critical care unit.

**References**

[1] Page VJ, Navarange S, Gama S, et al. Routine delirium monitoring in a UK critical care unit. Crit Care. 2009 13(1):R16.

[2] Kamdar BB, King LM, Collop NA, et al. The effect of a quality improvement intervention on perceived sleep quality and cognition in a medical ICU. Crit Care Med. 2013 41(3):800-9.

[3] Ely EW, Margolin R, Francis J, et al. Evaluation of delirium in critically ill patients: validation of the Confusion Assessment Method for the Intensive Care Unit (CAM-ICU). Crit Care Med. 2001 29(7):1370-9.

## P269 Factors associated with early analgesic treatment for acute abdomen in emergency departments

### A Takasu^1^, H Tanaka^1^, N Otani^1^, S Ohde^2^, S Ishimatsu^1^

#### ^1^St.Luke´s International hospital, Tokyo, Japan; ^2^St. Luke´s International University Center for Clinical Epidemiology, Tokyo, Japan

**Introduction**

Treatment guidelines strongly recommend the earliest possible administration of analgesics for acute abdomen, regardless of cause, degree, or character. Nonetheless, delayed analgesic administration is often observed in clinical practice. This study aimed to examine the clinical use of analgesics for acute abdomen, as well as factors related to delayed administration.

**Methods**

We reviewed clinical records of patients brought to our hospital by ambulance, with acute abdomen as their chief complaint and having received an analgesic from July 2014 through June 2015. After excluding patients who were <16 years old, pregnant women, and patients with impaired consciousness, 279 patients were included in the analyses. The primary study outcome was time between hospital arrival and analgesic administration. Data were compared with regard to the following candidate factors: age, sex, body temperature, month of hospital visit, physician experience (1-2 years, 3-6 years, and 7- years), Numerical Rating Scale (NRS) score, and presence of localized tenderness. Univariate and multivariate Cox regression analyses were performed.

**Results**

The mean patient age was 44.2 years (SD;18.52), and 47.7% of the patients were men (n = 133). Men had a significantly shorter time to analgesic administration, compared to women (59.55 min vs. 90.28 min; HR: 1.709, 95% CI: 1.340-2.180). In addition, a shorter time to analgesic administration was observed for patients who were treated by more experienced physicians (1-2 years: 85.47 min, 3-6 years: 70.1 min, 7- years: 43.84 min; HR for 1-2 years vs. 3-6 years: 1.359, 95% CI: 1.040-1.775, HR for 1-2 years vs. 7- years: 2.376, 95% CI: 1.526-3.700).

**Conclusions**

Delayed analgesic administration for acute abdomen was associated with female patient sex and less physician experience. Therefore, it may be useful to encourage inexperienced physicians to follow the guideline recommendations regarding early analgesic administration, which may in turn lessen care discrepancies and improve patient satisfaction.

**References**

1) Toshihiko M, Masahiro Y et al. JPN guidelines for the management of acute abdomen 2015 Journal of Critical Care, 30(4),833.

2) Falch C, Vicente D, Haberle H et al. Treatment of acute abdominal pain in the emergency room. Eur J Pain 2014;18(7)902–913.

## P270 The duration of methoxyflurane analgesia in adult patients in the emergency department: a sub-analysis of stop! - a randomised, double-blind, placebo-controlled study

### F Coffey^1^, P Dissmann^2^, K Mirza^3^, M Lomax^4^

#### ^1^Nottingham University Hospitals NHS Trust, Nottingham, United Kingdom; ^2^James Cook University Hospital, Middlesbrough, United Kingdom; ^3^Colchester Hospital University Foundation NHS Trust, Colchester, United Kingdom; ^4^Mundipharma Research Limited, Cambridge, United Kingdom

**Introduction**

Acute pain is a frequent complaint in the Emergency Department (ED) [1] but remains widely undertreated [2]. Low-dose methoxyflurane, self-administered via a handheld inhaler (Penthrox®, 3 mL dose) is a fast-acting, non-narcotic analgesic agent. The STOP! study [3] investigated efficacy and safety of methoxyflurane analgesia for the treatment of acute pain in patients > =12 years presenting to the ED with minor trauma. Efficacy of methoxyflurane in the first 20 min after the start of treatment has been previously reported [3,4]. Here we describe the efficacy of methoxyflurane beyond this time point in adult patients.

**Methods**

In the STOP! study 300 patients were randomised in a 1:1 ratio to receive methoxyflurane (up to 6 mL) or placebo (normal saline), both via a Penthrox® inhaler. Study medication was self-administered by the patient as required by inhaling from the device. Rescue medication (paracetamol/opioids) was available immediately upon request. The primary efficacy endpoint was change in visual analogue scale (VAS) pain intensity from baseline up to 20 min after the start of study drug inhalation. We performed a further analysis of VAS pain intensity beyond 20 min.

**Results**

**Fig. 30 (abstract P270).**
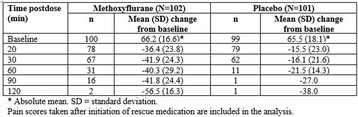
VAS Pain Intensity (0-100 Scale)

Data for adult patients (104 male, 99 female, mean age 36 years) are presented. Mean VAS pain intensity continued to decrease beyond 20 min after the start of treatment and was consistently lower for methoxyflurane-treated patients than placebo-treated patients (Fig. 30). The small sample size at later timepoints was due mainly to discharge of patients from the ED or start of a planned ED procedure. Treatment-related adverse events (mostly transient dizziness and headache) were reported by 42% of methoxyflurane patients and 15% of placebo patients; none caused withdrawal.

**Conclusions**

These results show that the previously reported early reduction in pain intensity with low-dose methoxyflurane administered via the Penthrox® inhaler is maintained for the duration of use during this study.

**References**

1. Cordell WH et al. Am J Emerg Med;20:165–9, 2002.

2. Pierik JGJ et al. Pain Med;16:970–84, 2015.

3. Coffey F et al. Emerg Med J;31:613–8, 2014.

4. Coffey F et al. Adv Ther;33:2012–31, 2016.

## P271 Global medication performance and safety of methoxyflurane analgesia in adult patients with contusions and lacerations treated in the emergency department

### P Dissmann^1^, F Coffey^2^, K Mirza^3^, M Lomax^4^

#### ^1^Emergency Department, James Cook University Hospital, Middlesbrough, United Kingdom; ^2^Emergency Department, Nottingham University Hospitals NHS Trust, Nottingham, United Kingdom; ^3^Accident and Emergency Department, Colchester Hospital University Foundation NHS Trust, Colchester, United Kingdom; ^4^Mundipharma Research Limited, Cambridge, United Kingdom

**Introduction**

Low-dose methoxyflurane administered via a handheld inhaler (Penthrox®) has been used for over 30 years in Australia and New Zealand for short-term pain relief in emergency medicine. The STOP! Study [1,2] was a double-blind placebo-controlled UK study that investigated the efficacy and safety of methoxyflurane analgesia for the treatment of acute pain in adult and adolescent patients presenting to the ED with minor trauma. We present a sub-analysis of global medication performance (GMP) and adverse events (AEs) in adult patients with contusions or lacerations.

**Methods**

300 patients were randomised in a 1:1 ratio to receive methoxyflurane (up to 6 mL) or placebo (normal saline), both inhaled as required via a Penthrox® inhaler. Rescue medication (paracetamol/opioids) was available immediately upon request. The patient, treating physician and research nurse each rated GMP at ED discharge as ‘Poor’, ‘Fair’, ‘Good’, ‘Very Good’, or ‘Excellent’. AEs were recorded from enrolment until discharge, and at safety follow up (Day 14 ± 2).

**Results**

**Fig. 31 (abstract P271).**
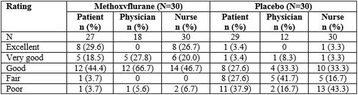
Global Medication Performance

GMP and safety data for adult patients with contusions or lacerations (N = 60; 29 male, 31 female, aged 18-68 years) are presented in Fig. 31. 92.5% of patients rated methoxyflurane excellent, very good or good compared to 34.4% for placebo. The GMP ratings by the patient, physician and nurse at ED discharge were all significantly better in the methoxyflurane group compared with the placebo group (p < 0.0001 for patient and nurse ratings and p = 0.0186 for physician rating).

AEs were reported by 73% of methoxyflurane patients (47% for related AEs) and 33% of placebo patients (10% for related AEs) and were mainly mild and transient in nature. AEs reported by > =3 methoxyflurane patients were dizziness (methoxyflurane: 11, placebo: 2), headache (methoxyflurane: 4, placebo: 1) and somnolence (methoxyflurane: 3, placebo: 4). One unrelated serious AE (lower respiratory tract infection) occurred in the methoxyflurane group and 1 patient in the placebo group withdrew due to an AE (vomiting).

**Conclusions**

A high degree of patient and treating physician/nurse satisfaction (> = 93%) with low-dose methoxyflurane treatment administered via the Penthrox® inhaler was recorded for patients presenting to the ED with contusions or lacerations.

**References**

1. Coffey F et al. Emerg Med J;31:613–8, 2014.

2. Coffey F et al. Adv Ther;33:2012–31, 2016.

## P272 Safety and efficacy of sufentanil sublingual tablet 30 mcg for management of acute traumatic pain

### JR Miner^1^, R Leto^2^

#### ^1^Hennepin County Medical Center, Minneapolis, MN, United States; ^2^Derriford hospital PHNT, Plymouth, United Kingdom

**Introduction**

Pain is the most common reason people visit the emergency department (ED). A review of ED pain management practices however demonstrates treatment inconsistency and inadequacy. Many patients still suffer from acute pain while waiting to be evaluated, waiting for intravenous (IV) access to be established and/or waiting for treatment or procedures.1 A rapid-acting, potent analgesic that does not require an IV may facilitate early pain control in the ED. A phase 3 trial using sufentanil sublingual tablet 30 mcg (SST 30 mcg) for treatment of undifferentiated acute pain in the ED was recently completed. The primary objective of the study was to evaluate the safety and efficacy of SST 30 mcg in this setting.

**Methods**

This was a multi-center, open label study for up to 5 hours in adult patients presenting to the ED with acute pain brought on by recent trauma or injury. Patients were administered up to four doses of SST 30 mcg, given at least 60 minutes apart. Efficacy was assessed by patient reports of pain intensity on an 11-point numerical rating scale (0 = no pain and 10 = worst possible pain), where each reduction of 1.3 is considered clinically meaningful to the patient.2 Primary efficacy variable was the summed pain intensity difference to baseline over the first hour (SPID1). Safety was assessed via vital signs and adverse event (AE) reporting.

**Results**

Overall, 76 patients were enrolled; 40 were offered a single dose of SST 30 mcg (single-dose cohort) while 36 were permitted additional doses, if needed (multi-dose cohort). The mean baseline pain intensity for all patients was 8.1 and following a single dose of SST 30 mcg, pain intensity scores were reduced by over 35%, to a mean of 5.2. Only 9 of the 36 multi-dose cohort patients required or requested more than one dose. Mean pain intensity for all patients was significantly lower at 15 minutes following initiation of SST 30 mcg (p < 0.001). 79% of patients reported no adverse events. The majority (97%) of AEs were mild with nausea (7 patients; 9%) and vomiting (3 patients; 4%) the most commonly reported.

**Conclusions**

SST 30 mcg was well tolerated and effective in managing moderate-to-severe acute pain in an ED setting and may provide a viable analgesic option for opioid-naive trauma patients.

**References**

1. Todd KH et al. Pain in the emergency department: results of the pain and emergency medicine initiative (PEMI) multicenter study. J Pain 2007;8:460–6.

2. Bijur PE et al. Validation of a verbally administered numerical rating scale of acute pain for use in the emergency department. Acad Emerg Med 2003;10:390–2.

## P273 Sevoflurane does not change intracranial pressure in neurocritical patients: a case series

### AM Markota^1^, PG Gradišek^2^, VA Aleksejev^1^, AS Sinkovič^1^

#### ^1^University Medical Centre Maribor, Maribor, Slovenia; ^2^University Medical Centre Ljubljana, Ljubljana, Slovenia

**Introduction**

Treatment with sevoflurane was associated with uncontrollable increases in intracranial pressure (ICP) in 32% of patients with subarachnoid hemorrhage or stroke [1]. The aim of this study was to evaluate changes in ICP in our case series of neurocritical patients.

**Methods**

A retrospective analysis performed between November 2015 and November 2016 of mechanically ventilated patients with acute brain injury and inserted ICP probe that received sevoflurane treatment.

**Results**

**Fig. 32 (abstract P273).**
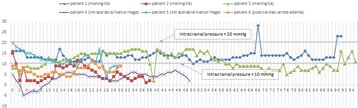
Intracranial pressure changes over time during the treatment with sevoflurane

Overall 6 patients were included: 4 females (26, 36, 58 and 83 years old) and 2 males (55 and 67 years old). Admission diagnosis were: meningitis in 3 patients, intracerebral hemorrhage in 2 and cerebral edema after cardiac arrest in 1 patient. Duration of concomitant treatment with sevoflurane and ICP monitoring was 30, 77, 38, 51, 95 and 30 h, respectively. ICP was > =20 mmHg in 1.4% of measurements (23, 23 and 28 mmHg), > = 11 and < =19 mmHg in 42.8%, and < =10 mmHg in 55.7% (Fig. 32). Target mean alveolar concentration of sevoflurane was 1-1.5% in 1 patient, and 0.5-1% in all other patients. During the treatment with sevoflurane, 2 patients received a total of 4 boluses of mannitol, normocapnia was maintained in all patients, normothermia was targeted in 5 patients, and therapeutic hypothermia was used in one.

**Conclusions**

In our case series of neurocritical patients sevoflurane was not associated with increases of ICP.

**Reference**

[1] Purrucker JC et al. Br J Anaesth 114: 934–43, 2015

## P274 Critical care sedation with sevoflurane: a single center experience with the new mirus system

### S Romagnoli, C Chelazzi, G Zagli, F Benvenuti, P Mancinelli, P Boninsegni, L Paparella

#### Osp. Azienda Ospedaliero Univ Careggi, Florence, Italy

**Introduction**

Volatile anesthetics (VA) are considered alternative strategies to standard intravenous agents for critically ill patients requiring moderate-to-deep sedation [1]. We performed a prospective interventional study to evaluate the feasibility, efficacy, and adverse events related to inhaled sevoflurane (Sevo) delivered with the MIRUS system (Pall International Sarl, Fribourg, Switzerland,) and to evaluate the atmospheric pollution.

**Methods**

Patients requiring sedation after general abdominal surgery and in ICU were included in the study. Sedation was targeted to a RASS scale of -3/-5. The following data were collected: RASS, MAC, Fi-Sevo, Fe-Sevo, RR, Vt, minute ventilation, PaO2/FiO2, PaCO2, wake-up time, duration of sedation, Sevo consumption, CO (MostCare, Vygon, Italy), HR. Atmospheric pollution was measured (Sevo,ppm) at 30-sec intervals: 1) baseline measure (B): before Sevo delivery, with MIRUS with the probe placed 15 cm up to the system; 2) during sedation (S1) with the probe placed 15 cm up to the system; 3) during sedation (S2) with the probe placed 15 cm from the filter-reflector group. Statistical analysis: The Shapiro-Wilk test was used to test all data for normality. The paired t-test, the Wilcoxon signed rank and the McNemar´s test were used as appropriate.

**Results**

A total of 46 patients were enrolled. No technical failure occurred. MAC resulted 0.41 (0.14)%, Fi-Sevo and Fe-Sevo were 0.68 [0.55-0.91]% and 0.68 [0.6-0.87] respectively; Vt and RR were 582 [550-705]ml and 12.30 [11-16.48]b/m respectively; minute ventilation was 8.61 [6.81-9.68]L/min; PaO2/FiO2 and PaCO2 were 330.2 [288-388] and 39.1 [36-49]mmHg respectively. Awakening time was 4 [2-5]min. Total VA administration was 4.8 [2.4-11.36]h, range (1-22 h) with a consumption of 9.1 (5.1)ml/h. Hemodynamics did not show a difference between arrival in the ICU and 1 hr after Sevo sedation. No adverse events were registered. Ambient pollution showed the following levels: B: 0.10 [0.07-0.15], S1: 0.17 [0.14-0.27], S2:0.15 [0.07-0.19]ppm.

**Conclusions**

Sevo-sedation with MIRUS is feasible and safe. Atmospheric pollution remained largely below the indicated thresholds [2]. All the patients were efficaciously sedated at the targeted levels.

**References**

[1] Baron R, et al. Evidence and consensus based guideline for the management of delirium, analgesia, and sedation in intensive care medicine. Ger Med Sci. 12;13, 2016

[2] Molina Aragonés JM, et al. Occupational exposure to volatile anaesthetics: A systematic review. Occupational Medicine 66:202–207, 2016

## P275 The anaconda® and sevoflurane: data on ambient pollution and staff exposure

### AT Bos, O Thomas

#### VieCuri MC Venlo, Venlo, Netherlands

**Introduction**

The AnaConDa® is a reflection filter used to administer volatile anesthetics to intubated patients. Little data are available on staff exposure and ambient pollution when using the device. A risk analyses was performed as requested by our occupational health service. Aim was to quantify ambient pollution and staff exposure to sevoflurane and relate outcome to current safety standards.

**Methods**

Air sampling was performed by an independent institute. A Miran SapphIRe ambient analyzer was used for realtime measurements on 8 connecting points of the breathing circuit, AnaConDa® and FlurAbsorb canister. Charcoal sorbent tubes were used for 4 seperate 8-hour stationary and 2 personal 8-hour measurements on consecutive shifts. Samples were analyzed in a laboratory. Swedish occupational exposure guidelines were used as a reference; no national guidelines are available. Exposure limit is 5 ppm (time weighted average (TWA) 42 mg/m3 over an 8-hour period). North American guidelines are stricter, with a maximum of 2 ppm. The UK is more liberal, allowing limits up to 50 ppm.

**Results**

**Fig. 33 (abstract P275).**
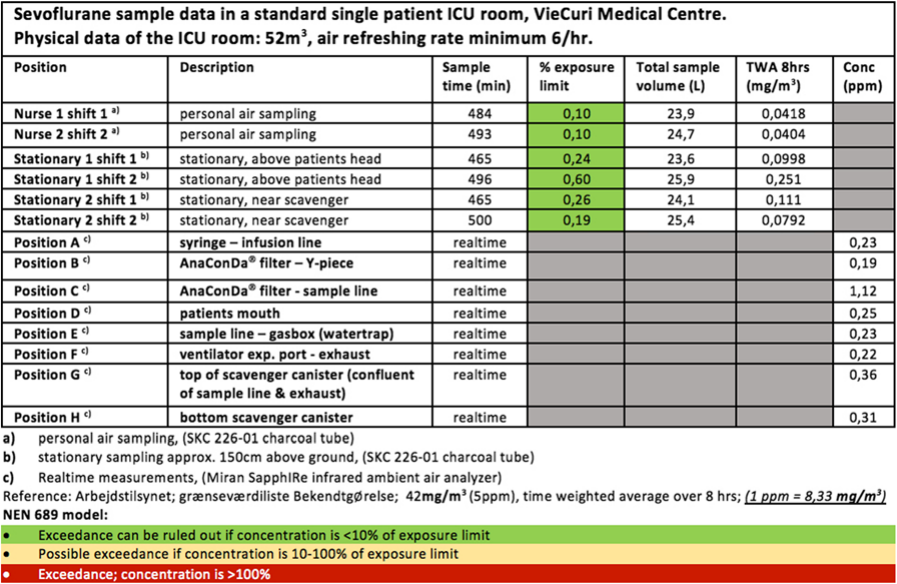
Sevoflurane sample data in a standard single patient ICU room, VieCuri Medical Centre. Physical data of the ICU room: 52 m3, air refreshing rate minimum 6/hr

TWA is expressed as a percentage of exposure limits (Fig. 33). Exceeding is ruled out if concentrations are <10% of exposure limits (NEN mod.). We show that peak concentrations and TWA are below the occupational exposure limits.

**Conclusions**

Sedation with sevoflurane and the AnaConDa® system is safe in an ICU. Exposure is negligible and meets international standards. Our data highly contribute to staff safety perception and awareness and are helpful to those who want to implement the AnaConDa® in the ICU.

**Reference**

Crit Care Med 2005(33):585–90

## P276 Does anaconda scavenge iNO?

### T Goslar, R Knafelj

#### Rihard Knafelj, Ljubljana, Slovenia

**Introduction**

Nitric oxide (NO) is used in many centers as a rescue therapy in refractory hypoxemia. ICU sedation with sevoflurane using Anaconda is gaining wide acceptance. NO and volatiles use require scavengers. In our ICU volatile sedation is standard of care. We recently reported no interference of sevoflurane and NO on measurements any of those agents. Potential NO scavenging properties of Anaconda is unknown.

**Methods**

In 22 consecutive ventilated patients with ARI, receiving NO and sevoflurane, NO concentration was measured at different parts of breathing circuit. All patients were mechanically ventilated (Elisa 800, HL) at constant minute ventilation, NO and sevoflurane concentration. NO was delivered and concentration measured with NOx (CareFussion) and sevoflurane via Anaconda. Additionally sevoflurane scavenger (FlurAbsorb) was evaluated for NO scavenging properties

**Results**

NO concentration drop across Anaconda was 49,9 ± 7,9% (range 40 - 64%) independently of NO concentration (9 - 40 ppm), achieving 4 - 24 ppm at Y piece. In tested range of different NO concentrations (9 - 40 ppm), no NO or NO2 could be detected at FlurAbsorb expiratory orifice. NO/NO2 scavenger does not scavenge sevoflurane at 2 vol% tested.

**Conclusions**

Measuring NO concentration before Anaconda gives falsely higher NO values, as approximately 50% of NO is scavenged by Anaconda. Actual concentration of NO should be measured at Y piece. Scavenging properties of Anaconda did not differ across tested NO concentrations. FlurAbsorb is highly efficient sevoflurane, NO and NO2 scavenger, offering additional savings by eliminating NO/NO2 scavengers. NO/NO2 scavengers do not scavenge sevoflurane and should not be used as sole scavangers when NO and sevoflurane are used.

**Reference**

R. Knafelj, P. Kordis. Co-administration of nitric oxide and sevoflurane using anaconda. P374 Critical Care201620(Suppl 2):94

**Fig. 34 (abstract P276).**
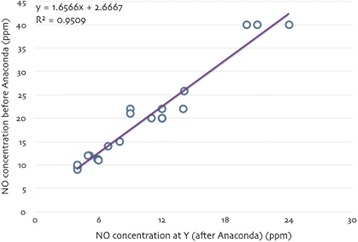
See text for description

**Fig. 35 (abstract P276).**
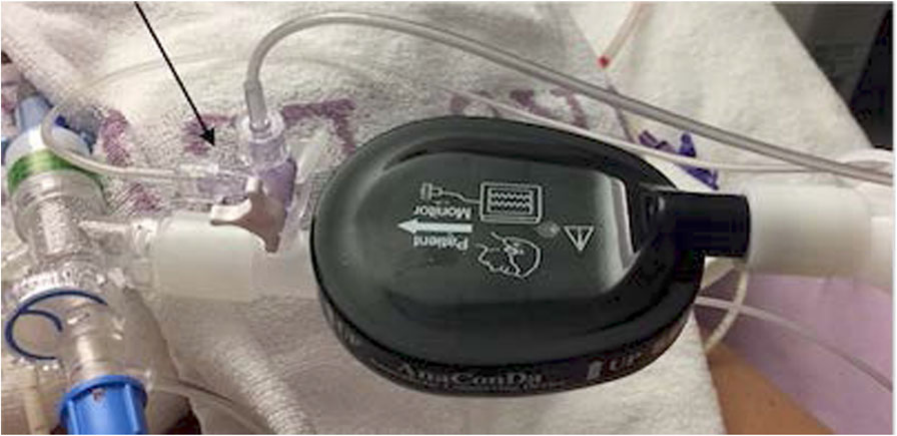
See text for description

## P277 Withdrawal assessment in adult ICU patients: validation of the WAT-1 scale

### M Perreault^1^, A Martone^1^, PR Sandu^1^, VA Rosu^2^, A Capilnean^1^, P Murgoi^1^, AJ Frenette^3^, A Lecavalier^1^, D Jayaraman^1^, P Rico^3^, P Bellemare^3^, C Gelinas^4^, D Williamson^3^

#### ^1^The Montreal General Hospital, Montreal, Canada; ^2^Hôpital de Verdun, Verdun, Canada; ^3^Hopital du Sacre-Coeur, Montreal, Canada; ^4^Jewish General Hospital, Montreal, Canada

**Introduction**

The use of the WAT-1 (Withdrawal Assessment Tool) scale for the assessment of opioid withdrawal syndrome in critically ill pediatric patients on mechanical ventilation has been validated and has gained significant clinical acceptance. Little is known about this syndrome in critically ill adults. Such bedside tool has the potential to increase recognition of this condition and lead to preventive therapy. The aim of our study was to evaluate the validity and reliability of the WAT-1 tool in the critically ill adult.

**Methods**

A prospective observational open cohort study was conducted in critically ill adults (> = 18yold) on mechanical ventilation and on regular opioids for at least 72 hours from two Canadian critical care trauma centers over an 8 month enrollment. Patients underwent withdrawal assessments twice daily on weekdays and once daily on weekends using the WAT-1 scale once a weaning episode of opioids was identified (i.e. dose reduction by at least 10% and sustained over 4 hours for continuous infusions or over 12 hours for intermittent opioid administration). Intensivists or ICU fellows evaluated the presence of opioid withdrawal once daily using a modified DSM-V criteria. All evaluations were blinded and performed independently. Criterion validity of the WAT-1 was calculated from 2x2 frequency tables using standard definitions of sensitivity and specificity and overall accuracy. Interrater reliability for WAT-1 and DSM-V evaluations (performed once at random per patient) were assessed with the kappa coefficient.

**Results**

During an 8 month period, 54 adults (74.1% male and 81.5% caucasian) with a median age of 50 years and APACHE II score of 22 were enrolled. Median ICU length of stay was 17.5 days. Two patients were excluded from the analysis as no paired WAT-1 and DSM-V evaluations were performed. A total of 212 withdrawal assessments occurred (median of 4 assessments per patient). Eight patients (15.4%) had at least one positive DSM-V assessment during their ICU stay compared to 19 patients (36.5%) having at least one WAT-1 scale positive. The overall sensitivity of the WAT-1 was 50% and its specificity was 65.9%. The interrater agreement between the WAT-1 and DSM-V evaluations was measured with a Kappa of 0.102.

**Conclusions**

The use of the WAT-1 tool to assess presence of opioid withdrawal is not valid nor reliable in the critically ill adult on mechanical ventilation.

## P278 Impact of early deep sedation on duration of mechanical ventilation in psychiatric patients requiring intensive care: a retrospective analysis

### T Nishida, T Kinoshita, N Iwata, K Yamakawa, S Fujimi

#### Osaka General Medical Center, Osaka, Japan

**Introduction**

Several studies have demonstrated that early deep sedation (within 48 hours after initiation of mechanical ventilation) is associated with delayed extubation and longer hospital length of stay. However, the efficacy of early light sedation in psychiatric patients is still unclear since psychiatric patients are excluded from analysis in the most of these studies. The purpose of this study is to evaluate the relationships between early sedation depth and clinical outcomes among ventilated psychiatric patients in the intensive care unit.

**Methods**

We retrospectively assessed consecutive adult psychiatric patients who were sedated and ventilated for 48 hours or more in our intensive care unit (ICU), from January 2013 to October 2016. Patients were excluded if they were <18 years, had neurological impairment, had burns, or further aggressive treatment was not preferred. We categorized all patients into deeply sedated group (Richmond Agitation Sedation Scale [RASS] -5 to -3) or lightly sedated group (RASS -2 or more). We set primary endpoint as duration of mechanical ventilation dichotomized into short (3 to 6 days) or long (7 days or more). Secondary endpoints included ICU length of stay, the occurrence of delirium, and the occurrence of accidental extubation. Age, APACHE-II score, admission diagnosis, and types of psychiatric disease were adjusted by using multivariable logistic regression analysis.

**Results**

Among 109 included patients, 50 (46%) underwent deep sedation and 59 (54%) underwent light sedation. Patients who were lightly sedated had shorter duration of mechanical ventilation (5 vs 8, p = 0.002) and ICU length of stay (6 vs 10, p = 0.008). After adjusting for baseline characteristics, light sedation was significantly associated with lower incidence of long duration of mechanical ventilation (adjusted odds ratio [OR], 0.27; 95% confidential interval [CI], 0.09-0.70; p = 0.009). There were no significant differences in the occurrence of delirium (adjusted OR, 0.53; 95% CI, 0.17-1.60; p = 0.262) and accidental extubation (adjusted OR, 0.85; 95% CI, 0.01-67.6; p = 0.906) between the two groups.

**Conclusions**

Early light sedation in patients with psychiatric illnesses was associated with shorter mechanical ventilation without increasing risk of delirium and accidental extubation.

## P279 Impact of sedation on diaphragm thickness in ICU patients: preliminary results

### L Maggi^1^, F Sposato^2^, G Citterio^1^, C Bonarrigo^2^, M Rocco^2^, V Zani^2^, RA De Blasi^2^

#### ^1^Campus Biomedico, Rome, Italy; ^2^Azienda Ospedaliera Sant´Andrea, Università La Sapienza, Rome, Italy

**Introduction**

The aim of our study was to compare the in vivo effects of Propofol and Dexmedethomidine infusion on diaphragm thickness by ultrasound. Few studies investigated the effects of sedation on diaphragm in healthy volunteers but no studies evidenced how sedation affects diaphragm contraction in patients with a systemic inflammation. Only experimental data showed an opposing effect of Propofol and Dexmedethomidine on skeletal muscle during sepsis (1).

**Methods**

We enrolled 19 consecutive patients (12 treated with Propofol, group P, and 7 with Dexmedethomidine, group D) admitted in ICU who needed mechanical ventilation for at least 48 h. All patients received a Remifentanil infusion for pain control. Sedation was set by clinicians to achieve a RASS scale between -2 and 0. Sonography was performed on the zone of apposition of diaphragm on the rib cage with a linear 10 MHz probe (2). Diaphragm thickness was assessed at end inspiration (TEI) and end expiration (TEE). Time-point of examination was at ICU admission (T0), 24 h after admission (T1) and after 48 h (T2). Data were compared with the Student-T test and comparison between times was performed using ANOVA and post-hoc analysis with Tukey test using Sigma Plot.

**Results**

**Fig. 36 (abstract P279).**
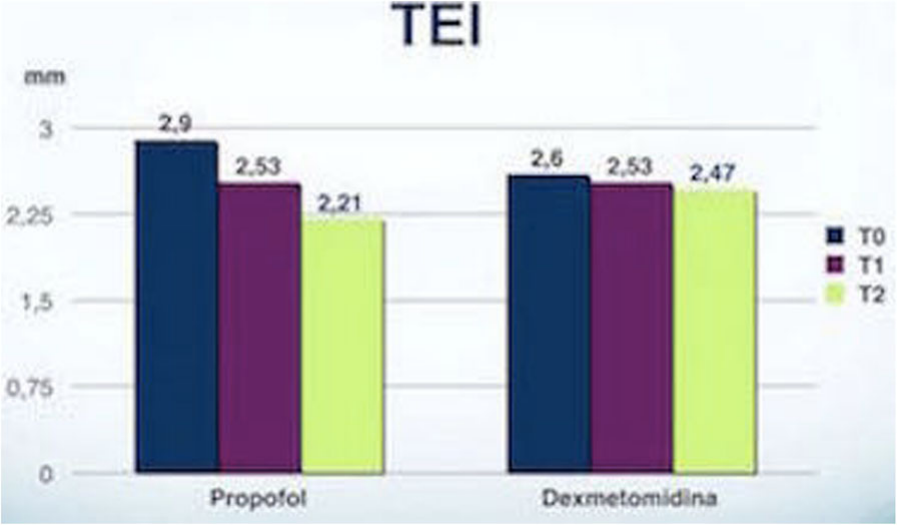
TEI variation

Diaphragm thickness underwent an overall progressive reduction in the P group (p = 0.039). Particularly, TEI showed a reduction between T0 and T2 only in the P group (p <0.05), (Fig. 36). On the contrary, no reduction was observed for the TEE during the time. The P group showed a significant reduction T2 in TEI compared to the D group (p = 0.03).

**Conclusions**

From our preliminary results Dexmedethomidine could have a protective role on diaphragmatic contraction in sedated and mechanically ventilated ICU patients. Data needs to be confirmed with a larger, randomized trial.

**References**

1. Pandharipande PP et al. :Crit Care. 2010;14(2):R38

2. Matamis D et al: Intensive Care Med 2013:801–810

## P280

### Withdrawn

## P281 The real cost of sugammadex: from patient to society

### D Alcorn, L Barry

#### Royal Alexandra Hospital, Paisley, United Kingdom

**Introduction**

Sugammadex has been available for use in Scotland since 2008, and since then it has been subject to much discussion. Although with the potential to change modern anaesthetic practice, this first ever y-cyclodextrin has an associated financial cost in comparison to conventional treatments. Currently, it is accepted for restricted use within NHS Scotland for the routine reversal of neuromuscular blockade and for restricted use for high risk patients. (1)

**Methods**

We carried out a paper-based audit across Greater Glasgow and Clyde incorporating a total of eight hospitals over a three-month period. We asked questions seeking information regarding reasons for use. We also looked at admission to HDU (high dependency unit) and ICU (intensive care unit) and whether this was a planned or unplanned admission.

**Results**

In total, 1263 200 mg vials of sugammadex were documented as being used over the three month period. We received data for 764. (Compliance rate 59%). Obesity was the most common co-morbidity (35.4%), followed by ischaemic heart disease (14.6%), followed closely by COPD.(13%). 50.7% of patients had a BMI great than 30 and 44.2% had an ASA 2. Inadequate reversal was reported as the main reason for use in 27% of cases, but interestingly 35% documented planned usage of the drug. 29.5% of the cases in question were regarded as emergency cases. Overall, there were no failed extubations. 105 patients were admitted to level 2 care; only 5% of these were unplanned admissions.

**Conclusions**

Obesity is the main cause of use and as each vial of sugammadex costs approximately £60 in comparison to £2 for a conventional treatment, this raises the concern of our growing population. Obesity in critically ill patients is associated with an increased morbidity and mortality and a prolonged ICU stay. The annual cost to the NHS of overweight and obese patients is estimated at £600 million (2) Our study demonstrated a small percentage of unplanned admissions to a critical care setting. However, further studies are required to review if the number of post-operative complications and admissions to level 2 and 3 care are reduced by sugammadex.

**References**

1. Sugammadex 100 mg/ml solution for injection. SMC (527/09). Merch, Sharp and Dohme Limited. Scottish Medicines Consortium. 08 February 2013.

2. SPICe Briefing: Obesity in Scotland.07 January 2015.

## P282 Effects of propofol on the microcirculation in children with continuous video microscopy imaging

### M.A. Riedijk^1^, D.M. Milstein^2^

#### ^1^Academic Medical Center, Amsterdam, Netherlands; ^2^Academic Medical Center, Oral & Maxillofacial Surgery, Amsterdam, Netherlands

**Introduction**

Procedural sedation is widely used in the pediatric population to facilitate painful or potentially unpleasant procedures. Effective anxiolysis and sedation improve the experience for the pediatric patient, increase chances of success. Propofol is the ideal sedative agent; it has a rapid onset of action inducing unconsciousness within minutes of intravenous administration (1). A well-known side effect of propofol is a decrease of peripheral vascular resistance resulting in hypotension. In contrast to systemic hemodynamic effects, little is known about the effects of propofol on the microcirculation in humans. Only one study in healthy adult females was published and showed that propofol reduces microcirculatory perfusion (2). Measurements were obtained intermittently by orthogonal polarization spectral imaging. However, it is not possible to find the exact same region of interest by intermittent imaging. The aim of this study was to evaluate the effects of propofol on the microcirculation in relatively healthy children by continuous video microscopy imaging.

**Methods**

In a single-center observational study children between 8-18y who were admitted to the Pediatric Intensive Care Unit of the Emma Children’s Hospital Amsterdam for procedural sedation were enrolled for participation. Continuous monitoring of the sublingual microcirculation was performed by incident dark-field illumination imaging. Video clips were saved before and three min after induction with propofol. Microcirculatory parameters were measured and the differences between before and three min after induction were calculated.

**Results**

In 6 patients (15 ± 3y) mean induction dose of propofol was 2.3 ± 0.3 mg/kg. Macrocirculation parameters are presented in Table 20. Mean arterial pressure (MAP) was significantly decreased after induction with propofol (p < 0.05). Total and perfused vessel densities for all vessels increased by 11% and 18% respectively (p < 0.05). Microvascular flow index was unaltered.

**Conclusions**

Induction with propofol induces a marked reduction in MAP and a rise in sublingual microvascular perfusion during pediatric procedural sedations. The observed effects of propofol on the microcirculation may be due to a decrease in microvascular resistance.

**References**

1. Langley MS, Heel RC. Propofol. A review of its pharmacodynamic and pharmacokinetic properties and use as an intravenous anaesthetic. Drugs 35:334–372,1988

2. Koch M, et al. Effect of propofol on human microcirculation. British Journal of Anaesthesia 101:473–478,2008

**Table 20 (abstract P282).** See text for description

Induction propofol	T0	T3
Systolic pressure mmHg	119 ± 27	91 ± 14*
MAP mmHg	80 ± 22	57 ± 13*
Heartrate bmp	78 ± 15	75 ± 10

Legend: Basic hemodynamic parameters mean ± SD, *p < 0.05

## P283 Dynamic cerebral autoregulation: a marker of post-operative delirium?

### J Caldas^1^, R Panerai^2^, L Camara^1^, G Ferreira^1^, E Bor-Seng-Shu^1^, M Lima^1^, F Galas^1^, N Mian^1^, R Nogueira^1^, G Queiroz de Oliveira^1^, J Almeida^1^, J Jardim^1^, TG Robinson^2^, F Gaioto^1^, LA Hajjar^1^

#### ^1^University of Sao Paulo, Sao Paulo, Brazil; ^2^University of Leicester, Leicester, United Kingdom

**Introduction**

Post-operative delirium, a frequent complication of cardiac surgery with cardiopulmonary bypass, has been associated with poor outcome in critically ill patients.

**Methods**

A observational, prospective, single center study was performed. Adults with EuroSCORE ¡Ý 6 or left ventricular ejection fraction < 40% undergoing coronary artery bypass graft surgery with cardiopulmonary bypass were included. Cerebral blood flow velocity (transcranial Doppler) and blood pressure (Finometer or intra-arterial line) were continuously recorded during 5 minutes preoperatively (T1), after 24 h (T2) and 7 days after surgery (T3). Autoregulation index (ARI) was estimated from the cerebral blood flow response to a step change in blood pressure derived by transfer function analysis. Impaired dynamic CA was defined as ARI < 4. Diagnosis of a post-operative delirium was performed using the confusion assessment method for ICU (CAM-ICU).

**Results**

Sixty-seven patients (51 male), mean age 64.3¡À9.5 years, were studied. Cerebral blood flow velocity step responses at T2 were markedly different from T1 and T3 with corresponding values of ARI reduced at T2 (3.9 ¡À 1.7), compared to T1 (5.6 ¡À 1.7) and T3 (5.5 ¡À 1.8) (p < 0.001). Impaired CA was found in 37 (55%) patients at T2 and in 7 patients (20%) at T3. PD was diagnosed in 17 patients; of these, 13 (76.5%) had impaired CA, compared to 4 (23.5%) with normal CA (p = 0.041). At T3, 52.9% of the patients with impaired CA presented with PD compared to only 10% of those with normal CA (p = 0.001). Lower ARI at T1 and T2 were predictors of PD (p = 0.003).

**Conclusions**

Dynamic CA is impaired after coronary artery bypass graft surgery with cardiopulmonary bypass and was associated with post-operative delirium.

**References**

Brown CH: Delirium in the cardiac surgical ICU.Curr Opin Anaesthesiol 2014, 27(2):117–122

**Table 21 (abtsract P283).** See text for description

Variable	Parameter estimated	Standard error	P
MoCA T1	-0.158	0.069	0.022
ARI T1	-0.377	0.178	0.034
Constant	4.097		
MoCA T2	-0.241	0.085	0.005
ARI T2	-0.068	0.023	0.003
CBFV t2	-0.707	0.269	0.009
Constant	11.128		

Legend: Multivariate logistic regression analysis

**Fig. 37 (abstract P283).**
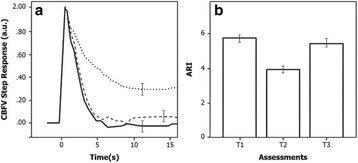
CBFV Step Response in T1, T2 and T3

## P284 Prediction of delirium after major abdominal surgery

### I Zabolotskikh, T Musaeva

#### Kuban State Medical University, Krasnodar, Russia

**Introduction**

Based on behavioral and motoric characteristics, existence of two different types of delirium was confirmed: hyperalert/hyperactive and hypoalert/hypoactive. In the critically ill patients the hypoactive delirium is the most common – 45-65% and is often overlooked. CAM-ICU and ICDSC scales have not demonstrated the agreement in diagnosing delirium [1]. Registration of the direct-current potential (DCP) in a forehead-palm lead [2] allow the clinicians to identify three different functional state of the human body. The purpose of the study is to determine relationship between direct current potential level and the type and the incidence of delirium.

**Methods**

A retrospective study of the perioperative period after major abdominal operations in 690 patients was performed. The physical condition of patients corresponded to 3 class of ASA. The median age was 46.0 (38,0-62,0) years. The duration of the operations was more than 180 minutes. All patients received standard postoperative management and were divided into 3 groups according to the level of direct current potential (DCP) that was measured in first 6 hours of postoperative period: low negative level of DCP (< - 14 mV) (n = 216), average level of DCP (-15 - (-29) mV) (n = 224), high level of DCP (> -30 mV) (n = 250). The measurement was conducted in a continuous recording for 10 minutes from the active electrode located in the middle of the forehead, and the reference electrode - in the tenor region. For statistical analysis AUROC (Area Under Receiver Operator Curve) was performed.

**Results**

Overall delirium incidence was 40%. The maximum frequency of postoperative delirium (74.0%) was observed at low negative values of DCP (< -14 mV) with AUROC - 0.89 (95% CI 0.86–0.92) for incident delirium; 0.81 (0.74–0.85), for prevalent (hypoactive) delirium. At high level of DCP (> -30 mV) postoperative delirium was observed in 31,2%; AUROC was 0.82 (95% CI 0.79–0.86) for incident delirium; 0.89 (0.82–0.93), for prevalent (hyperactive) delirium. At average level of DCP (-15 - (-29) mV) postoperative delirium was observed in 17,0%; AUROC was 0.73 (95% CI 0.71–0.77) for incident delirium; 0.68 (0.63–0.71), for prevalent (hyperactive) delirium.

**Conclusions**

The DCP monitoring allows to predict the incidence and type of delirium in patients with low negative and high level of direct current potential registered in early postoperative period.

**References**

1. Tomasi C. Journal of Critical Care 27(2): 212–217, 2012

2. Zabolotskikh IB. European Journal of Anaesthesiology 32(53):260, 2015

## P285 Frequency of low minute ventilation events as indication of post-operative respiratory depression

### W Saasouh^1^, J Freeman^2^, A Turan^1^

#### ^1^Cleveland Clinic Foundation, Cleveland, OH, United States; ^2^Respiratory Motion, Inc, Waltham, MA, United States

**Introduction**

Opioids manage postoperative pain but can cause opioid-induced respiratory depression (OIRD). Early identification of patients at-risk for OIRD after surgery could improve safety, allowing time for modification of opioid dosing or additional monitoring. We evaluated the ability of a non-invasive respiratory volume monitor (RVM, ExSpiron, Respiratory Motion, Waltham, MA) to identify patients at-risk for OIRD by monitoring them in the early post-operative period.

**Methods**

RVM data (minute ventilation (MV), tidal volume, and respiratory rate) were collected from 102 patients (53 males, BMI:27.4(15.0-41.1)kg/m2) for 40.9 ± 1.2 hr following abdominal surgery. Predicted MV (MVPRED) was calculated by body surface area. A Low MV event (LMVe) was defined as MV < 40%MVPRED sustained for > =2 min. Patients were grouped by LMVe Rate (A:LMVe Rate < 1/hr, B:1-3/hr, C:> = 3/hr).

**Results**

**Fig. 38 (abstract P285).**
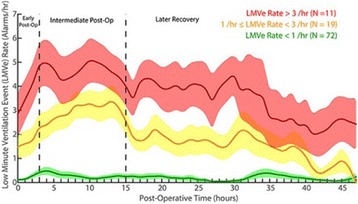
Mean (Solid lines) ± SEM (shaded regions) LMVe Rate as a function of time following PACU admission. Patients were grouped based on LMVe Rate **a**: LMVe Rate <1/hr (*red*), **b**: LMVe Rate between 1 & 3/hr (*yellow*), **c**: LMVe Rate ≥ 3/hr (*red*)). In all groups, Early Post-Op LMVe Rate was highly correlated with rate of LMVe in Later Recovery. For Groups B & C, the LMVe Rate was higher in the Intermediate Post-Op (**b**: 2.8/hr, **c**:4.7/hr) compared to the Early Post-Op (**b**: 1.6/hr, **c**: 3.3/hr) & Later Recovery (**b**: 1.2/hr, **c**: 3.3/hr)

25% of patients had no LMVes and 72% of monitored hours were LMVe-free. 70.6% of patients (“A”, low-risk) maintained adequate MV (108 ± 5%MVPRED) and had few LMVes (1LMVe/4.4 hr lasting 4.6 ± 0.3 min). A smaller group (18.6%, “B”, moderate-risk) maintained lower MV (56 ± 2%MVPRED¬) and had more frequent LMVes (1LMVe/30 min lasting 5.7 ± 0.3 min). The smallest group (10.8%, “C”, high-risk) had repetitive LMVes (1LMVe/16 min lasting 7.6 ± 1.0 min) indicating potentially unsafe MV. Figure 38 shows the changes in LMVe Rate over time across the 3 groups. In all groups, Early Post-Op (0-3 hr) LMVe Rate was highly correlated with the rate of LMVe in Later Recovery (15–48 hr). For Groups B&C, the LMVe Rate was higher in the Intermediate Post-Op (3-15 hr; B:2.8/hr, C:4.7/hr) compared to the Early Post-Op (B:1.6/hr, C:3.3/hr) and Later Recovery (B:1.2/hr, C:3.3/hr).

**Conclusions**

Most patients maintained adequate MV with few LMVe in the PACU and GHF while a small fraction (C) had repetitive LMVe indicative of OIRD. The RVM could identify these high-risk patients early post-operatively, and changes in therapeutic strategy could help prevent future OIRD.

## P286 Noise levels in an adult ICU - a comparative study

### S Saseedharan, E Pathrose, S Poojary

#### S L Raheja Hosp, Mumbai, India

**Introduction**

To observe the effect of implementation of noise reducing strategies after the findings of our previous study revealed a high percentage of modifiable factors.

**Methods**

Noise levels were measured in the ICU of S L Raheja hospital (A Fortis Associate) for 8 days over a period of 24 hours. 5 recordings were taken at the beginning of every hour using a digital sound meter. An average of these 5 readings were taken and observations were also made.

**Results**

Levels of noise in the ICU were found to be higher than the values suggested by the WHO. The main source of noise were found to be the monitor alarms primarily as a result of not silencing them on time or addressing the cause for the alarm. In comparison to our previous study, the noise levels were found to be higher in the day time than in the night time in spite of the lower values.

**Conclusions**

Modifiable factors from the previous study were noted and measures were taken prior to this study to reduce the noise levels. This study proves that once these factors are controlled, there is reduction in the level of noise in the ICU which also helps to provide comfort to the patient particularly during the night. Our study definitely helps to find a solution to a quieter ICU for the well being of the patients as well as the staff members.

**References**

1. Saseedharan S, Pathrose EJ, Chaddha R, Dahale D. Statistical Study of Noise Levels in an Adult ICU–A Case from India. International Journal of Biomedical Research. 2015 Nov 30;6(11):880–6.

2. Berglund B, Lindvall T, Schwela DH. Guidelines for community noise. InGuidelines for community noise 1999. OMS.

## P287 Effect of a musical intervention on tolerance and efficacy of non-invasive ventilation: the MUS-IRA randomized controlled trial

### J Messika^1^, Y Martin^1^, N Maquigneau^2^, M Henry-Lagarrigue^2^, C Puechberty^3^, A Stoclin^3^, L Martin-Lefevre^2^, F Blot^3^, D Dreyfuss^1^, A Dechanet^1^, D Hajage^1^, J Ricard^1^

#### ^1^Hôpital Louis Mourier, Colombes, France; ^2^CHD de Vendée, La Roche-sur-Yon, France; ^3^Gustave Roussy, Villejuif, France

**Introduction**

Music therapy is effective in ICU to reduce anxiety in invasively ventilated subjects (1). We investigated the effect of a musical session on NIV tolerance and efficacy in ICU patients with acute respiratory failure (ARF).

**Methods**

Randomized 3-center, 3-arm open-label trial. Inclusion of adult ARF patients requiring NIV, with a GCS > 11, without non-inclusion criteria (contra-indication to NIV; severe hearing impairment; withdrawal of life sustaining therapies). Interventions consisted in: “musical intervention” (MI) and “sensory deprivation” (SD) where patients received a sleeping mask and an insulating around-ear headphone, during 30-minutes. In addition, MI subjects received a 30-minute music session (MUSIC CARE© software (2). The “NIV alone” group received conventional care (no headphone, no sleeping mask).

The main objective was to determine if MI improved NIV tolerance at 30 minutes (T30) in comparison to conventional care. Respiratory comfort was assessed with a numeric visual scale. Primary endpoint: change in respiratory comfort between T0 and T30 of the first NIV session. Secondary endpoints: NIV failure; NIV-associated psychotrauma assessed by the Peritraumatic Distress Inventory (PDI) at ICU discharge and after 3 months (M3); M3 evaluation of anxiety and depression (HADS); quality of life (SF36). 99 subjects had to be included to show a 2-unit difference in comfort between 2 groups.

**Results**

From May 2015 to June 2016, 114 subjects (63 men; 55.3%, aged 67y [60-74]) were randomized (MI n = 37; SD n = 38, NIV-only n = 39). Baseline respiratory comfort for the first NIV-session did not differ among groups. Mean change in respiratory comfort between T0 and T30 did not differ (MI 0.54 ± 3.6; SD 0.55 ± 2.3; NIV-alone 0.66 ± 2.9; p = 0.91 for MI vs NIV-alone). NIV failure rates in ICU did not differ (MI 16.2%, SD 10.5%, NIV-alone 12.8%; p = 0.74). NIV-associated psychotrauma was significantly lower in MI group (PDI scores 8 [5,3-13]; SD 16 [5,5-22,5]; NIV-only 16 [12,5-25]; p = 0.03), without difference at M3. HADS at M3 were the following (9.5 [6-13]; 11 [8-17]; 14.5 [8.8-18.5], for MI, SD and NIV-alone respectively, p = 0.17). SF36 score was not different between arms at M3.

**Conclusions**

An early musical intervention during NIV for ARF did not modify respiratory comfort, but allowed a reduction in NIV-related psychotrauma at ICU discharge.

**References**

1. Chlan LL, et al. Jama. 2013; 309:2335–44.

2. Guetin S et al. Doul Analg. 2005; 1:19–25.

## P288 Early mobilization program improves functional capacity after major abdominal cancer surgery: a randomized and controlled trial

### E Almeida^1^, J Almeida^1^, G Landoni^2^, F Galas^1^, J Fukushima^1^, E Fominskiy^3^, C De Brito^1^, L Cavichio^1^, L Almeida^1^, U Ribeiro^1^, E Osawa^1^, R Boltes^1^, L Battistella^1^, L Hajjar^1^

#### ^1^Instituto do Cancer, Sao Paulo, Brazil; ^2^IRCCS San Raffaele Scientific Institute, Milan, Italy; ^3^Academician EN Meshalkin Novosibirsk State Budget Research Institute of Circulation Pathology, Novosibirsk, Russia

**Introduction**

Major abdominal oncology surgery is associated with substantial postoperative loss of functional capacity and decline in physical status that may turn cancer therapy impractical and compromise patient outcomes. Exercise is an effective intervention to improve outcomes after high-risk surgery.

**Methods**

We performed a single-blind, randomized and controlled trial in patients who underwent major abdominal oncology surgery in a tertiary university hospital. Patients were randomized to an early mobilization postoperative program based on supervised aerobic exercise, resistance and flexibility training or to standard rehabilitation care. The primary outcome was inability to walk, defined as the inability to cross the room or to walk a distance of 3 meters without human assistance, at postoperative day 5.

**Results**

A total of 108 patients were enrolled, 54 into the early mobilization program group and 54 into the standard rehabilitation care group. Nine (16.7%) patients in the early mobilization group were not able to cross the room or to walk 3 meters without human assistance at postoperative day 5 compared to 21 (38.9%) of patients in the standard rehabilitation group (P = 0.01) with an absolute risk reduction of 22.2% (95%CI 5,9 to 38.6%) and a number needed to treat of 5 (95% CI 3 to 17).

**Conclusions**

An early postoperative mobilization program improves functional capacity compared to a standard rehabilitation care in patients undergoing major elective abdominal oncology surgery.

**Reference**

Castelino T, Fiore JF Jr, Niculiseanu P, Landry T, Augustin B, Feldman LS. The effect of early mobilization protocols on postoperative outcomes following abdominal and thoracic surgery: A systematic review. Surgery 2016; 159: 991–1003.

## P289 Early mobilization in mechanically ventilated patients: a one-day prevalence point study in intensive care units in Brazil

### P Fontela^1^, T Lisboa^1^, L Forgiarini Junior^2^, GF Friedman^1^

#### ^1^UFRGS, Porto Alegre, Brazil; ^2^Centro Universitário Metodista - IPA, Porto Alegre, Brazil

**Introduction**

There is growing evidence supporting the safety, feasibility, and benefit of early mobilization in mechanically ventilated patients in the intensive care unit (ICU). However, there is little knowledge about practices in early mobilization in ICUs. We conducted a multicenter one-day prevalence study in Brazilian ICUs to assess the prevalence of early mobilization in patients under mechanical ventilation (MV), as well as to identify barriers associated with their performance.

**Methods**

A cross-sectional prevalence point study of one day performed in 11 ICUs, including all adult patients under MV, during the 24 hours of June 21, 2016. Demographic data, airway type, highest level of mobilization (scale of 8 levels and the most important barrier to mobilize at a higher level. The mobilization was evaluated as a binary variable: "in bed" (level 1-3) or "out of bed" (level 4-8).

**Results**

A total of 140 patients were included, 57(17) years of age, of which 90 (64%) were male. Median time in MV and ICU stay was 7 days. Of the total, 14 (10%) patients were mobilized out of bed. Among patients with endotracheal tube, tracheostomy, and noninvasive ventilation, 2%, 23% and 50% were mobilized out of bed, respectively (p < 0.001 between groups) (Table 22). The most common barriers to mobilizing at a higher level were weakness (20%), cardiovascular instability (19%) and deep sedation (18%).

**Conclusions**

In this prevalence point study, only 10% of all mechanically ventilated patients and only 2% of endotracheal tube patients were mobilized out of bed as part of the routine care.Modifiable barriers, such as deep sedation, seem to be important to increase mobilization in Brazilian ICUs.

**References**

1. The TEAM Study Investigators. Early mobilization and recovery in mechanically ventilated patients in the ICU: a bi-national, multi-centre, prospective cohort study. Crit Care. 2015;19:1–10.

2. Nydahl P, Ruhl AP, Bartoszek G, Dubb R, Filipovic S, Flohr HJ, et al. Early Mobilization of Mechanically Ventilated Patients: A 1-Day Point-Prevalence Study in Germany. Crit Care Med. 2014;42:1178–86.

**Table 22 (abstract P289).** See text for description

Level of mobilization	Total (n = 140) n (%)	Endotracheal tube (n = 98) n (%)	Tracheostomy (n = 34) n (%)	Non-invasive MV (n = 8) n (%)
Kept in bed (p < 0.01)	126 (90)	96 (98)	26 (76)	4 (50)
Absence of mobilization	25 (18)	23 (23)	2 (6)	0 (0)
Turn in the bed	58 (41)	45 (46)	13 (38)	0 (0)
Sitting on the bed	43 (31)	28 (29)	11 (32)	4 (50)
Mobilized out of bed (p < 0.01)	14 (10)	2 (2)	8 (23)	4 (50)
Sitting on the edge of the bed	2 (1)	1 (1)	1 (3)	0 (0)
Sitting outside the bed	9 (6)	1 (1)	5 (15)	3 (37)
Standing out of bed	1 (1)	0 (0)	0 (0)	1 (12)
Stationary gait	1 (1)	0 (0)	1 (3)	0 (0)
Walk	1 (1)	0 (0)	1 (3)	0 (0)

Legend: Highest Level of Mobilization on the Study Day

## P290 Ultra early mobilization reduces the time of mechanical ventilation and ICU stay

### F Abruzzi^1^, J Azevedo Peixoto Primo^1^, P Marques Filho^1^, J Stormorvski de Andrade^1^, K Matos Brenner^1^, M Scorsato boeira^1^, C Leães^2^, C Rodrigues^1^, A Vessozi^1^, A SantAnna Machado^1^, M Weiler^1^

#### ^1^Hospital Ernesto Dornelles, Porto Alegre, Brazil; ^2^Hospital Mae de Deus, Porto Alegre, Brazil

**Introduction**

Prolonged use of mechanical ventilation can lead to a number of deleterious effects on patients admitted in intensive care units. Early mobilization provides a reduction in the days of mechanical ventilation during the permanence in the ICU and also provides an improvement in the quality of life. Therefore our study aimed to evaluate the initial effect of physiotherapeutic treatment during the period of hospitalization at the ICU and measure the time of mechanical ventilation.

**Methods**

The sample was characterized with patients admitted in the ICU who were able to received physiotherapy sessions. We evaluated the patients in 3 moments: 0-12 hours (P1), those that were attended between 13-24 hours (P2) and those that were attended after 24 hours (P3).The data were analyzed by ANOVA / SNK and were expressed as mean ± standard deviation. Significant p <0.05.

**Results**

There were includes 120 patients, these 54.4% were male. The mean age was 77.1 ± 11.9. P3 had a lower SAPS III (P3 vs. P1, P2 F(2.304) = 9.4, P <0.05). The underlying pathologies of these patients were: chronic obstructive pulmonary disease 5.9%, acute respiratory failure 15.7%, sepsis 28.2%, stroke 3.3%, postoperative of abdominal surgery 5.6%, cardiovascular surgery 19.7% and others 21.6%. We observed that patients who started treatment within a period of less than 24 hours had a shorter mechanical ventilation time (F(2,275) = 13.5, p <0.05) and a reduction in hospitalization time (F(2,434) = 29.5. p <0.05) compared to the P3 group (>24 h). There were no incidents during the mobilization or accidental extubation process in any of the groups.

**Conclusions**

Our study demonstrates the importance of early physiotherapeutic treatment that may help reduce ICU length of stay and decrease the mechanical ventilation time. No incident or damage occurred to the patients, which reinforces that early mobilization is safe and viable. Our data demonstrate that it is important to encourage early mobilization in critically ill patients.

**References**

PARRY, Selina M.; PUTHUCHEARY, Zudin A. The impact of extended bed rest on the musculoskeletal system in the critical care environment. Extreme Physiology & Medicine, 2015.

SCHWEICKERT, William D et al. Early physical and occupational therapy in mechanically ventilated, critically ill patients: a randomised controlled trial. The Lancet, 2009.

## P291 Improved physiotherapy outcome measures by the use of cycle ergometry in critical care patients.

### H Bryce, A Hudson, T Law, R Reece-Anthony, A Molokhia

#### University Hospital Lewisham, London, United Kingdom

**Introduction**

The aim of this study is to determine the efficacy of cycle ergometry in addition to standard physiotherapy, compared to standard physiotherapy alone, in an adult critical care unit over an 8 week period.

Patients who develop critical care-acquired weakness are at higher risk of mortality, longer duration of mechanical ventilation, longer critical care length of stay, and higher hospital costs [1]. Muscle weakness can be present within hours of starting mechanical ventilation and is evident in 25-100% of patients ventilated for over 7 days [2]. Whilst the cause for this is multifactorial, early rehabilitation can help reduce loss of muscle mass and loss of physical conditioning [3].

**Methods**

The treatment group received standard physiotherapy with an additional 20-minute exercise bike session (seated or supine devices), 5 days per week. The control group received standard physiotherapy. Exclusion criteria was defined as per published literature [4]. 5 patients were allocated to each group. Outcome measures recorded were handgrip strength, quadriceps strength using Medical Research Council Scale (MRC), and Critical Care Physical Assessment Tool (CPAx). These were measured on the day the patient was deemed eligible, and the day they were deemed stable to step down to ward level.

**Results**

No adverse effects were identified during and immediately after exercise training. There was notable improvement in the treatment group in all three outcome measures, compared to that of the control group (Table 23). Positive subjective feedback was received from all treatment group participants.

**Conclusions**

Data collected demonstrated improvement in all variables measured in the physiotherapy and cycle ergometry group compared to those receiving standard physiotherapy alone.

Interpretation of data has been limited by a small sample size, however, early data suggests benefit of cycle ergometry with physiotherapy in reducing critical care acquired weakness, morbidity, and as an effective form of early rehabilitation. Results would suggest benefit in expanding the project to a larger patient group over a longer time period.

**References**

1. Hermans et al. Am J Respir Crit Care Med 2014;190:410–20

2. Williams et al. Physiother Theory Pract. 2014;30(1):6–11

3. Troung et al. Crit Care. 2009;13(4):216

4. Burtin et al. Crit care Med 2009;37(9):2499–2505

**Table 23 (abstract P291).** See text for description

OUTCOME MEASURE	% IMPROVEMENT CONTROL GROUP	% IMPROVEMENT TREATMENT GROUP
GRIP STRENGTH (kg) RT / LT	9% / 12%	33% / 32%
QUADRICEPS STRENGTH (MRC) RT / LT	3% / 6%	11% / 13%
CPAx	10%	49%

Legend Table 23

## P292 The effect of night shift and melatonin imbalance on neurological situations of critical care nurses: a literature review

### F Abtahinezhadmoghaddam

#### Shiraz University of Medical Sciences (SUMS), Shiraz, Iran

**Introduction**

Due to the special role of critical care nurses as a key member of the medical team and their direct communication with patients and other health-care community, their health is very important. Therefore, this review was done to determine the effect of night shift and melatonin imbalance on neurological situations of critical care nurses.

**Methods**

This review conducted on 15 studies which were investigated the effect of night shift on nurses health during 5 years.

**Results**

The review showed that Long-term night-shift in nurses getting more cynical and less empathetic as their training progresses, and might lead to many health-related problems. Disrupted body rhythms during shiftwork, lead to major physiological and psychological effects on nurses that may also affect badly on patient’s safety and the quality of care provided. The major cause of these problems is the deficiency of melatonin which is a hormone, secreted from the pineal gland, a pea-sized conical mass of tissue behind the third ventricle of the brain. Suppression of the night-time production of melatonin due to exposure to light at night has been the major point. This hormone is able to cross BBB and it has neuroprotective role in human being and is proved to be a versatile hormone having antioxidative property. On the other hand, oxidative stress, characterized by increased free radical damage, has been implicated in neurological disorders especially Alzheimer’s disease and this effect is more risky and common in those over 40 years of age and may be more profound in a critical care setting. Deprived daytime sleeping may influence body homeostatic functions and causes fatigue and other health problems. Researches recommend that fatigue can negatively affect nurses’ health, quality of performance and safety.

**Conclusions**

It is concluded that nurses working in these environments are able to maintain careful and astute observation of their vulnerable patients, and concern arises when they may be unable to do so.

**References**

1. Deori D. International Journal of Latest Research in Science and Technology. 2012; 201–204

2. MUECKE S. Journal of Advanced Nursing .2005 ; 433–439

3. Shinozuka K et al.: International Journal of Molecular Sciences 2013; 14: 8924-8947

4. Naseem M et al.: The Scientific World Journal. 2014; 13 pp

## P293 Blood tests in the intensive care unit: a necessary cost?

### E Cumber, L Channon, A Wong

#### John Radcliffe Hospital, Oxford, United Kingdom

**Introduction**

The ICU is a unique clinical setting where the human body functions at the extremes of physiological capacity. Thus, more regular monitoring of observations and blood results is often appropriate. However, investigations should be clinically justified as they can contribute to anaemia and not an insubstantial cost.

Tests are often requested by nurses on a daily basis with no clinician input as a matter of routine. These include clotting, full blood count, urea and electrolytes (including Mg, Ca and PO4), CRP and liver function.

We propose that this is potentially costly, clinically unnecessary and may be driven by a lack of understanding of the cost implications.

**Methods**

Over 5 randomly selected days, all patients on the ICU for > =3 days had all requested blood tests recorded for the preceding 7 days (or their total ICU stay if this was less). The average number of investigations per patient day was calculated. The costs of all investigations was obtained from the relevant department managers, allowing average and total costs to be calculated. ICU staff were concomitantly surveyed to assess their understanding of common investigations.

**Results**

On average patients had 3.7 laboratory blood tests and 7.3 point of care (POC) tests per day. In our trust, at maximum capacity this average amounts to a total cost of £362,587.80 per year (£266,841 of which is POC).

Survey results revealed a complete lack of insight into test costs, with no staff members confidently able to state costs. The free text response highlighted an overwhelming opinion that laboratory tests are requested as routine rather than based on clinical need. The average ICU patient stay in this trust is 3 days. It was felt clotting was most often repeated despite multiple previous normal results. Requesting clotting once every 3 days rather than daily would save £23,360 a year. Ca is also requested daily and is duplicated on multiple POC tests. Removing calcium from daily requests would save a further £2890.

**Conclusions**

Using clinical judgement to break the habit of daily routine blood requesting could save significant amounts of money. The process can be further enhanced through the use of electronic checklists which could have significant financial benefit without any impact on patient safety.

## P294 The electronic documentation of FAASTHUG on the Intensive Care Unit ward round

### R Groome, D Gearon, J Varley

#### Addenbrooke’s Hospital, Cambridge University Hospitals NHS Foundation Trust, Cambridge, United Kingdom

**Introduction**

A checklist is a cognitive aid used to reduce the risk of human errors during complex task completion. FASTHUG is a mnemonic devised by JL Vincent in 2005[1] that encompasses key aspects of patient care recommended for daily review on the Intensive Care Unit (ICU): Feeding, Analgesia, Sedation, Thromboprophylaxis, Head of bed elevation, Ulcer prophylaxis, Glucose control. We hypothesise that as a standard of care every patient on the ICU should receive a daily version of FASTHUG.

**Methods**

An audit was performed that reviewed the electronic morning ward round notes for documented evidence of the components of FASTHUG. The mnemonic was modified to include a second ´A´ for antibiotics. One point was allocated for each component of the modified FAASTHUG documented in the notes (maximum score 8). Data was collected retrospectively each Thursday in March 2016. Ward round entries were classified as systematic if the clinician used an electronic note template and non-systematic if they used free text.

**Results**

The number of patients included in the study was 70. Three patients were excluded (1 death and 2 underwent surgery during the ward round). 6 out of 8 (61%) of the FAASTHUG components were documented on average for each patient. The number of patients with a systematic vs non-systematic ward round entry were 49 and 21 respectively. On average, 75% of the FAASTHUG components were documented in patients with a systematic review, compared to 29.2% of the patients with a non-systematic review (Tables 24 and 25). Universally, sedation and antibiotics scored highly. In contrast, head up elevation was not documented in any of the patients’ notes. The Mann Whitney U test proved statistical significance (p < 0.011) between the documentation of systematic and non-systematic ward round entries.

**Conclusions**

Our study proves that using a electronic note template increases the documentation of the FAASTHUG components. We have introduced an electronic template for the modified FAASTHUG and are currently re-auditing to identify whether implementation of this improves documentation. This has the potential to improve the quality of care in our patients.

**References**

1. Vincent JL. Give your patient a Fasthug (at least) once a day. Crit Care Med. 33(6):1225–9, 2005.

2. Ferreira CR et al. The effectiveness of a bundle in the prevention of ventilator acquired pneumonia. The Brazilian Journal of Infectious diseases. 20(3):267–271, 2016.

3. Papadimos TJ et al. Implementation of the “FASTHUG” concept decreases the incidence of ventilator acquired pneumonia in a surgical intensive care unit. Patient Saf Surg. 2:3, 2008.

**Table 24 (abstract P294).** See text for description

Systematic	F	Abx	Ana	S	T	H	U	G	Average documented %
% Documented	95.92	97.96	59.18	95.92	93.88	0	79.59	77.56	75.00

Legend: Percentage of FAASTHUG components documented in systematic review patients

**Table 25 (abstract P294).** See text for description

Non-systematic	F	Abx	Ana	S	T	H	U	G	Average documented %
% Documented	28.57	57.14	14.29	80.95	28.57	0	0	23.81	29.19

Legend: Percentage of FAASTHUG components documented in non-systematic review patients

## P295

### Withdrawn

## P296 Effect of weekend admission on the hospital mortality in ICU patients

### FG Zampieri^1^, FA Bozza^2^, M Ferez^3^, H Fernandes^4^, A Japiassú^5^, J Verdeal^6^, AC Carvalho^7^, M Knibel^8^, JI Salluh^2^, M Soares^2^

#### ^1^HCor-Hospital of the Heart, São Paulo, Brazil; ^2^D’Or Institute for Research and Education, Rio de Janeiro, Brazil; ^3^Hospital São Francisco, Riberão Preto, Brazil; ^4^Hospital São Luiz Brasil, São Paulo, Brazil; ^5^Rede Amil, Rio de Janeiro, Brazil; ^6^Hospital Barra D’Or, Rio de Janeiro, Brazil; ^7^UDI Hospital, São Luís, Brazil; ^8^Hospital São Lucas Copacabana, Rio de Janeiro, Brazil

**Introduction**

ICU admission during weekends may be associated with increased mortality in critically ill patients although putative mechanisms for this are lacking. We assessed whether weekend admission was associated with hospital mortality while adjusting for ICU staffing and organization.

**Methods**

Secondary analysis of a multicenter retrospective cohort study including 59,614 (medical patients, 67%) unique ICU admissions to 78 ICUs during 2013 in Brazil. Weekend admission was defined as any ICU admission from Friday 7 pm until Monday 7 am. We also determined the percentage of weekend hours (weekend hours / total hours spent in the ICU) in the first 5 days of ICU admission, regardless of admission day. We used multilevel logistic regression analysis to evaluate the association between weekend admission and hospital mortality adjusting for patients’ characteristics, percentage of weekend hours, presence of weekend multidisciplinary rounds, intensivist 24/7 in the ICU, and changes in nurse/bed ratio (decrease during weekend versus no decrease).

**Results**

17,720 (27.9%) patients were admitted during the weekends. Compared to patients admitted during the weekdays, they were older, had higher SOFA, worse performance status and higher hospital length of stay before admission. In univariate analyses, both admission during the weekend [OR = 1.28 ((1.22-1.35)] and the percentage weekend hours [OR = 1.03 (1.02-1.04), per 10% increase] were associated with higher hospital mortality. However, adjusting for other confounders, admission during weekends [OR = 1.03(0.97-1.10)] was no longer associated with increased hospital mortality, but percentage weekend hours was still associated with mortality [OR = 1.01(1.00-1.02)]. In subgroup analyses, the association between higher percentage weekend hours and mortality was only evident in the subgroup of ICUs with low baseline nurse/bed ratios (<0.25 nurses/bed).

**Conclusions**

A ‘weekend effect” on the outcomes of ICU patients was explained by a higher exposure to the weekend in the first days of ICU rather than simply being admitted during the weekend. This association as more evident in ICUs with a lower nurse/bed ratios.

## P297 Dermatology consultations in a specialist ECMO and cardiothoracic ICU

### J Gao, E Ahmadnia, B Patel

#### Royal Brompton Hospital, London, United Kingdom

**Introduction**

Whilst dermatological diagnoses are an uncommon cause for ICU admission (<0.5% of all admissions) [1], the incidence of new skin lesions in the general ICU population is not insignificant (up to 42%) [2]. These often include infection mimics which may receive inappropriate antimicrobial therapy - this can be mitigated by dermatology consultations [3]. There is no literature on dermatological consults in the setting of Extracorporeal Membrane Oxygenation (ECMO)/cardiothoracic ICU. The aim of this study was to determine the burden and nature of skin conditions referred for dermatological consultation in a specialist ECMO/cardiothoracic ICU setting, and whether management changed as a result.

**Methods**

A retrospective analysis was conducted of all adult patients admitted to a specialist ECMO/cardiothoracic ICU between October 2014 and October 2016. All patients referred for dermatology consultation were included, and data on organ support, nature of consultation, and final dermatological diagnosis were collected.

**Results**

27 dermatology referrals were made, from a total of 1329 patients. Of these referrals, 9 (33%) were patients on ECMO (predominantly veno-venous); the majority were invasively ventilated (92.5%) and on vasoactive support (78%). 48% underwent skin biopsy. The most common diagnosis seen was drug-related rash (52%). Other diagnoses included vasculitis (12%), pressure or trauma related skin injuries (12%), folliculitis (8%), fungal rash (4%), bacterial skin infection (4%), and subcutaneous haematoma (4%). Within the ECMO subset, the most common diagnosis was drug-related rash (6/9) and 4/9 underwent skin biopsy. Overall, there was a change made in patient management based on dermatology consultation in 76% of cases. Of note, 44% of dermatology consultations were conducted via tele-dermatology and the remainder were traditional consultations.

**Conclusions**

This study is the first in the literature to describe dermatology consultations in the setting of ECMO and cardiothoracic ICU. In this patient population, the most common skin disorders are drug-related rash, vasculitis and pressure-related skin injuries. Tele-dermatology is a viable option for obtaining dermatology consultations in ICUs where there is no on-site dermatology service. Further research is needed to evaluate whether ECMO is a risk factor for secondary skin disorders.

**References**

[1] George SM et al. Crit Care 12(Suppl 1):S1

[2] Agrawal P et al. Postgrad Med J 2013 89:501–507

[3] Burnham JP et al. Intensive Care Med (2016) 42: 1899

## P298 Introduction of regular critical care consultant cover in medical high dependency: effects on unit activity, unit outcomes, and quality metrics

### J McCartney, A MacKay, S Binning, C Wright

#### Queen Elizabeth University Hospital, Glasgow, United Kingdom

**Introduction**

The Queen Elizabeth University Hospital opened in May 2015. The medical high dependency unit (MHDU) opened on 15/06/2015 – it predominantly provides level 2 care for medical patients, however multi-organ support (out with invasive mechanical ventilation and renal replacement therapy) can also be provided within MHDU if appropriate. The MHDU was run initially as an “open model” with admissions through on-call medical teams and their subsequent medical care being the admitting team’s responsibility. From 05/01/16 this system changed with a critical care consultant being based within the unit during the hours of 0800 -1800, Monday- Friday, their responsibility was to provide overall supervision of care, assess the appropriateness of admissions, and provide ongoing education and support for nursing and medical staff. The aim of this study was to evaluate the impact of this intervention on unit activity, outcomes, and quality metrics.

**Methods**

Retrospective, observational cohort study for purposes of service review. Local critical care database interrogated. Data on unit activity, unit outcomes, and quality metrics collected and period pre (15/06/15-04/01/16) and post (05/01/16-26/07/16) intervention compared by univariate analysis using MedCalc to detect significant changes following intervention.

**Results**

Post intervention there was a significant decrease in out of hours discharges (OOHDC) (pre 13%, post 6.7%, p = <0.01) and a significant increase in days at a dependency level of > =2 (pre 63.8%, post 76.1%, p = <0.01). There was no significant change in unit mortality rate (pre 7.5%, post 9.7%, p = 0.18), readmission rate (pre 4%, post 5.2%, p = 0.35), rate of requirement for escalation to ICU (pre 7%, post 7.3%, p = 0.89), or median length of stay (pre 1.9 days, post 1.9 days, p = 0.87).

**Conclusions**

In our cohort, regular critical care input into MHDU was associated with a decrease in OOHDC rate and an increase in days spent at level 2 dependency or higher. The reduction in OOHDC was a desirable outcome given the association between OOHCDC and excess morbidity in critically ill patients. The increase in days at level 2 or higher dependency suggests a more appropriate use of the resource, it may also be suggestive of a sicker cohort of patients, it is interesting to note that there was no significant increase in unit mortality associated with this. We believe that regular input from clinicians with critical care training is beneficial in the MHDU.

## P299 A survey of VAP prevention practices and beliefs in Wales

### RJ Pugh ^1^, C Battle^2^, C Hancock^3^, W Harrison^3^, T Szakmany^4^

#### ^1^Glan Clwyd Hospital, Rhyl, United Kingdom; ^2^Morriston Hospital, Swansea, United Kingdom; ^3^Public Health Wales, Cardiff, United Kingdom; ^4^Cardiff University, Cardiff, United Kingdom

**Introduction**

Efforts to reduce ventilator-associated morbidity have been well-established in Welsh ICUs since the introduction of a standard care bundle in 2006. However, as evidence for interventions has evolved, variations in practices seem likely. Our aims were to investigate practices across Welsh ICUs and to sample views from staff regarding interventions for the prevention of ventilator-associated pneumonia (VAP) using an online survey.

**Methods**

From August-November 2016, we surveyed ICU staff via the Welsh Intensive Care Society (WICS) Audit and Research Group. We used a mix of binary questions to understand current practice, Likert scale questions to gauge beliefs, and free text for additional commentary.

**Results**

80 healthcare professionals responded from all 13 (100%) centres: consultants (35%), nurses (24%), physiotherapists (14%), medical trainees (11%), nurse practitioners (5%), microbiologists (4%), non-trainee doctors (4%). 93% felt episodes of VAP are associated with poorer outcomes and 82% that VAP is preventable. Routine measurement of sedation interruption was reported by 82% and bed elevation by 65%. 90% felt that bed elevation and 73% that routine de-sedation reduces incidence of VAP. Stress ulcer prophylaxis is administered unselectively (83%) and increases VAP incidence (according to 34%). Responses to other interventions are summarised in the Tables.

**Conclusions**

VAP prevention practices vary between Welsh ICUs. Oral chlorhexidine and routine stress ulcer prophylaxis are reported by the majority, despite recent suggestions of harm or doubtful benefit. In contrast, SDD has little support despite evidence of efficacy. Redesign of VAP bundles to ensure a more uniform approach is recommended.

**Table 26 (abstract P299).** See text for description

Intervention	Is performed in my unit (%)	Reduces VAP incidence (%)
Routine ventilator circuit change	64	44
ETTwith sub-glottic suction	73	57
ETT with automated cuff pressure	10	19
Silver coated ETT	4	13

Legend: Interventions: ETT/ ventilator circuit

**Table 27 (abstract P299).** See text for description

Intervention	Is administered in my unit (%)	Reduces VAP incidence (%)	Reduces mortality (%)
Oral chlorhexidine	65	42	14
SOD	10	18	14
SDD	3	23	16

Legend: Interventions: enteral/ systemic decontamination

## P300 The cost of intensive care in a major peripheral Belgian hospital: reappraisal of the cost-block method anno 2016

### F Mulders, J Vandenbrande, J Dubois, B Stessel, K Siborgs, D Ramaekers

#### Jessa Hospitals, Hasselt, Belgium

**Introduction**

Healthcare expenditures keep rising due to a variety of reasons. Since governments try to economize healthcare funding, cost-effectiveness analyses gain importance. ICUs are a major cost, though costing is complex. The cost-block method was developed to identify costing in a standardized top-down manner. Six cost blocks were defined: equipment cost, estates, non-clinical support services, clinical support services, consumables and staff. In our study we evaluated the cost of the Jessa Hospital ICU over one fiscal year. The ICU counts 34 beds, spread over 4 units on 2 campuses. Our primary aim was to develop a pie chart summarizing our ICU costing.

**Methods**

We performed a retrospective observational study. We reviewed the cost pool of 2014. Each topic was allocated to a strictly defined block as postulated by the Medical Economics and Research Centre. Concerning blocks 3 and 4, the cost pools of respective departments were consulted. Proportionate ICU use calculation of their operating expenses was performed.

**Results**

Staffing appeared to be the major contributor to ICU costing in 2014 (53.5%). Clinical support services and consumables made up 24.6% and 15% respectively. Non-patient related cost blocks (1-3) contributed 6.95% to total Jessa ICU cost in 2014.

**Conclusions**

Our findings are in line with ICU cost-block analyses performed in other European countries. The staffing block makes up 53.5% of costs. Block 4 and 5 form 40% of our ICU cost. Clinical support services weighted more than consumables. Non-patient related blocks are a minor ICU cost. We found them to make up an even smaller proportion than in former studies. This might be explained by our relative older hospital estates which are payed off. We confirm that the cost-block method still is a useful tool. In times of healthcare economization, cost-blocks can be used to determine cost drivers of excessive ICU costs.

**References**

The development of a method for comparative costing of individual intensive care units. Edbrooke D et al. Anaesthesia 1999:54(110-120).

International Programme for Resource Use in Critical Care (IPOC) – a methodology and initial results of cost and provision in four European countries. Negrine D et al. Acta Anaesthesiol Scand 2006;50:72-79.

**Fig. 39 (abstract P300).**
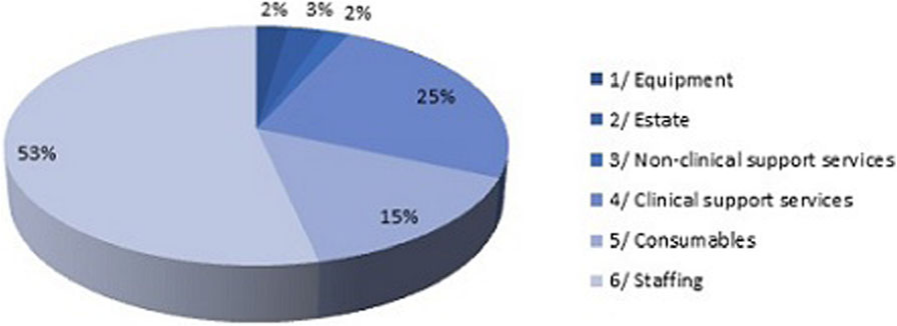
Proportion of each cost block to the total cost

## P301 Family care, visiting policies, ICU performance and efficiency in resource use: insights from the ORCHESTRA study

### M Soares ^1^, UV Silva^2^, WS Homena Jr^3^, GC Fernandes^4^, AP Moraes^5^, L Brauer^6^, MF Lima^7^, F De Marco^8^, FA Bozza^1^, JI Salluh^1^

#### ^1^DOr Institute for Research and Education - IDOR, Rio De Janeiro, Brazil; ^2^Hospital de Câncer de Barretos, Barretos, Brazil; ^3^Hospital Barra DOr, Rio de Janeiro, Brazil; ^4^Santa Casa de Misericórdia de Juiz de Fora, Juiz de Fora, Brazil; ^5^Hospital de Câncer do Maranhão Dr Tarquinio Lopes Filho, Sao Luis, Brazil; ^6^Hospital São Luiz Itaim, Sao Paulo, Brazil; ^7^Hospital Esperança Recife, Recife, Brazil; ^8^Hospital viValle, Sao Jose dos Campos, Brazil

**Introduction**

Despite several recommendations, visiting hours remain restricted in many ICUs. Among the several objections to a more liberal access of families, there are concerns regarding the interference with patients’ management and ICU workflow which may adversely affect patients’ outcomes, and ultimately ICU performance. Here, we explored the association between family care related policies with ICU performance and resource use adjusted for the severity of illness.

**Methods**

Secondary analysis of a multicenter retrospective cohort study of critical care organization and outcomes in 59,693 (68% medical) patients admitted to 78 ICUs during 2013 in Brazil.[1] We retrieved patients’ data from an ICU registry and surveyed participating ICU about characteristics related to ICU organization, incluidng visiting policies, communication and participation of family members in patients’ management. We evaluated outcomes and efficiency in resource use for each ICU by estimating the standardized mortality rates (SMR) and resource use (SRU) according to the SAPS 3.

**Results**

Participating ICUs were mostly medical/surgical (79%) and located at private hospitals (92%). Median SAPS 3 score was 41 (33-52) points and median estimated SMR and SRU were 0.97 (0.72-1.15) and 1.06 (0.89-1.37), respectively. The hospital mortality was 14.4%. Median visiting hours was 2 (1.5-6) and only 3 (4%) ICUs had unrestricted open visiting policies. In general, ICUs with lower SMRs had more liberal policies in different domains, includinh higher number of visiting hours and allowing participation of family members in patients´ care. The same was observed in ICUs with a high-efficiency in resource use (i.e. ICUs with both low SMR and SRU). In addition, visiting hours were higher in high-efficiency ICUs [4 (2-7) vs. 2 (1-4), p = 0.001].

**Conclusions**

ICU performance and efficiency in resource use are potentially associated with family care related and visiting policies. Although a causal association cannot be established, more liberal visiting policies and family participation in patients’ care can be implemented without compromising these outcomes. Alternatively, we can infer that the overall ICU organization in more efficient ICUs creates conditions to allow a more liberal access of families.

**Reference**

1. Soares et al. ICM 2015. doi: 10.1007/s00134-015-4076-7

## P302 Burnout in ICU-10 year experience

### N Maric, M Mackovic, N Udiljak

#### Clinical Hospital Sveti Duh, Zagreb, Croatia

**Introduction**

Burnout is a psychological job-related condition consisting of feelings of emotional exhaustion (EE), depersonalization (DEP) and reduced personal accomplishment (PA). It is a well recognized entity among healthcare professionals, especially among ICU staff. Current data show that burnout prevalence among critical care staff have reached alarming levels - up to 45% of physicians and nearly 1/3 of critical care nurses are experiencing severe burnout. In the light of aforementioned, we were interested in the current burnout status in a single center MICU as well as burnout dynamics since the year 2005.

**Methods**

Maslach Burnout Inventory (MBI) consisting of 22 items was used as a psychometric instrument for measuring burnout. Standardized questionnaire was anonymously filled in by 6 MICU physicians and 23 nurses. Obtained data were compared to results from an identical questionnaire provided in the same MICU in the year 2005. Statistical analyses used were X ± SD and Mann-Whitney rank sum t test (p < 0.05).

**Results**

Total MBI score for ICU staff was 70.1 ± 15.4. In general, ICU staff showed moderate degree of burnout in all 3 MBI subscales (EE, DEP, PA). When subgrouped, physicians had moderate degree of EE (26.67 ± 15.92) and high degree of DEP (14.17 ± 6.56) and PA (29.83 ± 3.19). College nurses experienced moderate degree of EE (29.58 ± 12.1) and DEP (11.17 ± 6.6), and high level of PA (32.58 ± 6.1) while moderate degree for all 3 subscales (EE 25.36 ± 14.04, DEP 6.91 ± 5.86, PA 34 ± 7.66) was observed in registered nurses. Total scores of MBI were higher in the year 2016 (70.1 ± 15.4) than 2005 (65.5 ± 6.7), but statistically insignificant. Between two investigated periods, in 2016 higher scores for EE (27.38 ± 13,30 vs 24.97 ± 11.25) and DEP (10.17 ± 6.71 vs 6.06 ± 5.64) were detected, but only for DEP at significant level (p < 0.05). PA scores were lower in 2016 (32.55 ± 6.29 vs 34.44 ± 8.5), without statistical significance. In addition, in 2016 ICU staff was significantly younger (37.21 ± 12.3 vs 42.33 ± 5.51) and with shorter length of employment (11.17 ± 7.36 vs 14.33 ± 5.77) than in 2005 (p < 0.05).

**Conclusions**

Although significant level of burnout was observed 10 years ago, MBI scores remain high despite personnel being younger and with shorter length of ICU employment. Our findings are consistent with results of many other worldwide conducted studies making it almost a universal phenomenon among ICU staff. Taking this into account, it is questionable whether these results suggest that burnout is really a job related psychological disorder or rather an adaptive mechanism to ICU working conditions.

## P302 Impact of multidisciplinary healthcare team in mortality and readmission rate in a Brazilian cardiac intensive care unit

### CE Bosso^1^, RD Caetano^1^, AP Cardoso^1^, OA Souza^2^, R Pena^2^, MM Mescolotte^2^, IA Souza^2^, GM Mescolotte^2^

#### ^1^Instituto do Coração de Presidente Prudente, Presidente Prudente, Brazil; ^2^Universidade do Oeste Paulista, Presidente Prudente, Brazil

**Introduction**

The growth in the complexity of health care has been accompanied by the need for greater collaborative actions and sharing of information in intensive care units (ICU). The incorporation of daily rounds by a multidisciplinary care team allows better decision making, which results in the improvement of quality of care. The objective of this study was to determine the impact of a multidisciplinary healthcare team on mortality and readmissions of CICU patients.

**Methods**

The database from a cardiac ICU of a medium-sized hospital in Brazil was analyzed to conduct this study. Admissions during the years of 2013 and 2015 were compared, using variables of equity and performance. In 2013, daily rounds were conducted by physicians, nurses and a physiotherapist; whereas, in 2015 the multidisciplinary round was fully equipped with the additional presence of phonoaudiologists, pharmacists, dentists and nutritional professionals. Data were collected from EPIMED MONITOR system and statistically analyzed using EPI INFO, version 3.5.2 software. We considered two tailed test with p < 0.05 significant, and confidence intervals of 95% (95% CI) were used for multivariate logistic regressions.

**Results**

The populations analyzed during 2013 and 2015, respectively, were characteristically similar: number of admissions (715 and 758), mean age (67.9 ± 13.8 and 67.8 ± 13.4), higher proportion of male patients (54.71% and 59.07%), three main diagnoses (coronary angiography with stent, unstable angina, acute ST elevation myocardial infarction). However, during admission the 2015 group averaged a higher SAPS3 score (42.6 ± 11.6 against 40 ± 13.3), indicating these patients were significantly more severe (T statistic = 4.0530; p = 0.0001). The rate of readmissions was 22.08% without the multidisciplinary team and 11.20% when this assistance was implemented, a significant decrease (OR = 0.4452 95%CI = 0.3343 - 0.5930 p < 0.001). Readmissions within 24 hours decreased from a total of 5 to none between 2013 and 2015 (p = 0.021). The mortality rate (13.3% and 9.5%) was also significantly lower in the group exposed to the multidisciplinary healthcare team (OR = 0.6850 IC95% = 0.4949-0.9480 p = 0.0219).

**Conclusions**

Despite the greater severity of patients in the 2015 group, we observed lower mortality and readmission rates in the group of patients attended by a multidisciplinary healthcare team.

**Reference**

Kim M, Barnato AE, Angus DC, Fleisher LF, Kahn JM. The Effect of Multidisciplinary Care Teams on Intensive Care Unit Mortality. Arch Intern Med 2010; 170(4):369–376.

## P304 Intensive care networking: setting objective measures for referral-the paediatric burn matrix

### H Bangalore^1^, E Borrows^1^, D Barnes^2^

#### ^1^Great Ormond Street Hospital & St Andrews Burns Centre, Chelmsford, United Kingdom; ^2^St Andrew´s Burns Centre, Chelmsford, United Kingdom

**Introduction**

Current risk of mortality scores such as PRISM or PIM are not suitable as trigger points for transfer between intensive care units providing different levels of support. St Andrew’s Burns Centre at Chelmsford is a unit located remotely from a tertiary Paediatric Intensive Care Unit (PICU) but provides specialised care to children with severe thermal injuries. The intensive care provision is limited to short term invasive mechanical ventilation and inotrope support but does not provide treatment such as High Frequency Oscillation or renal replacement therapy. In order to facilitate better communication between our burns intensive care unit and a distantly located tertiary PICU, a scoring system was implemented. This was developed as an objective measure to identify children requiring escalation of care beyond the intensive care provision capabilities of St Andrew’s Burn’s Centre. The Paediatric Burn Matrix was introduced in January 2014 to help guide such decisions and balance the risk of transferring these critically ill children across significant distances.

**Methods**

This was based on: age of the child, number of organ failures, expected duration of ventilation for respiratory failure and the percentage of full thickness burn. The scores range from 4 to a maximum 12. A score of 8 or less meant the provision of care was deemed to be appropriate to the capabilities of the Burns Centre. Scores of 9 to 11 would trigger a discussion with a PICU tertiary care centre and a score of more than 12 mandated move to another centre. To demonstrate compliance of service provision, the audit of the implementation of the score was done from January 2014 over a nearly two year period.

**Results**

There were 25 admissions with thermal injuries admitted between January 2015 and November 2015. All children were scored two times a day on the ward rounds. The mean age of the children was 3.9 years with a mean percentage of burn injury of 22.2%. A score of more than 8 triggering a discussion was noted in three children. All three children were discussed with the tertiary PICU. None of the children had a score more than 12. One child was transferred to the tertiary centre and the other children remained on site. There was no mortality in this period.

**Conclusions**

Such scoring systems may be difficult to validate but useful to provide an objective, safe auditable pathway for referral triggers.

## P305 Long-term outcomes and healthcare resource utilization of patients admitted to Brazilian public intensive care units: a populational study

### V Ferreira^1^, L Azevedo^1^, G Alencar^2^, A Andrade^3^, A Bierrenbach^1^

#### ^1^Research and Education Institute, Hospital Sirio-Libanes, São Paulo, Brazil; ^2^University of Sao Paulo, Sao Paulo, Brazil, ^3^Federal University of Goias, Goiania, Brazil

**Introduction**

Studies on long-term outcomes of critically ill patients in developing countries are scarce, especially at a populational level. Our purpose is to describe long-term outcomes and readmissions of critically ill patients admitted to public Brazilian intensive care units (ICU).

**Methods**

Retrospective cohort study using national hospitalization database of adult patients admitted to public hospitals in 10 Brazilian state capitals in 2006 and 2007. ICU hospitalized patients were paired to ward patients by frequency matching in a 1:2 ratio according to postal code and semester of index admission. Hospitalization records were linked through a deterministic linkage to data from national mortality information system from 2005 to 2011. Long-term mortality was defined as occurring after discharge of the index hospitalization and multiple Cox regression was used correcting for age, gender, presence of cancer, hospital characteristics and surgical status.

**Results**

We included 324,594 patients with 108,302 admitted to ICUs and 216,292 admitted to wards at index hospitalization. ICU patients were older (56.7 ± 18.4 vs 45.7 ± 19.4 years, p < 0.001), had increased hospital length of stay (14.0 ± 15.7 vs 5.7 ± 10.4 days, p < 0.001) and hospital mortality (18.5% vs 3.6%; p < 0.001) as compared to ward patients. Hospital readmissions (20.9% vs 16.8%, p < 0.001) and ICU readmissions (6.6% vs. 1.2%, p < 0.001) in one year were more frequent in previous ICU patients. Mortality rate up to one year was 14.3% for ICU patients and 3.9% for non-ICU patients (p < 0.001). The crude hazard ratio for mortality up to 90 days was 5.8 (CI95% 5.6-6.1) and up to one year was 3.9 (CI95% 3.8-4.0). We found a significant interaction between surgical status and mortality for ICU patients. Adjusted hazard ratios (HR) for mortality up to 90 days and one year were 4.1 (CI95% 3.6-4.6) and 2.7 (CI95% 2.5-3.0) for surgical patients, and 5.3 (CI95% 5.1- 5.5) and 3.4 (CI95% 3.3-3.5) for non-surgical ones.

**Conclusions**

Patients discharged from Brazilian public ICUs have increased long-term mortality and hospital readmissions up to one year after discharge. The burden of critical illness is more pronounced in the first ninety days after discharge.

## P306 Animal assisted therapy as an adjunctive therapy for the care of critically ill patients

### L Tadini Buoninsegni, M Bonizzoli, L Cecci, M Cozzolino, A Peris

#### Careggi Teaching Hospital, Florence, Italy

**Introduction**

A strong bond exists between humans and animals. Various studies have shown significant positive psychological and physiological effects on humans. The purpose of this study was to investigate the potential use of this relationship as an adjunctive therapy to alleviate patient stress and increase comfort with care during stressful experience as intensive care unit (ICU) admission. Owing to important medical and technological advances in the treatment of the critically ill, survival of a critical illness is now high. Nevertheless this had created a subpopulation of patients whose need for ICU services and life support is prolonged with an increased risk to develop adverse psychological outcomes. Animal assisted therapy (AAT) is an intentional healing modality used to achieve therapeutic goals through a facilitated interaction between patients and animals, with specific behavioural characteristics, accompanied by trained handler. Interventions direct to reduce stress due to an extended admission to ICU could help to decrease psychological disorders and related morbidity.

**Methods**

Our observational study included critical patients admitted before the introduction of animal assisted therapy (AAT) in acute care (control group) and patients who were involved in an AAT program (intervention group). Individual session of AAT took place twice a week for 45 minutes at patient bed. Intensive Care Psychological Assessment Tool (IPAT) was used to assess psychological distress during ICU stay and the Emotion Thermometers tool (ET) to monitor the patient emotional state pre- and post AAT session. The primary outcome was to determine the effect of AAT on emotional symptoms and memories of ICU stay.

**Results**

Control and intervention groups showed similar demographic and clinical characteristics. A total of 23 patients were enrolled in the control group and 20 in the intervention group (mean age 57 ± 15.6 years). Mean duration of mechanical ventilation was 37 ± 13.5 days and ICU stay was 44 ± 22 days. Patients in the intervention group showed a decreased rates of stress (8.8%vs17.2%) and sadness (6.4%vs15.4%) on the basis of ET scores measured pre- and post AAT; they also felt more positive immediately following AAT (85% of pts enrolled). Patients who received AAT reported greater improvement in their mood, perceived level of feeling homeless and reduced upsetting memories of intensive care on the basis of IPAT scores.

**Conclusions**

Our results suggest that implementing ICU treatment with AAT may help critically ill patients recover from this stressful experience. Our findings indicate a new focus for study of its adjunctive therapeutic role in the care of critically ill especially with prolonged ICU stay.

## P307 Family satisfaction in the critical care unit: does length of stay play a role?

### J Lindskog, K Rowland, P Sturgess, A Ankuli, A Molokhia

#### Lewisham & Greenwich NHS Trust, London, United Kingdom

**Introduction**

The aim of this survey is to evaluate whether patient length of stay affects family satisfaction in critical care.

In 2011 the NHS Outcome Framework recognised “ensuring people have a positive experience of care” as a key domain of quality [1]. In critical care family members play a crucial role in patient care. Meeting their emotional needs is essential to delivering good quality care.

Family Satisfaction in the Intensive Care Unit FS-ICU has been developed and validated for assessing families’ satisfaction with care [2]. Longer length of critical care stay has been associated with greater physical, social and psychological impact on family members.

**Methods**

An anonymous, prospective survey was run across two critical care sites over a 4-week period. The questionnaire utilized was based on the FS-ICU questionnaire and was modified to include a question regarding length of patient stay. Questionnaires were distributed to family members of patients once they were deemed fit for discharge from critical care. A maximum of 4 questionnaires were issued per patient, consistent with the 2014 FREE study [3].

**Results**

57 responses were received. Mean length of stay was 10.2 ± 25.1 days with a median length of stay of 3.5 days. Results were divided by length of stay using the median as cut-off, giving 28 responses in the short stay group and 30 responses in the long stay group. Table 28. shows results as percentage of positive answers divided into short and long stay.

**Conclusions**

These results show that longer length of critical care stay correlates with higher rates of family satisfaction, particularly with the care and communication received from doctors and the environment. Longer length of stay allowing relatives to establish better rapport with doctors and become more familiar with the critical care environment may account for this finding.

**References**

1. Departmemt of Health. NHS Outcomes Framework 24–28, 2010.

2. Heyland et al. Crit Care Med 30:1413–1418, 2002.

3. Wright S et al. ICNARC Free V1:3, 2014.

**Table 28 (abstract P307).** See text for description

Satisfaction with	Short stay (%)	Long stay (%)
Nursing care	96	94
Nursing communication	93	91
Doctor´s care	88	94
Doctor´s communication	75	81
Critical care atmosphere	79	91
Overall care	84	91

Legend: Results by selected domains, divided by length of stay

## P308 Effectiveness and safety of an extended visitation policy in the ICU: a before and after study

### R Rosa^1^, T Tonietto^1^, A Ascoli^1^, L Madeira^1^, W Rutzen^1^, M Falavigna^1^, C Robinson^1^, J Salluh^2^, A Cavalcanti^3^, L Azevedo^4^, R Cremonese^1^, D Da Silva^1^, A Dornelles^1^, Y Skrobik^5^, J Teles^6^, T Ribeiro^1^, C Eugênio^1^, C Teixeira^1^

#### ^1^Hospital Moinhos de Vento, Porto Alegre, Brazil; ^2^Instituto D´Or de Pesquisa e Ensino, Rio de Janeiro, Brazil; ^3^Hospital do Coração, São Paulo, Brazil; ^4^Universidade de São Paulo, São Paulo, Brazil; ^5^McGill University, Montreal, Canada; ^6^Hospital Universitário de Goiânia, Goiânia, Brazil

**Introduction**

Few studies have evaluated the impact of distinct visitation policies on patient outcomes, and, specifically in the context of critical care, no study has evaluated the potential of an extended visitation model in the prevention of delirium and its associated outcomes. The present study aimed to evaluate the impact of an extended visitation model (EVM) compared to a restricted visitation model (RVM) on the incidence of delirium and associated outcomes among ICU patients.

**Methods**

A prospective before and after study was conducted at the 31-bed medical-surgical ICU of a tertiary hospital in southern Brazil. The main study intervention was change of visitation policy practices from a restricted (4.5 hours/day) to an extended model (12 hours/day). All patients > =18 years with an expected length of stay > =24 hours consecutively admitted to the ICU from May 2015 to November 2015 were evaluated. The primary outcome was incidence of delirium, assessed twice daily using the confusion assessment method for the ICU. The secondary outcome measures included all-cause ICU mortality; length of ICU stay; length of profound sedation during ICU stay; any ICU-acquired infection; ICU-acquired bloodstream infection, pneumonia and urinary tract infection.

**Results**

In total, 286 patients were enrolled in the study (141 admitted during the RVM and 145 during the EVM). The median duration of visits increased from 133 min (interquartile range [IQR], 97.7-162.0) in the RVM to 245 min (IQR, 175.0-272.0) in the EVM (p < 0.001). A total of 14 (9.6%) patients developed delirium in the EVM compared with 29 (20.5%) in the RVM (adjusted relative risk [aRR], 0.55; 95% confidence interval [CI], 0.29-0.95; p = 0.03). The median length of ICU stay was 3 days (IQR 2-4) in the EVM and 4 days (IQR 2-6) in the RVM (aRR, 0.89; 95% CI, 0.79-0.99; p = 0.04). The length of profound sedation during ICU stay, and the rate of ICU-acquired infections and all-cause ICU mortality did not differ significantly between the two study groups.

**Conclusions**

Among ICU patients, an EVM was associated with a significant reduction in the incidence of delirium. There was a significant reduction in the length of ICU stay with the EVM as well. Multicenter randomized studies are needed to replicate these findings.

## P309 Assessing the level of stress and anxiety in family members of patients hospitalized in the intensive care units

### M Zarei^1^, H Hashemizadeh^2^

#### ^1^North Khorasan University of Medical Sciences, Bojnurd, Iran; ^2^Quchan Branch, Islamic Azad University, Quchan, Iran

**Introduction**

The present study has been conducted with the aim of assessing and comparing the stress, anxiety and depression in family members of patients hospitalized in the intensive care unit.

**Methods**

This descriptive- analytic study is of comparative type that was conducted on 200 family members, who were the first degree relatives of patients hospitalized in Quchan's Musa-ibn-Jafar hospital in 2013. Data were collected by using the depression, anxiety and stress scale (DASS) and they were analyzed by T-test and chi-square test.

**Results**

Regarding the study of the psychological reactions of family members of patients hospitalized in intensive care units of ICU and CCU, it was found that the mean score of anxiety, stress and depression, in family members of patients hospitalized in the ICU, were respectively 19.10 ± 5.11 and 20.55 ± 4.730 and 18.61 ± 5.26 and in family members of patients hospitalized in the CCU, these scores were 16.05 ± 5.52 and 17.02 ± 5.43 and 15.63 ± 5.10, respectively.

**Conclusions**

Therefore, the supports provided by nurses for families of patients, through the creation of appropriate therapeutic relationships and helping them to discharge their feelings and emotions, creates hope in families and reduce their stress levels.

**References**

**Table 29 (abstract P309).** See text for description

Unit	ICU	CCU	Result of t-test
Characteristics	Mean ± SD	Mean ± SD	
Depression	18.61 ± 5.26	15.63 ± 5.10	P < 0.001
Anxiety	19.10 ± 5.11	16.05 ± 5.52	P < 0.001
Stress	20.55 ± 4.73	17.02 ± 5.43	P < 0.001

Legend

## P310 Intraosseous sampling in medical emergencies

### M Eriksson^1^, G Strandberg^1^, M Lipcsey^1^, A Larsson^2^

#### ^1^Surgical Sciences, Uppsala, Sweden; ^2^Medical Sciences, Uppsala, Sweden

**Introduction**

Intraosseous (IO) administration of fluids and drugs is established when vascular access is difficult to achieve. However, IO needles may be used for sampling of bone marrow aspirates. We wanted to evaluate whether such aspirates may reflect results obtained from conventional blood sampling in an experimental shock model.

**Methods**

Anaesthetized pigs were given E. Coli endotoxin over a 6 hour period. IO access was achieved in the tibial bone (EZ-IO®, Teleflex Medical, Morrisville, NC, USA). Blood gases were analyzed by Point – of – Care – Technology (POCT). A broad spectrum of blood tests used in medical emergencies were analyzed at a centralized laboratory.

**Results**

Endotoxemia caused an expressed decrease in left ventricular stroke work index, which was reduced by approximately 80%. IO pH levels were approximately 0.1 units lower in IO samples than in arterial blood. Base Excess was lower in IO versus arterial samples. PCO2 levels in IO samples were clearly higher than those seen in arterial blood. IO lactate was also higher in IO samples than in arterial blood [1]. Troponin I increased, both in IO samples and in venous ones. At 1 hour mean troponin I was more than doubled with very small inter-individual differences. The levels of troponin I in IO aspirates differed approximately 1% compared to plasma obtained from arterial as well as venous samples after 1 hour of endotoxemia. At 2 hours, all mean troponin I levels were raised approximately 5 fold. At 2 hours and 3 hours troponin I in IO aspirates was 20% lower than in venous plasma. The levels of troponin I in bone marrow aspirates more or less reached a plateau at 3 hours, whereas troponin I in plasma samples continued to increase. Still, all mean levels of troponin I were markedly elevated [2]. We found no clinically relevant average differences between ALAT, ALP, ASAT, ALAT, CK, and gamma-GT in IO and blood samples, respectively. A possible difference in IO and blood samples may be considered [3].

**Conclusions**

Although IO and blood samples differ somewhat, there seems to be a consistency regarding these differences. The direction of bias can be predicted. Thus, when conventional blood samples are difficult to achieve, IO samples may help to steer initial therapy. When whole blood is analysed e.g. blood gasses, POCT is recommended.

**References**

1. Strandberg G et al. AAS 58: 337–44, 2014

2. Eriksson M et al. Clin Lab 61: 825–9, 2015

3. Eriksson et al. Scand J Lab Clin Invest 29:1–4, 2016

## P311 Peripherally inserted central catheter (PICCs) in critically ill patients: complications and risk factors

### M Lignos^1^, E Crissanthopoulou^1^, K Flevari^1^, P Dimopoulos^2^, A Armaganidis^1^

#### ^1^Attiko University Hospital, Haidari, Greece; ^2^Beng MSc in Bioengineering, Athens, Greece

**Introduction**

Peripherally inserted central venous catheters have been introduced several years earlier in order to treat patients receiving chemotherapy, parenteral nutrition or prolonged antibiotic courses. In the last decades the scientific community has placed its focus on the potential utility of peripherally inserted catheters (PICCs) in critically ill patients. The present study examines the complications of the PICCs’ use in critically ill patients and their related risk factors.

**Methods**

This is a retrospective study conducted in a general ICU of a tertiary University hospital. Candidates for the insertion of peripheral catheters were all patients > 18 years who were stable and did not require the use of vasopressors. Exclusion criteria were the severe renal impairment, sepsis or bacteremia and the limited vessel diameter (<4 mm). All PICC’s were inserted by an ICU physician under ultrasonographic guidance. Location of catheter tip was always confirmed by an X – ray of the chest.

**Results**

The sample of the study consisted of 68 subjects from 15 to 85 years old (58 ± 18). The mean duration of the catheter insertion was 27 days. The internal medicine and surgical patients were equally distributed, while a small number of cases considered to belong to both categories. The most common cause of admission has been neurological disorders and various types of malignancies (19.1 and 16.2%). In the 63% of the cases (N =43) the catheters remained in their position and no replacement was needed (end of therapy or hospitalization). In 12 cases the catheter was removed because of suspected infection; however in the 83% of them infection was not confirmed. Except 4 cases (6%) of accidental catheter removal and 3 cases (4.5%) where corrective manipulations were needed no other of the reported complications were recorded (Table 30).The incidence of catheter related infection (CR-BSI) in this study was 4.4/1000 catheter-days (8 cases) up to half of the cases the pathogens were Gram positive cocci. No association was established upon sex, age or the cause of patients’ admission (p > 0.05). On the contrary, the duration of catheterization was statistically significant as regards CR-BSI occurrence (RR:1.035).

**Conclusions**

Ultrasound guided, PICC insertion minimizes the occurrence of many of the complications. CR-BSI remain the most common complication and is mainly associated with the length of stay. Large multicenter studies are needed nevertheless, in order to draw solid general conclusions about the usefulness of the PICCs as an alternative option to standard central venous catheters, in selected ICU patients.

**Table 30 (abstract P311).** See text for description

	%(N)
art. puncure	0(0)
thrombosis	0(0)
cath.migration	4.5(3)
accid.removed	6(4)
CR-BSI	12(8)

Legend: Complications of the piccs use in critically ill patients

## P312 Feasibility of subclavian vein ultrasound imaging for central venous catheter placement

### JG Golub^1^, AM Markota^1^, AS Stožer^2^, AS Sinkovič^1^

#### ^1^University Medical Centre Maribor, Maribor, Slovenia; ^2^University of Maribor, Maribor, Slovenia

**Introduction**

Ultrasound guided central venous catheter (CVC) insertion is supported by guidelines, however, the majority of studies have evaluated the use of ultrasound in internal jugular and femoral veins [1]. The objective of our study was to evaluate feasibility of subclavian vein (SV) ultrasound imaging for ultrasound-guided central venous catheter placement.

**Methods**

We performed a prospective study on 25 successive critically ill patients in the Medical Intensive Care Unit, University Clinical Centre Maribor, Slovenia. With the use of a linear ultrasound probe (probe L12-3, ultrasound machine HD11 XE, Philips Ultrasound, Andover, MA, USA), we evaluated SV ultrasound visibility in short axis in the clavicopectoral triangle of infraclavicular space on both sides in mechanically ventilated and non-ventilated patients.

**Results**

Of the 25 patients included in the study, 17 (68%) were males, mean age was 66,3 ± 11,7 years. 13 patients (52%) were intubated and mechanically ventilated. Altogether, we evaluated 50 locations. SV was visible in 41 locations (82%). In one patient, SV was not visible on neither side. There was no statistically significant difference in SV ultrasound visibility between the right and the left side (McNemar´s test, p = 0.45). SV visibility was significantly better in mechanically ventilated patients. In the group of mechanically ventilated patients, SV was visible on both sides in 96,1% of cases, whereas in the spontaneously breathing patients it was visible in 66,7% of cases (Barnard´s test p = 0.01).

**Conclusions**

According to our small prospective study, SV visibility for ultrasound-guided CVC insertion is possible in most patients on both sides, and successful visualization is more frequent in the group of mechanically ventilated patients.

**Reference**

1. Frankel HL et al. Crit Care Med 43: 2479–502, 2015

## P313 Central venous cannulation in ed patients - time to rethink the standards

### H Rüddel, C Ehrlich, CM Burghold, C Hohenstein, J Winning

#### Jena University Hospital, Jena, Germany

**Introduction**

Patients in emergency departments (ED) frequently need catheterization of the internal jugular vein (IJV) for e.g. application of drugs, fluids, blood products or taking of blood samples. Following the guidelines the application of a central venous catheter is typically performed while the patient is in Trendelenburg-Position (TP) (1). It is clinical every-days experience that this head-down positioning is freuently not tolerated by critically ill patients. Yet, these patients often require central venous cannulation. We therefore performed a two-step trial to investigate the tolerance of TP in critically ill patients and 30° elevation position (EP) with non-invasive ventilation (NIV) as an alternative. Both issues have not been investigated in critically ill patients before.

**Methods**

For both steps, all adult, non-intubated patients brought to the ED by ambulance were eligible. Approval of the treating team and consent was obtained.

Step a: 117 patients were screened for contraindications to TP, and if non were present positioning was performed for a maximum of 10 minutes. Rate of contraindications and abandonement of positioning by the patients was recorded.

Step b: 91 patients had an ultrasound measurement of the cross-sectional area (CSA) of the right IJV in TP, supine, and EP with and without NIV in all positions.

**Results**

Step a: In 45 of 117 patients (38%), TP was contraindicated (n = 21, 18%) or positioning was either impossible or not tolerated for 10 minutes (n = 24, 21%). Reasons are shown in Tables 31 and 32.

Step b: Without NIV, the CSA of the IJV was largest in TP (0.99 ± 0.66 cm^2^) compared to supine (0.57 ± 0.58 cm^2^) and EP (0.25 ± 0.41 cm^2^). CSA in EP with NIV was significant larger than without NIV (0.62 ± 0.70 cm^2^). NIV was not tolerated by 2 patients (2%). In 62 patients (68%), EP with NIV showed a CSA > =0.4 cm^2^, indicating a safe puncture (2).

**Conclusions**

Guideline´s recommendation for central venous catheterizatin in TP are not evidence-based and not feasible in critically ill ED patients and should be reviewed. EP with NIV offers a well-tolerated, feasible and safe alternative.

**References**

(1) American Society of Anesthesiologists Task Force on Central Venous, A., et al., Practice guidelines for central venous access: a report by the American Society of Anesthesiologists Task Force on Central Venous Access. Anesthesiology, 2012. 116(3): p. 539–73.

(2) Lichtenstein D, Saifi R, Augarde R, et al. The Internal jugular veins are asymmetric. Usefulness of ultrasound before catheterization. Intensive Care Med 2001; 27:301–305

**Table 31 (abtsract P313).** See text for description

Contraindications: 21	
- Dyspnea: 4	
- Acute coronary syndrome: 4	
- Cardiac decompensation: 3	
- Spinal injury of lumbar vertebrae: 2	
- Nausea: 1	
- Syncope of unknown origin: 1	
- Glioblastoma with edema: 1	
- Severe ascites, suspicion of Ileus: 1	
- Angina pectoris: 1	
- Subdural hematoma: 1	
- Post operation (intracranial):	
- Intracerebral hemorrhage: 1	

Legend: Contraindications to TP

**Table 32 (abtsract P313).** See text for description

Termination during positioning: 10	Termination of TP: 14
- Thoracic tightness, dyspnea: 2	- Vertigo: 5
- Vertigo: 2	- Nausea: 3
- Pain in the inguinal region (hip fracture): 1	- Dyspnea: 1
- Nausea: 1	- Severe pain right shoulder: 1
- Anxiety to fall off the bed: 1	- Pain lumbar vertebrae: 1
- Flank pain: 1	- Pain cervical vertebrae: 1
- Increased feeling of intracranial pressure: 1	- Feeling of intracranial pressure: 1
- Severe pain in the lumbar region: 1	- Indisposition (not definable): 1

Legend Termination of positioning or TP

## P314 Increase in the diameter of the subclaviar vein according to the arm’s position: a preliminary echographic study

### W Sellami, Z Hajjej, M Bousselmi, H Gharsallah, I Labbene, M Ferjani

#### Military Hospital of Tunis, Tunis, Tunisia

**Introduction**

The canulation of the subclaviar vein may seem difficult due to the proximity of the clavicle and the pleura. Changing the arm position amend the anatomical relationship between the clavicle and the vein and could amend its access. To confirm this hypothesis, we conducted a preliminary anatomical study on the hospitalized patients. Its aim was to compare the subclaviar vein diameter in two arm positions: neutral position (PN) and 90° abduction and 90° external rotation position (ARE).

**Methods**

This prospective study was conducted during 3 months. Patients older than 18 years old, hospitalized consecutively in the ICU were included. A unique expert operator performed the ultrasound exams with a vascular probe. The probe was placed perpendicularly to the skin, parallel to the clavicle in its median third portion. The measures were realized for the right side exclusively. At each time, after obtaining a cross section of the vein, the depth and the diameter of the vein were measured, as well as the distances vein-pleura and vein-artery.

**Results**

The subclaviar vein diameter increased significantly after the arm mobilization (11 mm vs 14 mm, p = 0.03). The artery, the pleura and the blachial plexus were viewed as frequently in both positions without a significant difference in the distance between these structures and the subclaviar vein.

**Conclusions**

Changing the position of the arm in 90° abduction and 90° external rotation enhances significantly the cross section area of the vein which can make the ultrasound guided cannulation easier and safer.

## P315

### Withdrawn

## P316 Analysis of closing frequency and reasons for closure of stroke units in Munich, Germany

### J Sattler^1^, D Steinbrunner^2^, H Poppert^1^, G Schneider^1^, M Blobner^1^, KG Kanz^1^, SJ Schaller^1^

#### ^1^Klinikum Rechts der Isar der TUM, Munich, Germany; ^2^Branddirektion München, Munich, Germany

**Introduction**

According to the premise “time is brain“,[1] an immediate admission of stroke patients to the closest specialized stroke unit is crucial for the best outcome. Hence, delays of further treatments caused by closure of the next stroke unit might affect outcome. Munich provides 63 monitored beds in 6 stroke units. The following study analyses frequency and reasons of closing of strokes units in Munich, Germany.

**Methods**

After Ethics Committee approval, retrospective analysis of IVENA eHealth data program regarding admission to and closing of Munich's stroke units between January 2014 and June 2016 was conducted. Calculation was done using chi-square test with STATA14.

**Results**

From January 2014 to June 2016, 7802 patients (2014: 2816 / 2015: 3180 / Jan-Jun 2016: 1806) were assigned to a stroke unit in Munich. 78.69% of the patients were assigned to a stroke unit with neuroradiology.

**Fig. 40 (abstract P316).**
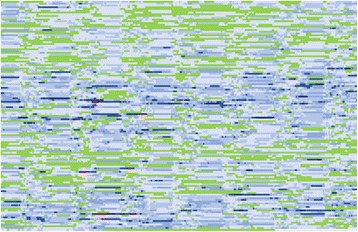
Amount of closed stroke units (*green* = 0-1, *blue* = 2-5, *red* = 6)

Time of closing of at least one stroke unit was increasing over time (2014: 71.88% / 2015: 74.99% / Jan-Jun 2016: 84.51%, p < =0.001, Fig. 40). Furthermore, we see an increase of more than one closed stroke unit at a time (2014: 32.40%, 2015: 40.30% / Jan-Jun 2016: 50.60%, p < = 0.001) as well as all stroke units closed (2014: 0.00%, 2015: 0.10% / Jan-Jun 2016: 0.14%, Fig. 40). Closing of at least one, more than one and all stroke units happened significantly more often during night time from 22:00-06:00 and less often on weekends (p < =0.001 for all scenarios). One of the hospitals was significantly less often signed off compared to the other five. Main reasons given for closing was no capacity (either full or over occupancy; 2014: 85.26% / 2015: 83.09% / Jan-Jun 2016: 88.78%).

**Conclusions**

Since closing times are increasing significantly in Munich with the main reason of "no further capacity", an undersupply of stroke beds in Munich seems to be present.

**Reference**

[1] Saver, Jeffrey L. Time Is Brain – Quantified, Stroke. 2006 Jan;37(1):263–6.

## P317 The management of acute allergic reactions at the emergency department - room for improvement?

### K Apap, G Xuereb

#### Mater Dei Hospital, Msida, Malta

**Introduction**

This study was conducted to assess and evaluate the management of patients presenting to the accident and emergency (A&E) department with an acute allergic reaction.

**Methods**

A retrospective review of all patients over the age of 18 presenting to the A&E department at Mater Dei Hospital, Malta with acute allergic reaction between April-June 2016 were included in the study. Data was collected from the case notes and electronic records and analysed. Local Acute Allergic Reaction guideline (February 2012) was used to establish best practice for the management of acute allergic reactions.

**Results**

A total of 76 patients were identified; of these, 22 were admitted (29%), 54 were discharged (71%). The causative allergen was identified in 57 (75%) patients. The most common triggers were antibiotics (23%), seafood (12%) and non-steroidal anti-inflammatory drugs (NSAIDs) (7%). Anaphylactic shock risk was assessed in 73 (96%) patients. Vital signs on admission were monitored in 75 (99%) patients; repeat vital signs were monitored hourly in 4 (5%) patients. Repeat vital signs four-hourly were monitored in 20 patients (28%; of the 71 remaining patients). 60 (79%) patients received histamine 1 (H1) receptor antagonist, 39 (51%) patients received histamine 2 (H2) receptor antagonist and 60 (79%) patients received steroids. On discharge, 50 (66%) patients were educated regarding avoidance of the causative allergen. Along the course of treatment, 2 patients were discharged against medical advice. 19 (26%) patients were referred for specialist follow-up and 13 (21%) patients were referred for general practitioner (GP) follow-up.

**Conclusions**

The study findings show that the management of patients presenting with an acute allergic reaction at the A&E department should be optimised with the guidelines in order to provide better care. Recommended areas of improvement in the management of these patients include more regular monitoring of the vital signs and increased patient education regarding avoidance of the causative allergen, for instance through the introduction of educational leaflets. Possible ways to improve management of patients presenting to A&E with acute allergic reactions include the implementation of a pro-forma sheet, establishment of a referral criteria, continuous medical education to the emergency staff and the updating of hospital guidelines according to the latest studies.

## P318 The management of patients with alcohol intoxication at the emergency department - the pitfalls

### G Xuereb, K Apap, L Massa

#### Mater Dei Hospital, L-Imsida, Malta

**Introduction**

The aim of this study was to evaluate the management of patients presenting with alcohol intoxication at the accident and emergency (A&E) department.

**Methods**

A retrospective analysis of all patients presenting to the A&E department at Mater Dei Hospital, Malta with alcohol intoxication within the period of April-June 2016 was conducted. Data was gathered from the electronic records and case notes. The Local Management of Alcohol Intoxication guideline (February 2012) was used to determine best practice for the management of alcohol intoxication.

**Results**

A total of 104 patients were identified; of these, 33 were admitted (31.7%), 62 were discharged (59.6%), 4 were discharged against medical advice (3.8%) and 5 patients failed to turn up (4.8%). 35 (34%) patients presented to hospital on a Sunday, with the most frequent time of admission being between 00:00 and 04:00 (47 patients; 45%). The blood alcohol level was calculated in 87 (91.6%) patients. The repeated blood alcohol level was documented in 33 (34.7%) patients. Baseline investigations were taken in 88 (92.6%) patients. The core temperature was documented in 53 (55.8%) patients. The Glasgow Coma Scale (GCS) was assessed and documented in 34 (35.8%) patients and of these. 1 patient was found to have a GCS of 6, and no anaesthetist was consulted. The alert, voice, pain unresponsive scale (AVPU) was the chosen alternative in 15 (15.8%) patients. Activated charcoal was not administered in any of the cases, even in cases with findings of concomitant drug abuse. Intravenous (IV) fluids were given in 83 (87.1%) patients. 23 (24.2%) patients were noted to be chronic alcoholics, with thiamine administered in 19 (82.6%) of these patients. Suicidal intentions were noted in 23 (24.2%) patients.

**Conclusions**

Analysis of the study findings suggests that optimisation with the guidelines would provide better care to patients presenting with alcohol intoxication to the A&E department. Recommended areas of improvement in the management of these patients include increased psychiatric referral, improved liaison with the anaesthesia department in cases of a GCS lower than 8, administration of activated charcoal in patients with findings of concomitant drug use and routinely taking repeated alcohol level investigations. The management of patients with alcohol intoxication may be improved through the continuous medical education of emergency staff, the implementation of a pro-forma sheet and a more recently updated set of hospital guidelines based on recent studies.

## P319 The management of renal colic at the emergency department - are mistakes being overlooked?

### G Xuereb, K Apap, L Massa

#### Mater Dei Hospital, L-Imsida, Malta

**Introduction**

The study was performed to analyse the management of patients presenting with renal colic at the accident & emergency (A&E) department.

**Methods**

A retrospective evaluation of all patients aged over 18 presenting to the A&E department at Mater Dei Hospital, Malta with renal colic within a 3-month period from April-June 2016 were included in this study. Electronic records and case notes were used for the collection of the data. The Royal College of Emergency Medicine 2014 guidelines were used to establish best practice for the management of renal colic.

**Results**

A total of 139 patients were identified; of these, 57 (41%) were admitted, 69 (50%) were discharged, 7 (5%) were discharged against medical advice and 6 (4%) failed to turn up. The initial pain score was documented in 38 (30%) patients, while a repeated pain score was documented in 12 (10%) patients. Analgesia was given within 20 minutes to 23 (18%) patients at the A&E department. 15 (12%) patients were documented to have administered analgesia prior to arrival at A&E (due to self-administered analgesia and/or analgesia administration at local health centres prior to referral).The average time to administer analgesia was 53.4 minutes. Baseline investigations were taken in all of the 126 (100%) patients. A computed tomography scan of kidneys, ureters, bladder (CT KUB) was performed in 103 (82%) patients, with an average time of 120 minutes for the scan to be performed. 73 (58%) patients were followed up at urology out patients.

**Conclusions**

The management of patients presenting with renal colic at the A&E department should be optimised with the guidelines in order to provide better care for renal colic patients. Recommended areas of improvement include the implementation of a local guideline, increased clinician education of the importance of pain score documentation and administration of analgesia within an appropriate period of time. Potential ways to improve management of patients presenting with renal colic include the introduction of a pro-forma sheet, establishment of a standardised referral criteria and continuous education given to emergency staff.

## P320 Effect of noninvasive ventilation on carboxyhemoglobin toxicokinetic after acute carbon monoxide intoxication: a swine model.

### N Delvau^1^, A Penaloza^1^, G Liistro^1^, F Thys^2^, IK Delattre^3^, P Hantson^1^, PM Roy^4^, P Gianello^5^

#### ^1^Cliniques Universitaires Saint-Luc, Brussels, Belgium; ^2^GHdC, Charleroi, Belgium; ^3^UCL, LTAP, Brussels, Belgium; ^4^CHU, Angers, France; ^5^UCL, CHEX, Brussels, Belgium

**Introduction**

Fraction of inspired oxygen (FiO2%) and minute ventilation influence half-life of carboxyhemoglobin (COHb T1/2) after acute carbon monoxide (CO) poisoning.

**Methods**

We compared classical O2 therapy by non-rebreathing mask (NRM) to continuous positive airway pressure (CPAP) using high flow O2 and two procedures of noninvasive positive pressure ventilation (NiPPV) to decrease COHb T1/2. Eight spontaneously breathing pigs sedated by Propofol were exposed several times separated by washout period to 940 ppm CO until COHb level of 30%. CPAP (CPAPBoussignac®,7.5 cmH2O), NiPPV-Vy (Vylife Boussignac®), NiPPV-Leg (Legendair®) [Inspiratory/Expiratory PAP 12/4 cmH2O] were used in a randomized order and compared to NRM [O2 at 15 l/min] and atmospheric air (AA). COHb T1/2 was assessed by non-compartmental analysis. Multiple comparisons were performed by Dunn’s tests. Results are expressed as median [range].

**Results**

**Fig. 41 (abstract P320).**
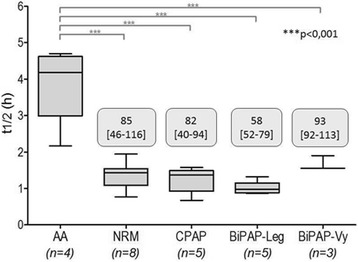
COHb T1/2 (minutes), median [range]

Hemoglobin level, blood pressure, cardiac frequency, lactate, temperature and basal COHb were similar between treatments as well as time to reach 30% COHb (110 min [80-140]), pCO2, pO2 and minute ventilation. FiO2 was 72% [59-83], 54% [49-73], 92% [76-93], 51% [51-53] for NRM, CPAP, NiPPV-Vy, NiPPV-Leg, respectively. COHb T1/2 was significantly higher in pigs under AA than in treated pigs but without significant difference between treatments (Fig. 41).

**Conclusions**

In our swine model of CO poisoning, CPAP or NiPPV did not show a significant decrease of COHb T1/2 compared to NRM. However, a lack of statistical power may explain this absence of significant difference especially with NiPPV-Leg that has shown the lowest COHb T1/2 as compared to NRM (58 vs 85 min). Further studies are required to explore the comparative efficacy of NiPPV in CO poisoning.

## P321 Epidemiology of acute exogenous intoxications

### L Hadîrcă^1^, A Ghidirimschi^1^, N Catanoi^1^, N Scurtov^2^, M Bagrinovschi^1^

#### ^1^National Centre of Prehospital Emergency Medicine, Chisinau, Moldova; ^2^State Medical and Pharmaceutical University "Nicolae Testemitanu", Chisinau, Moldova

**Introduction**

Acute exogenous in children remain a major cause of infant morbidity and mortality.

**Methods**

The study material consisted of 476 medical records of children whit acute exogenous intoxications who were admitted to the emergency department of the Institute of Mother and Child from 5 January 2015 – 12 March 2016.

**Results**

The study found the following from: 288 children received medical care in the emergency department but 188 of them in the life – threatening condition were transferred to the pediatric and toxicology resuscitation department. According to: the first level includes children to 6 years – 255 cases; the second level was registered in children younger 12 years – 97 cases. In the transportation of children emergency health care was provided in 440 cases. The evaluation result of the epidemiologic spectrum of children whit exogenous intoxication showed the following a high prevalence of drug intoxications in 224 children; followed by intoxications with household things in 71 children, alcohol intoxications in 40 cases, mushrooms in 36 cases, carbon monoxide in 32 children; and ethnobotanical intoxications, intoxications with opiates, rodenticides, pesticides, poisonous plants representing each less than 10 cases, unidentified etiology of intoxication in 61 children. At the pre-hospital stage, the emergency department initiated the administration of antidotes in 35 patients, in children with specific symptoms and combinations specific to intoxication.

**Conclusions**

The epidemiological structure of intoxications in children mainly was registered in intoxication whit drugs, followed by household things, while the number of children whit intoxications of unidentified etiology remains high at 12.8%, indicator that justifies the compulsory screening of urine and plasma concentration of toxic substance for administration of antidotes in children whit exogenous intoxications in precocious terms.

## P322 Acute heart failure after oral intake of liquid nicotine: case report

### YS Sohn, YC Cho

#### Soon Chun Hyang University Hospital Seoul, Seoul, South Korea

**Introduction**

This is a report of a patient of an acute heart failure after intake of liquid nicotine used in electronic cigarette. The market for electronic cigarette is growing annually. However, there are not many studies about the toxicity of electronic cigarette nicotine.

**Methods**

A 32 year old female patient was admitted to the hospital with acute heart failure after taking liquid nicotine.

**Results**

A 32-year-old female patient was admitted to the emergency room with dizziness, nausea and abdominal pain after taking 10 mg of liquid nicotine for electronic cigarettes with 2 glasses of soju and a glass of beer, 4 hours prior to the visit.

At the time of admission, her conscious level was drowsy. Her blood pressure was 78/57 mmHg and other vital signs were normal. On laboratory tests, complete blood counts were normal range but the cardiac enzymes were increased to 7.91 ng/mL for CK-MB and 0.266 ng/mL for troponin T and 77.49 ng/mL for myoglobin. The cotinine serum level was also increased to 7069 ng/mL. Initial echocardiography taken after 1 hour of admission showed akinesia in the apex and the anterior wall, hypokinesia in the inferior and the posterior wall and severe LV systolic dysfunction with ejection fraction of 20%. Gastric lavage was done and activated charcoal was administered. The patient was referred to the cardiology department due to acute heart failure caused by nicotine.

Her consciousness was restored within the first day of admission. On the 4th day, the laboratory test showed decrease in cadiac enzymes. Follow up echocardiography showed restored function on the anterior, posterior, and inferior walls and only the apex showed hypokinesia. Ejection fraction was recovered to 65%.

**Conclusions**

As e-cigarettes are becoming more popular, more patients with liquid nicotine overdose are expected. Therefore, it is necessary to study the toxicity and prepare for situations that may occur.

**Consent to publish**

We received a written consent form from the patient.

**Fig. 42 (abstract P322).**
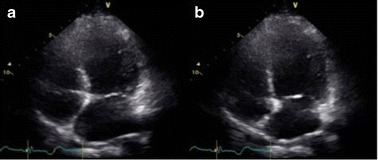
See text for description

**Fig. 43 (abstract P322).**
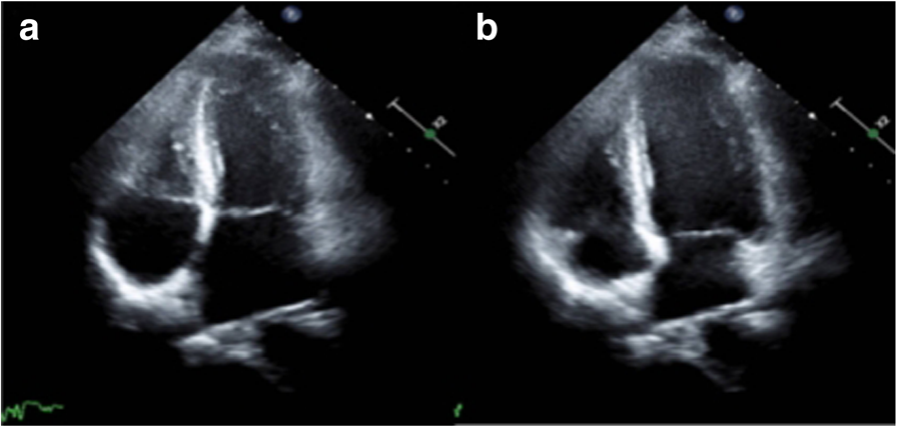
See text for description

## P323 Incidence and management of anaphylaxis in prehospital

### B Golovin^1^, O Creciun^2^, A Ghidirimschi^1^, M Bagrinovschi^1^

#### ^1^National Centre of Prehospital Emergency Medicine, Chisinau, Moldova; ^2^Ministry of Healthcare, Chisinau, Moldova

**Introduction**

Anaphylaxis is a life-threatening syndrome resulting from the sudden release of mast cell- and basophil-derived mediators into the circulation. The incidence of anaphylaxis representing 15 cases per 100 thousand population.

**Methods**

The goals of study are studying of incidence and epidemiology of anaphylaxis. Management of emergency medical assistance in prehospital stage. In accordance with the purpose and objectives of the study were studied 60 patients during the three months of 2016.

**Results**

It was determined that the age of patients with anaphylaxis is 18- 75 years (±46 years), of which women from 38 to 63.3%, men 22 to 36.6%. According to etiology triggers the most frequently cause were food in 24 cases - 40%, drugs in 18 cases- 30%, insect bites (wasps) 10 cases - 16.6%,unidentified etiology in 5 cases - 8.3%, chemical factor 3 cases - 5%. Of the total group of patients 54 patients - 90% were in serious condition or medium severity of which 2 patients - 3.7 refused hospitalization, and 6 patients - 10% in extremely serious condition were admitted to the ICU ward. The main clinical manifestations was - hTA- 61.6%, dyspnea 68.3%, edema - 23.3%, redness - 18.3%, hives prevailed in 53.3% cases. In the prehospital phase all patients received emergency medical aid according to the protocol and the drug of choice was the administration of adrenaline.

**Conclusions**

Anaphylaxis is an acute, potentially fatal systemic reaction with varied clinical presentations. The most frequently affected are female. The most common causes of anaphylaxis are foods and medication. Prompt recognition and treatment of anaphylaxis are imperative; however, both patients and healthcare professionals often fail to recognize and diagnose anaphylaxis in its early stages.

## P324 Surviving medical emergencies: a pioneering project in near-peer simulation

### R Tabbara, JZ Whitgift

#### Queen Elizabeth Kings Lynn Hospital, Norfolk, United Kingdom

**Introduction**

Simulation as a teaching tool has swiftly become popular amongst instructors & trainees in the education of medical students/junior doctors. In the UK, sessions are delivered by more senior doctors. Due to other clinical demands, this can limit availability of simulation training for medical students. Whilst junior doctors are often involved in simulation, this is generally under the supervision of senior doctors. Our work demonstrates that junior doctors can design & deliver high-quality simulation experiences for medical students without the need for direct input from senior clinicians

**Methods**

A 6 week simulation series was provided to final year medical students at the University of Cambridge & University of East Anglia. Each session was designed around a topic with two scenarios & tutorial provided. All sessions were planned & delivered by doctors with 1-3 years of post-graduate experience. Pre/post-session self-assessment forms with Likert scale scoring were completed focusing on four domains. Parametric distribution was confirmed with Anderson-Darling tests, mean of the pooled scores compared with Unpaired Student T-tests

**Results**

**Fig. 44 (abstract P324).**
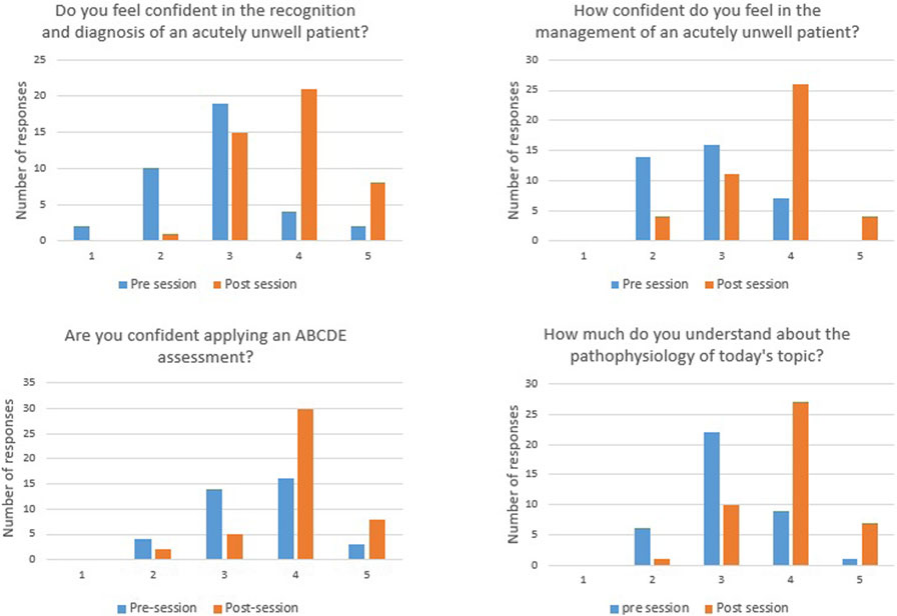
Student Self-Assessment Questionnaire Results

Figure 44 demonstrates the distribution of self-assessment scores. These indicate a general improvement amongst all domains. When mean scores were compared (Table 33) we found a statistically significant improvement in the diagnosis, management and pathophysiology areas. Cambridge subgroup analysis showed an improvement in assessment of unwell patients that was not observed in the pooled mean

**Conclusions**

Near-peer delivered simulation can train students in a comfortable environment that translates to statistically significant improvements in self-assessed performance. We encourage centres to explore near-peer delivered sessions in the expansion of simulation training.

**Table 33 (abstract P324).** See text for description

Category	Mean pre-course score (95% CI)	Mean post-course score (95% CI)	P-value
Diagnosis of unwell patients	2.8 (2.5-3.1)	3.8 (3.6-4.0)	<0.0001
Assessment	3.5 (3.2-3.7)	3.8 (3.6-4.0)	0.075
Assessment (UoC subgp)	3.2 (2.9-3.5)	3.8 (3.5-4.1)	<0.01
Management	2.8 (2.6-3.0)	3.7 (3.4-3.9)	<0.0001
Pathophysiological process	3.1 (2.9-3.4)	3.9 (3.7-4.1)	<0.0001

Legend: Mean self-assessment scores

## P325 The issue of emergency medical transport in an aging society at Tokyo

### A Ishimaru, A Yaguchi, N Akiduki, M Namiki, M Takeda

#### Tokyo Women´s Medical University, Tokyo, Japan

**Introduction**

Recently, Japan is one of the most aged countries, which peoples over the age of 65 consist of 26% of total population, in the world. Tokyo, the capital of Japan, in which society is aging in a similar manner. Furthermore, the number of emergency medical transport patients is growing every year at Tokyo. It seems those growths is influenced by increasing elder patients. The purpose of this study is to clarify the issue of emergency medical transport in an aging society.

**Methods**

Data sources using the reports of Fire and Disaster Management Agency of the Ministry of Internal Affairs and Communications, and Tokyo Fire Department were conducted. The number of emergency medical transport patients, the type of emergency cases and the number of cardiopulmonary arrest in patients with 65 years old and over called ambulance at Tokyo were compared between in 2005 and in 2015. The growth rates of those numbers were also calculated.

**Results**

Table 34 shows the number of total population at Tokyo, of total emergency medical transport, of illness patients, of injured patient, and of cardiopulmonary patients in emergency medical transport in 2015 and in 2005. The growth rate of total population, total emergency medical transport, illness patients, injured patient and cardiopulmonary patients in 65 years old and over were 33%, 36.7%, 33.2%, 58.7% and 39.5% respectively. However, the numbers of ambulance were 335 in 2015 and 305 in 2005. And the growth rate of the number of ambulance was 9.8%.

**Conclusions**

Due to be well on the way to an aging society at Tokyo, the number of emergency medical transport in 65 years old and over increased, especially injured patients significantly increased. On the other hand, the number of ambulances were shortage. The more useful and practical emergency medical transport system is required in the Metropolitan area.

**Table 34 (abstract P325).** See text for description

	Total population at Tokyo	Total emergency medical transport patient	Ill patient	Injured patient	Cardiopulmonary arrest patient
2015 (total)	13,510,000	673,145	441,043	118,021	12,365
2015 (65 y.o. and over)	3,060,000	335,564	230,972	66,83	9,3
2005 (total)	11,770,000	643,849	397,006	96,75	9,794
2005 (65 y.o. and over)	2,300,000	245,441	173,432	42,112	6,666

Legend: The number of emergency medical transport between 2015 and 2005 at Tokyo

## P326 Alcohol-related intensive care unit admissions predict subsequent alcohol-related or violent deaths: a 12-year follow-up study

### JN Tamminen^1^, M Reinikainen^2^, A Uusaro^1^

#### ^1^Kuopio University Hospital, Kuopio, Finland; ^2^North Karelia Central Hospital, Joensuu, Finland

**Introduction**

Intensive Care Unit (ICU) admission is often related to alcohol [1,2]. We aimed to investigate the long term mortality and causes of death of patients with alcohol-related ICU admission.

**Methods**

This is a follow-up of a previous cohort study [2]. The study cohort consists of emergency admissions to a University hospital ICU from March 2002 to April 2003. For each patient, the admitting physician assessed whether there was a relationship between alcohol use and the need for intensive care. We identified the patients using the Finnish social security number, which is a unique identifier of people in Finland. Only the first admission during the study period was included. We acquired the Death Certificates for the patients from the Statistics Finland's Death Certificate Registry. P-value were calculated using Whitney-Mann U-test and Fisher’s exact test as appropriate.

**Results**

There were 806 unique patients admitted to the ICU during the study period. Two patients were admitted to the ICU with temporary social security number and they were excluded from the study. There were two missing death certificates (0.5%), both in the non-alcohol related group. There were 202 alcohol-related admissions (25.1%). Patients in the alcohol-related admission group were younger (median 47.8 yrs vs. 59.3 yrs, p < 0.001) and there were more men (78.7% vs. 58.9%, p < 0.001). SAPS II scores (mean 36 vs. 36, p = 0.83) and ICU mortality was similar in both groups (9.4% vs. 10.3%, p = 0.79). During the 12 year follow-up period after the ICU discharge more patients died in the alcohol-related group (59.6% vs. 50.4%, p = 0.03). The cause of death was alcohol-related, accidental or violent in 77 patients (70.6%) in the alcohol-related group vs. in 30 patients (11.1%) in the non alcohol-related group (p < 0.001). Mortality was at its highest during the first year after ICU discharge.

**Conclusions**

An alcohol-related ICU admission means a high risk for alcohol-related, violent or accidental death in subsequent years.

**References**

[1] McKenny M, O’Beirne S, Fagan C, O’Connell M. Alcohol-related admissions to an intensive care unit in Dublin. Irish Journal of Medical Science 2010;179:405–8.

[2] Uusaro A, Parviainen I, Tenhunen J, Ruokonen E. The proportion of intensive care unit admissions related to alcohol use: a prospective cohort study. Acta Anaesthesiologica Scandinavica 2005;49:1236–40.

## P327 Estimating the impact of injecting drug use for patients in critical care

### CG Taylor, ED Mills, AD Mackay

#### NHS Greater Glasgow & Clyde, Glasgow, United Kingdom

**Introduction**

Glasgow has some of the highest rates of problem drug use in Scotland with a prevalence rate of 3.2% in 2012 (this includes both injecting drug use and illicit use of opiates and benzodiazepines). People who inject drugs (PWID) are at significant risk of ill health and infection. In 2015 an outbreak of HIV in the city resulted in 40 new cases (four times the average yearly incidence). In the United Kingdom, there is an estimated 40% prevalence of Hepatitis C and a 1% prevalence of HIV amongst PWID.

We aimed to investigate the prevalence of blood-borne viruses in PWID admitted to intensive care and to compare diagnoses, measures of morbidity and mortality against patients thought not to have a drug use history.

**Methods**

We undertook a retrospective review of the records of 1109 admissions to intensive care at the Queen Elizabeth University Hospital Glasgow, between August 2015 and September 2016. Patients were identified as PWID or without an illicit drug use history (this includes both problem drug use described above, and recreational illegal drugs) by admission documentation. All measures were retrieved from the unit’s electronic audit system. Statistics were performed using R version 3.3.2

**Results**

Of the 1109 patients, there were 380 records of patient’s drug history. 37 (9.7%) were identified as PWID and 232 (61%) were thought not to have a history of illicit drug use.

The average age in PWID was 43 years (versus 63 in the comparison group, p < 0.001), median APACHE II score in PWID was 16 (19 in the comparison group, p = 0.02). The average mortality and predicted mortality in PWID was 8.6% and 16.1% (versus 19% and 26.6%, p = 0.02 and 0.003). There was no significant difference in gender split or length of ICU stay.

There were 0 cases of Hepatitis B and 1 case of HIV in our cohort. There was a 59.5% prevalence of Hepatitis C amongst PWID (0.9% in the comparison group, p < 0.001). The most common APACHE II diagnoses amongst PWID were: drug overdose, and aspiration/poisoning/toxic. The most common diagnoses in patients without an illicit drug use history were: respiratory infection, and seizure disorder.

**Conclusions**

The most frequent reasons for admission amongst people who inject drugs can mostly be attributed to their drug use. PWID tend to be younger than other patients, with a lower APACHE score, predicted mortality, and actual mortality. The prevalence of Hepatitis C in our patients who inject drugs is approximately 1.5 times higher than the estimates of the national average for PWID, and 60 times greater than in the non-injecting drug user population.

## P328 Readmission to intensive care unit: incidence, risk factors, resource use and outcomes: a retrospective cohort study

### C Ponzoni, R Rabello, A Serpa, M Assunção, A Pardini, G Shettino, T Corrêa

#### Hospital Israelita Albert Einstein, São Paulo, Brazil

**Introduction**

Readmission to intensive care unit (ICU) is associated with poor clinical outcomes, increased length of ICU and hospital stay and higher costs [1]. Knowledge on epidemiology of unplanned ICU readmissions, risk factors and attributable outcomes are restricted to developed countries. Our objectives were to determine incidence of unplanned ICU readmissions, identify predictors of ICU readmissions and of hospital mortality and compare resources use and outcomes between readmitted and nonreadmitted patients.

**Methods**

This retrospective single center cohort study was conducted in a forty bed, open clinical-surgical, high-density intensivist ICU of a private, tertiary care, hospital in São Paulo, Brazil. The Local Ethics Committee at Hospital Israelita Albert Einstein approved the study protocol and the need for informed consent was waived. All consecutive adult (> = 18 years) patients admitted to the ICU between June 1, 2013 and July 1, 2015 were enrolled in this study. Comparisons were made between patients readmitted and nonreadmitted to ICU, between readmissions < =72 hours and >72 hours after ICU discharge and between index ICU admission and first ICU readmission. Logistic regression analyses were performed to identify predictors of ICU readmissions and hospital mortality.

**Results**

Out of 5,779 patients admitted to ICU, 576 (10%) were readmitted to ICU during the same hospitalization. Compared to nonreadmitted patients, patients readmitted to ICU were more often male, showed a higher SAPS 3 score at index ICU admission and were more frequently admitted due to medical reasons. SAPS 3 score, admission source, vasopressors and renal replacement therapy need during index ICU stay, discharge to step-down unit and length of ICU stay were independent predictors of ICU readmission. After adjusting for severity of illness, ICU readmission was associated with increased risk of in-hospital death (OR, 4.07; 95%CI, 3.20 to 5.17; p < 0.001) as well as admission source, use of vasopressors, mechanical ventilation or renal replacement therapy, length of ICU stay and nighttime ICU discharge.

**Conclusions**

Unplanned readmissions to ICU were frequent and strongly related to poor outcomes. The degree at which ICU readmissions are preventable as well as the main causes of preventable ICU readmissions need to be further determined.

**References**

1. Wong EG et al. Association of severity of illness and intensive care unit readmission: A systematic review. Heart Lung 45:3–9, 2016.

## P329 Mortality predictor in HIV critically ill patients: “retro-VIH” score

### PV Vidal-Cortés^1^, L Álvarez-Rocha^2^, P Fernández-Ugidos^1^, A Virgós-Pedreira^2^, MA Pérez-Veloso^1^, IM Suárez-Paul^2^, L Del Río-Carbajo^1^, S Pita Fernández^2^, A Castro-Iglesias^2^

#### ^1^CHU Ourense, Ourense, Spain; ^2^CHU A Coruña, A Coruña, Spain

**Introduction**

Our objective is to predict hospital mortality en HIV critically ill patients.

**Methods**

Retrospective study. HIV patients admitted to CHU A Coruña and CHU Ourense ICUs from 2000 to 2014. We analyzed demographic variables, comorbidities, nutritional and inmunological status, reason for admission and need for organ support during the first 72 hours of admission and their impact on hospital mortality. We used logistic regression to calculate odds ratio. Statistically significant variables related to hospital mortality were assigned a coefficient. Retro-VIH score was calculated as the sum of these coefficients. We calculated the area under the ROC curve of Retro-VIH score to predict hospital mortality and we compared it with APACHE II, SOFA score at 24 and 72 hours and DeltaSOFA0-72 h(SOFA72h-SOFA0h).

**Results**

297 patients, 43,2 ± 9,6 years, 72,4% men. Hospital mortality (HM): 34.3%. Variables related with HM: male (OR = 1.91; p = 0.03, IC95% 1.07-3.39), admission from ward (OR = 4.18; p < 0.001, IC95% 2.50-6.97), no antirretroviral therapy (ART) on admission (OR = 1.86; p = 0.01, IC95% 1.14-3.02), cirrhosis (OR = 2.12; p = 0.03, IC95% 1.06-4.22), AIDS (OR = 1.80; p = 0.03, IC95% 1.04-3.12), caquexia (OR = 2.56, p = 0.001, IC95% 1.50-4.36), albuminemia < 2.2 g/dL (OR = 2.63; p < 0.001, IC95% 1.61-4.31), infection on admission (OR = 2.00, p = 0.005, IC95% 1.23-3.27), mechanical ventilation (MV) in the first 72 h (OR = 5.01, p < 0.001, IC95% 2.80-8.97), vasopressors in the first 72 h (OR = 2.51, p < 0.001, IC95% 1.52-4.13), renal replacement (RR) in the first 72 h (OR = 5.70, p < 0.001, IC95% 2.41-13.47), DeltaSOFA0-72 h > =0 (OR = 3.44, p < 0.001, IC95% 1.90-6.24). Assigned coefficient: male:2, admission from ward:4, no ART on admission:2, cirrhosis:2, AIDS:2, caquexia or albuminemia < 2.2 g/dL:2, infection on admission:2, MV in the first 72 h:5, vasopressors in the first 72 h:2, RR in the first 72 h:5, DeltaSOFA0-72 h > =0:3. RetroVIH score (0-31 points) and HM:0-7:0%, 8-11:20%, 12-15:36.6%, 16-19:49%, 20-23:67.5%, 24-27:90.9%, 28-31:100%. RetroVIH score is related with HM: B = 1.27 (p < 0.001, IC95% 1.20-1.36). Area under ROC curve RetroVIH score to predict HM:0.82, APACHE II:0.79, SOFA0h:0.74, SOFA72h:0.72, DeltaSOFA0-24 h:0.66.

**Conclusions**

We developed a prognostic score combining demographic variables, comorbidities, nutritional and inmunological status, reason for admission, and need for organ support on first 72 h.

Mortality increases significantly with each point of the RetroVIH score, and it predicts hospital mortality more accurately than APACHE and SOFA in HIV critically ill patients.

## P330 Correlation of ICU scoring systems with mortality outcomes after lung transplantation: a 5 year, single center experience

### A Butt, AA Alghabban, SK Khurshid, ZA Ali, IN Nizami, NS Salahuddin

#### King Faisal Specialist Hospital & Research Center, Riyadh, Saudi Arabia

**Introduction**

There is very little data on variables within the immediate post-transplant period that may directly influence outcomes after lung transplant. This study attempts to identify modifiable risk factors during the ICU admission.

**Methods**

We carried out a cohort study of all single or bilateral lung transplant patients admitted to the ICU, period of study 2010 - 2015.

**Results**

Total number of transplants was 80, mean age 33.3 years ±15.1, 44 (55%) were male. Indications for transplantation were COPD in 1 patient, cystic fibrosis and bronchiectasis in 38 patients (48%) and interstitial lung diseases in 41 patients (51%). Mean APACHE II score was 15.6 ± 5.6, SAPS II score 26 ± 11.4, mean Tidal volume 325.9 ml ±66.9, mean FiO2 61.6% ±18.2 (range 30,100%), mean PEEP 6.6 ± 1.9 (range 5,18), mean PIP 25 ± 3.8 (range 15,37), mean driving pressure was 16.4 ± 6.1, mean PaO2 105.2 ± 38.8, median packed RBC units transfused 5.6 (IQR3.3), non-RBC units 6.9 ± 3.7. Fluid balance in the first shift after admission to the ICU was +330 ml ±997 (range -930,5362) and in the first 48 hours was +563 ml ± 1691 (range -3549,8087). 1 month mortality rate was 16.3% (13 died). 1, 3 and 5 year survival rates were 81, 64 and 57% respectively. Acute rejection developed in 26 patients (32.5%), mean time to rejection was 187.9 ± 229 (range 10,640). Variables associated with increased 1 month mortality: age: p value 0.002, APACHE II score: p value 0.043, tidal volume: p value 0.01.Significant differences between survivors and non-survivors were observed in numbers of blood product units transfused (p =0.001) and tidal volume (p <0.001). PaO2 was significantly lower in patients who ultimately developed transplant rejection, p = 0.016. On univariate regression analysis, variables significantly associated with 1 month-mortality were; non-RBC units (OR 1.6, 95% CI 1.063, 2.5, p = 0.025), PRBC units OR 1.2, 95% CI 1.060, 1.393, p = 0.005), PaO2 (OR 0.96, 95% CI 0.93, 0.996, p = 0.029), age (OR 1.06, 95% CI 1.02, 1.1, p = 0.003), Transplant type (OR 0.11, 95% CI 0.029, 0.454, p = 0.002), APACHE II score (OR 0.902, 95% CI 0.805, 1.01, p = 0.073), Tidal volume (OR 0.989, 95% CI 0.978, 1.0, p = 0.060). On multivariate regression, age, p = 0.014 and number of PRBC units, p = 0.007, remained significantly associated with 1 month mortality.

**Conclusions**

We found that in the immediate post-transplant period, decreased PaO2, increased APACHE II scores, lower tidal volumes and increased blood product transfusion were all associated with increased short-term mortality.

## P331 Prediction of emergency department triage category and clinical presentation on disposition and clinical outcome of h1n1 patients

### M Alshahrani^1^, AW Alsubaie^1^, AS Alshamsy^1^, BA Alkhiliwi^1^, HK Alshammari^1^, MB Alshammari^1^, NK Telmesani^1^, RB Alshammari^1^, LP Asonto^2^

#### ^1^Dammam University, Khobar, Saudi Arabia; ^2^King Fahad Hospital of the University, Khobar, Saudi Arabia

**Introduction**

In 2009, the first case of H1N1 influenza infection was reported. Seasonal outbreaks in different parts of the world still occur until date wherein most patients present to the emergency departments with flu-like symptoms; although some develop a more severe respiratory symptoms that needed admission and further treatments, no available data has been made as to how we can predict admission and clinical outcome upon initial presentation.

Therefore, the aim of this study is to identify predicting factors for the need of admission and clinical outcome of H1N1 patients in emergency department.

**Methods**

A retrospective chart review of all patients who presented to the emergency department at King Fahad Hospital of the University, Dammam, Saudi Arabia with positive H1N1 flu polymerase chain reaction (PCR) for the period between November to December 2015 was made. We excluded patients with no documented vital signs and/ or with no documented triage category in the ED visit. Regression analysis was conducted to look for factors that predict the outcome, which are the need for hospital admission or discharge from the emergency department, length of hospital stay or death of patient as a result of this illness.

**Results**

333 positive H1N1 patients were identified; of those, 80 patients (24%) were admitted to the hospital through the emergency department and 4 patients (1.2%) died during their hospitalization. Triage category 3 was the most prevalent with 64.8%, 18% of them got admitted compared to 46.8%. Multivariate regression analysis showed that among all vital signs, Tachypnea was found to be a risk for getting admitted in hospital OR = 1.1; 95% CI: 1.02 – 1.13) (p < 0.01). The association between triage category and hospital stay was significant (χ =6.068,p = 0.037) where the proportion of triage category 3 was more in the longer hospital stay group compared to category 4. Another significant finding is that patients had dyspnea; they are 4.5 times more likely to have a longer hospital stay (OR = 4.5; 95% CI: 1.2 -17.1) (p = 0.025).

**Conclusions**

In H1N1 infected patients, the initial triage category of 3 or less, and increased respiratory rate are found to predict the need for hospital admission wherein patients with dyspnea symptom have higher chance of longer hospital stay.

## P332 Is illness severity variance at admission associated with icu standardized mortality ratio?

### FG Zampieri^1^, LP Damiani^1^, F Bozza^2^, JI Salluh^2^, AB Cavalcanti^1^

#### ^1^HCor-Hospital of the Heart, São Paulo, Brazil; ^2^D’Or Institute for Research and Education, Rio de Janeiro, Brazil

**Introduction**

ICUs admit patients with variable degrees of severity. It is unknown whether this very variability (illness severity variance) is different domain apart from mean illness severity. We hypothesized that 1) Variance of illness severity would be independently associated with unit's standardized mortality (SMR) and 2) Units with a higher illness severity variance would have higher SMR.

**Methods**

This is a secondary analysis of the CHECKLIST Trial. Unit SMR was calculated based on SAPS3 predicted mortality over observed mortality. A linear regression was performed to assess whether SAPS3 standard deviation (SD) would be associated with SMR while adjusting for the ICUs mean SAPS3 and their interaction.

**Results**

**Fig. 45 (abstract P332).**
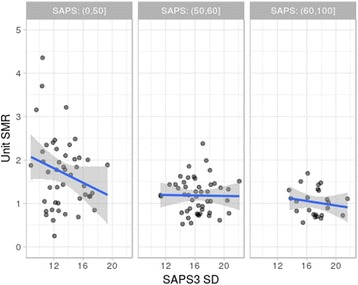
Association between SAPS3 SD and SMR stratified by unit's SAPS3 Mean

13,685 patients from 118 Brazilian ICUs were included in this analysis (mean = 115 patients/unit). Mean SAPS3 was 52.5 (SD = 17.5) and mean unit’s SMR was 1.35 (SD = 0.66). Higher SAPS3 SD was associated with lower SMR on univariate analysis, but only when mean SAPS3 was low (Fig. 45). Both SAPS3 variance and mean unit admission SAPS3 were negatively associated with unit’s SMR (Table 35).

**Conclusions**

Variance of illness severity is a different domain apart from units mean SAPS3 that may be relevant to understand the full picture of illness severity in a ICU. Contrary to our initial hypothesis, variance of illness severity at admission was associated with lower ICU SMR; this was only evident when the mean unit SAPS3 was low. Variance of illness severity should be considered when interpreting unit’s SMR.

**Table 35 (abstract P332).** See text for description

Variable	Estimate	SE	p
SAPS3 Mean	-0.127	0.032	<0.001
SAPS3 SD	-0.306	0.112	0.007
Interaction	0.005	0.002	0.007

Legend: Linear regression results

## P333 Health related quality of life trajectories of patients after acute illness.

### A El Khattate, M Bizrane, N Madani, J Belayachi, R Abouqal

#### Ibn Sina University Hospital, Rabat, Morocco

**Introduction**

This study aimed to identify and describe a set of longitudinal HRQoL trajectories then determine factors associated with trajectory class membership of patients after acute illness.

**Methods**

This was a prospective cohort study conducted in an acute medical unit (AMU) of Ibn Sina University Hospital, Rabat, Morocco, between June and September 2014. Patients aged more than 17 years admitted to AMU were included. Demographic, medical history, clinical and paraclinical characteristics were recorded at admission. EQ5D index, EQ-VAS and survival status of patients were collected four times: at admission, 3, 6 and 18 months of follow-up. Latent class growth analysis (LCGA) was applied to identify classes of HRQoL trajectories, while association between baseline covariate and class membership were identified using polynominals logistic regression. Statistical analysis was carried out in STATA version 14.

**Results**

Two hundred fifty one patients were included. The mean age was 55.6 ± 18.9 years and women were 54.6%. In-hospital and 540 days post-discharge follow-up mortality were respectively 11.6% and 34.3%. For 229 patients included in LCGA, three trajectory classes where identified for EQ5D Index; stably low (16.2%), stably moderate (30.6%), and high initially increasing (53.2%). The three trajectory classes of EQ-VAS were low increasing, moderate initially increasing and high initially increasing with, respectively, 29.7%, 48.5% and 21.8% of the patients. Concerning EQ5D index, comparing to high initially increasing trajectory, factors associated to; a)stably low trajectory membership were:age > =70 years (OR:9.3; CI1.7to49; p = 0.008), intensive care unit transit (OR:5.1;CI0.01to10;p = 0.01) and low hemoglobinemia (OR:0.8;CI0.05to1.1;p = 0.05); b) stably moderate trajectory membership were: comorbidity (OR:2.8; CI0.01to 6.4; p = 0.01) and low hemoglobinemia (OR:0.8;CI0.001to0.9;p = 0.001). Concerning EQ-VAS, comparing to high initially increasing trajectory, factors associated to: a) low increasing trajectory membership were: female gender (OR:5; CI 1.7to10;p = 0.006), Km hospital-residence (OR: 0.9; CI 0.9 to 0.9; p = 0.01) and comorbidity (OR: 9.6; CI 3.1 to 29; p < 0.001), b) moderate initially increasing trajectory membership was comorbidity (OR: 9.5; CI 3.7 to 24; p < 0.001).

**Conclusions**

Three HRQoL trajectories were identified. Aged patients with low hemoglobinemia and who transited through ICU had the worst EQ5D index. Females, with comorbidities, living far from hospital perceived an amelioration of their, previously low, EQ-VAS.

## P334 Assessment of cognitive function in critically ill patients prior to ICU admission.

### D Ramnarain, B Gouw-Donders

#### Elisabeth Tweesteden Hospital Tilburg, Tilburg, Netherlands

**Introduction**

Cognitive dysfunction after severe illness has been identified in multiple patient groups leading to disability and poor quality of life. In ICU survivors cognitive dysfunction is part of the so called post-ICU syndrome. In order to identify patients at risk for developing cognitive dysfunction due to critical illness or ICU treatment one has to discriminate between patients having cognitive decline prior to ICU admission with those developing new cognitive dysfunction or worsening of cognitive function after ICU treatment. We therefore assessed cognitive function in ICU patients on admission using the Informant Questionnaire on Cognitive Decline in the Elderly (IQCODE). (1)

**Methods**

On admission patients relative were asked to fill in the IQCODE to rate the subject’s improvement or decline in 16 aspects of memory and intelligence over the last ten year period. (2) This questionnaire covers changes in learning, recall abilities, recognition, comprehension, and other aspects of intelligence. Each item is rated on a one to five scale. An overall change score is calculated by averaging the scores on each item. One represents considerable improvement, 3 indicates no change and five represents considerable deterioration.

**Results**

We analyzed the IQCODE of 213 consecutive patients admitted to our ICU. Population consisted of 118 men and 98 women. Mean age was 63 ± 16 years. A large group of patients, 34.3% (n = 73) showed decline in cognitive functioning prior to ICU admission. After analysis cognitive dysfunction could be divided in three groups; 1. 64.3. % (n = 47) indicated slight decline, 2. 27.4% (n = 20) indicated moderate decline, and 3. 8.2% (n = 6) indicated severe decline of cognitive functioning prior to ICU admission.

**Conclusions**

Incidence of pre-existing cognitive dysfunction in critically ill patients was 34.3%. A great percentage of critically ill patients show cognitive decline prior to ICU admission. Interpretation of data concerning cognitive dysfunction related to critical illness or ICU treatment is therefore difficult. Data on cognitive dysfunction in ICU survivors should be corrected for patients pre-ICU cognitive functioning.

**References**

1. Harrison JK, Stott DJ, McShane R, et al: Informant questionnaire on cognitive decline in the elderly (IQCODE) for the early diagnosis of dementia across a variety of healthcare settings. Cochrane Database Syst Rev 2016;11:CD011333

2. Jorm AF: A short form of the informant questionnaire on cognitive decline in the elderly (IQCODE): Development and cross-validation. Psychol Med 1994;24:145–153

## P335 Standardizing outcomes for adult cardiac surgery trials: a consensus study

### C Benstoem^1^, A Moza^1^, P Meybohm^2^, C Stoppe^1^, R Autschbach^1^, D Devane^3^, A Goetzenich^1^

#### ^1^University Hospital RWTH Aachen, Aachen, Germany; ^2^University Hospital Frankfurt, Frankfurt, Germany; ^3^National University of Ireland Galway, Galway, Ireland

**Introduction**

When planning clinical trials, it is a key element to choose appropriate outcomes to assure the comparability of effects of interventions in ways that minimize bias; however, there is a growing body of evidence indicating that inadequate attention has been paid to the outcomes measured in cardiac trials. Choice and definitions of outcomes vary considerably. These problems are well recognized by systematic reviewers as inconsistencies and heterogeneity in outcome reporting significantly limits the ability of research synthesis. Core outcome sets (COS, a set of outcomes that should be measured and reported, as a minimum, in all clinical trials for a specific clinical field) help minimize this problem. In light of the above, we developed a COS for cardiac surgery effectiveness trials.

**Methods**

Potential core outcomes were identified a priori by analyzing data on 371 RCTs of 58,253 patients. We used robust methods as recommended by the COMET Initiative to develop a COS for adult cardiac surgery trials. We obtained ethical approval and published a study protocol. We reached consensus on core outcomes in an international three-round eDelphi exercise (participants, n = 86) from 23 different countries involving adult cardiac patients, cardiac surgeons, anesthesiologists, nursing staff and researchers. Participants indicated if an outcome was important enough to be included in the COS. Outcomes for which at least 60% of the participants chose the response option “no” and less than 20% chose the response option “yes” were excluded from the list of potential outcomes. We followed the COS-STAR reporting guideline for COS studies.

**Results**

The panel reached consensus after the third round of the eDelphi on four core outcomes: 1) Measure of mortality, 2) Measure of quality of life, 3) Measure of hospitalization and 4) Measure of cerebrovascular complication to be included in adult cardiac surgery trials.

**Conclusions**

This study used robust research methodology to develop a core outcome set for use in cardiac surgery trials. This does not imply that primary outcomes should always and exclusively be those of the COS. However, to assure the comparability of results across trials, the outcomes included in this COS should be measured and reported in all trials evaluating the effectiveness of treatments in the setting of cardiac surgery. As a next step, appropriate outcome measurement instruments have to be selected.

## P336 Machine learning for prediction of mortality and prolonged length of stay at intensive care unit in a Brazilian cohort of critically ill patients

### LU Taniguchi ^1^, L Araujo^2^, G Salgado^2^, JM Vieira Jr^1^, J Viana^2^, N Ziviani^2^

#### ^1^Research and Education Institute, Sao Paulo, Brazil; ^2^Kunumi, Belo Horizonte, Brazil

**Introduction**

Machine learning is a branch of computational methods, which allows an algorithm to program itself by learning from a large database. The objectives of this study was: (1) to compare hospital mortality prediction between this technique and usual prognostic model (SAPS 3) in a Brazilian cohort of critically ill patients, and (2) to evaluate the prediction of prolonged length of stay at the intensive care unit.

**Methods**

This is a retrospective cohort study conducted at a private tertiary hospital (Hospital Sirio-Libanes) at São Paulo, Brazil. We extracted relevant information from the adult intensive care unit database (sistemaEpimed™). Gradient boosting using 5-fold cross-validation was applied for creation of machine learning model (MLMmortality). A comparison was performed between SAPS 3 score model and MLMmortality for predicting hospital mortality. Models discriminations were compared using area under the ROC curves. Another machine learning model was developed for prediction of prolonged length of stay, defined as more than 10 days at the intensive care unit, using the same procedure.

**Results**

Between June 2013 to June 2016, we studied 5305 patients (mean age, 65.9 years; 53% male sex; hospital mortality of 12.5%). Area under ROC curve for hospital mortality for SAPS 3 was 0.79 (95% CI 0.76 – 0.82)and for MLMmortality was 0.87 (95% CI0.86 – 0.88]; p < 0.01 for comparisons between SAPS 3 and MLMmortality). Area under ROC curve for prolonged length of stay was 0.79 (95% CI 0.77 – 0.81).

**Conclusions**

Machine learning is a promising tool for prediction in critical care.

## P337 Continuous automated modified sofa score is a good early predictor of outcome in patients with hemodynamic instability

### I Pessach^1^, A Lipsky ^2^, A Nimrod^3^, M O´Connor^4^, I Matot^3^, E Segal^5^

#### ^1^Sheba Medical Center, Tel-Hashomer, Israel; ^2^Intensix, Predictive Critical Care LTD, Netanya, Israel; ^3^Tel Aviv Medical Center, Tel-Aviv, Israel; ^4^University of Chicago Medicine, Chicago, IL, United States; ^5^Assuta Medical Centers, Tel-Aviv, Israel

**Introduction**

SOFA score is a useful tool for predicting outcome of critically ill patients and is used to stratify patients and compare outcomes. The SOFA score is typically measured once daily, and utilizes the worst parameters within a 24 hours window for the calculation of the score. Thus, its possible utility for real time on-going management has not been fully explored. Changes in SOFA score over 72 hours have been shown to predict survival in emergency room patients with septic shock.(1) We hypothesized that a computerized system which analyzes the SOFA parameters continuously will be able to differentiate survivors from non- survivors early in the course of their illness.

**Methods**

The ability of a computerized system to calculate a modified SOFA score continuously, and to predict outcome based on the temporal changes in the score was studied. The analysis and model development was performed on a database of 629 septic patients with severe hemodynamic instability events admitted to a general ICU in a tertiary medical center between 2007-2014, and validated in 92 patients with such events admitted during 2015.

**Results**

**Fig. 46 (abstract P337).**
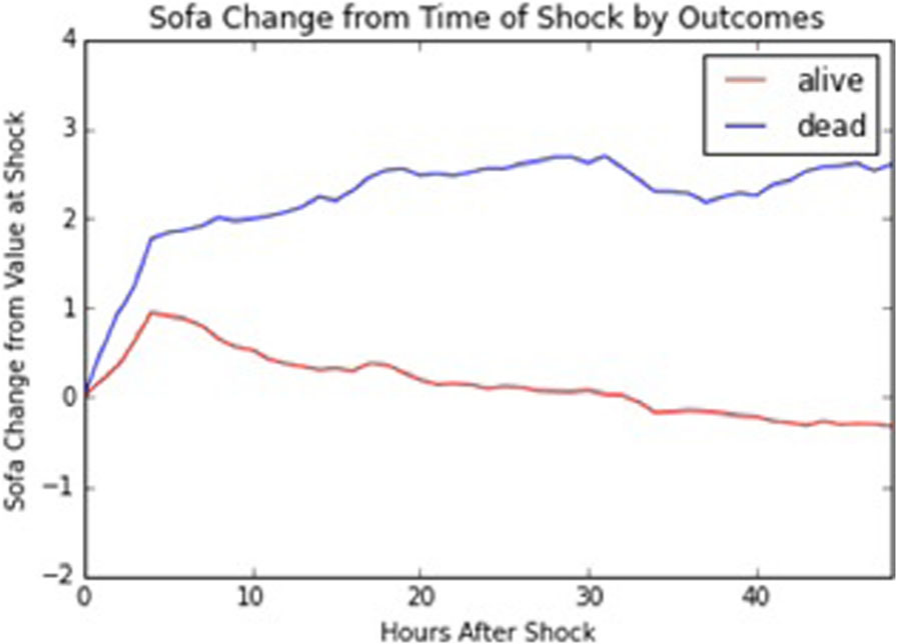
Continuous SOFA score in survivors versus non-survivors following a shock event

Continuous analysis of modified SOFA score could segregate survivors and non-survivors following a severe hemodynamic instability event. There was a clear pattern of behavior of the continuous SOFA in survivors versus non-survivors (Fig. 46). Following an episode of severe hemodynamic instability, patients whose continuous SOFA score deceased after an initial increase had a significantly lower mortality risk than those that maintained a steady increase in the continuous SOFA score. The difference in behavior of the score between survivors and non-survivors was evident and statistically significant as early as 4 hours following the initial event.

**Conclusions**

We propose that continuous analysis of the modified SOFA score by a smart automated system can detect and predict significant clinical outcomes early in the course of the event. The potential impact on clinical practice should be evaluated in a prospective manner.

**References**

1. Jones AE, Trzeciak S, Kline JA. Crit Care Med. 2009;37(5):1649–54.

## P338 Assessment and predictors of physical and mental functioning in adult survivors of critical illness

### A Kluzik^1^, A Gradys^1^, P Smuszkiewicz^1^, I Trojanowska^1^, M Cybulski^2^

#### ^1^Heliodor Swiecicki Clinical Hospital at the Karol Marcinkowski Medical University in Poznan, Poznan, Poland; ^2^University of Medical Science, Poznan, Poland

**Introduction**

Improvement of intensive care medicine leads to the increased survival rate among critically ill patients. On the other hand, it results in long-term consequences such as decreased muscle strength, mental disability and quality of life of survivors. The purpose of our study was to objectively assess muscle strength, daily activity and mental functioning in survivors of critical illness in general ICU and to find prognostic factors contributing to loss of functional capacity.

**Methods**

This prospective study was conducted on the general ICU between March 1, 2014 and June 30, 2015 and included patients subjected to mechanical ventilation for at least 3 days. Patients were assessed at admission, discharge and one year post-ICU discharge. The SOFA and APACHE II scores, demographic data and the cause of admission were evaluated. Muscle strength was measured using hand-held dynamometry and MRC (Medical Research Council) score. Mental state was assessed by means of MMSE (Mini-Mental State Examination) scale and daily functioning using FIM (Functional Independence Measure) score. Variables were evaluated using the Mann-Whitney, Kruskal-Wallis and Spearman’s tests. Study was approved by institutional Bioethics Committee.

**Results**

Totally 30 patients (age 24-78; 56.1 ± 16.4) were enrolled and among them 7 died during first year after discharge, and another 9 patients were lost to follow-up, including 4 who gave information by phone only about daily functioning (FIM). The study proved correlation between SOFA scale at admission (11.6 ± 4 pts) and muscle strength assessed by hand-held dynamometry at the time of discharge and between SOFA and mental functioning (MMSE at admission 22.5 ± 7) at the time of discharge of the patient, however were lost in the first year post ICU. It wasn’t any correlation with APACHE II score (at admission 26.4 ± 7 pts). Despite the severity of illness and LOS (Length of Stay) other singular risk factors (such as kidney insufficiency, septic shock, neoplasm, obesity, hemorrhagic shock, COPD, reintubation and readmission to ICU) did not influence the outcomes in handgrip strength, MRC score, MMSE and FIM scales.

**Conclusions**

It has been shown a direct relationship between severity of illness determined by SOFA score and muscle strength measured with a hand-held dynamometry as well as the decreased outcomes in MMSE scale during discharge from ICU. Decreased mental functioning, muscles strength and daily activity were not observed among ICU survivors after one year follow-up.

## P339

### Withdrawn

## P340 Medical versus surgical icu obese patient outcome: a propensity-matched analysis to resolve clinical trial controversies

### A De Jong, M Sebbane, G Chanques, S Jaber

#### Montpellier University Hospital, Montpellier, France

**Introduction**

In the obese intensive care unit (ICU) population, the association between prognosis and category of admission (medical vs surgical) remains unknown. The primary objective of this study was to determine the short and long term mortality of obese ICU patients following medical as opposed to surgical admission.

**Methods**

A prospective, observational cohort study, using a propensity-score-matched analysis of patients with medical or surgical admission was conducted in a mixed medical-surgical ICU. All consecutive adult obese patients (body mass index (BMI) > =30 kg/m2) admitted during a 14-year period were included. The primary endpoint was ICU mortality rates. Impact of category of admission on mortality was analyzed.

**Results**

**Fig. 47 (abstract P340).**
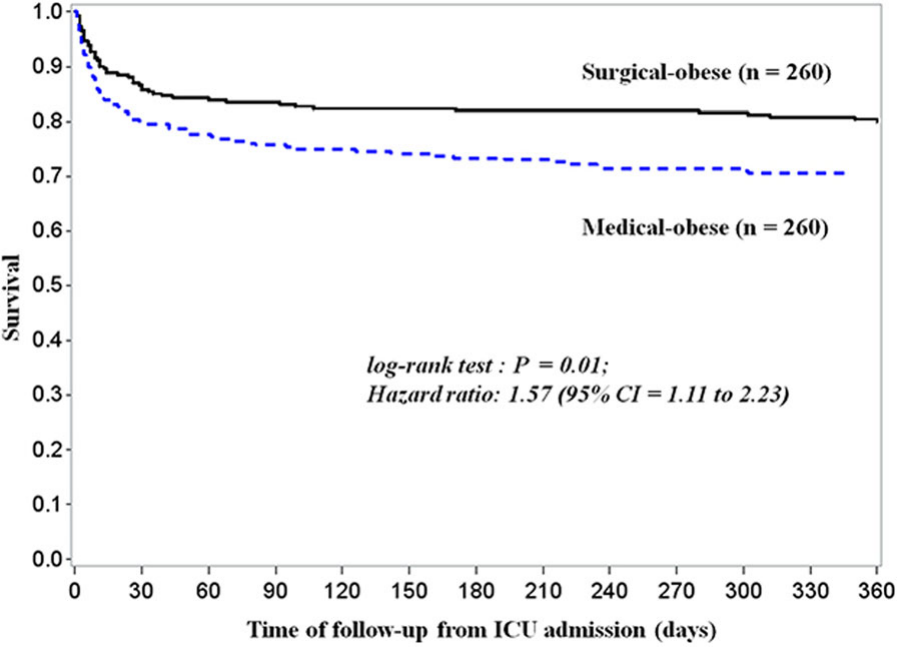
See text for description

5,435 patients were included: 4,864 (75%) patients non-obese (data not shown) and 791 (12%) obese patients, including 338 (43%) medical and 453 (57%) surgical patients. Mortality was significantly higher in medical than in surgical patients in ICU (25%vs12%, P < 0.001) and up to 365 days (36%vs18%, P < 0.001) post ICU admission. One-to-one propensity-score matching generated 260 pairs with well-balanced baseline characteristics. After matching on propensity score, mortality was still significantly higher in medical patients both in the ICU (21%vs13%, P = 0.03) and up to 365 days (30%vs20%, P = 0.01) post ICU admission. Figure 47 shows the Kaplan Meier curve at one year after ICU admission in medical and surgical obese matched patients. The SAPS II predicted ICU mortality were 25% and 27% for an observed mortality of 21% (P = 0.30) and 13% (P < 0.001), respectively in medical and surgical patients.

**Conclusions**

After careful matching the data suggest that ICU mortality in obese population was higher in the medical group than in the surgical group and remains significantly higher 365 days post ICU admission.

## P341 The impact of comorbidities on functional status and quality of life among adult icu survivors: a prospective cohort study

### R Rosa, C Robinson, M Bessel, L Cavalheiro, L Madeira, W Rutzen, R Oliveira, J Maccari, M Falavigna, E Sanchez, F Dutra, C Dietrich, P Balzano, J Rezende, C Teixeira

#### Hospital Moinhos de Vento, Porto Alegre, Brazil

**Introduction**

Many ICU survivors have coexistent chronic diseases or comorbidities that have the potential to influence the rehabilitation outcomes of these patients. The purpose of this study was to evaluate the association between comorbidities and functional status and quality of life among adult ICU survivors.

**Methods**

A prospective cohort study was conducted in mixed clinical-surgical ICUs of 6 tertiary hospitals in Southern Brazil from March 2014 to October 2016. Adult patients who required a length of ICU stay >48 hours and were discharged alive from the ICU were followed. Comorbidities were assessed using self-report and verified by medical record review and the Charlson comorbidity index (CCI). Functional status and quality of life were measured by the Barthel index of daily living and the SF-12v2 physical and mental health summary scores, respectively. Additional outcomes included all-cause mortality and any hospital readmission during the follow-up. The association between the CCI and outcomes was performed using adjustment for relevant covariates.

**Results**

In total 399 patients were evaluated. The mean CCI of the study population was 2.6 (SD, 2.4). Higher CCIs were associated with lower scores of SF-12v2 physical health summary at 3 months (β, -1.16; 95%CI, -1.80 to -0.51, p < 0.001) and at 6 months (β, -0.92; 95%CI, -1.70 to -0.14, p = 0.01) after discharge from ICU. The CCI was found to be a predictor of hospital readmission within 6 months after discharge from the ICU (RR, 1.13; 95%CI, 1.03 to 1.24, p = 0.004). The CCI wasn’t associated with SF-12v2 mental health summary score or the Barthel index of daily living at 3 and 6 months after ICU discharge, and all-cause mortality.

**Conclusions**

The weight of comorbid conditions measured by the CCI play an important role in quality of life and ICU readmissions rates after discharge from ICU.

## P342 Practice pattern of end of life care plan in a tertiary ICU from India

### S Sinha, K Majhi

#### Apollo hospitals, bhubaneswar, India

**Introduction**

Policy on end of life care has been unclear and debated for long time in India. There are certain guidelines in place for care of the dying. [1] We see considerable indecisiveness among the treating physicians and family members when it comes to discuss about futility of care and limiting therapy for a terminally ill patient.

**Methods**

We conducted a prospective study to analyse the end of life (EOL) issues in our centre. It is a semi-closed 30 bedded ICU. After a consensus between intensivist and primary team, 29 patients deemed appropriate for EOL care were included in the study. Families who disagreed for EOL plan were put in group A and who agreed were included in group B. The time to initiate EOL discussion, billing category, the initiating team, response of the next of kin and outcome were noted. A meeting is held between the medical teams, an administrative staff and family members. The decision is signed by all and plan is documented. EOL is to be done by withdrawal or non escalation of ongoing treatment ensuring comfort care. There is no provision of dedicated family counsellor or palliative care team in our centre. Withdrawal means extubating a patient who is on ventilator and giving non invasive ventilation or plain oxygen and stopping vasopressors if any. Non escalation was achieved by not augmenting the level of support either ventilator, vasopressor or medicines. Billing was either by cash or by insurance cover.

**Results**

The average time to initiate such a discussion was around 8.1 days. In 25 out of 29 (86.20%) cases it was initiated by intensivists (P value < 0.0001,CI-0.694-0.945).Families o f20 patients did not agree to EOL plan(group A). Among 9 cases who agreed (group B), none agreed for withdrawal of life support. They opted for non-escalation of ongoing treatment and DNR (do not resuscitate) orders in case of eventuality. Reasons for disagreement with EOL plan were emotion, cultural belief and lack of agreement among family members. Seventeen patients had health insurance cover while only 12 cases were paying by cash (p value 0.0946,CI-0.255-0.593). In group A,15 patients died while 5 were in vegetative state at the time of discharge from ICU with no decision of limitation of therapy while in group B, all 9 patients died (p = 0.13,CI-0.58-0.96). Average length of stay in group A and B were 30.8 and 14.22 days respectively (p <0.005). The average bill amount was USD 9763.23 in group A while it was USD 7591.17 in group B.

**Conclusions**

Valuable resources and cost can be saved by improving practice of EOL care. It can be better accomplished by combined effort of ICU team, primary physicians, dedicated family counsellor and palliative care team.

**References**

1. Myatra SN, Salins N, Iyer S, Macaden SC, Divatia JV et al. End-of-life care policy: An integrated care plan for the dying.Indian J Crit Care Med 2014;18:615–35

## P343 Is it possible and relevant to involve more the general practitioner in the decisions of withholding and withdrawing of treatments in intensive care units?

### JG Gorlicki^1^, FP Pousset^2^

#### ^1^Hôpital Lariboisière, Paris, France; ^2^Hôpital Pitié-Salpêtrière, Paris, France

**Introduction**

Withholding and withdrawing of life-sustaining treatments (WWT) stands as an answer to the unreasonable obstinacy arising from modern medicine [1]. According to the law, it is set up after a collegial discussion for which the inclusion of the family general practitioner (GP) is recommended.[2] GPs usually know the general conditions of their patients and are used to the management of ends-of-life at home. Several studies show that only 20% of them are contacted in case of WWT for one of their hospitalized patients.[3] We aimed to see if it was feasible and relevant to involve them more often.

**Methods**

We conducted a monocentric prospective study in the intensive care unit (ICU) of the Sud-Francilien hospital near Paris, where we recruited during a 4 months period every patients for whom a WWT decision was forecasted. We designed a systematic-call protocol to contact the GP in each case. For each patient, we determined whether the GP has been reached and if his opinion had an impact on the ethic discussion.

**Results**

Between April and July 2016, 28 patients were included. The GP were reached in 24 cases (86%), and for 16 of them (57%) it was done before the ethic discussion. They had an opinion in favor of a WWT in 14 cases (58%), against it in 3 cases (12%) and didn’t give their opinion in 7 cases (29%). During the ethic discussion, the influence of this opinion on the decision and the interest of receiving it were mostly rated « not at all » (67% and 60%).

**Conclusions**

The setting up of a protocol leads therefore to an increase rate of GP whose opinions are collected in perspective of a WWT decision. However, in order to make this consultation fully relevant, there are some improvements to be made, regarding the collaboration between intensivists and GP, the setting up of protocols in the ICU, the training of the GP to the WWT decisions and the enhancing of advance directives in general practices.

**References**

1. Ferrand E, Robert R, others. Withholding and withdrawal of life support in intensive-care units in France: a prospective survey. The Lancet. 2001

2. EPILAT study group, Lesieur O, Leloup M, Gonzalez F, Mamzer M-F. Withholding or withdrawal of treatment under French rules: a study performed in 43 intensive care units. Ann Intensive Care 2015 Dec

3. Ferrand E, et al. Participation of French general practitioners in end-of-life decisions for their hospitalised patients. J Med Ethics. 2006 Dec

## P344 Improving end of life care in the intensive care unit – the follow up study

### J Kelly, J Aron, A Crerar Gilbert

#### St George's Hospital, London, United Kingdom

**Introduction**

Ongoing organ support for dying patients may not be in their best interest. The decision to limit or withdraw active treatment for patients in the intensive care unit (ICU) is often complex and emotive [1]. This study aimed to evaluate the ICU multi-disciplinary team (MDT) experience of managing patients with treatment limitations, and to identify ways to improve patient care, family experience and MDT efficacy. A proforma was introduced to aid communication and decision-making during this difficult time.

**Methods**

ICU MDT members completed an anonymous questionnaire detailing their personal experiences of managing end of life patients. Responses were analysed with quantitative and qualitative methods. Based on results a proforma was designed and implemented. Nine months later a follow up questionnaire was conducted to evaluate the impact of the proforma.

**Results**

In the first questionnaire 40 responses were obtained. All staff felt that decisions about treatment limits and withdrawal were not always effectively communicated; 69% of junior staff and 38% of senior staff stated that MDT communication was ‘below ideal’.

Thematic analysis identified several issues. These included the sole use of verbal communication, unclear documentation, a lack of specific guidance, and inconsistency with frequent rotation of medical staff. When instructions were unclear junior staff, less experienced decision-makers, would practice caution and sometimes continue inappropriate treatments.

Respondents described times where they felt miscommunication had limited the support they could offer patients and families. They reported feeling upset, frustrated and embarrassed due to a lack of information. This led to a feeling that they were not providing the standard of care they desired.

The follow-up questionnaire showed overwhelming support for the proforma. Staff agreed it improved their experience of providing end of life care, MDT communication and quality of patient care. Key areas included clear and easily found documentation, early planning of possible treatment withdrawal and providing a useful tool to approach family discussions.

**Conclusions**

End of life care is vital for patients and their families. Before proforma implementation, staff felt that communication was sub-standard and led to personal and professional difficulties. Afterwards staff felt their experience of providing end of life care was significantly improved, due to better communication, clearer documentation and early planning of care.

**References**

Wilkinson DJC et al. Current Opinion In Anaesthesiology 24:160-5, 2011.

## P345 Stop treating does not mean stop caring - beyond withholding and withdrawing

### N Prevec Urankar, R Knafelj

#### Rihard Knafelj, Ljubljana, Slovenia

**Introduction**

Ethical committee approved forms for treatment withholding and withdrawing used at University Medical Clinic Ljubljana was introduced in our ICU in April 2015. We report and discuss its 10 months’ implementation use

**Methods**

Consecutive patient data were analyzed in period between 13/04/2015 and 29/02/2016 (implementation period)

**Results**

737 patients were admitted to ICU during reported period. For 66 patients (8.9%) decision for palliative care was made. In 48 patients (6.5%) decision to withhold and in 4 to withdraw the treatment was made. Patients were daily reevaluated. 14 patients from withholding group were reallocated to withdrawing group (total 2.4%). Average patient age was 69 ± 15 years (range 38-89). 44 patients remained in our ICU, 22 were transferred to regional hospitals or general wards. All patients remaining in MICU died (24 h-21 days after palliative plan implemented, mean 48 h). Of 22 patients transferred to regional hospitals, 20 died (mean 14 days, range 24 h-83 days), 2 were discharged to long term care facility. Terminal weaning was not performed in any patient. Antibiotics and vasopressors were most commonly withdrawn and withheld drugs. Morphine infusion was most commonly used sedative/analgesic

**Conclusions**

64 out of 66 patients in palliative care died while hospitalized. Patients transferred to regional hospitals died latter compared to patients staying in ICU. Possible reasons include medical personnel unease with withholding/withdrawal concept and avoiding the discussions with family members. We advocate early and open discussion with family members and care givers about implementing palliative care in ICU

**References**

R. Knafelj, M.Fister. Octagenerians in medical icu-adding days to life or life to days? Critical Care2016;20(Suppl2):94

**Fig. 48 (abstract P345).**
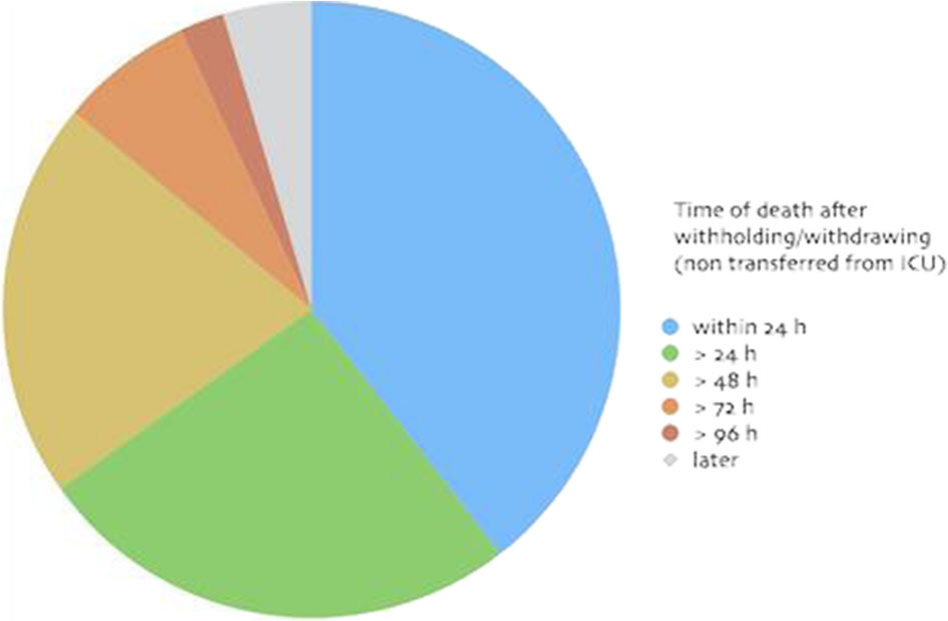
See text for description

**Fig. 49 (abstract P345).**
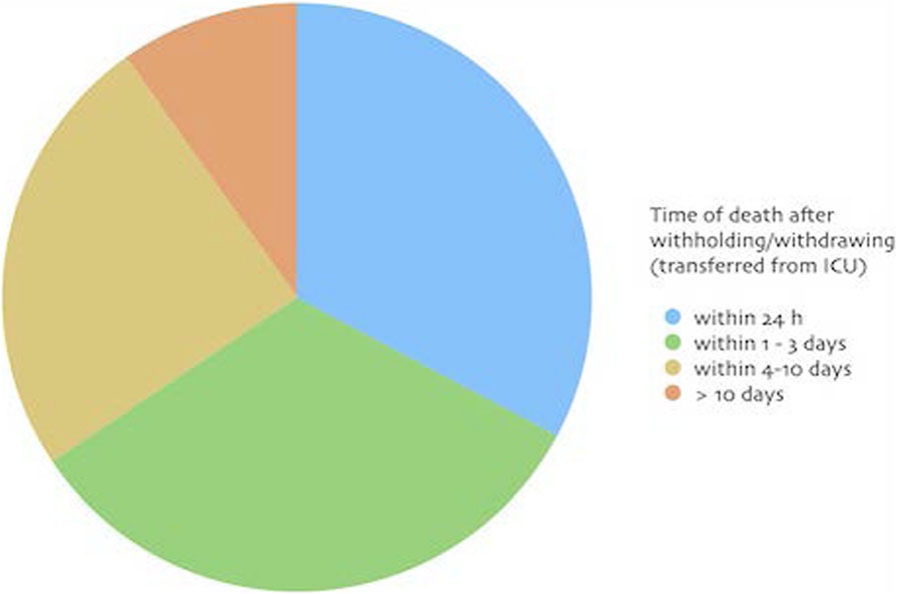
See text for description

## P346 Targeted application of the organ donation European quality system (odequs) in a third level Catalan public hospital (hgtip) compared to private one (hgc) with an active dbd program.

### M Irazabal^1^, M Bosque^1^, J Manciño^2^

#### ^1^Hospital General de Catalunya, Barcelona, Spain; ^2^Hospital Germans Trias i Pujol, Barcelona, Spain

**Introduction**

The aim of this study was to apply the key indicators proposed in the ODEQUS system to verify if achieved quality criteria in the donation process (DP). The HGTIP and HGC are both third level Catalan hospitals (with a total of 500 and 300 beds respectively), with a Neurosurgery Department that covers over a million population area. The proportion of critical care units for medical and postoperative care patients is HGTIP/HGC 2:1. The donation team (DT) includes a transplant coordinator (TC) with 24 hour availability and part time dedication.

**Methods**

We performed a retrospective analysis focused on the DP after brain death (DBD). We reviewed the 17 quality criteria listed in the ODEQUS project to select 6 indicators useful to apply, based on the donation activity reported to the OCATT in 2015. We also considered all 3 aspects in the DP: structure (S), procedures (P) and outcomes (O). Data was obtained from DT registries and medical records from each hospital. We applied the proposed formula for:

1) Proactive donors identification protocol (S1)

2) DT members with ICU background (S2)

3) Identification of all possible donors in ICU (P1)

4) Referral of possible DBD donors (P2)

5) Family consent (O1)

6) Conversion rate in DBD (O2)

**Results**

Regarding to proactive donors identification there was no registered any limitation in HGTIP compared to HGC (even in the absence of a formal protocol available outside the ICU in the last one). All members of the DT in both centres are intensivists with more than 10 years’ experience in the DP. All patients with devastating cerebral lesion (DCL) admitted in the ICU (GCS < 8) who also met the DBD criteria were referred to the DT. There was not detected any family refusals. All eligible donors were correctly identified and converted into actual donors.

**Conclusions**

Related to the identification protocol it is mandatory to involve all the departments that also represents a source of potential donors. It is essential to designate a responsible person to facilitate communication among professionals to avoid the leakage of donors at all hospital levels. Every cause of refusal should be properly documented after applying the ODEQUS methodology in order to create improvement measures enhancing the efficiency in the DP in BCN.

**References**

Manyalich M. et al. "ODEQUS: Organ Donation European Quality System". Universitat de Barcelona. Executive practice of Health and Consumers. October 2010 - December 2013.

## P347 A national survey of controlled DCD donors not progressing into circulatory death within 120 minutes

### A Kotsopoulos^1^, N Jansen^2^, W Abdo^3^

#### ^1^Elisabeth Tweesteden Hospital, Tilburg, Netherlands; ^2^Dutch Transplant Foundation, Leiden, Netherlands; ^3^Radboud UMC, Nijmegen, Netherlands

**Introduction**

One out of five (18%) initiated Donations after Controlled Circulatory Death (cDCD) procedures does not result into organ retrieval because circulatory death does not ensue within the predefined time of warm ischemia. The main aim of this study is to determine the characteristics of these cDCD donors.

**Methods**

We conducted a four year (2012-2015) nationwide retrospective observational cohort study on all 146 cDCD donors entering the donor procurement protocol, not having a circulatory death within 120 minutes after withdrawal of life-sustaining treatment (WLST). Data were obtained both from the database of The Dutch Transplant Foundation and concomitant review of the medical files. We compared this group with a previous derived dataset of cDCD donors arresting within 60 minutes after WLST. Univariate and multivariate logistic regression analyses were performed.

**Results**

There were no differences concerning age, gender, body mass index or APACHE IV score between groups. More patients in the >120 group suffered from post-anoxic encephalopathy OR 9.29[95% CI 3.11-27.6].Traumatic brain injury and subarachnoid hemorrhage were significantly more prevalent in the <60 minutes group; OR 2.39 [95% CI 1.22-4.68] and 4.26 [95% CI 1.90-9.55], respectively. Absence of brainstem reflexes and a Glascow Coma Scale of 3 were associated with death within 60 minutes. However, 49% of patients dying after 120 minutes had also a GCS of 3. Norepinephrine was administered in 35% of patients in the >120 minutes group and in 32% of patients in the <60 minutes group (p = 0.769). Multivariate logistic regression analysis with forward selection showed that the diagnosis of post-anoxic encephalopathy was associated with death after 120 minutes [AUC of 0.878, [95%CI 0.757-1.00].

**Conclusions**

The absence of brainstem reflexes could predict time to death in potential cDCD donors. Interestingly, the diagnosis of post-anoxic encephalopathy in cDCD donors, is strongly associated with circulatory death >120 minutes.

## P348 An analysis of 10 years of organ donation in Cork University Hospital

### ÚM Casey, B O'Brien, R Plant, B Doyle

#### Cork University Hospital, Cork, Ireland

**Introduction**

In Ireland, data from each patient who becomes an organ donor is routinely collected for a national database. This study reviews data collected from each organ donor, at a single centre, over a period of 10 years. We compare the average number of organs donated per patient before and after the introduction of guidelines on the use of pituitary hormone replacement therapy.

**Methods**

We obtained data for each donor, declared brain dead in the ICU at Cork University Hospital, over a 10 year period from 2006-2015. Each patient’s record was assessed for 1) donor age; 2) cause of death, 3) number of organs donated; and for those patients who became donors after 2010, 4) use of pituitary hormone replacement therapy.

**Results**

90 patients donated their organs, after the declaration of brain death, over the 10 year period reviewed, an average of 9 donors per year (range 1–13). The average donor age was 41 years and 4 months (range 8 months - 71 years). Subarachnoid haemorrhage (33/90 – 37%) and traumatic brain injury (37/90 – 41%) were the two most common causes of death. The causes of death recorded for the remaining patients included intracerebral haemorrhage; ventriculo-peritoneal shunt blockage; arterio-venous malformation and stroke.

Over the 10 years, the average number of organs donated per patient was 3.7 (range 0 – 6). An average of 3.5 organs per patient were donated in the years prior to the introduction of pituitary hormone replacement therapy in 2010. The average number of organs donated per patient from 2011 was 3.8, a marginal increase of 8.5%. Donor numbers increased slightly from 2011, with 42 patients (8.4 per year, range 1 – 13) until 2010, and 48 patients, (9.6 per year, range 8-12) thereafter.

**Conclusions**

Each of the 90 patients who donated organs in CUH suffered an intra-cerebral injury which led to their death. The introduction in May 2010, by the Intensive Care Society of Ireland, of guidelines for the use of pituitary hormone replacement therapy in potential donors [1], appears to have resulted in only a modest increase in the number of organs retrieved per donor. However, the averages presented here are basic. In order for the full impact of the use of hormone replacement to be assessed, it would be prudent to further assess each individual patient, and their medical history, for pre-existing conditions or new trauma which would preclude the donation of individual organs. Work is ongoing by the authors to fully ascertain the impact of hormone replacement on overall numbers of organs donated.

**References**

Diagnosis of Brain Death & Medical Management of the Organ Donor. Guidelines for Adult Patients 2010. Intensive Care Society of Ireland.

